# Recorded and predicted occurrence of slime moulds (Eumycetozoa) in Poland from Central and Eastern European data

**DOI:** 10.7717/peerj.21492

**Published:** 2026-07-09

**Authors:** Tomasz Pawłowicz

**Affiliations:** Institute of Forest Sciences, Faculty of Civil Engineering and Environmental Sciences, Bialystok University of Technology, Białystok, Podlaskie, Poland

**Keywords:** Eumycetozoa, Myxobiota, Species checklist, Species distribution modelling, Potential richness, Poland, Central and Eastern Europe, Biodiversity assessment, Forest bioindication

## Abstract

**Aim:**

To quantify recorded and potential (model-based) occurrence of slime moulds (Eumycetozoa) in Poland within a harmonised Central and Eastern European (CEE) framework, and to provide a reproducible basis for targeted survey planning and biodiversity assessment.

**Location:**

Poland, with a comparative regional context based on harmonised occurrence data from 16 countries in Central and Eastern Europe.

**Methods:**

We used a taxonomically standardised, georeferenced Darwin Core dataset of Eumycetozoa compiled for 16 CEE countries. A Polish national checklist was assembled and annotated with habitat, substrate and national bibliographic information. We delineated an additional set of candidate taxa from the wider CEE pool and, where data permitted, evaluated them using presence-only species distribution models to estimate broad-scale climatic suitability in Poland and to identify regions and taxonomic groups concentrating potential diversity.

**Results:**

The Polish checklist comprised 278 species in six orders, strongly dominated by Physarales, Trichiales, Stemonitidales and Cribrariales, with known records spatially clustered in a limited set of well-surveyed regions. Comparison with the regional pool indicated 365 additional candidate taxa in 11 orders not yet recorded from Poland. Of these, 101 species could be modelled, and 31 achieved medium or high model-based suitability within Poland. Adding these 31 taxa to the recorded checklist yields a conservative estimate of 309 species likely to occur nationally, whereas including all candidates provides a theoretical regional maximum of 643 taxa. Model predictions highlighted western and northern voivodeships as concentrating suitable climatic conditions for multiple candidate species.

**Conclusions:**

Harmonised occurrence data, an annotated national checklist and model-based predictions together indicate substantial survey gaps and a considerable pool of likely yet unrecorded taxa in Poland. These outputs provide a transparent, reproducible framework to prioritise future fieldwork, fill spatial and taxonomic gaps, and support the development of slime moulds as biodiversity indicators for forests in Poland, with regional climatic priorities to be validated against forest type, substrate availability and survey-time weather.

## Introduction

Slime moulds (Eumycetozoa) form a monophyletic lineage within Amoebozoa that comprises three principal groups: Myxogastria (traditionally “myxomycetes”), Dictyostelia and Protosporangiida (Adl et al., 2012; Adl et al., 2019; Tekle, Lahr & Katz, 2022). Molecular and ultrastructural evidence has clarified their separation from fungi and other protists, and their life cycles are characterised by an alternation between microscopic trophic stages and macroscopic sporocarps (Stephenson & Stempen, 2000; Stephenson, Schnittler & Novozhilov, 2008). In temperate forests, well-studied Myxogastria forage as amoeboid and amoeboflagellate cells within moisture-buffered substrates such as coarse woody debris, bark, bryophyte mats and leaf litter, and form sporocarps when conditions allow (Stephenson & Rojas, 2017; Stephenson, 2023). By grazing on bacteria and other microorganisms, Eumycetozoa help to structure detrital food webs and contribute to nutrient turnover in dead organic matter (Stephenson & Rojas, 2017; Keller et al., 2008). This study followed the Eumycetozoa classification of Lado and Eliasson (Lado & Eliasson, 2025).

Because their trophic stages depend on humid, shaded and porous substrates, slime mould assemblages (myxobiota) are expected to respond to forest structure, microclimate and substrate continuity (Leontyev et al., 2019; Leontyev & Schnittler, 2017). Lignicolous communities on dead wood, corticolous assemblages on living bark, litter-dwelling consortia and bryophilous taxa on moss cushions can differ markedly in composition, and shifts in tree species, dead-wood availability or canopy closure may be reflected in changes in Eumycetozoa assemblages (Stephenson & Stempen, 1994; Fukasawa et al., 2015; Wrigley de Basanta & Estrada-Torres, 2017). These properties, together with the relative ease of recording sporocarps in the field or *via* moist-chamber cultures, have led to growing interest in slime moulds as ecological indicators in forest ecosystems (Stephenson, Schnittler & Novozhilov, 2008; Baba & Sevindik, 2023). At the same time, Eumycetozoa have negligible impact on crop productivity or tree health and are therefore better framed as biodiversity indicators than as pests or pathogens.

Despite this potential, large-scale syntheses remain rare for Central and Eastern Europe (CEE). Much of the available knowledge is embedded in local or national checklists and inventories, which differ in sampling design, nomenclatural treatment and spatial resolution (Drozdowicz, 2003a; Drozdowicz, 2003b; Paul, Janik & Ronikier, 2023; Pawłowicz et al., 2025). For Poland, published records demonstrate that true slime moulds are widespread and taxonomically diverse, but they provide only a partial and spatially uneven picture of national diversity. Heterogeneous protocols and incomplete georeferencing have hindered robust comparisons across regions, limited the ability to quantify spatial gaps in sampling and constrained efforts to use slime moulds operationally in biodiversity assessment or bioindication (Pawłowicz et al., 2025). To date, no national synthesis has combined recorded and potential components of the Polish myxobiota within a harmonised regional context.

Recent harmonisation efforts have begun to address these limitations by assembling Darwin Core–mapped, georeferenced occurrence archives for Eumycetozoa in CEE, standardising taxonomic usage and consolidating environmental descriptors relevant to forests (Pawłowicz, 2025). Such resources provide a common framework within which national checklists can be constructed, spatial coverage can be quantified consistently, and candidate taxa can be identified across political borders. They also create the preconditions for applying species distribution models (SDMs) based on presence-only data, allowing environmental suitability to be projected from regional occurrences to unsampled parts of a country (Phillips, Anderson & Schapire, 2006; Elith et al., 2011; Franklin, 2010). For relatively well-sampled but incompletely documented groups such as Eumycetozoa, this combination of harmonised occurrences and SDMs offers a way to link recorded and potential components of diversity and to prioritise field validation. Because substrate availability, forest structure and short-term weather act as local filters on realised records, the SDMs used here are intended as broad regional screening tools rather than direct stand-level predictors of occurrence.

From a Polish perspective, two complementary questions arise. First, given a harmonised regional dataset, how many Eumycetozoa species are currently recorded from Poland, how are they distributed among orders and how heterogeneous is their spatial coverage at a national scale? Second, what can occurrences from neighbouring CEE countries tell about additional taxa that may plausibly occur in Poland but have not yet been reported, and about the regions where environmental conditions are most favourable for these candidate species? Addressing these questions is necessary both to place the Polish myxobiota in a broader regional context and to provide an explicit baseline for targeted surveys, conservation planning and the future use of slime moulds in ecological assessment.

To address these questions, a harmonised, georeferenced Darwin Core dataset for 16 CEE countries was combined with existing Polish records to compile a taxonomically standardised national checklist, to characterise spatial patterns of recorded richness and composition by order, and to identify candidate species from the regional pool that were not yet documented from Poland. Occurrence coordinates from the regional dataset were then linked to gridded bioclimatic variables to model climatic suitability for candidate taxa and to summarise their likely distributions among Polish voivodeships. By integrating recorded and modelled components of the myxobiota, the study identified taxonomic and regional priorities for future work on Eumycetozoa in Poland and the wider CEE region.

## Materials & Methods

### Dataset and taxonomic framework

Analyses were based on a georeferenced Darwin Core checklist and occurrence dataset of slime moulds (Eumycetozoa) from 16 Central and Eastern European countries, with presence–only records spanning 1857–2025 and explicit spatial and contextual metadata (Pawłowicz, 2025). Coordinates were stored as *decimalLatitude* and *decimalLongitude* in WGS 84. Contextual fields included *country*, taxonomic ranks from species to order, *minimumElevationInMeters*, *maximumElevationInMeters*, climatic descriptors, forest type descriptors, a consolidated habitat class, a management intensity class, and substrate and microhabitat fields. A midpoint elevation variable, *elevationMid*, was computed as the mean of *minimumElevationInMeters* and *maximumElevationInMeters*, and a three-level elevation band *elevationBand* (<300 m, 300–1,000 m, >1,000 m) followed the original data paper (Pawłowicz, 2025). Taxonomic usage followed the nomenclature in that resource, ensuring consistent mapping between scientificName and higher ranks. The classification and nomenclature of Lado & Eliasson (2025) were adopted, with order-level assignments harmonised accordingly, infraspecific varieties retained where applicable, and the GBIF Species Backbone (GBIF Secretariat, 2023) used only as a fallback for unresolved names or higher-taxon placement. For each name encountered in the Polish and regional literature, we verified the accepted name, basionym, synonymy and nomenclatural status in nomen.eumycetozoa.com before compiling the checklist. Records published under historical combinations, replacement names or later synonyms were consolidated under the accepted name used here. This procedure standardised nomenclature and lineage placement, but it did not constitute de novo re-identification of every underlying collection or observation, nor specimen-by-specimen voucher review; the checklist therefore retains source determinations after nomenclatural reconciliation, while recognising that some original identifications may remain uncertain. Where recent studies indicate cryptic diversity within a morphologically delimited species, historical records were not redistributed among putative hidden lineages unless a published taxonomic revision or diagnostic evidence supported that transfer; such records were retained under the accepted species concept applied here (Shchepin et al., 2022).

### Computational environment and data assembly

All processing was implemented in R (R Core Team, 2024). Core packages were *data.table* for efficient handling of large tables (Barrett et al., 2024), *dplyr* and related tools for data manipulation (Wickham et al., 2019), *tidyr* for reshaping (Wickham & Girlich, 2022), *sf* for vector spatial data (Pebesma, 2018), *terra* for raster data (Hijmans, 2025), *geodata* for climatic data access (Hijmans, 2023) and *ggplot2* for graphics (Wickham, 2016). At the start of each run, the script checked package availability, attached them and recorded R and package versions.

The Darwin Core export was read under UTF–8 encoding. Character fields were trimmed, multiple internal spaces were collapsed and empty strings were converted to missing values. Temporal, spatial and elevation fields (year, decimalLatitude, decimalLongitude, minimumElevationInMeters, maximumElevationInMeters) were coerced to numeric types. The genus was derived as the first word of scientificName where present. A coordinate validity flag identified records with decimalLatitude ∈ [−90, 90] and decimalLongitude ∈ [-180, 180]; no records were removed at this step. These operations standardised formatting and identified obvious coordinate anomalies, but they did not independently verify the biological correctness of each occurrence record. The analyses therefore assumed that the retained records were sufficiently reliable in taxonomic identification and geographic position for broad-scale checklist synthesis and climatic modelling.

The Polish subset was obtained by filtering records with *country* equal to Poland, retaining both georeferenced and non-georeferenced records. A Polish species checklist was constructed as all distinct combinations of *order*, *genus*, *species* and *scientificName* in this subset. An analogous taxonomic index for the full Central and Eastern European (CEE) dataset was constructed from distinct combinations of these fields in the complete table. Both checklists were ordered by higher taxonomy.

### Polish checklist: ecological annotation and spatial summaries

For the Polish checklist, mandatory fields were order, genus, species, scientificName and associatedReferences. Candidate macrohabitat fields (*e.g.*, forestTypeDetailed, habitat, macrohabitat) and candidate substrate fields (*e.g.*, microhabitat, substrateCategory, substrate) were identified by name and intersected with the available columns; where no such fields were present, the corresponding summaries were left missing. For macrohabitat, values in the selected columns were aggregated by scientificName after reshaping to long format, discarding empty entries and concatenating distinct descriptors into a single semicolon-separated string macrohabitatRaw per species. Substrate descriptors were treated identically, yielding substrateRaw for each species. Because the source vocabulary of habitat, substrate and microhabitat fields varied among publications and datasets, these descriptors were retained as aggregated raw strings rather than recoded into a single standardised ecological classification. They were used to annotate the checklist and summarise ecological context, not as predictive variables in the SDMs. For Polish bibliographic provenance, associatedReferences values were grouped by scientificName, cleaned of empty strings and exact duplicates, and concatenated into references. Before these bibliographic strings were aggregated, records published under historical synonyms or superseded combinations were reassigned to the accepted scientificName adopted in the checklist. The Polish species checklist (distinct order–genus–species–scientificName combinations) was then joined with macrohabitatRaw, substrateRaw and references by scientificName, producing a national checklist with taxonomic placement, raw ecological descriptors and collated Polish references, with the references field in Table S1 recording the bibliographic provenance of checklist records and source publications used only for this purpose listed in Further Reading. These annotated records were merged with a pre-existing list of species recorded in Poland to yield a single consolidated national checklist, ordered by order, genus, species and scientificName.

For spatial summaries, Polish records with valid coordinates were converted to an *sf* point object in WGS 84 and intersected with a polygon of Poland derived from Natural Earth *via rnaturalearth* (South, 2017; Natural Earth, 2024). Points outside the national polygon were excluded. For area-based summaries, both polygon and points were reprojected to the official Polish equal-area coordinate reference system (EPSG:2180). An equal-area grid of 10 km × 10 km cells was generated and intersected with the national polygon, and each georeferenced record was assigned to a grid cell. Observed species richness per cell was calculated as the number of distinct *scientificName* values. Richness by order in the observed Polish myxobiota was derived as the number of checklist species in each *order*. Observed richness was summarised on a 10 × 10 km equal-area grid over Poland, whereas species distribution model projections and cumulative suitability maps were computed on the coarser 10-arc-minute WorldClim grid, so these maps were not directly comparable at the level of individual grid cells. Consequently, comparisons between observed richness and model-based suitability were summarised at the voivodeship level.

### Candidate species identification and ecological characterisation

Candidate species were defined as taxa present in the 16-country Central and Eastern European dataset but absent from the Polish checklist. The regional species pool was obtained as all distinct combinations of *order*, *genus*, *species* and *scientificName* in the full dataset. A set of Polish taxa was derived from unique *scientificName* values in the national checklist; candidate taxa were those regional species whose *scientificName* did not occur in this set. Among the 365 candidate taxa identified in this way, 336 (92%) were recorded from at least one country bordering Poland (Belarus, Czech Republic, Germany, Lithuania, Russia, Slovakia or Ukraine), supporting their biogeographic plausibility as potential additions to the Polish myxobiota, whereas the remaining 29 candidates were known only from more distant parts of the region.

Regional occurrence context for candidates was computed by filtering the full dataset to these taxa, grouping by scientificName and concatenating distinct non-empty country values into a semicolon-separated field countriesPresent. Candidate occurrence records were used to collate bibliographic data by grouping associatedReferences by scientificName, removing empty strings and exact duplicates, and concatenating remaining entries into associatedReferencesAll. As in the Polish checklist, records traceable to historical synonyms or alternative combinations were first mapped to the accepted scientificName before these species-level summaries were compiled. Macrohabitat and substrate descriptors for candidates were aggregated using the same column selection and long-format approach as for the Polish checklist: distinct non-empty macrohabitat descriptors per scientificName were concatenated into macrohabitatRaw, and distinct substrate and microhabitat descriptors into substrateRaw. If no macrohabitat or substrate columns were available, the corresponding species-level field was set to missing. These fields were retained only as species-level ecological annotations. Joining the candidate taxonomic table with countriesPresent, associatedReferencesAll, macrohabitatRaw and substrateRaw yielded a candidate ecology table with one row per candidate taxon. Of the 365 candidate taxa, 101 species with at least five unique occurrence locations were retained for species distribution modelling, and the remaining 264 species lacked sufficient or usable records and were treated as unmodelled candidates.

### Environmental predictors, collinearity reduction and background sampling

Environmental covariates for species distribution models (SDMs) were assembled as a raster stack of WorldClim 2.1 bioclimatic variables at 10-arc-minute resolution obtained *via* geodata (Fick & Hijmans, 2017; Hijmans, 2023) and combined into a multi-layer raster in WGS 84. The modelling domain was defined from candidate occurrences by filtering to records with valid coordinates, taking minima and maxima of decimalLongitude and decimalLatitude, adding a fixed buffer in all directions and cropping the environmental stack to this extent. All models were fitted and projected on WorldClim 2.1 bioclimatic variables at 10-arc-minute resolution, which corresponds to grid cells of approximately 18–19 km in the north–south direction and 11–12 km in the east–west direction at the latitude of Poland. Although the occurrence data included descriptors of forest type, habitat, management and substrates, these variables were heterogeneous across countries and among source records and were not available as consistent gridded covariates for the entire study region, so SDMs were fitted using only the reduced set of WorldClim 2.1 climatic predictors. This avoided treating semantically overlapping or differently recorded habitat and substrate fields as directly comparable model inputs. At this scale, the retained bioclimatic variables were interpreted as proxies for the broad temperature and moisture regime, seasonal variability and climatic extremes that influence how often wood, bark, litter and bryophyte substrates remain sufficiently moist for trophic activity and sporocarp development, and that also covary with the regional distribution of forest environments (Stephenson & Rojas, 2017; Stephenson, 2023). They therefore define a macroclimatic envelope within which suitable local habitats are more likely, but they do not describe the actual presence, continuity or quality of substrates in a given stand. Choropleth maps by voivodeship were derived by averaging or counting these raster predictions within each administrative polygon, rather than by changing the underlying grid resolution.

To reduce collinearity among predictors (Dormann et al., 2013), values of all layers in the cropped stack were sampled at up to 10,000 randomly selected cells with complete data. A Pearson correlation matrix was computed, and an iterative procedure removed variables with the highest mean absolute correlation until all pairwise absolute correlations among retained predictors were ≤ 0.7. The remaining layers constituted the reduced predictor stack used for SDMs.

Target-group background points were constructed from the full Eumycetozoa dataset to reflect the sampling pattern of the taxon group (Phillips et al., 2009; Barbet-Massin et al., 2012; Warton & Shepherd, 2010). Records with valid coordinates within the extent of the reduced predictor stack were deduplicated to unique coordinate pairs; up to a predefined maximum number were sampled at random. Environmental values at these locations were extracted from the reduced stack, and rows with missing values were discarded, yielding a background dataset with coordinates and complete predictors.

Candidate species were evaluated for modelling based on minimum unique occurrence locations. Candidate records with valid coordinates were deduplicated by rounding *decimalLongitude* and *decimalLatitude* to five decimal places and counting distinct coordinate pairs per *scientificName*. Species with fewer than five unique locations were excluded from modelling and retained only as unmodelled candidates, given the poor performance of SDMs at very small sample sizes (Wisz et al., 2008; van Proosdij et al., 2016). Species with at least five unique locations were carried forward to SDM fitting.

### Species distribution modelling, prediction to Poland and synthesis

Species distribution models were fitted for eligible candidate taxa using a maximum entropy (MaxEnt) presence–background framework (Phillips, Anderson & Schapire, 2006; Elith et al., 2011; Merow, Smith & Silander, 2013). For each species, environmental predictors were extracted at all unique presence locations from the reduced stack and combined with the target-group background data. A binary response indicated presences *versus* background. MaxEnt-type models were fitted using the maxnet package (Phillips, 2017), with linear, quadratic, product and hinge feature classes and the built-in regularisation to control overfitting (Phillips, Anderson & Schapire, 2006; Merow, Smith & Silander, 2013). Only predictors retained after collinearity reduction were used. The MaxEnt logistic output was treated as a relative index of modelled environmental suitability, rather than as a calibrated probability of occurrence. High values were therefore interpreted as indicating climatic similarity to the conditions represented by known regional occurrences, not confirmed local occupancy. Model evaluation employed k-fold cross-validation, with presence locations randomly partitioned into k folds and the same partitioning applied to background points. The number of folds was set to *k* = 5 where possible; otherwise, k equalled the number of presence locations for the species concerned. Both the area under the receiver operating characteristic curve (AUC) and a continuous Boyce index were attempted, but in the chosen implementation Boyce statistics (*boyceMean* and *boyceSd*) are undefined when the distribution of predicted suitability across evaluation bins lacks sufficient variation; under our settings they were therefore returned as NA for all species, so discrimination was assessed solely using cross-validated AUC.

After cross-validation, a final MaxEnt-type model was fitted per species using all presences and background. These models were projected onto the reduced environmental stack, and the resulting suitability maps were cropped and masked to the polygon of Poland (South, 2017); Natural Earth, 2024). For each species, the distribution of predicted suitability at all known occurrence locations in the Central and Eastern European dataset was used to define a single threshold equal to the 10th percentile of training-presence predictions, following recommendations for presence-only SDMs (Liu et al., 2005; Liu, White & Newell, 2013). Continuous predictions over Poland were then reclassified to binary suitability maps by applying this 10th-percentile threshold.

Within the Polish national boundary, three indicators were computed for each species: maximum suitability (*maxSuitabilityPL*), mean suitability (*meanSuitabilityPL*) and the proportion of area predicted suitable (*propAreaSuitablePL*). Voivodeship-level summaries were obtained by overlaying continuous and binary suitability maps with first-level administrative regions and calculating mean suitability and proportion of suitable area per region. For each species, up to three voivodeships with the largest proportion of suitable area (breaking ties by mean suitability) were recorded as top regions.

Model performance and suitability metrics were combined to assign each of the 101 modelled candidate species to one of three qualitative model-based climatic suitability classes for occurrence in Poland. These classes summarised climatic support for occurrence only and did not incorporate direct information on substrate availability, forest structure or short-term weather. Suitability classes (high, medium, low) were defined only for these 101 modelled candidate species, based on combinations of mean AUC and Polish suitability indicators. Species with high discrimination (mean AUC at least 0.80) and high suitability in Poland, defined as maxSuitabilityPL ≥ 0.60, meanSuitabilityPL ≥ 0.20 and propAreaSuitablePL ≥ 0.10, were assigned to a high-suitability class; species with intermediate AUC (at least 0.70) and moderate suitability and area indicators were assigned to a medium-suitability class; the remaining modelled species were assigned to a low-suitability class. These classification rules were fixed a priori and applied consistently. The 264 candidate species without SDMs were retained in the regional species pool without SDM-based suitability assignments and were labelled as not modelled.

Observed richness in Poland by order was calculated as the number of checklist species per *order*, and candidate richness by order as the number of candidate species per *order*. Because candidates were defined as taxa lacking Polish records, combined richness per order was the sum of observed and candidate counts. A cumulative hotspot map of candidate suitability in Poland was produced by summing continuous suitability maps of all modelled candidate species cell-wise, yielding a spatial index of the number of candidates for which conditions were predicted to be suitable (Franklin, 2010; Guisan & Zimmermann, 2000; Araújo & Guisan, 2006). In a final quality-control step, voivodeship names and order names were harmonised to a fixed set of 16 Polish regions and 11 target orders before recomputing richness by order and regenerating maps summarising the importance of voivodeships and the distribution of richness among orders.

## Results

### Harmonised occurrence data and Polish species list

The harmonised Central and Eastern European Eumycetozoa dataset used here contained 278 species recorded from Poland and 365 additional candidate taxa present elsewhere in the region, giving a regional pool of 643 species across 11 orders. This pool was treated as a theoretical maximum based on the regional species pool rather than as a model-derived estimate of Polish richness.

Cleaning produced a harmonised occurrence dataset with standardised character and numeric fields, an explicit *genus* column and a coordinate validity flag. Filtering this dataset to records assigned to Poland yielded a dedicated subset for analyses of national Eumycetozoa diversity. From this subset, a Polish species list was compiled as unique combinations of *order*, *genus*, species epithet and *scientificName*. The checklist comprised 278 species representing 53 genera in six orders. Species richness was uneven across orders: Physarales comprised 112 species, Trichiales 56, Stemonitidales 55, Cribrariales 50, Echinosteliales 3, and Ceratiomyxales 2 (Table S1). For the full Central and Eastern European dataset, a separate taxonomic lookup table collated all unique combinations of order, genus, species epithet, and scientificName, providing a reference for cross-checking taxonomic assignments.

### National checklist and ecological annotations

The Polish checklist (Table S1) comprised 278 species arranged in four taxonomic fields (order, genus, species, scientificName) and three derived summaries (habitatRaw, substrateRaw, references). The table reports currently accepted names only, with taxa placed in the current lineage and higher-rank framework adopted in ‘Dataset and taxonomic framework’; historical synonyms and superseded combinations from the underlying literature were reconciled to these accepted names during checklist assembly. Habitat information (habitatRaw) was available for 201 species, whereas 77 species lacked aggregated habitat descriptors. Substrate information (substrateRaw) was available for 230 species, with 48 species lacking a recorded substrate summary. Bibliographic coverage was complete, with non-empty references entries for all checklist rows.

For species with ecological information, *habitatRaw* and *substrateRaw* consisted of strings concatenating the distinct macrohabitat and substrate or microhabitat descriptions drawn directly from Polish records. Two entries lacked a populated species epithet while retaining a full *scientificName*; all other taxonomic fields were complete. The underlying record-level subset used to construct these summaries, containing the same taxonomic, ecological and bibliographic fields prior to aggregation, was exported separately as a supporting dataset.

### Spatial distribution of recorded richness and composition in Poland

Filtering for valid coordinates produced a spatially consistent subset of Polish Eumycetozoa records that could be represented as points within the national polygon in an equal-area projection. A regular 10 × 10 km grid was generated over the reprojected polygon of Poland, and each spatially valid record was assigned to a grid cell. Species richness per cell, defined as the number of distinct *scientificName* entries, was calculated and associated with each grid cell, with cells without records assigned richness zero. This provided a grid-based summary of recorded richness at 10 × 10 km resolution.

Using the national checklist (Table S1), richness by order confirmed the dominance of Physarales, Trichiales, Stemonitidales and Cribrariales in the recorded Polish myxobiota. These order-level summaries were calculated after nomenclatural harmonisation to accepted names, so historical synonyms did not inflate the observed richness counts. Because this observed richness surface was based on a 10 × 10 km equal-area grid, whereas species distribution model projections and cumulative suitability maps were computed on the coarser 10-arc-minute WorldClim grid, comparisons between observed and predicted patterns were restricted to the voivodeship scale and were not made at the level of individual grid cells.

### Candidate species from Central and Eastern Europe

Candidate species were defined as taxa present in the 16-country Central and Eastern European dataset but absent from the Polish checklist. Comparison of this regional dataset with the Polish species list yielded 365 candidate taxa whose *scientificName* was not recorded from Poland. Among these 365 candidate taxa, 336 (92%) were recorded from at least one country bordering Poland (Belarus, Czech Republic, Germany, Lithuania, Russia, Slovakia or Ukraine), supporting their biogeographic plausibility as potential additions to the Polish myxobiota, whereas the remaining 29 candidates were known only from more distant parts of Central and Eastern Europe. These candidate taxa spanned 11 orders and 64 genera and were collectively recorded from 14 countries in the region. Individual candidate species were documented from between 1 and 12 countries (median 2), with some countries, such as Germany and Russia, contributing particularly large numbers of candidate taxa.

All 365 candidate taxa were associated with at least one bibliographic source. Across species, the number of unique references per taxon, derived from the aggregated *associatedReferencesAll* field, ranged from 1 to 93, with a median of 4 and an interquartile range of 2–12. Thus, some candidate taxa were documented by only a few references, whereas others were supported by extensive bibliographic coverage.

Ecological information was available for most, but not all, candidate taxa. Macrohabitat descriptions (*macrohabitatRaw*) were present for 264 species and substrate descriptions (*substrateRaw*) for 314 species, leaving 44 species without any recorded macrohabitat or substrate information. Among species with macrohabitat data, the number of distinct macrohabitat descriptors per species ranged from 1 to 71 (median 2), while the number of distinct substrate descriptors among species with substrate data ranged from 1 to 81 (median 3). Across all candidate taxa, 679 unique macrohabitat descriptors and 1,355 unique substrate descriptors were represented, encompassing a wide variety of vegetation types, altitudinal zones and both living and dead plant substrates. The integrated ecology table therefore documented, for each candidate taxon, its regional distribution (*countriesPresent*), aggregated bibliography (*associatedReferencesAll*) and concatenated macrohabitat and substrate descriptors (*macrohabitatRaw*, *substrateRaw*).

### Species distribution modelling and model performance

Of the 365 candidate species, 101 with at least five unique occurrence locations were retained for species distribution modelling, and the remaining 264 species lacked sufficient or usable records and were treated as unmodelled candidates. The modelled subset comprised 101 candidate Eumycetozoa species spanning six orders, numerically dominated by Physarales and Stemonitidales, with additional contributions from Cribrariales, Trichiales and several less speciose orders. Among these 101 modelled candidate species, the number of unique occurrence locations per species ranged from 5 to 66, with a median of 9; 50 species were represented by 5–9 occurrences, 36 by 10–19 and only 15 by 20 or more.

Within the modelled subset, representation was distributed across six orders, with Physarales contributing 37 species and Stemonitidales 22. Cribrariales and Trichiales accounted for 18 and 17 species, respectively, while Echinosteliales contributed seven species and Liceales a single species.

**Figure 1:**
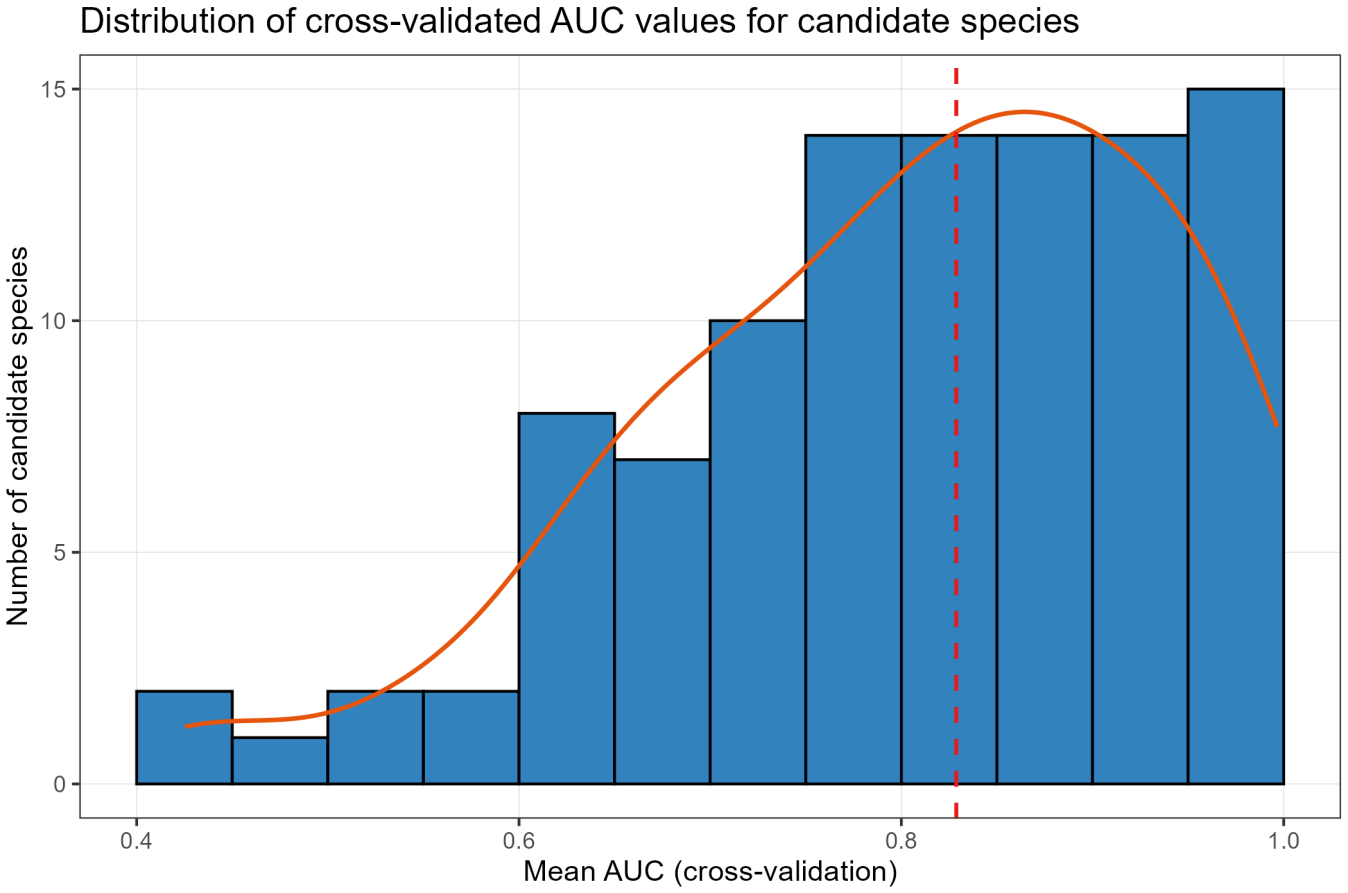
Distribution of cross-validated mean AUC values for MaxEnt-type models of candidate Eumycetozoa species. Bars show how many species fell within each AUC interval, illustrating the range and median discrimination across the candidate pool.

**Figure 2:**
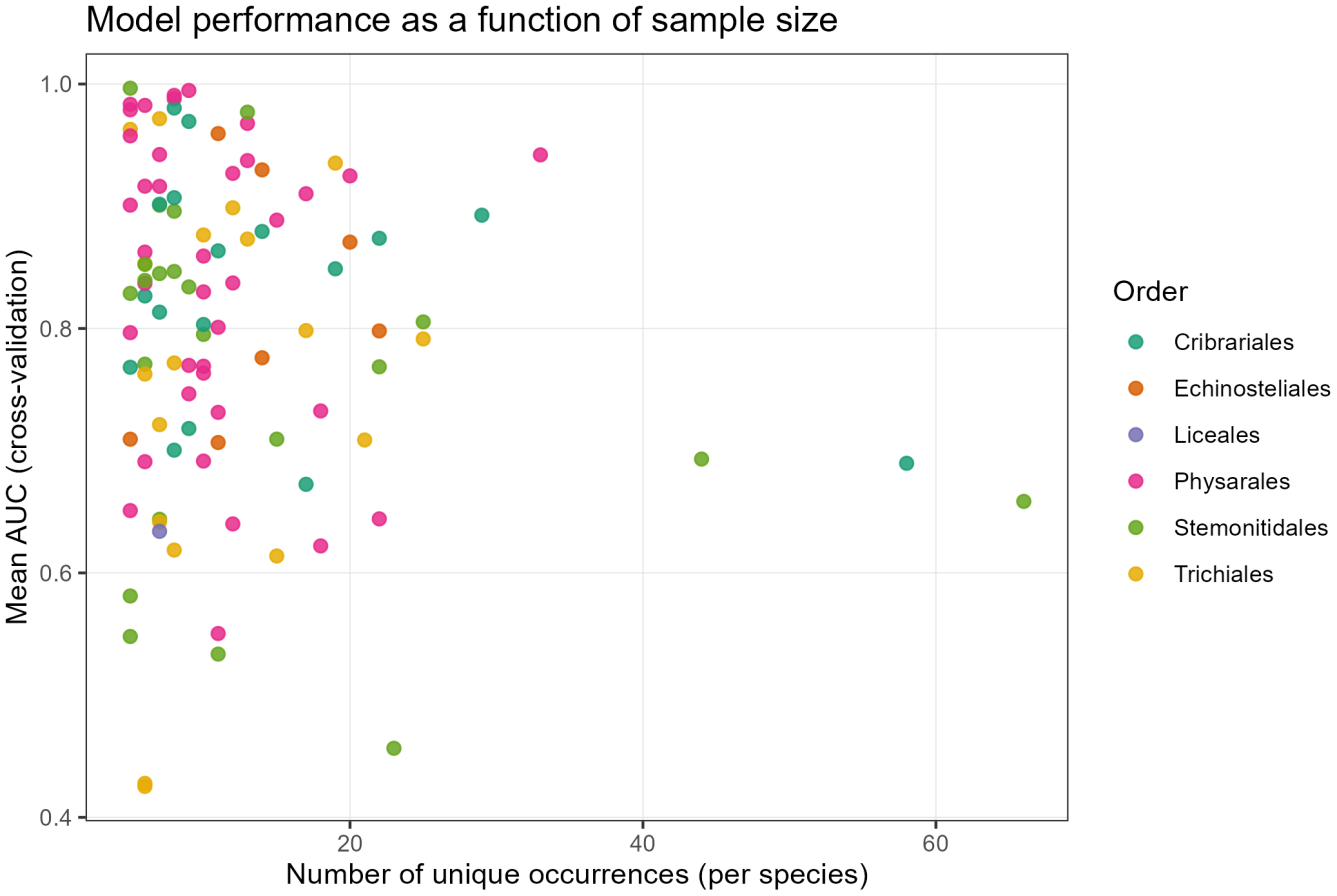
Sample size *versus* model discrimination in candidate Eumycetozoa. Points show species by unique occurrences after thinning and mean cross-validated AUC, illustrating the variable relationship between sample size and model performance.

**Figure 3:**
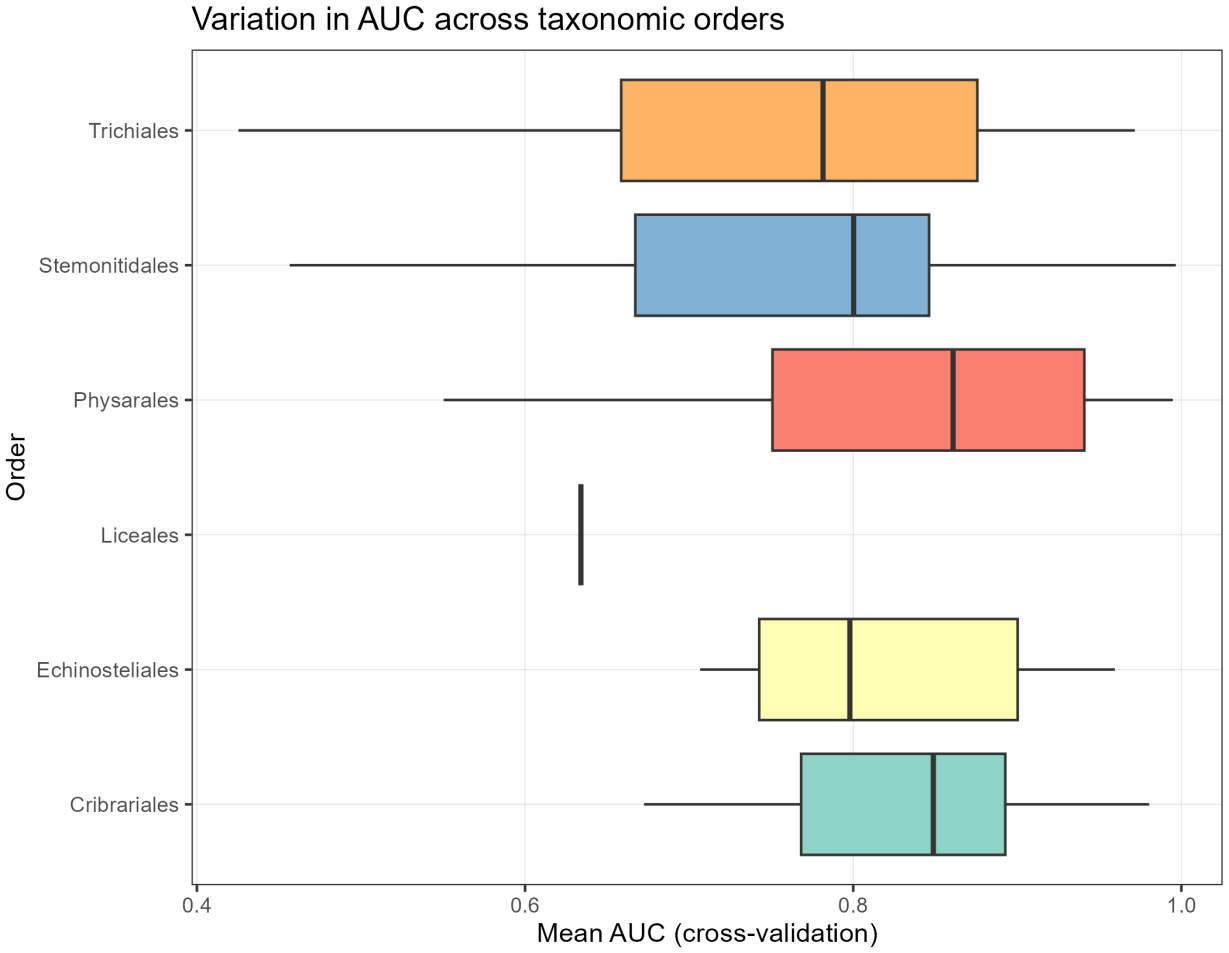
Variation in cross-validated mean AUC among Eumycetozoa orders. Boxplots show AUC distributions by order, highlighting differences among major lineages and the dominance of Physarales and Stemonitidales in the modelled set.

Species distribution models were successfully fitted and evaluated for these 101 species (Supplemental Information, Section 1.3; Pawłowicz, 2026). Across the 101 modelled species, cross-validated mean AUC values ranged from 0.43 to 1.00 with a median of 0.83, whereas Boyce statistics were unavailable because all models returned NA for this metric. The distribution of sample sizes across modelled species and the spread of mean AUC values are shown in Fig. 1, while the relationship between sample size and model discrimination was depicted by a scatterplot on Fig. 2. Variation in AUC among orders was summarised by boxplots of AUC by order on Fig. 3.

### Predicted suitability in Poland and regional importance of voivodeships

**Figure 4:**
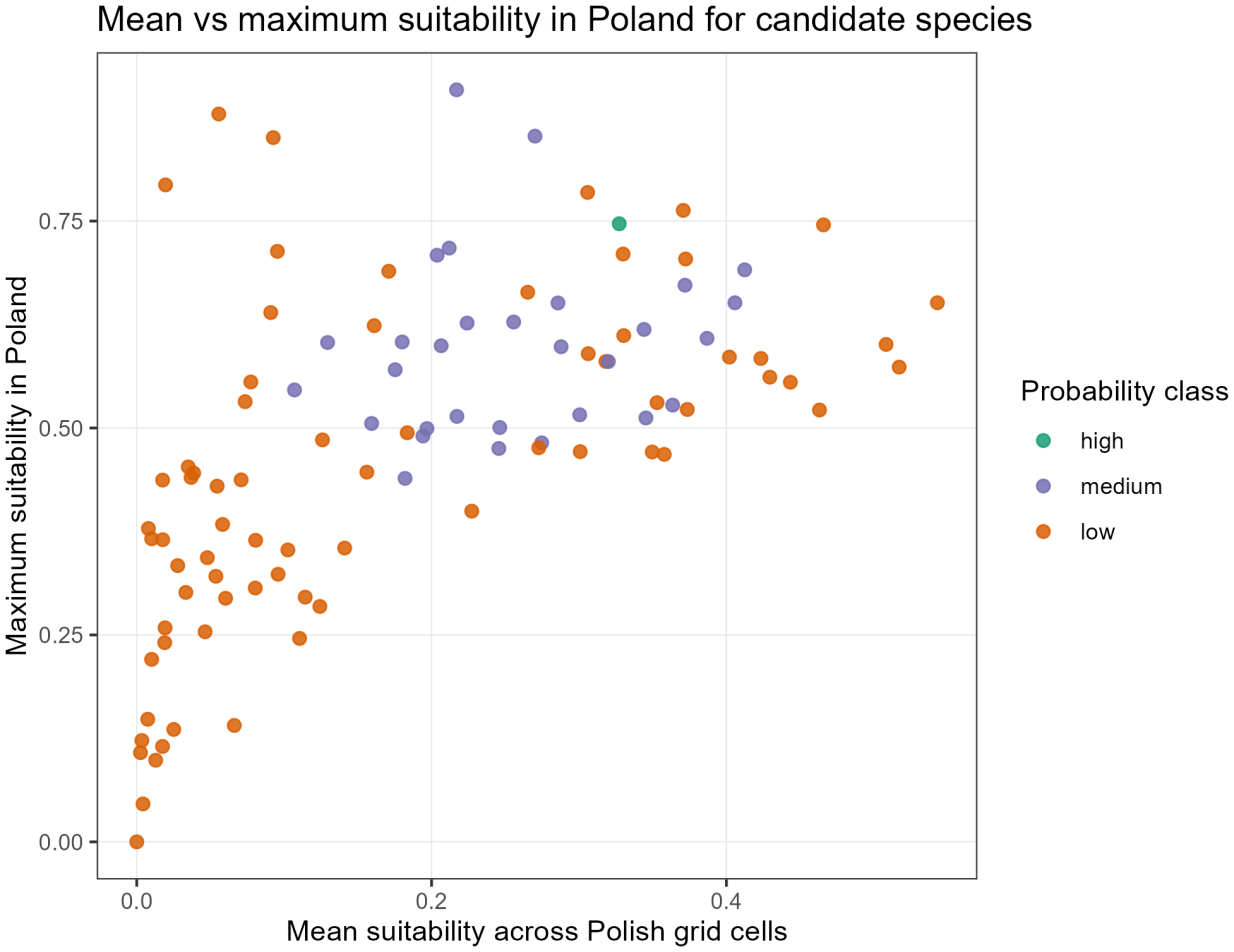
Maximum and mean predicted climatic suitability in Poland for modelled candidate Eumycetozoa species. Points show species by maximum logistic suitability and mean suitability across all Polish grid cells.

Suitability summaries in Poland were obtained for the 101 candidate species with fitted SDMs, each characterised by maximum suitability, mean suitability and the proportion of Polish grid cells predicted suitable. Maximum suitability values ranged from near zero to approximately 0.91 and mean suitability from near zero to approximately 0.54, indicating that some species were predicted to encounter highly suitable conditions within at least part of Poland, whereas others had uniformly low suitability. With respect to area, 73 of the 101 modelled species had at least some grid cells above the 10th-percentile suitability threshold within Poland, whereas 28 had no predicted suitable cells; 10 species were predicted to have climatically suitable conditions in at least half of the Polish grid cells. The joint distributions of maximum and mean suitability, and of maximum suitability against the proportion of suitable area are visualised in scatterplots that show how these indicators covaried across species (Fig. 4 and Fig. S1).

**Figure 5:**
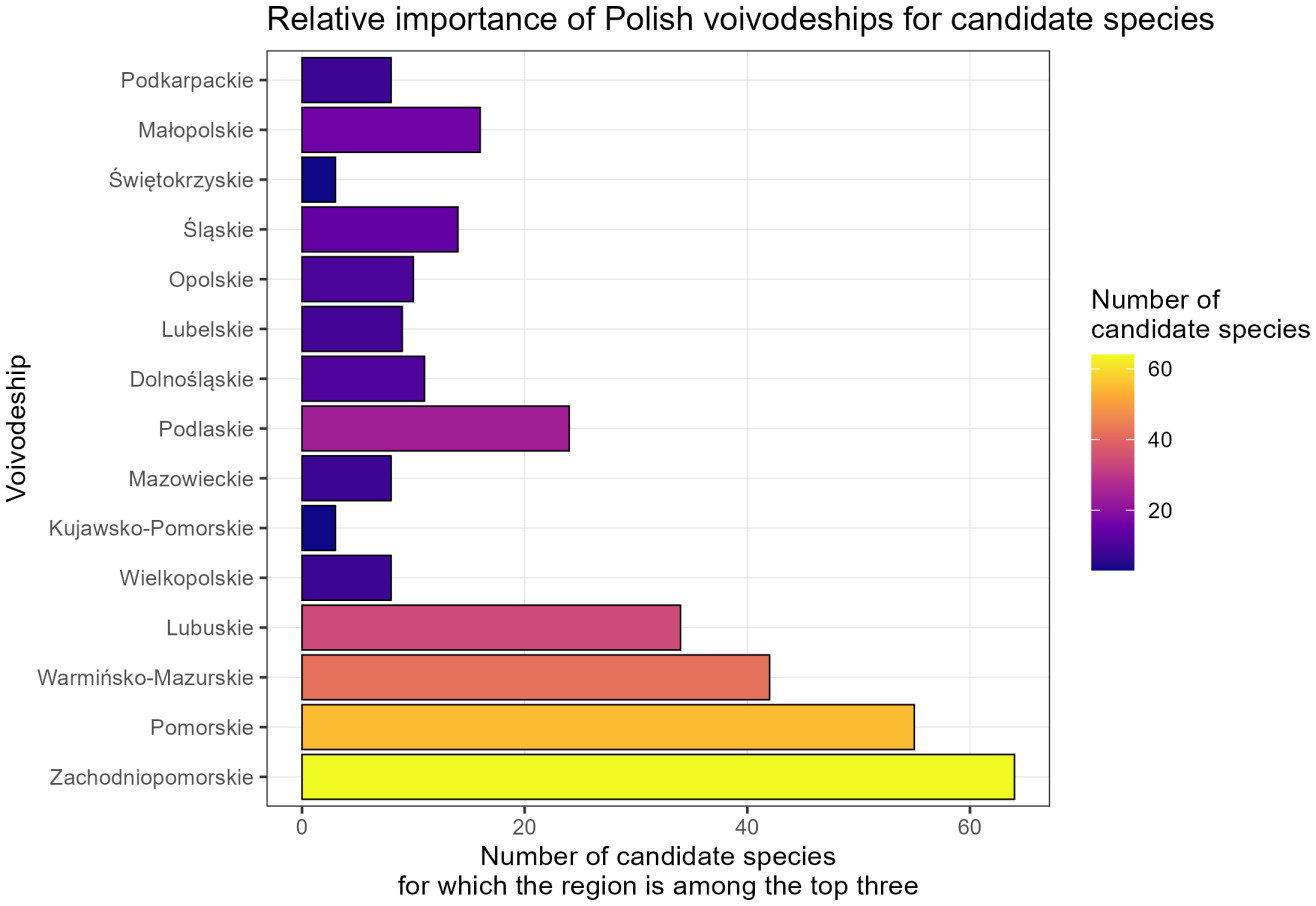
Voivodeship importance for Eumycetozoa. Bars show how many species rank each voivodeship in the top 3 by suitable area at the 10th-percentile threshold, highlighting Zachodniopomorskie, Pomorskie and Warmińsko-Mazurskie.

Region-level summaries showed that, for each modelled candidate species, up to three Polish voivodeships with the largest proportion of area classified as suitable were identified and then the number of species highlighting each voivodeship among their top three regions was counted without weighting (Supplemental Information, Section 1.4; Pawłowicz, 2026). After harmonisation of region names, Zachodniopomorskie emerged as the most frequently selected voivodeship, appearing among the top three predicted regions for 64 species, followed by Pomorskie (55 species) and Warmińsko-Mazurskie (42 species). Lubuskie and Podlaskie were also frequently selected among the top three regions for multiple candidate species. Several voivodeships, such as Kujawsko-Pomorskie and Świętokrzyskie, were seldom selected and Łódzkie did not occur among the top predicted regions. These tallies were unweighted counts of modelled species and therefore provided indicative rather than formally weighted measures of regional importance. The frequency with which each voivodeship was selected as a top predicted region across candidate species was displayed as a horizontal bar plot (Fig. 5). Because both the candidate hotspot surface and the voivodeship tallies were derived from SDM projections on the 10-arc-minute WorldClim grid, comparisons with the observed 10 × 10 km richness summaries were restricted to the voivodeship scale rather than to individual grid cells.

**Figure 6:**
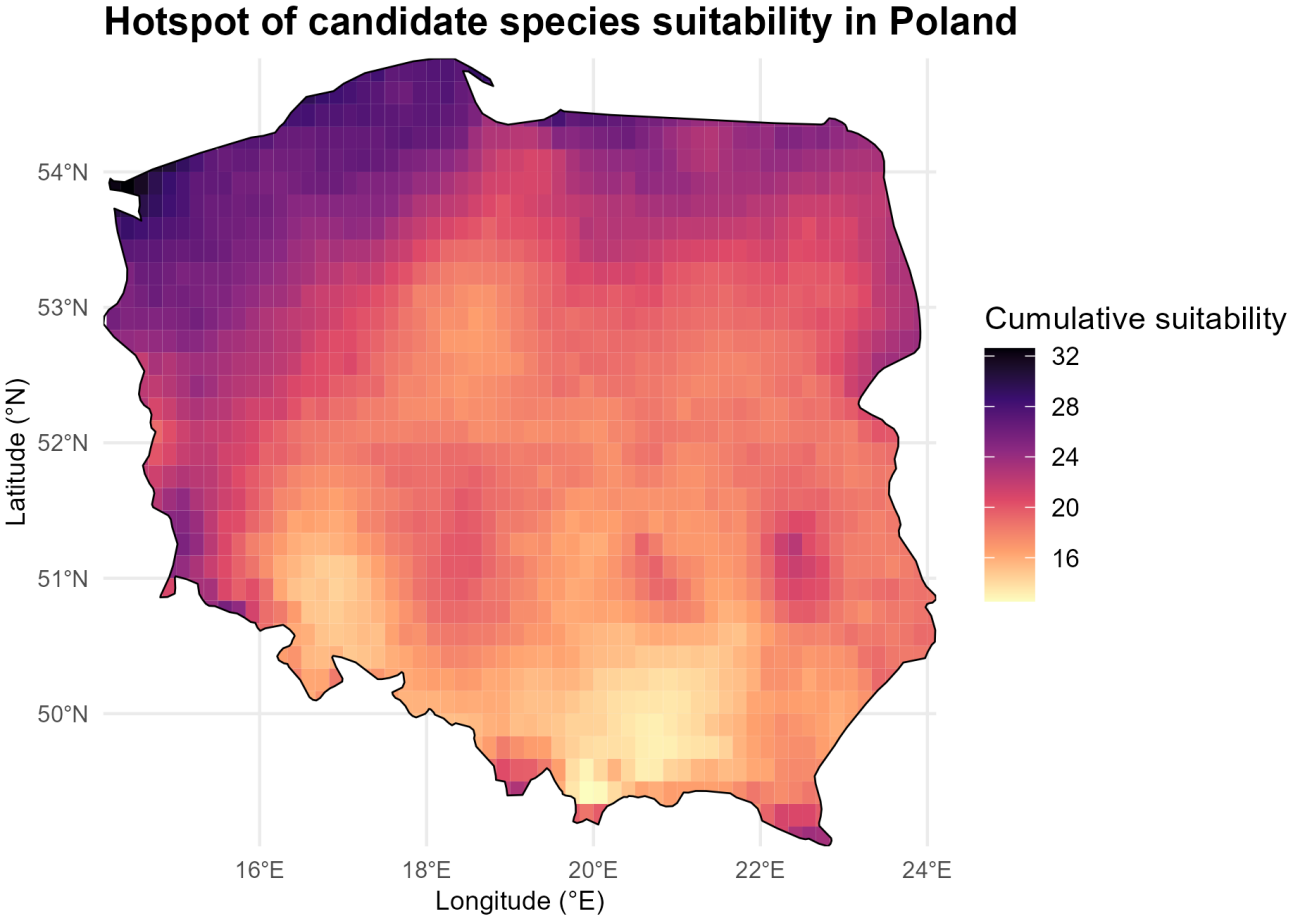
Hotspot map of cumulative climatic suitability for Eumycetozoa species in Poland. Each 10-arc-minute cell sums suitability across species; higher values mark multi-species hotspots, especially in western/northern Poland.

Summing continuous suitability across all modelled species produced a cumulative suitability surface that represented a hotspot map for candidate taxa within Poland (Fig. 6).

### Climatic suitability classes, synthesis by order and predictive maps

Combining model performance with Polish suitability indicators yielded a categorical climatic suitability assessment for the modelled candidate species. Suitability classes (high, medium, low) were defined only for the 101 modelled candidate species. Among these, 30 were classified as medium and one as high climatic suitability for occurrence in Poland, so 31 species had medium or high modelled climatic suitability; the remaining modelled species fell into the low-suitability class. Adding these 31 taxa to the 278 species already recorded from Poland yields a conservative climate-screened estimate of 309 species likely to occur nationally under the present modelling framework. The 264 candidate species without SDMs were retained in the regional species pool without SDM-based suitability assignments and were labelled as not modelled.

The sole high-suitability candidate, *Badhamia melanospora*, exhibited relatively strong model discrimination (aucMean = 0.83) together with high maximum suitability, moderate mean suitability and a substantial proportion of suitable area within Poland (Supplemental Information, Section 1.2; Pawłowicz, 2026). The consolidated candidate species table integrated order, genus, species and full scientific names with known country-level distributions, macrohabitat and substrate descriptors, bibliographic references, numbers of occurrences used for modelling, AUC summaries, Polish suitability metrics, suitability groups and top regions, providing a summary for all 365 candidate taxa. Synthesis by order based on the Polish checklist and the candidate list showed substantial increases in potential richness when candidate species were added to the currently observed Polish myxobiota (Table S2). These increases were concentrated mainly in Physarales, Stemonitidales, Trichiales and Cribrariales. Of the 11 orders represented in the regional pool, six are currently recorded from Poland, whereas five (Acytosteliales, Cavosteliales, Dictyosteliales, Liceales and Protosteliales) are represented only by candidate taxa. Because Table S2 includes all 365 candidate taxa irrespective of modelling status, its combined values should be interpreted as theoretical maxima derived from the regional pool rather than as SDM-based estimates of Polish richness. These patterns are visualised on Figs. S2, S3.

Predicted climatic suitability maps for the 101 candidate species whose SDMs met data sufficiency and quality-control criteria were provided as Supplementary Figures (Supplemental Information, Section 1.5; Pawłowicz, 2026). These MaxEnt-type projections depicted the potential distribution of environmentally suitable conditions for each mapped species within Poland and complemented the aggregated regional and by-order summaries described above.

## Discussion

### Reproducible analytical framework and initial data screening

The analytical workflow was implemented in R with explicit logging of software versions and key dataset characteristics, so that all steps from data harmonisation to mapping can be repeated on the published Darwin Core resource. Early inspection of core taxonomic, temporal and geographic fields after data import provided a first assessment of data suitability for analyses of Eumycetozoa in Poland, allowing the detection of obvious anomalies or gaps before checklist construction and modelling.

The harmonised occurrence dataset and associated taxonomic products provide a consistent basis for assessing Eumycetozoa diversity in Poland and placing it in a Central and Eastern European context. A dedicated Polish subset separates national records from the broader regional pool, avoiding conflation of patterns across countries. The usefulness of any output derived from Darwin Core data nevertheless depends on two linked conditions: sufficiently consistent core occurrence records and sufficiently reliable source identifications. In the present study, nomenclature, lineage placement and core occurrence fields were standardised, whereas heterogeneous habitat and substrate descriptors were retained only as contextual annotations and were not used in the SDMs. For the checklist totals, candidate pool and climatic projections to be informative at the national scale, the analysis assumes that most retained records represent real occurrences of the reported taxon under the accepted species concept and are georeferenced accurately enough for assignment to the modelling grid. However, standardisation cannot retrospectively verify every original identification. Where vouchers are available, questionable records should ideally be reassessed; as illustrated by Bates et al. (2025), a processed rarity list may retain an implausible taxon until the underlying specimen is reviewed and the misidentification corrected. The Polish outputs should therefore be read as broad-scale, data-dependent hypotheses for prioritising future survey effort rather than as definitive statements for every individual taxon. Spatially, the Polish subset shows pronounced clustering of records, with many 10 × 10 km grid cells lacking any documented occurrences, indicating substantial gaps in sampling that are important when interpreting richness and model predictions.

The national checklist shows a strongly uneven taxonomic composition. Physarales is the most species-rich order with 112 species, followed by Trichiales (56), Stemonitidales (55) and Cribrariales (50), whereas Echinosteliales and Ceratiomyxales are represented by only three and two species, respectively, giving a total of 278 species in six orders. These totals reflect the currently accepted names and species concepts applied in the harmonised checklist. This skew implies that biodiversity metrics and any bioindication schemes based on the current checklist will be dominated by a few speciose orders, while orders with very few recorded species will contribute little to statistical signals and will be particularly sensitive to incomplete sampling.

### Candidate species pool and ecological breadth

Comparison of the Polish checklist with the wider Central and Eastern European dataset identifies 365 species present regionally but absent from the current Polish list (Supplemental Information, Section 1.2 Pawłowicz, 2026). Most of these candidates are already documented from countries bordering Poland, which strengthens their biogeographic plausibility, whereas the smaller subset known only from more distant parts of the region remains more speculative. The candidate pool spans 11 orders and many genera, indicating that potential additions to the Polish myxobiota are taxonomically widespread rather than confined to peripheral lineages. Their occurrence in more than one country further supports this regional plausibility.

Aggregated bibliographic information shows marked heterogeneity in documentation, from species cited in a single publication to others supported by extensive literature. Well-documented candidates may be suitable for early inclusion in biodiversity assessments and exploratory bioindication schemes, whereas poorly documented taxa may require additional taxonomic and ecological study before they can be used with confidence. The ecological fields in Supplemental Information, Section 1.2 (Pawłowicz, 2026), which combine raw macrohabitat and substrate descriptors from across Central and Eastern Europe, highlight the wide environmental spectrum occupied by candidate species. The text-based descriptors encompass contrasting vegetation types, elevation bands and a broad array of living and dead plant substrates. Because these descriptors were aggregated from heterogeneous source fields and publications, the integrated table is best treated as descriptive ecological context rather than as a fully standardised comparative substrate or habitat classification. It is useful for screening broad ecological associations and for prioritising field verification, but it was not used to drive the SDM predictions. The candidate pool therefore combines a large set of species with clear biogeographic proximity to Poland and a smaller group known only from more distant parts of Central and Eastern Europe, for which potential occurrence in Poland is more speculative.

### Species distribution modelling strategy

The occurrence summary for candidate species shows that most taxa are known from very few unique locations. Of the 365 candidate species, only 101 had at least five unique occurrence locations and could be retained for species distribution modelling, whereas the remaining 264 lacked sufficient or usable records and were treated as unmodelled candidates. The modelling set therefore emphasised relatively better-sampled species while remaining taxonomically broad, although some lineages lacked species that met the threshold. As a result, predictions from the species distribution models mainly reflect the environmental niches of more frequently recorded taxa, whereas rare species remain under-represented in SDM-derived richness and bioindication analyses. This design balanced the need to include a meaningful fraction of the candidate pool against the well-known tendency of SDMs to perform poorly at very small sample sizes.

Model calibration used a reduced, low-collinearity environmental predictor stack and a target-group background dataset. The modelling extent was defined around the pooled candidate occurrences, and background points were sampled from the spatial distribution of all Eumycetozoa records to anchor calibration in the environmental space actually sampled for the group. Systematic removal of highly correlated predictors reduced redundancy among environmental variables and supports more stable estimation of species–environment relationships for the taxa that could be modelled.

All SDMs were fitted and projected on WorldClim 2.1 bioclimatic variables at 10-arc-minute resolution, so the resulting maps described coarse-grained climatic potential rather than microhabitat conditions within individual forest stands. This choice is ecologically justified at the regional scale because temperature and precipitation regimes affect substrate moisture balance, the duration and recurrence of favourable periods for trophic stages and sporocarp formation, and the broad distribution of forest environments in which suitable substrates occur (Stephenson & Rojas, 2017; Leontyev & Schnittler, 2017; Stephenson, 2023). Fine-scale heterogeneity in moisture-buffered substrates was not explicitly represented. Although the occurrence data included descriptors of forest type, habitat, management and substrates, these variables were heterogeneous across countries and among source records and were not available as consistent gridded covariates for the entire study region, so the SDMs relied on climatic predictors alone. Thus, variability in record-level habitat or substrate wording could influence the descriptive ecological summaries, but it did not directly influence SDM calibration, projection or suitability classification. Accordingly, the models estimate climatic permissiveness rather than complete habitat suitability.

### Model performance and evaluation

MaxEnt-type models could be calibrated for 101 candidate species, and the resulting distribution of *AUC* values indicates that the climatic predictors captured meaningful distributional patterns for many, though not all, taxa (Supplemental Information, Section 1.3; Pawłowicz, 2026). Across the 101 modelled species, cross-validated mean *AUC* values ranged from 0.43 to 1.00 with a median of 0.83, whereas Boyce statistics were unavailable because all models returned *NA*. Some species exhibited markedly lower *AUC* values, demonstrating that model performance was uneven and that contributions from poorly discriminating models to aggregated biodiversity or indicator metrics should be treated cautiously. Because more than half of the modelled species were represented by fewer than 10 unique occurrences, their fitted niches and projected distributions should be regarded as exploratory, even when *AUC* values were relatively high.

Model evaluation in this framework therefore relied solely on *AUC*, limiting the capacity to assess how well predicted suitability aligns with the empirical distribution of presences beyond discrimination. The distribution of *AUC* scores and its relationship with sample size indicate that performance tends to improve with increasing occurrence number but remains variable across orders. Overall, the climatic predictors captured non-trivial structure in the distributions of many species, but the combination of small sample sizes, spatial clustering and the absence of spatially structured evaluation argues for cautious interpretation of individual model outputs.

### Predicted climatic suitability and implications for occurrence in Poland

Species-level suitability metrics for Poland reveal a wide gradient in how strongly candidate Eumycetozoa are predicted to be capable of occupying national environments (Supplemental Information, Section 1.2 Pawłowicz, 2026). These values indicate climatic similarity to known regional occurrences, not confirmed local occupancy or the presence of the required substrates. The relationship between maximum and mean suitability values for Poland and the distribution of suitable area per species summarise these gradients. To translate continuous outputs into a decision-oriented framework, species were assigned to climatic suitability groups based on a combination of AUC, maximum and mean suitability within Poland and the proportion of the country classified as suitable (Supplemental Information, Section 1.2 Pawłowicz, 2026). This grouping was intended as a conservative prioritisation tool rather than direct evidence of occurrence. Among the 101 modelled species, 31 reached medium or high climatic suitability, whereas most remained low and 264 candidates were not modelled. These taxa therefore represent the most robust priorities for field confirmation and for initial bioindication-oriented testing. The spatial patterns illustrated by the 101 suitability maps in Supplemental Information (Section 1.5), (Pawłowicz, 2026) highlight a concentration of environmentally suitable conditions for multiple candidate species in western and northern Poland, particularly in Zachodniopomorskie, Pomorskie, Warmińsko-Mazurskie, Lubuskie and Podlaskie. Because these maps were generated on the 10-arc-minute climatic grid, their main value is to prioritise survey regions rather than to infer fine-scale distributions within individual forest stands.

### Regional and taxonomic patterns of potential diversity and implications for bioindication

Aggregating model outputs at the regional scale clarifies how potential Eumycetozoa diversity is partitioned among Polish voivodeships. Western and northern voivodeships emerge repeatedly as climate-priority areas for candidate species, whereas some central regions are selected much less often. These tallies are useful for survey design but should not be treated as weighted measures of regional importance, because they do not adjust for voivodeship area or variation in model performance. Because the hotspot surface was computed on the 10-arc-minute WorldClim grid, whereas observed richness was summarised on a 10 × 10 km equal-area grid, comparisons between observed and predicted patterns remain interpretable only at the voivodeship scale.

On the taxonomic axis, combining observed and candidate richness per order clarifies how much additional diversity is expected within each of the 11 targeted orders beyond the current checklist. Candidate additions are concentrated mainly in Physarales, Stemonitidales and Trichiales, while five orders represented in the regional pool remain unrecorded in Poland and are currently supported only as candidate lineages. These latter orders should therefore be treated as hypotheses for future verification rather than as evidence of confirmed national occurrence. Within the broader regional pool, the 31 candidate species with medium or high modelled climatic suitability provide the most tractable targets for validation and for initial exploration of the indicator potential of Eumycetozoa in Polish forests. For biodiversity assessment and bioindication, these spatial and taxonomic syntheses have several implications. The dominance of a few speciose orders in both observed and potential richness implies that composite biodiversity metrics and indicator assemblages derived from the present work will be heavily influenced by these lineages, whereas orders with few species—recorded or predicted—will contribute little to statistical signals and remain vulnerable to sampling incompleteness. The regional concentration of candidate taxa in western and northern voivodeships suggests clear priorities for field validation: areas repeatedly highlighted by multiple species, especially in Zachodniopomorskie, Pomorskie, Warmińsko-Mazurskie, Lubuskie and Podlaskie, represent promising targets for surveys aimed at confirming currently undocumented taxa and testing the feasibility of slime-mould-based bioindication. In practice, these should be treated as climate-priority regions within which survey effort is further directed to suitable forest stands, substrate types and sampling periods.

## Conclusions

This study showed that the Polish myxobiota is richer and more taxonomically structured than previously documented, with a national checklist that integrates taxonomic identities, ecological descriptors and literature sources into a harmonised framework. The checklist reveals a strong dominance of a few speciose orders and substantial gaps in spatial coverage, highlighting both the value of existing data and the need for targeted surveys in undersampled areas.

Comparison with a regional Central and Eastern European dataset identified a large and taxonomically diverse pool of candidate species not yet recorded from Poland. Most candidates are already known from countries bordering Poland, and their collated habitat and substrate descriptors indicate that suitable environments are likely to exist within Polish forests. A substantial fraction of these candidates could be modelled using climatic predictors, providing exploratory projections of potential suitability and a first assessment of which taxa are most likely to occur in Poland. At this scale, the bioclimatic variables are best understood as describing the regional climatic envelope within which suitable forest conditions and moist substrates are more likely, rather than as direct predictors of local occurrence. Species distribution models suggested that suitable conditions for many candidate species are concentrated in western and northern Poland. In particular, Zachodniopomorskie, Pomorskie, Warmińsko-Mazurskie, Lubuskie and Podlaskie repeatedly emerged as priority regions when suitability was summarised across species. These coarse-grained patterns should be interpreted with caution because the models relied on presence-only data, relatively small sample sizes for many species and a climatic grid that does not capture microhabitat heterogeneity. They should therefore be used to prioritise field surveys and to test SDM-based hypotheses about the Polish myxobiota, rather than to infer local occurrence directly. Field validation within the highlighted regions must still evaluate forest type, substrate availability and survey-time weather.

The combination of a harmonised Polish checklist, a regionally defined candidate pool and exploratory SDM projections underscores that many Eumycetozoa species likely remain undocumented in Poland. It also shows that slime moulds have potential as components of forest biodiversity assessment and bioindication, particularly when interpreted in conjunction with other taxonomic groups and structural attributes of forests. Realising this potential will require coordinated taxonomic work, intensified surveys in climatically favourable regions and targeted studies that validate SDM predictions and assess how slime mould assemblages respond to forest management and environmental change.

## Supplemental Information

10.7717/peerj.21492/supp-1 Supplemental Information 1 Polish checklist of Eumycetozoa listing accepted names and Polish references Records under historical synonyms were reconciled; the checklist with order, genus, species, substrate and habitat is in Supplementary Section 1.1.

10.7717/peerj.21492/supp-2 Supplemental Information 2 Order-level richness of recorded and candidate Eumycetozoa associated with Poland For each of 11 orders in the regional pool, the table shows recorded species, candidates and their theoretical maximum total.

10.7717/peerj.21492/supp-3 Supplemental Information 3 Proportion of Poland climatically suitable for modelled candidate Eumycetozoa species For each species, the share of 10-arc-minute cells above the 10th-percentile threshold is shown, from small to large suitable areas.

10.7717/peerj.21492/supp-4 Supplemental Information 4 Order-level richness of Eumycetozoa associated with Poland Bars show, for each of 11 orders in the regional pool, recorded species, additional candidates and their sum as the theoretical maximum per order.

10.7717/peerj.21492/supp-5 Supplemental Information 5 SDM-based climatic suitability classes among Eumycetozoa orders The heatmap shows, for each order, candidate species numbers in high, medium, low and not-modelled groups, highlighting Physarales and Stemonitidales.

10.7717/peerj.21492/supp-6 Supplemental Information 6 Replication Code in .R format

10.7717/peerj.21492/supp-7 Supplemental Information 7 Codebook for Supplemental Files

10.7717/peerj.21492/supp-8 Supplemental Information 8 Polish Species; level checklist Species-level checklist of Eumycetozoa recorded from Poland. For each taxon (order, genus, species, scientificName), the table includes semicolon; separated habitat and substrate summaries and a collated list of Polish references. It represents the full, annotated national checklist underlying the shortened version in the main text.

10.7717/peerj.21492/supp-9 Supplemental Information 9 Candidate species table List of 365 candidate species that occur in Central and Eastern Europe but are not yet recorded from Poland. For each taxon, the table provides taxonomic placement, countries of occurrence, aggregated habitat and substrate descriptors, collated bibliography and, where available, species distribution model (SDM) metrics, Polish suitability indicators and probability class, together with top Polish regions.

10.7717/peerj.21492/supp-10 Supplemental Information 10 Model performance metrics Evaluation results for MaxEnt-type SDMs fitted to candidate species with sufficient data. For each modelled taxon, the table reports sample size, number of cross validation folds and AUC statistics, together with attempted Boyce indices. It allows users to assess the strength and uncertainty of individual models.

10.7717/peerj.21492/supp-11 Supplemental Information 11 Top Polish regions per species Region-level summary indicating, for each modelled candidate species, up to three Polish voivodeships with the largest proportion of grid cells classified as climatically suitable. Voivodeships are listed by their Polish names and can be used to prioritise areas for targeted surveys and validation.

10.7717/peerj.21492/supp-12 Supplemental Information 12 Map collection Set of 101 raster maps showing predicted potential distributions in Poland for modelled candidate species. Each figure displays continuous climatic suitability and the subset of grid cells exceeding a 10th-percentile training presence threshold.

## Additional Information and Declarations

### Data Availability

The following information was supplied regarding data availability: The code is available in the Supplemental Files. The Global Biodiversity Information Facility data is available at: Pawłowicz, T. (2025) Biodiversity Data Journal https://doi.org/10.15468/jqukxm. The data is available at: Pawłowicz, Tomasz (2026), “Recorded and predicted occurrence of Eumycetozoa in Poland from Central and Eastern European data”, Mendeley Data, V1, doi: 10.17632/b8ych9v3km.1 https://doi.org/10.17632/b8ych9v3km.2.
