# Supplementary material for: Recorded and predicted occurrence of slime moulds (Eumycetozoa) in Poland from Central and Eastern European data
Source: PeerJ. 2026 Jul 9;14:e21492. doi: 10.7717/peerj.21492 (PMC13356830; doi:10.7717/peerj.21492)
Supplement: Supplemental Information 1 — Records under historical synonyms were reconciled; the checklist with order, genus, species, substrate and habitat is in Supplementary Section 1.1. [file peerj-14-21492-s001.docx]

| Species | Polish bibliographic sources |
| --- | --- |
| *Amaurochaete atra* (Alb. & Schwein.) Rostaf., 1874 | Krupa, 1889; Błoński, 1890; Namysłowski, 1910; Jarocki, 1924; Krzemieniewska, 1933; Krzemieniewska, 1960b; Kalinowska-Kucharska, 1975; Drozdowicz, 1992; Drozdowicz, 1997; Drozdowicz, 1997a; Stojanowska and Panek, 2004; Panek and Romański, 2010; Ławrynowicz et al., 2011 |
| *Amaurochaete tubulina* (Alb. & Schwein.) T. Macbr., 1922 | Kalinowska-Kucharska, 1975; Krzemieniewska, 1933; Krzemieniewska, 1957; Krzemieniewska, 1960b; Stojanowska, 1972; Stojanowska, 1977; Stojanowska 1983; Stojanowska, 1983; Stojanowska, 1984; Drozdowicz, 1992; Panek and Romański, 2010; Ławrynowicz et al., 2011; Stojanowska, 2004a; Stojanowska, 2004b; Paul et al., 2023 |
| *Angioridium sinuosum* (Bull.) Grev., 1827 | Kalinowska-Kucharska, 1975; Jarocki, 1924; Krzemieniewska, 1933; Krzemieniewska, 1957; Krzemieniewska, 1960b; Krzemieniewska and Badura, 1954; Drozdowicz, 1992; Bujakiewicz and Fiebich, 1992; Bujakiewicz, 1999; Stojanowska, 1977b; Stojanowska, 1983; Stojanowska, 1984; Stojanowska and Panek, 2002; Ławrynowicz et al., 2011; Panek and Romański, 2010; Stojanowska, 2004b; Stojanowska and Panek, 2004; Stojanowska and Panek, 2005; Ślusarczyk, 2021; Pawłowicz et al., 2025; Stojanowska, 1977; Stojanowska 1983 |
| *Arcyria affinis* Rostaf., 1875 | Kalinowska-Kucharska, 1975; Krzemieniewska, 1957; Krzemieniewska, 1960b; Stojanowska, 1972; Stojanowska, 1977; Stojanowska, 1980a; Stojanowska, 1980b; Wrońska, 1974; Stojanowska, 2000a; Drozdowicz et al., 2012; Ławrynowicz et al., 2011; Panek and Romański, 2010; Stojanowska, 2004; Stojanowska and Panek, 2004; Salamaga, 2021; Paul et al., 2023 |
| *Arcyria cinerea* (Bull.) Pers., 1801 | Krupa, 1887; Jarocki, 1924; Krawiec, 1965; Krzemieniewska, 1929; Krzemieniewska, 1933; Krzemieniewska, 1947; Krzemieniewska, 1957; Krzemieniewska and Badura, 1954; Kalinowska-Kucharska, 1975; Wrońska, 1974; Drozdowicz, 1992; Miśkiewicz, 2001; Drozdowicz, 2001; Stojanowska, 1970; Stojanowska, 1972; Stojanowska, 1977b; Stojanowska, 1980b; Stojanowska, 1983; Stojanowska, 1984; Stojanowska, 1992; Drozdowicz and Wilga, 2002; Stojanowska, 2000a; Stojanowska and Panek, 2002; Bochynek and Drozdowicz, 2011; Drozdowicz, 2005; Drozdowicz et al., 2007; Drozdowicz et al., 2012; Wilga and Ciechanowski, 2007; Ławrynowicz et al., 2011; Salamaga et al., 2016; Salamaga, 2021; Bochynek & Drozdowicz, 2012; Ślusarczyk, 2010; Ślusarczyk, 2021; Panek and Romański, 2010; Paul et al., 2023; Pawłowicz et al., 2025 |
| *Arcyria congesta* (Sommerf.) Berk. & Broome, 1876 | Jarocki, 1924; Krzemieniewska, 1933; Krzemieniewska, 1960b; Stojanowska, 1972; Stojanowska 1983; Pawłowicz et al., 2025 |
| *Arcyria denudata* (L.) Wettst., 1886 | Krupa, 1887; Błoński and Drymmer, 1889; Krupa, 1889; Steinecke, 1918; Jarocki, 1924; Krawiec, 1965; Krzemieniewska, 1933; Krzemieniewska, 1947; Krzemieniewska, 1957; Krzemieniewska and Badura, 1954; Kalinowska-Kucharska, 1975; Miśkiewicz, 2001; Drozdowicz, 1992; Stojanowska, 1970; Stojanowska, 1972; Stojanowska, 1977b; Stojanowska, 1980b; Stojanowska, 1981; Stojanowska, 1983; Stojanowska, 1984; Stojanowska, 1992; Drozdowicz, 2005; Drozdowicz and Wilga, 2002; Stojanowska, 2000a; Stojanowska and Panek, 2002; Drozdowicz et al., 2007; Drozdowicz et al., 2012; Wilga and Ciechanowski, 2007; Ławrynowicz et al., 2011; Bochynek and Drozdowicz, 2011; Bochynek & Drozdowicz, 2012; Panek and Romański, 2010; Chachuła et al., 2021; Salamaga, 2021; Ślusarczyk, 2010; Ślusarczyk, 2021; Paul et al., 2023; Pawłowicz et al., 2025 |
| *Arcyria helvetica* (Meyl.) H. Neubert, Nowotny & K. Baumann, 1989 | Stojanowska 1983; Stojanowska, 2004; Paul et al., 2023 |
| *Arcyria imperialis* (G. Lister) Q. Wang & Yu Li, 2006 | Panek and Romański, 2010 |
| *Arcyria incarnata* (Pers. ex J. F. Gmel.) Pers., 1796 | Błoński, 1888; Jarocki, 1924; Krawiec, 1965; Krupa, 1889; Krzemieniewska, 1933; Krzemieniewska, 1947; Krzemieniewska, 1957; Krzemieniewska, 1960b; Krzemieniewska and Badura, 1954; Kalinowska-Kucharska, 1975; Drozdowicz, 1992; Miśkiewicz, 2001; Stojanowska, 1970; Stojanowska, 1972; Stojanowska, 1977b; Stojanowska, 1980b; Stojanowska, 1983; Stojanowska, 1984; Stojanowska, 1992; Stojanowska, 2000a; Drozdowicz et al., 2007; Stojanowska and Panek, 2002; Jarocki, 1924; Wrońska, 1974; Panek and Romański, 2010; Ławrynowicz et al., 2011; Krzysztofiak et al., 2010; Bochynek and Drozdowicz, 2011; Salamaga et al., 2016; Salamaga, 2021 |
| *Arcyria major* (G. Lister) Ing, 1967 | Wrońska, 1974; Krzemieniewska, 1957; Krzemieniewska, 1960b; Stojanowska and Panek, 2002; Panek and Romański, 2010; Salamaga, 2021 |
| *Arcyria marginoundulata* Nann. -Bremek. & Y. Yamam., 1983 | Ronikier et al., 2013 |
| *Arcyria minuta* Buchet, 1927 | Tabacki, 1977; Drozdowicz et al., 2012; Panek and Romański, 2010; Stojanowska and Panek, 2004 |
| *Arcyria stipata* (Schwein.) Lister, 1894 | Krzemieniewska, 1957; Krzemieniewska, 1960b; Drozdowicz et al., 2012; Krzysztofiak et al., 2010; Stojanowska and Panek, 2002; Panek and Romański, 2010; Stojanowska, 2004; Salamaga, 2021 |
| *Arcyria virescens* G. Lister, 1921 | Bochynek, 2015 |
| *Badhamia affinis* Rostaf., 1874 | Kalinowska-Kucharska, 1975; Ławrynowicz et al., 2011 |
| *Badhamia albescens* (Ellis ex T. Macbr.) J.M. García-Martín, J.C. Zamora & Lado, 2023 | Ronikier et al., 2008; Drozdowicz, 1988, 1997b, 2001; Komorowska and Drozdowicz, 1996; Stojanowska, 2004a; Shchepin et al., 2022; Paul et al., 2023 |
| *Badhamia bethelii* (T. Macbr. ex G. Lister) J.M. García-Martín, J.C. Zamora & Lado, 2023 | Firich, 1962; Stojanowska 1983; Panek and Romański, 2010; Stojanowska and Panek, 2004 |
| *Badhamia capsulifera* (Bull.) Berk., 1852 | Rostafiński, 1874; Krzemieniewska, 1957; Krzemieniewska, 1960b; Kalinowska-Kucharska, 1975; Stojanowska, 1977b; Stojanowska, 1980a; Stojanowska, 1980b; Panek and Romański, 2010; Ławrynowicz et al., 2011 |
| *Badhamia foliicola* Lister, 1897 | Jarocki, 1924; Krzemieniewska, 1957; Krzemieniewska, 1960b; Stojanowska, 1980b; Stojanowska, 1980a; Salamaga, 2021 |
| *Badhamia lilacina* (Fr.) Rostaf., 1874 | Jarocki, 1924; Salamaga and Grzesiak, 2013 |
| *Badhamia macrocarpos* (Ces.) Rostaf., 1874 | Rostafiński, 1874; Krzemieniewska, 1957; Krzemieniewska, 1960b; Stojanowska 1983; Panek and Romański, 2010; Salamaga, 2021 |
| *Badhamia ovispora* Racib., 1884 | Raciborski, 1884a; Raciborski, 1884b; Szulczewski, 1951; Drozdowicz et al., 2003 |
| *Badhamia panicea* (Fr.) Rostaf., 1873 | Rostafiński, 1874; Krzemieniewska, 1957; Krzemieniewska, 1960b; Drozdowicz, 1992; Stojanowska, 1972; Stojanowska, 1977b; Stojanowska, 1980b; Stojanowska, 1983; Stojanowska, 1984; Stojanowska, 1992; Drozdowicz, 1997; Stojanowska and Panek, 2002; Stojanowska and Panek, 2004; Stojanowska, 2004; Stojanowska 1983; Panek and Romański, 2010; Bochynek and Drozdowicz, 2011; Salamaga, 2021 |
| *Badhamia populina* Lister & G. Lister, 1904 | Panek and Romański, 2010 |
| *Badhamia utricularis* (Bull.) Berk., 1852 | Rostafiński, 1874; Krzemieniewska, 1933; Krzemieniewska, 1957; Krzemieniewska, 1960b; Kalinowska-Kucharska, 1975; Stojanowska 1983; Stojanowska, 1992; Stojanowska, 2004; Stojanowska and Panek, 2004; Drozdowicz, 1992; Ławrynowicz et al., 2011; Panek and Romański, 2010; Ślusarczyk, 2010 |
| *Badhamia versicolor* Lister, 1901 | Pliszko and Bochynek, 2017; Krzemieniewska, 1960b |
| *Barbeyella minutissima* Meyl., 1914 | Jarocki, 1931; Krzemieniewska, 1947; Krzemieniewska, 1960b |
| *Brefeldia maxima* (Fr.) Rostaf., 1873 | Krzemieniewska, 1933; Stojanowska 1983; Krzemieniewska, 1960b; Stojanowska and Panek, 2002; Stojanowska and Panek, 2005 |
| *Calomyxa metallica* (Berk.) Nieuwl., 1916 | Krzemieniewska, 1933; Krzemieniewska, 1957; Krzemieniewska, 1960b; Stojanowska 1983; Stojanowska, 1984; Stojanowska, 2004; Paul et al., 2023 |
| *Ceratiomyxa fruticulosa* (O.F. Müll.) T. Macbr., 1899 | Błoński, 1890; Błoński and Drymmer, 1889; Bresadola, 1903; Michalski, 1951; Krzemieniewska, 1933; Krzemieniewska, 1947; Krzemieniewska, 1957; Krzemieniewska and Badura, 1954; Kalinowska-Kucharska, 1975; Drozdowicz, 1992; Miśkiewicz, 2001; Stojanowska, 1970; Stojanowska, 1972; Stojanowska, 1977b; Stojanowska, 1980b; Stojanowska, 1983; Stojanowska, 1984; Stojanowska, 2000a; Drozdowicz, 1997b; Drozdowicz, 2001; Drozdowicz and Wilga, 2002; Drozdowicz et al., 2007; Drozdowicz et al., 2012; Wilga and Ciechanowski, 2007; Krzysztofiak et al., 2010; Ławrynowicz et al., 2011; Bochynek and Drozdowicz, 2011; Bochynek & Drozdowicz, 2012; Panek and Romański, 2010; Salamaga et al., 2016; Salamaga, 2021; Ślusarczyk, 2010; Ślusarczyk, 2021; Paul et al., 2023; Pawłowicz et al., 2025 |
| *Ceratiomyxa porioides* (Alb. & Schwein.) J. Schröt., 1889 | Błoński and Drymmer, 1889; Kalinowska-Kucharska, 1975; Miśkiewicz, 2001; Drozdowicz et al., 2007; Drozdowicz et al., 2012; Wilga and Ciechanowski, 2007; Ławrynowicz et al., 2011; Panek and Romański, 2010; Chachuła et al., 2021; Salamaga, 2021; Stojanowska, 2004a; Stojanowska and Panek, 2004 |
| *Clastoderma debaryanum* A. Blytt, 1880 | Krzemieniewska, 1947; Krzemieniewska, 1957; Krzemieniewska, 1960b; Ławrynowicz et al., 2011; Paul et al., 2023 |
| *Claustria didermoides* (Pers.) Fr., 1849 | Stojanowska 1983 |
| *Collaria arcyrionema* (Rostaf.) Nann. -Bremek. ex Lado, 1991 | Rostafiński, 1874; Krzemieniewska, 1947; Krzemieniewska, 1957; Krzemieniewska, 1960b; Krzemieniewska and Badura, 1954; Michalski, 1951; Jarocki, 1924; Jarocki, 1931; Stojanowska, 1972; Stojanowska, 1977b; Stojanowska, 1983; Drozdowicz, 1992; Drozdowicz et al., 2007; Drozdowicz et al., 2012; Ławrynowicz et al., 2011; Panek and Romański, 2010; Salamaga et al., 2016; Salamaga, 2021; Stojanowska, 2004a; Stojanowska, 2004b; Stojanowska and Panek, 2004; Ślusarczyk, 2010 |
| *Colloderma oculatum* (C. Lippert) G. Lister, 1910 | Jarocki, 1931; Krzemieniewska, 1960b; Stojanowska, 2004b |
| *Comatricha dictyospora* L.F. Celak., 1893 | Stojanowska, 1977 |
| *Comatricha elegans* (Racib.) G. Lister, 1909 | Drozdowicz et al., 2012; Drozdowicz et al., 2007; Jarocki, 1931; Panek and Romański, 2010; Salamaga, 2021; Stojanowska 1983; Stojanowska and Panek, 2004 |
| *Comatricha ellae* Härk., 1978 | Drozdowicz, 2017 |
| *Comatricha fusiformis* (Kowalski) Kowalski, 1968 | Magiera and Drozdowicz, 2004 |
| *Comatricha laxa* Rostaf., 1874 | Krupa, 1889; Jarocki, 1927; Krzemieniewska, 1933; Krzemieniewska, 1957; Krzemieniewska, 1960b; Stojanowska, 1972; Stojanowska 1983; Stojanowska, 1983; Drozdowicz, 1992; Stojanowska, 1992; Drozdowicz, 1997; Drozdowicz, 1997a; Stojanowska, 2004; Salamaga, 2021; Paul et al., 2023 |
| *Comatricha nigra* (Pers. ex J. F. Gmel.) J. Schröt., 1885 | Krupa, 1886; Krupa, 1889; Jarocki, 1924; Jarocki, 1927; Jarocki, 1931; Krzemieniewska, 1933; Krzemieniewska, 1947; Michalski, 1951; Krzemieniewska, 1957; Krawiec, 1965; Stojanowska, 1970; Stojanowska, 1972; Kalinowska-Kucharska, 1975; Kalinowska-Kucharska, 1975; Stojanowska, 1977; Stojanowska, 1977b; Stojanowska, 1980a; Stojanowska, 1980b; Stojanowska 1983; Stojanowska, 1983; Stojanowska, 1984; Drozdowicz, 1992; Stojanowska, 1992; Stojanowska, 2000a; Miśkiewicz, 2001; Stojanowska and Panek, 2002; Drozdowicz, 2003a; Stojanowska, 2004; Stojanowska and Panek, 2004; Drozdowicz, 2005; Drozdowicz et al., 2007; Panek and Romański, 2010; Ślusarczyk, 2010; Bochynek and Drozdowicz, 2011; Ławrynowicz et al., 2011; Bochynek & Drozdowicz, 2012; Drozdowicz et al., 2012; Paul et al., 2023; Pawłowicz et al., 2025 |
| *Comatricha pulchella* (C. Bab.) Rostaf., 1876 | Jarocki, 1924; Jarocki, 1931; Krzemieniewska, 1933; Krzemieniewska, 1960b; Stojanowska, 1977; Stojanowska, 1977b; Stojanowska 1983; Stojanowska, 1983; Stojanowska, 1984; Drozdowicz, 1992; Stojanowska, 2000a; Stojanowska, 2004; Stojanowska and Panek, 2004; Panek and Romański, 2010; Ławrynowicz et al., 2011; Salamaga, 2021 |
| *Craterium aureonucleatum* Nann. -Bremek., 1961 | Panek and Romański, 2010 |
| *Craterium aureum* (Schumach.) Rostaf., 1874 | Rostafiński, 1874; Jarocki, 1924; Krzemieniewska, 1960b; Stojanowska 1983; Stojanowska and Panek, 2002; Stojanowska and Panek, 2003; Stojanowska and Panek, 2004; Panek and Romański, 2010; Salamaga, 2021 |
| *Craterium concinnum* Rex, 1893 | Panek and Romański, 2010; Stojanowska and Panek, 2003 |
| *Craterium leucocephalum* (Pers. ex J.F. Gmel.) Ditmar, 1813 | Jarocki, 1924; Krzemieniewska and Badura, 1954; Krzemieniewska, 1957; Krzemieniewska, 1960b; Stojanowska, 1972; Kalinowska-Kucharska, 1975; Kalinowska-Kucharska, 1975; Stojanowska, 1980a; Stojanowska, 1980b; Stojanowska 1983; Stojanowska, 1983; Drozdowicz, 1992; Drozdowicz, 1997; Drozdowicz, 1997a; Stojanowska and Panek, 2002; Stojanowska and Panek, 2003; Stojanowska, 2004; Stojanowska and Panek, 2004; Drozdowicz et al., 2007; Panek and Romański, 2010; Ławrynowicz et al., 2011; Paul et al., 2023 |
| *Craterium minutum* (Leers) Fr., 1829 | Rostafiński, 1874; Jarocki, 1924; Krzemieniewska, 1933; Krzemieniewska, 1960b; Stojanowska, 1972; Stojanowska, 1977; Stojanowska, 1977b; Stojanowska, 1980a; Stojanowska, 1980b; Stojanowska 1983; Stojanowska, 1983; Drozdowicz, 1992; Drozdowicz, 1997; Drozdowicz, 1997a; Stojanowska and Panek, 2003; Stojanowska, 2004; Stojanowska and Panek, 2004; Drozdowicz et al., 2007; Panek and Romański, 2010; Bochynek and Drozdowicz, 2011; Ławrynowicz et al., 2011 |
| *Craterium roseum* (Berk. & Broome) J.M. García-Martín & Lado, 2023 | Paul et al., 2023 |
| *Cribraria argillacea* (Pers. ex J.F. Gmel.) Pers., 1794 | Krupa, 1886; Krupa, 1887; Błoński and Drymmer, 1889; Krupa, 1889; Eichler, 1907; Jarocki, 1924; Jarocki, 1931; Krzemieniewska, 1933; Krzemieniewska, 1947; Krzemieniewska and Badura, 1954; Krzemieniewska, 1957; Krawiec, 1965; Stojanowska, 1972; Kalinowska-Kucharska, 1975; Stojanowska, 1977b; Stojanowska 1983; Stojanowska, 1983; Stojanowska, 1984; Drozdowicz, 1992; Stojanowska, 1992; Stojanowska, 2000a; Miśkiewicz, 2001; Drozdowicz and Wilga, 2002; Stojanowska, 2004a; Stojanowska, 2004b; Stojanowska and Panek, 2004; Stojanowska and Panek, 2005; Drozdowicz et al., 2007; Panek and Romański, 2010; Bochynek and Drozdowicz, 2011; Ławrynowicz et al., 2011; Bochynek & Drozdowicz, 2012; Drozdowicz et al., 2012; Salamaga et al., 2016; Chachuła et al., 2021; Salamaga, 2021; Paul et al., 2023 |
| *Cribraria aurantiaca* Schrad., 1797 | Namysłowski, 1910; Jarocki, 1924; Krzemieniewska, 1957; Stojanowska, 1977b; Stojanowska 1983; Stojanowska, 1983; Stojanowska, 1984; Stojanowska, 1992; Miśkiewicz, 2001; Stojanowska, 2004a; Stojanowska, 2004b; Stojanowska and Panek, 2004; Stojanowska and Panek, 2005; Drozdowicz et al., 2007; Panek and Romański, 2010; Ławrynowicz et al., 2011; Salamaga et al., 2016; Salamaga, 2021; Paul et al., 2023 |
| *Cribraria cancellata* (Batsch) Nann. -Bremek., 1975 | Krupa, 1886; Krupa, 1887; Eichler, 1904; Namysłowski, 1910; Jarocki, 1924; Jarocki, 1931; Krzemieniewska, 1933; Krzemieniewska, 1947; Krzemieniewska and Badura, 1954; Krzemieniewska, 1957; Stojanowska, 1970; Stojanowska, 1972; Wrońska, 1974; Stojanowska, 1977b; Stojanowska, 1981; Stojanowska 1983; Stojanowska, 1983; Stojanowska, 1984; Drozdowicz, 1992; Stojanowska, 1992; Stojanowska, 2000a; Stojanowska, 2004a; Stojanowska, 2004b; Stojanowska and Panek, 2004; Stojanowska and Panek, 2005; Drozdowicz et al., 2007; Panek and Romański, 2010; Bochynek and Drozdowicz, 2011; Ławrynowicz et al., 2011; Drozdowicz et al., 2012; Salamaga et al., 2016; Salamaga, 2021; Paul et al., 2023 |
| *Cribraria costata* Dhillon & Nann. -Bremek., 1978 | Bochynek, 2015 |
| *Cribraria elegans* Berk. & M.A. Curtis, 1873 | Bochynek, 2015 |
| *Cribraria exigua* Meyl., 1931 | Magiera and Drozdowicz, 2004 |
| *Cribraria ferruginea* Meyl., 1913 | Jarocki, 1931; Krzemieniewska, 1960b; Stojanowska 1983; Stojanowska, 1983; Drozdowicz, 1992; Stojanowska, 2004a; Stojanowska, 2004b; Panek and Romański, 2010; Bochynek and Drozdowicz, 2011; Bochynek & Drozdowicz, 2012 |
| *Cribraria intricata* Schrad., 1797 | Jarocki, 1924; Krzemieniewska, 1933; Krzemieniewska, 1957; Krzemieniewska, 1960b; Stojanowska, 1972; Wrońska, 1974; Stojanowska 1983; Stojanowska, 1983; Stojanowska, 2004a; Stojanowska, 2004b; Stojanowska and Panek, 2005; Panek and Romański, 2010; Salamaga et al., 2016 |
| *Cribraria languescens* Rex, 1891 | Bochynek, 2015; Salamaga et al., 2016 |
| *Cribraria macrocarpa* Schrad., 1797 | Krzemieniewska, 1947; Krzemieniewska, 1957; Krzemieniewska, 1960b; Krawiec, 1965; Stojanowska, 1972; Wrońska, 1974; Stojanowska 1983; Stojanowska, 1983; Stojanowska, 1984; Drozdowicz, 1992; Stojanowska, 1992; Stojanowska, 2000a; Miśkiewicz, 2001; Drozdowicz, 2003a; Stojanowska, 2004a; Drozdowicz, 2005; Drozdowicz et al., 2007; Bochynek and Drozdowicz, 2011; Ławrynowicz et al., 2011; Bochynek & Drozdowicz, 2012; Drozdowicz et al., 2012; Paul et al., 2023 |
| *Cribraria macrospora* Nowotny & H. Neubert, 1993 | Paul et al., 2023 |
| *Cribraria microcarpa* (Schrad.) Pers., 1801 | Jarocki, 1931; Krzemieniewska, 1947; Krzemieniewska, 1957; Krzemieniewska, 1960b; Stojanowska, 1972; Stojanowska 1983; Drozdowicz, 1992; Stojanowska and Panek, 2004; Drozdowicz, 2005; Panek and Romański, 2010; Pawłowicz et al., 2025 |
| *Cribraria minutissima* Schwein., 1832 | Stojanowska, 1972; Stojanowska 1983 |
| *Cribraria mirabilis* (Rostaf.) Massee, 1892 | Bochynek, 2015 |
| *Cribraria oregana* H.C. Gilbert, 1932 | Magiera and Drozdowicz, 2004 |
| *Cribraria pertenuis* Flatau & Schirmer, 1994 | Bochynek, 2015; Salamaga et al., 2016 |
| *Cribraria piriformis* Schrad., 1797 | Krzemieniewska, 1947; Krzemieniewska, 1957; Krzemieniewska, 1960b; Stojanowska 1983; Stojanowska, 1983; Stojanowska, 1984; Drozdowicz, 1992; Stojanowska, 2000a; Stojanowska, 2002; Stojanowska and Panek, 2002; Stojanowska, 2004; Drozdowicz, 2005; Panek and Romański, 2010; Bochynek and Drozdowicz, 2011; Bochynek & Drozdowicz, 2012; Drozdowicz et al., 2012; Salamaga, 2021 |
| *Cribraria purpurea* Schrad., 1797 | Jarocki, 1924; Jarocki, 1931; Krzemieniewska, 1947; Krzemieniewska, 1960b; Krzysztofiak et al., 2010; Chachuła et al., 2021; Paul et al., 2023 |
| *Cribraria rubiginosa* Fr., 1829 | Jarocki, 1931; Krzemieniewska, 1960b; Stojanowska 1983; Stojanowska, 1983; Stojanowska, 2004 |
| *Cribraria rufa* (Roth) Rostaf., 1875 | Rostafiński, 1875; Jarocki, 1924; Krzemieniewska, 1957; Krzemieniewska, 1960b; Stojanowska, 1970; Stojanowska, 1972; Kalinowska-Kucharska, 1975; Kalinowska-Kucharska, 1975; Stojanowska, 1977; Stojanowska, 1977b; Stojanowska, 1983; Stojanowska, 1984; Drozdowicz, 1992; Drozdowicz, 1997; Drozdowicz, 1997a; Stojanowska, 2000a; Drozdowicz, 2001; Stojanowska, 2002; Drozdowicz, 2003a; Stojanowska, 2004; Drozdowicz, 2005; Stojanowska, 2005; Drozdowicz et al., 2007; Krzysztofiak et al., 2010; Panek and Romański, 2010; Bochynek and Drozdowicz, 2011; Ławrynowicz et al., 2011; Bochynek & Drozdowicz, 2012; Drozdowicz et al., 2012; Salamaga et al., 2016; Salamaga, 2021; Paul et al., 2023 |
| *Cribraria splendens* (Schrad.) Pers., 1801 | Krzemieniewska, 1933; Krzemieniewska, 1947; Krzemieniewska, 1957; Krzemieniewska, 1960b; Wrońska, 1974; Stojanowska, 1977; Stojanowska, 1977b; Stojanowska, 1983; Stojanowska, 1984; Stojanowska, 1992; Stojanowska, 2004; Drozdowicz, 2005; Stojanowska, 2005; Panek and Romański, 2010; Salamaga et al., 2016; Salamaga, 2021; Paul et al., 2023 |
| *Cribraria tenella* Schrad., 1797 | Rostafiński, 1875; Jarocki, 1924; Krzemieniewska, 1933; Krzemieniewska, 1960b; Kalinowska-Kucharska, 1975; Kalinowska-Kucharska, 1975; Stojanowska, 1992; Bochynek and Drozdowicz, 2011; Ławrynowicz et al., 2011; Bochynek & Drozdowicz, 2012; Drozdowicz et al., 2012 |
| *Cribraria tubulina* (Fr.) J.C. Zamora, D. Rodrigues, García-Cunch. & Lado, 2025 | Krupa, 1886, 1887, 1888; Rostafiński, 1875; Jarocki, 1924; Kalinowska-Kucharska, 1975; Krzemieniewska, 1933, 1947, 1957, 1960b; Orzechowski, 1969; Stojanowska, 1972, 1977, 1977b, 1983, 1984, 2000a; Drozdowicz, 1992, 2005; Ławrynowicz et al., 2011; Panek and Romański, 2010; Stojanowska 1983; Drozdowicz et al., 2007; Bochynek and Drozdowicz, 2011; Stojanowska, 2004a, 2004b; Bochynek & Drozdowicz, 2012; Chachuła et al., 2021 |
| *Cribraria violacea* Rex, 1891 | Krzemieniewska, 1933; Krzemieniewska, 1934; Krzemieniewska, 1960b; Stojanowska, 2005 |
| *Cribraria vulgaris* Schrad., 1797 | Rostafiński, 1875; Gutwiński, 1901; Krzemieniewska, 1933; Krzemieniewska, 1947; Krzemieniewska, 1957; Krawiec, 1965; Stojanowska, 1972; Kalinowska-Kucharska, 1975; Kalinowska-Kucharska, 1975; Stojanowska, 1977; Stojanowska, 1977b; Stojanowska, 1980a; Stojanowska, 1980b; Stojanowska, 1983; Stojanowska, 1984; Drozdowicz, 1992; Stojanowska, 2002; Stojanowska, 2004; Drozdowicz, 2005; Drozdowicz et al., 2007; Panek and Romański, 2010; Bochynek and Drozdowicz, 2011; Ławrynowicz et al., 2011; Bochynek & Drozdowicz, 2012; Drozdowicz et al., 2012; Salamaga, 2021; Paul et al., 2023 |
| *Diachea leucopodia* (Bull.) Rostaf., 1874 | Błoński and Drymmer, 1889; Kwieciński, 1896; Bothe and Torka, 1906; Jarocki, 1924; Krzemieniewska, 1957; Golenia and Rebandel, 1970; Stojanowska, 1972; Kalinowska-Kucharska, 1975; Stojanowska, 1980a; Stojanowska, 1980b; Stojanowska 1983; Stojanowska, 1983; Stojanowska, 1984; Bujakiewicz and Fiebich, 1992; Drozdowicz, 1992; Stojanowska, 1992; Drozdowicz, 1997; Drozdowicz, 1997a; Bujakiewicz, 1999; Stojanowska and Panek, 2002; Stojanowska, 2004; Stojanowska and Panek, 2004; Stojanowska and Panek, 2005; Drozdowicz et al., 2007; Krzysztofiak et al., 2010; Panek and Romański, 2010; Ślusarczyk, 2010; Bochynek and Drozdowicz, 2011; Ławrynowicz et al., 2011; Drozdowicz et al., 2012; Ślusarczyk, 2021; Paul et al., 2023 |
| *Diachea muscorum* (Ing) J.M. García-Martín, J.C. Zamora & Lado, 2023 | Stojanowska and Panek, 2003 |
| *Diachea obovata* (Peck) J.M. García-Martín, J.C. Zamora & Lado, 2023 | Schroeter, 1889; Szulczewski, 1951; Stojanowska 1983 |
| *Diachea subsessilis* Peck, 1878 | Krupa, 1886; Krupa, 1888 |
| *Diacheopsis pieninica* Krzemien., 1948 | Krzemieniewska, 1947; Krzemieniewska, 1960b; Drozdowicz et al., 2003 |
| *Dianema depressum* (Lister) Lister, 1894 | Stojanowska, 2004a |
| *Dictydiaethalium plumbeum* (Schumach.) Rostaf. ex Lister, 1894 | Krupa, 1886; Krupa, 1887; Bresadola, 1903; Eichler, 1907; Jarocki, 1924; Krzemieniewska, 1947; Krzemieniewska, 1957; Krzemieniewska, 1960b; Stojanowska, 1977a; Stojanowska, 1977b; Stojanowska 1983; Stojanowska, 1983; Stojanowska, 1984; Drozdowicz, 1992; Stojanowska and Panek, 2002; Stojanowska, 2004a; Stojanowska and Panek, 2005; Drozdowicz et al., 2007; Panek and Romański, 2010; Drozdowicz et al., 2012; Chachuła et al., 2021; Salamaga, 2021; Paul et al., 2023 |
| *Diderma alpinum* (Meyl.) Meyl., 1917 | Drozdowicz, 1985; Drozdowicz, 1988; Komorowska and Drozdowicz, 1996; Drozdowicz, 1997; Drozdowicz, 1997a; Drozdowicz, 1997b; Drozdowicz, 2001; Stojanowska, 2004; Ronikier et al., 2008; Paul et al., 2023 |
| *Diderma chondrioderma* (de Bary & Rostaf.) Kuntze, 1898 | Rostafiński, 1874; Krzemieniewska, 1933; Krzemieniewska, 1934; Krzemieniewska, 1960b; Miśkiewicz and Drozdowicz, 1999; Miśkiewicz, 2001; Stojanowska and Panek, 2004 |
| *Diderma crustaceum* Peck, 1873 | Stojanowska, 1970; Stojanowska, 1972; Drozdowicz et al., 2007 |
| *Diderma deplanatum* Fr., 1829 | Migula, 1910; Jarocki, 1924; Miśkiewicz and Drozdowicz, 1999; Stojanowska, 2000a; Stojanowska, 2000b; Miśkiewicz, 2001; Stojanowska and Panek, 2002; Stojanowska and Panek, 2004; Panek and Romański, 2010; Salamaga et al., 2016 |
| *Diderma effusum* (Schwein.) Morgan, 1894 | Stojanowska 1983; Stojanowska, 1983; Drozdowicz, 1992; Stojanowska, 2000a; Miśkiewicz, 2001; Stojanowska and Panek, 2002; Stojanowska, 2004; Stojanowska and Panek, 2004; Drozdowicz et al., 2012 |
| *Diderma europaeum* (Buyck) A. Kuhnt, 2017 | Ronikier et al., 2008 |
| *Diderma floriforme* (Bull.) Pers., 1794 | Krzemieniewska, 1960b; Stojanowska, 2000a; Stojanowska and Panek, 2002; Stojanowska, 2004; Drozdowicz et al., 2012 |
| *Diderma globosum* Pers., 1794 | Rostafiński, 1874; Rostafiński, 1876; Jarocki, 1924; Drozdowicz, 1997; Drozdowicz, 1997a |
| *Diderma hemisphaericum* (Bull.) Hornem., 1829 | Rostafiński, 1874; Krzemieniewska, 1929; Krzemieniewska, 1933; Krzemieniewska, 1960b; Drozdowicz et al., 2003 |
| *Diderma montanum* (Meyl.) Meyl., 1913 | Miśkiewicz, 2001; Drozdowicz, 2003a; Stojanowska and Panek, 2005; Drozdowicz et al., 2007; Panek and Romański, 2010 |
| *Diderma niveum* (Rostaf.) E. Sheld., 1895 | Drozdowicz, 1997b; Drozdowicz, 2001; Stojanowska, 2004; Ronikier et al., 2008 |
| *Diderma ochraceum* Hoffm., 1795 | Krzemieniewska, 1960b; Stojanowska, 1972; Stojanowska, 1977; Stojanowska 1983 |
| *Diderma radiatum* (L.) Morgan, 1894 | Rostafiński, 1874; Jarocki, 1924; Krzemieniewska, 1947; Krzemieniewska and Badura, 1954; Krzemieniewska, 1957; Krzemieniewska, 1960b; Stojanowska 1983; Stojanowska, 1983; Miśkiewicz, 2001; Stojanowska, 2004a; Stojanowska, 2004b; Stojanowska and Panek, 2004; Panek and Romański, 2010; Ławrynowicz et al., 2011 |
| *Diderma roanense* (Rex) T. Macbr., 1899 | Stojanowska and Panek, 2005 |
| *Diderma saundersii* (Berk. & Broome ex Massee) E.Sheld., 1895 | Salamaga, 2021 |
| *Diderma simplex* (J. Schröt.) E. Sheld., 1895 | Migula, 1910; Krzemieniewska, 1933; Krzemieniewska, 1934; Krawiec, 1965; Stojanowska 1983; Stojanowska and Panek, 2004 |
| *Diderma testaceum* (Schrad.) Pers., 1801 | Jarocki, 1924; Krzemieniewska, 1957; Krzemieniewska, 1960b; Stojanowska, 1980a; Stojanowska, 1980b; Drozdowicz, 2003a; Stojanowska and Panek, 2004; Stojanowska, 2004b; Drozdowicz et al., 2007; Panek and Romański, 2010; Drozdowicz et al., 2012; Salamaga et al., 2016; Paul et al., 2023 |
| *Diderma tigrinum* (Schrad.) Prikhodko, Shchepin, Novozh., López-Vill., G. Moreno & Schnittler, 2023 | Krupa, 1889; Jarocki, 1924; Krzemieniewska, 1947; Krzemieniewska, 1960b; Stojanowska and Panek, 2004; Stojanowska and Panek, 2005; Panek and Romański, 2010; Chachuła et al., 2021; Paul et al., 2023 |
| *Diderma umbilicatum* Pers., 1801 | Krzemieniewska, 1960b; Kalinowska-Kucharska, 1975; Stojanowska, 2004b; Stojanowska and Panek, 2004; Ławrynowicz et al., 2011 |
| *Didymium anellus* Morgan, 1894 | Panek and Romański, 2010 |
| *Didymium clavus* (Alb. & Schwein.) Rabenh., 1844 | Jarocki, 1924; Krzemieniewska, 1960b; Wrońska, 1974; Stojanowska, 1977; Stojanowska, 1977b; Stojanowska 1983; Stojanowska, 1983; Stojanowska, 1984; Drozdowicz, 1992; Panek and Romański, 2010; Drozdowicz et al., 2012 |
| *Didymium comatum* (Lister) Nann. -Bremek., 1966 | Rostafiński, 1874; Migula, 1910; Krzemieniewska, 1933; Krzemieniewska, 1934; Krzemieniewska, 1960b; Drozdowicz et al., 2003 |
| *Didymium crustaceum* Fr., 1829 | Stojanowska, 1970; Stojanowska, 1972; Stojanowska and Panek, 2005; Panek and Romański, 2010 |
| *Didymium difforme* (Pers.) Gray, 1821 | Jarocki, 1924; Krzemieniewska, 1929; Krzemieniewska, 1933; Michalski, 1951; Krzemieniewska and Badura, 1954; Krzemieniewska, 1957; Krawiec, 1965; Stojanowska 1983; Stojanowska, 1992; Paul et al., 2023 |
| *Didymium dubium* Rostaf., 1874 | Ronikier et al., 2008 |
| *Didymium eximium* Peck, 1878 | Salamaga, 2021 |
| *Didymium iridis* (Ditmar) Fr., 1829 | Jarocki, 1924; Krzemieniewska, 1933; Krzemieniewska, 1957; Stojanowska, 1972; Stojanowska, 1980a; Stojanowska, 1980b; Stojanowska 1983; Stojanowska, 1983; Stojanowska, 1984; Stojanowska and Panek, 2002; Stojanowska, 2004a; Stojanowska and Panek, 2005; Panek and Romański, 2010; Ławrynowicz et al., 2011 |
| *Didymium leptotrichum* (Racib.) Massee, 1892 | Raciborski, 1884a; Raciborski, 1884b; Ławrynowicz et al., 2011 |
| *Didymium macrospermum* Rostaf., 1874 | Raciborski, 1884b; Schroeter, 1889; Drozdowicz et al., 2003 |
| *Didymium megalosporum* Berk. & M.A. Curtis, 1873 | Salamaga, 2021 |
| *Didymium melanospermum* (Pers.) T. Macbr., 1899 | Jarocki, 1924; Krzemieniewska, 1933; Krzemieniewska, 1957; Stojanowska, 1972; Wrońska, 1974; Kalinowska-Kucharska, 1975; Stojanowska 1983; Stojanowska, 1983; Drozdowicz, 1992; Drozdowicz, 1997; Drozdowicz, 1997a; Fałtynowicz, 2003; Stojanowska and Panek, 2004; Stojanowska, 2004; Stojanowska and Panek, 2005; Panek and Romański, 2010; Ślusarczyk, 2010; Ławrynowicz et al., 2011 |
| *Didymium minus* (Lister) Morgan, 1894 | Krzemieniewska, 1933; Krzemieniewska, 1957; Krzemieniewska, 1960b; Stojanowska, 1972; Wrońska, 1974; Kalinowska-Kucharska, 1975; Stojanowska, 1977; Stojanowska, 1977b; Stojanowska 1983; Stojanowska, 1984; Drozdowicz, 1992; Stojanowska, 2004; Panek and Romański, 2010; Ławrynowicz et al., 2011; Paul et al., 2023 |
| *Didymium nigripes* (Link) Fr., 1829 | Jarocki, 1924; Krzemieniewska, 1929; Krzemieniewska, 1933; Krzemieniewska and Badura, 1954; Krzemieniewska, 1957; Krzemieniewska, 1960b; Stojanowska, 1972; Stojanowska 1983; Stojanowska, 1984; Drozdowicz, 1992; Stojanowska and Panek, 2004; Stojanowska, 2004; Drozdowicz et al., 2007; Panek and Romański, 2010; Drozdowicz et al., 2012; Salamaga, 2021; Paul et al., 2023 |
| *Didymium nigrum* Krzemien., 1960 | Krzemieniewska, 1960a; Drozdowicz et al., 2003 |
| *Didymium serpula* Fr., 1829 | Jarocki, 1924; Krzemieniewska, 1960b; Stojanowska and Panek, 2004; Panek and Romański, 2010; Salamaga, 2021 |
| *Didymium spongiosum* (Leyss.) J.M. García-Martín, J.C. Zamora & Lado, 2023 | Błoński and Drymmer, 1889; Błoński et al., 1888; Jarocki, 1924; Kalinowska-Kucharska, 1975; Panek and Romański, 2010; Paul et al., 2023; Stojanowska 1983; Stojanowska, 1977; Stojanowska, 2004; Ślusarczyk, 2021; Błoński, 1888; Krzemieniewska, 1957, 1960b; Zweigbaumówna, 1924; Drozdowicz, 1992; Stojanowska, 1972, 1977b, 1983; Ławrynowicz et al., 2011 |
| *Didymium squamulosum* (Alb. & Schwein.) Fr. & Palmq., 1818 | Krupa, 1889; Jarocki, 1924; Krzemieniewska, 1929; Krzemieniewska, 1933; Michalski, 1951; Krzemieniewska and Badura, 1954; Krzemieniewska, 1960b; Stojanowska, 1972; Wrońska, 1974; Kalinowska-Kucharska, 1975; Stojanowska, 1977; Stojanowska, 1977b; Stojanowska, 1980a; Stojanowska, 1980b; Stojanowska 1983; Stojanowska, 1984; Drozdowicz, 1992; Drozdowicz, 1997; Drozdowicz, 1997a; Stojanowska and Panek, 2002; Fałtynowicz, 2003; Stojanowska and Panek, 2004; Stojanowska, 2004; Stojanowska and Panek, 2005; Drozdowicz et al., 2007; Panek and Romański, 2010; Ławrynowicz et al., 2011; Paul et al., 2023 |
| *Didymium trachysporum* G. Lister, 1923 | Krzemieniewska, 1929; Krzemieniewska, 1933; Krzemieniewska, 1934; Drozdowicz et al., 2003 |
| *Echinostelium minutum* de Bary in Rostaf., 1874 | Drozdowicz, 2017 |
| *Elaeomyxa cerifera* (G. Lister) Hagelst., 1942 | Krzemieniewska, 1960b; Magiera and Drozdowicz, 2004 |
| *Enerthenema berkeleyanum* Rostaf., 1876 | Drozdowicz et al., 2003; Raciborski, 1884b; Tabacki, 1977 |
| *Enerthenema intermedium* Nann. -Bremek. & R.L. Critchf., 1988 | Drozdowicz et al., 2003; Krzemieniewska, 1960b |
| *Enerthenema papillatum* (Pers.) Rostaf., 1876 | Błoński and Drymmer, 1889; Drozdowicz et al., 2007; Jarocki, 1924; Kalinowska-Kucharska, 1975; Krupa, 1889; Ławrynowicz et al., 2011; Panek and Romański, 2010; Paul et al., 2023; Salamaga et al., 2016; Stojanowska 1983; Stojanowska, 1980a; Stojanowska, 2004; Stojanowska, 2004; Stojanowska and Panek, 2002; Stojanowska and Panek, 2004; Drozdowicz, 1992; Jarocki, 1924, 1927; Kalinowska-Kucharska, 1975; Krzemieniewska, 1933, 1947, 1957, 1960b; Krzemieniewska and Badura, 1954; Stojanowska, 1972, 1977b, 1980b, 1983, 1984, 1992, 1998, 2000a; Stojanowska and Panek, 2002 |
| *Enteridium corticatum* (Lister.) J.C. Zamora, D. Rodrigues, García-Cunch. & Lado, 2025 | Stojanowska, 1977; Stojanowska, 2004; Krzemieniewska, 1957, 1960b |
| *Enteridium olivaceum* Ehrenb., 1819 | Panek and Romański, 2010; Paul et al., 2023; Stojanowska 1983; Stojanowska, 1977 |
| *Enteridium variabile* (Schrad.) J.C. Zamora, D. Rodrigues, García-Cunch. & Lado, 2025 | Chachuła et al., 2021; Jarocki, 1931; Ronikier et al., 2017; Salamaga et al., 2016; Salamaga, 2021; Stojanowska 1983; Stojanowska, 1977; Stojanowska, 1980a; Stojanowska, 2004; Stojanowska, 2004; Stojanowska, 2004; Drozdowicz, 1992; Krzemieniewska, 1933, 1957, 1960b; Rostafiński, 1875; Stojanowska, 1980b, 1983, 1984 |
| *Fuligo cinerea* (Schwein.) Morgan, 1896 | Drozdowicz et al., 2003; Szulczewski, 1951 |
| *Fuligo gyrosa* (Rostaf.) E. Jahn, 1902 | Ławrynowicz et al., 2011; Stojanowska 1983; Stojanowska and Panek, 2004 |
| *Fuligo intermedia* T. Macbr., 1922 | Panek and Romański, 2010 |
| *Fuligo leviderma* H. Neubert, Nowotny & K. Baumann, 1995 | Bochynek and Drozdowicz, 2011; Drozdowicz, 2005; Drozdowicz et al., 2012; Drozdowicz et al., 2007; Ławrynowicz et al., 2011; Miśkiewicz, 2001; Panek and Romański, 2010; Paul et al., 2023; Salamaga, 2021; Stojanowska, 2004a; Stojanowska, 2004b; Stojanowska and Panek, 2002; Stojanowska and Panek, 2005; Stojanowska and Panek, 2004; Ślusarczyk, 2010; Ślusarczyk, 2021; Wilga and Ciechanowski, 2007; Drozdowicz, 1997a; Jarocki, 1924; Kalinowska-Kucharska, 1975; Krzemieniewska, 1933, 1957, 1960b; Miśkiewicz, 2001; Stojanowska, 1972, 1977b, 1980b, 1983, 1984, 2000c; Stojanowska and Panek, 2002 |
| *Fuligo licentii* Buchet, 1939 | Paul et al., 2023 |
| *Fuligo luteonitens* L.G. Krieglst. & Nowotny, 1995 | Panek and Romański, 2010; Ślusarczyk, 2021 |
| *Fuligo septica* (L.) F. H. Wigg., 1780 | Błoński, 1890; Błoński and Drymmer, 1889; Błoński et al., 1888; Bochynek & Drozdowicz, 2012; Bochynek and Drozdowicz, 2011; Čelakovský, 1890; Chachuła et al., 2021; Drozdowicz, 1997; Drozdowicz, 2005; Drozdowicz et al., 2012; Drozdowicz et al., 2007; Gutwiński, 1901; Kalinowska-Kucharska, 1975; Krupa, 1887; Ławrynowicz et al., 2011; Miśkiewicz, 2001; Panek and Romański, 2010; Paul et al., 2023; Salamaga et al., 2016; Salamaga, 2021; Stojanowska 1983; Stojanowska, 1977a; Stojanowska, 1977b; Stojanowska, 1980a; Stojanowska, 2004a; Stojanowska, 2004b; Stojanowska and Panek, 2002; Stojanowska and Panek, 2005; Stojanowska and Panek, 2004; Ślusarczyk, 2010; Wilga and Ciechanowski, 2007; Wrońska, 1974; Błoński, 1888, 1890; Bresadola, 1903; Drozdowicz, 1992, 2003a; Drozdowicz and Wilga, 2002; Eichler, 1904; Gutwiński, 1901; Jarocki, 1924; Kalinowska-Kucharska, 1975; Komorowska and Drozdowicz, 1996; Krawiec, 1965; Krupa, 1886, 1888; Krzemieniewska, 1933, 1947, 1957; Krzemieniewska and Badura, 1954; Michalski, 1951; Miśkiewicz, 2001; Namysłowski, 1910, 1914; Stecki, 1910; Steinecke, 1918; Stojanowska, 1970, 1972, 1977b, 1980b, 1983, 1984, 1992, 2000a; Stojanowska and Panek, 2002; Wrońska, 1974 |
| *Gulielmina vermicularis* (Schwein.) García-Cunch., J.C. Zamora & Lado, 2022 | Drozdowicz, 2017; Raciborski, 1884b |
| *Hemitrichia abietina* (Wigand) G. Lister, 1911 | Drozdowicz, 1997; Stojanowska 1983; Drozdowicz, 1992, 1997a; Krzemieniewska, 1960b; Stojanowska, 1972 |
| *Hemitrichia calyculata* (Speg.) M. L. Farr, 1974 | Bochynek and Drozdowicz, 2011; Drozdowicz, 2005; Miśkiewicz, 2001; Panek and Romański, 2010; Salamaga, 2021; Stojanowska, 2004a; Stojanowska and Panek, 2005; Wilga and Ciechanowski, 2007; Drozdowicz, 2003a; Miśkiewicz, 2001 |
| *Hemitrichia chrysospora* (Lister) Lister, 1894 | Stojanowska 1983; Stojanowska and Panek, 2004; Firich, 1962 |
| *Hemitrichia clavata* (Pers.) Rostaf., 1873 | Bochynek and Drozdowicz, 2011; Drozdowicz, 2005; Drozdowicz et al., 2012; Drozdowicz et al., 2007; Jarocki, 1924; Krupa, 1887; Ławrynowicz et al., 2011; Miśkiewicz, 2001; Panek and Romański, 2010; Paul et al., 2023; Salamaga, 2021; Stojanowska 1983; Stojanowska, 1977a; Stojanowska, 2004a; Stojanowska, 2004b; Stojanowska and Panek, 2002; Stojanowska and Panek, 2005; Stojanowska and Panek, 2004; Ślusarczyk, 2010; Wrońska, 1974; Drozdowicz, 1992, 1997b, 2001, 2003a; Jarocki, 1924; Krawiec, 1965; Krupa, 1886, 1888; Krzemieniewska, 1933, 1947, 1957, 1960b; Krzemieniewska and Badura, 1954; Migula, 1910; Miśkiewicz, 2001; Stojanowska, 1970, 1972, 1977b, 1983, 1984, 1998; Stojanowska and Panek, 2002; Wrońska, 1974 |
| *Hemitrichia decipiens* (Pers.) García-Cunch., J.C. Zamora & Lado, 2022 | Bochynek & Drozdowicz, 2012; Bochynek and Drozdowicz, 2011; Chachuła et al., 2021; Drozdowicz, 2005; Drozdowicz et al., 2012; Drozdowicz et al., 2007; Jarocki, 1924; Kalinowska-Kucharska, 1975; Krupa, 1887; Krupa, 1889; Ławrynowicz et al., 2011; Panek and Romański, 2010; Paul et al., 2023; Salamaga, 2021; Stojanowska 1983; Stojanowska, 2004a; Stojanowska, 2004b; Stojanowska and Panek, 2002; Stojanowska and Panek, 2005; Stojanowska and Panek, 2004; Ślusarczyk, 2010; Wrońska, 1974; Drozdowicz, 1992; Drozdowicz, 1997b; Drozdowicz, 2003a; Jarocki, 1924; Kalinowska-Kucharska, 1975; Krawiec, 1965; Krupa, 1886; Krzemieniewska, 1929; Krzemieniewska, 1933; Krzemieniewska, 1947; Krzemieniewska, 1957; Krzemieniewska, 1960b; Stojanowska, 1972; Stojanowska, 1977b; Stojanowska, 1980b; Stojanowska, 1983; Stojanowska, 1984; Stojanowska, 2000a; Wrońska, 1974 |
| *Hemitrichia intorta* (Lister) Lister, 1894 | Drozdowicz, 2017; Krzemieniewska, 1957, 1960b |
| *Hemitrichia karstenii* (Rostaf.) Lister, 1894 | Krupa, 1889 |
| *Hemitrichia leiocarpa* (Cooke) Lister, 1894 | Wrońska, 1974; Raciborski, 1884a, 1884b |
| *Hemitrichia leiotricha* (Lister) G. Lister, 1911 | Stojanowska 1983; Stojanowska, 1977b; Krzemieniewska, 1960b; Stojanowska, 1972, 1983 |
| *Hemitrichia lutescens* (Lister) García-Cunch., J.C. Zamora & Lado, 2022 | Drozdowicz, 2017; Tabacki, 1977 |
| *Hemitrichia serpula* (Scop.) Rostaf. ex Lister, 1894 | Bochynek and Drozdowicz, 2011; Chachuła et al., 2021; Drozdowicz et al., 2012; Drozdowicz et al., 2007; Jarocki, 1924; Ławrynowicz et al., 2011; Miśkiewicz, 2001; Panek and Romański, 2010; Pawłowicz et al., 2025; Salamaga, 2021; Stojanowska 1983; Stojanowska, 1980a; Stojanowska, 2004a; Stojanowska, 2004b; Stojanowska and Panek, 2002; Stojanowska and Panek, 2004; Ślusarczyk, 2021; Drozdowicz, 1992, 1997b, 2001, 2003a; Jarocki, 1924; Krzemieniewska, 1947, 1957, 1960b; Miśkiewicz, 2001; Stojanowska, 1970, 1972, 1980b, 1983, 1984, 1998; Stojanowska and Panek, 2002 |
| *Heterotrichia ferruginea* (Saut.) Yatsiuk, Leontyev & Schnittler, 2024 | Bochynek and Drozdowicz, 2011; Chachuła et al., 2021; Drozdowicz, 1997; Drozdowicz et al., 2012; Jarocki, 1924; Kalinowska-Kucharska, 1975; Krupa, 1886; Krupa, 1887; Panek and Romański, 2010; Stojanowska 1983; Stojanowska, 1977a; Stojanowska, 1980a; Stojanowska, 2004a; Stojanowska and Panek, 2005; Wrońska, 1974; Drozdowicz, 1992; Drozdowicz, 1997a; Krawiec, 1965; Krzemieniewska, 1957; Krzemieniewska, 1960b; Rostafiński, 1875; Stojanowska, 1972; Stojanowska, 1977b; Stojanowska, 1983; Stojanowska, 1984; Stojanowska, 1992; Drozdowicz, 2005; Krzysztofiak et al., 2010; Ławrynowicz et al., 2011; Stojanowska, 2004; Stojanowska and Panek, 2002; Stojanowska and Panek, 2004; Paul et al., 2023 |
| *Heterotrichia insignis* (Kalchbr. & Cooke) Yatsiuk, Leontyev & Schnittler, 2024 | Panek and Romański, 2010; Stojanowska 1983; Krzemieniewska, 1947; Krzemieniewska, 1957; Krzemieniewska, 1960b; Stojanowska, 1972 |
| *Heterotrichia obvelata* (Oeder) Yatsiuk, Leontyev & Schnittler, 2024 | Błoński and Drymmer, 1889; Bochynek & Drozdowicz, 2012; Bochynek and Drozdowicz, 2011; Drozdowicz, 2005; Drozdowicz et al., 2012; Drozdowicz et al., 2007; Gutwiński, 1901; Jarocki, 1924; Kalinowska-Kucharska, 1975; Krupa, 1886; Ławrynowicz et al., 2011; Panek and Romański, 2010; Paul et al., 2023; Salamaga, 2021; Stojanowska 1983; Stojanowska, 1977a; Stojanowska, 1977b; Stojanowska, 1980a; Stojanowska, 2004a; Stojanowska and Panek, 2002; Stojanowska and Panek, 2005; Stojanowska and Panek, 2004; Ślusarczyk, 2010; Wrońska, 1974; Drozdowicz, 1992; Komorowska and Drozdowicz, 1996; Krawiec, 1965; Krzemieniewska, 1933; Krzemieniewska, 1947; Krzemieniewska, 1957; Krzemieniewska, 1960b; Krzemieniewska and Badura, 1954; Michalski, 1951; Namysłowski, 1914; Rouppert, 1909b; Stecki, 1910; Stojanowska, 1970; Stojanowska, 1980b; Stojanowska, 1983; Stojanowska, 1984; Stojanowska, 1992; Stojanowska, 2000a |
| *Heterotrichia oerstedii* (Rostaf.) Yatsiuk, Leontyev & Schnittler, 2024 | Chachuła et al., 2021; Drozdowicz, 1997; Jarocki, 1924; Krzysztofiak et al., 2010; Panek and Romański, 2010; Paul et al., 2023; Salamaga, 2021; Stojanowska 1983; Stojanowska, 1977a; Stojanowska, 1977b; Stojanowska and Panek, 2002; Stojanowska and Panek, 2004; Drozdowicz, 1992; Drozdowicz, 1997a; Jarocki, 1924; Krzemieniewska, 1957; Krzemieniewska, 1960b; Stojanowska, 1972; Stojanowska, 1977b; Stojanowska, 1983; Stojanowska, 1984; Stojanowska, 1992; Stojanowska, 2000a; Stojanowska and Panek, 2002 |
| *Heterotrichia pomiformis* (Leers) Yatsiuk, Leontyev & Schnittler, 2024 | Bochynek & Drozdowicz, 2012; Bochynek and Drozdowicz, 2011; Drozdowicz et al., 2012; Drozdowicz et al., 2007; Krzysztofiak et al., 2010; Ławrynowicz et al., 2011; Panek and Romański, 2010; Paul et al., 2023; Pawłowicz et al., 2025; Salamaga, 2021; Stojanowska 1983; Stojanowska, 1977a; Stojanowska, 1977b; Stojanowska, 1980a; Stojanowska, 2004b; Stojanowska and Panek, 2002; Stojanowska and Panek, 2005; Stojanowska and Panek, 2004; Wrońska, 1974; Drozdowicz, 1992; Krzemieniewska, 1933; Krzemieniewska, 1947; Krzemieniewska, 1957; Stojanowska, 1972; Stojanowska, 1977b; Stojanowska, 1980b; Stojanowska, 1983; Stojanowska, 1984; Stojanowska, 1992; Stojanowska and Panek, 2002; Wrońska, 1974 |
| *Lamproderma aeneum* Mar. Mey. & Poulain, 2002 | Ronikier et al., 2008 |
| *Lamproderma arcyrioides* (Sommerf.) Rostaf., 1874 | Jarocki, 1924; Paul et al., 2023; Stojanowska 1983; Stojanowska, 1980a; Stojanowska and Panek, 2002; Stojanowska and Panek, 2005; Stojanowska and Panek, 2004; Jarocki, 1924; Wrońska, 1974 |
| *Lamproderma argenteobrunneum* A. Ronikier, Lado & Mar. Mey., 2010 | Paul et al., 2023 |
| *Lamproderma columbinum* (Pers.) Rostaf., 1873 | Bochynek and Drozdowicz, 2011; Drozdowicz et al., 2012; Jarocki, 1924; Ławrynowicz et al., 2011; Panek and Romański, 2010; Paul et al., 2023; Stojanowska 1983; Stojanowska, 2004; Stojanowska and Panek, 2005; Stojanowska and Panek, 2004; Ślusarczyk, 2010; Drozdowicz, 1992; Jarocki, 1924; Komorowska and Drozdowicz, 1996; Krzemieniewska, 1947, 1960b; Stojanowska, 1972, 1983, 1984 |
| *Lamproderma echinosporum* Meyl., 1924 | Bochynek & Drozdowicz, 2012 |
| *Lamproderma echinulatum* (Berk.) Rostaf., 1876 | Paul et al., 2023 |
| *Lamproderma gulielmae* Meyl., 1919 | Drozdowicz et al., 2003; Krzemieniewska, 1933, 1934, 1960b |
| *Lamproderma maculatum* Kowalski, 1970 | Bochynek & Drozdowicz, 2012; Stojanowska, 2004; Drozdowicz, 2001 |
| *Lamproderma ovoideoechinulatum* Mar. Mey. & Poulain, 2005 | Ronikier et al., 2008 |
| *Lamproderma ovoideum* Meyl., 1932 | Bochynek & Drozdowicz, 2012; Ronikier et al., 2008; Drozdowicz, 2001 |
| *Lamproderma pulchellum* Meyl., 1932 | Krupa, 1887 |
| *Lamproderma pulveratum* Mar. Mey. & Poulain, 1991 | Ronikier et al., 2008 |
| *Lamproderma sauteri* Rostaf., 1874 | Paul et al., 2023; Stojanowska, 2004; Drozdowicz, 1988 |
| *Lamproderma scintillans* (Berk. & Broome) Morgan, 1894 | Panek and Romański, 2010; Stojanowska, 2004 |
| *Lamproderma spinulosporum* Mar. Mey., Nowotny & Poulain, 1994 | Janik and Ronikier, 2016 |
| *Lamproderma zonatum* Mar. Mey. & Poulain, 2004 | Stojanowska, 2004 |
| *Leocarpus fragilis* (Dicks.) Rostaf., 1874 | Błoński, 1890; Błoński and Drymmer, 1889; Bochynek & Drozdowicz, 2012; Bochynek and Drozdowicz, 2011; Drozdowicz, 1997; Jarocki, 1924; Kalinowska-Kucharska, 1975; Krupa, 1887; Krzysztofiak et al., 2010; Ławrynowicz et al., 2011; Panek and Romański, 2010; Paul et al., 2023; Salamaga, 2021; Stojanowska 1983; Stojanowska, 1977a; Stojanowska, 1980a; Stojanowska, 2004a; Stojanowska, 2004b; Ślusarczyk, 2010; Wilga and Ciechanowski, 2007; Wrońska, 1974; Błoński, 1890; Drozdowicz, 1997a; Drozdowicz and Wilga, 2002; Jarocki, 1924; Kalinowska-Kucharska, 1975; Krzemieniewska, 1933, 1957, 1960b; Michalski, 1951; Namysłowski, 1914; Nitardy, 1904; Rouppert, 1912; Steinecke, 1918; Stojanowska, 1972, 1977b, 1980b, 1983, 1984; Wrońska, 1974; Zweigbaumówna, 1924 |
| *Licea biforis* Morgan, 1893 | Krzemieniewska, 1960b; Ronikier et al., 2017 |
| *Licea castanea* G. Lister, 1911 | Krzemieniewska, 1960b; Ronikier et al., 2017 |
| *Licea clarkii* Ing, 1982 | Ronikier et al., 2017 |
| *Licea kleistobolus* G.W. Martin, 1942 | Jarocki, 1927; Jarocki, 1931; Ronikier et al., 2017; Paul et al., 2023 |
| *Licea minima* Fr., 1829 | Jarocki, 1931; Stojanowska 1983; Stojanowska, 2004a; Stojanowska and Panek, 2004; Krzemieniewska, 1933, 1947, 1960b; Bochynek and Drozdowicz, 2011; Bochynek & Drozdowicz, 2012; Drozdowicz et al., 2007; Drozdowicz et al., 2012; Salamaga et al., 2016; Ronikier et al., 2017; Salamaga, 2021; Paul et al., 2023 |
| *Licea parasitica* (Zukal) G. W. Martin, 1942 | Fałtynowicz, 2003; Jando and Kukwa, 2003; Ronikier et al., 2017 |
| *Licea pedicellata* (H.C. Gilbert) H.C. Gilbert, 1942 | Ronikier et al., 2017 |
| *Licea poculiformis* Ukkola, 1998 | Ronikier et al., 2017 |
| *Licea pusilla* Schrad., 1797 | Rostafiński, 1874; Jarocki, 1931; Krzemieniewska, 1947, 1960b; Stojanowska 1983; Stojanowska and Panek, 2004; Drozdowicz, 1992; Ronikier et al., 2017; Salamaga, 2021; Paul et al., 2023 |
| *Licea pygmaea* (Meyl.) Ing, 1982 | Ronikier et al., 2017 |
| *Lignydium muscorum* (Alb. & Schwein.) Kuntze, 1898 | Jarocki, 1924; Panek and Romański, 2010; Paul et al., 2023; Stojanowska, 2004a; Stojanowska and Panek, 2002 |
| *Lycogala confusum* Nann. -Bremek. ex Ing, 1999 | Krzemieniewska, 1947, 1960b; Krzemieniewska and Badura, 1954; Stojanowska, 1972; Drozdowicz, 2005; Drozdowicz et al., 2007; Bochynek & Drozdowicz, 2012; Salamaga, 2021 |
| *Lycogala conicum* Pers., 1801 | Jarocki, 1924; Krzemieniewska, 1947, 1957, 1960b; Stojanowska 1983; Miśkiewicz, 2001; Stojanowska and Panek, 2002; Stojanowska, 1972, 1983, 1984, 2000a; Wilga and Ciechanowski, 2007; Panek and Romański, 2010; Ławrynowicz et al., 2011; Bochynek and Drozdowicz, 2011; Drozdowicz et al., 2007; Drozdowicz et al., 2012; Salamaga, 2021; Ślusarczyk, 2021 |
| *Lycogala epidendrum* (L.) Fr., 1829 | Błoński et al., 1888; Błoński and Drymmer, 1889; Błoński, 1890; Gutwiński, 1901; Jarocki, 1924; Krupa, 1886, 1887, 1889; Kalinowska-Kucharska, 1975; Miśkiewicz, 2001; Drozdowicz, 2005; Ławrynowicz et al., 2011; Wilga and Ciechanowski, 2007; Wrońska, 1974; Bujakiewicz and Fiebich, 1992; Bujakiewicz, 1999; Friedrich, 1994; Krawiec, 1965; Michalski, 1951; Namysłowski, 1910; Nitardy, 1904; Rouppert, 1909a, 1909b, 1911, 1912; Steinecke, 1918; Zweigbaumówna, 1924; Krzemieniewska, 1933, 1947, 1957; Krzemieniewska and Badura, 1954; Komorowska and Drozdowicz, 1996; Drozdowicz, 1992, 1997b, 2001, 2003a; Drozdowicz and Wilga, 2002; Stojanowska, 1970, 1972, 1977b, 1980b, 1981, 1983, 1984, 1992, 2000a; Bochynek and Drozdowicz, 2011; Bochynek & Drozdowicz, 2012; Chachuła et al., 2021; Drozdowicz et al., 2007; Drozdowicz et al., 2012; Panek and Romański, 2010; Salamaga et al., 2016; Salamaga, 2021; Paul et al., 2023; Ślusarczyk, 2010, 2021 |
| *Lycogala exiguum* Morgan, 1893 | Drozdowicz, 1992; Krzemieniewska, 1947, 1957, 1960b; Stojanowska 1983; Stojanowska, 1977a, 1983, 1984, 2000a; Wrońska, 1974; Stojanowska and Panek, 2002; Drozdowicz et al., 2007; Ławrynowicz et al., 2011; Panek and Romański, 2010; Chachuła et al., 2021; Stojanowska, 2004a, 2004b |
| *Lycogala flavofuscum* (Ehrenb.) Rostaf., 1873 | Rostafiński, 1875; Gutwiński, 1901; Jarocki, 1924; Skupieński, 1934; Krzemieniewska, 1957, 1960b; Wrońska, 1974; Stojanowska, 1972, 1977b, 1992; Drozdowicz, 1992; Stojanowska 1983; Ławrynowicz et al., 2011; Chachuła et al., 2021 |
| *Macbrideola cornea* (G. Lister & Cran) Alexop., 1967 | Drozdowicz et al., 2007 |
| *Meriderma carestiae* (Ces. & De Not.) Mar. Mey. & Poulain, 2011 | Komorowska and Drozdowicz, 1996; Drozdowicz, 1997b; Janik and Ronikier, 2016; Panek and Romański, 2010; Ronikier et al., 2008; Paul et al., 2023 |
| *Meriderma cribrarioides* (Fr.) Mar. Mey. & Poulain, 2011 | Bochynek & Drozdowicz, 2012; Janik and Ronikier, 2016; Ronikier et al., 2008 |
| *Meriderma echinulatum* (Meyl.) Mar. Mey. & Poulain, 2011 | Janik and Ronikier, 2016 |
| *Meriderma fuscatum* (Meyl.) Mar. Mey. & Poulain, 2011 | Janik and Ronikier, 2016 |
| *Meriderma spinulosporum* ad int. | Janik and Ronikier, 2016 |
| *Metatrichia floriformis* (Schwein.) Nann.-Bremek., 1985 | Hazslinszky, 1884; Jarocki, 1924; Kalinowska-Kucharska, 1975; Krzemieniewska, 1947, 1957, 1960b; Wrońska, 1974; Stojanowska 1983; Stojanowska, 1972, 1977a, 1977b; Drozdowicz, 1992, 2005; Ławrynowicz et al., 2011; Panek and Romański, 2010; Drozdowicz et al., 2007; Drozdowicz et al., 2012; Bochynek and Drozdowicz, 2011; Bochynek & Drozdowicz, 2012; Chachuła et al., 2021; Paul et al., 2023; Stojanowska and Panek, 2004, 2005; Stojanowska, 2004a, 2004b |
| *Metatrichia vesparia* (Batsch) Nann. -Bremek. ex G.W. Martin & Alexop., 1969 | Błoński and Drymmer, 1889; Krupa, 1886, 1887, 1889; Jarocki, 1924; Kalinowska-Kucharska, 1975; Krawiec, 1965; Wrońska, 1974; Miśkiewicz, 2001; Krzemieniewska, 1933, 1947, 1957; Krzemieniewska and Badura, 1954; Drozdowicz, 1992, 1997b, 2001, 2003a; Stojanowska, 1970, 1972, 1977a, 1977b, 1980, 1980b, 1983, 1984, 1992, 2000a; Drozdowicz et al., 2007; Drozdowicz et al., 2012; Bochynek and Drozdowicz, 2011; Ławrynowicz et al., 2011; Panek and Romański, 2010; Wilga and Ciechanowski, 2007; Stojanowska 1983; Drozdowicz, 2005; Chachuła et al., 2021; Paul et al., 2023; Pawłowicz et al., 2025; Salamaga, 2021; Stojanowska and Panek, 2002, 2004, 2005; Ślusarczyk, 2010, 2021 |
| *Nannengaella alpestris* (Mitchel, S.W. Chapm. & M.L. Farr) J.M. García-Martín, J.C. Zamora & Lado, 2023 | Ronikier et al., 2008 |
| *Nannengaella alpina* (Lister & G. Lister) J.M. García-Martín, J.C. Zamora & Lado, 2023 | Stojanowska, 2004a |
| *Nannengaella contexta* (Pers.) J.M. García-Martín, J.C. Zamora & Lado, 2023 | Rostafiński, 1874; Jarocki, 1924; Krzemieniewska, 1960b; Stojanowska 1983; Stojanowska, 2004b; Drozdowicz et al., 2012; Panek and Romański, 2010 |
| *Nannengaella globulifera* (Bull.) J.M. García-Martín, J.C. Zamora & Lado, 2023 | Rostafiński, 1874; Jarocki, 1924; Kalinowska-Kucharska, 1975; Krzemieniewska, 1933, 1947, 1957, 1960b; Krzemieniewska and Badura, 1954; Wrońska, 1974; Stojanowska, 1970, 1972, 1977b, 1983, 1984, 2000a; Stojanowska 1983; Drozdowicz, 1992; Drozdowicz et al., 2007; Ławrynowicz et al., 2011; Panek and Romański, 2010; Salamaga, 2021; Drozdowicz et al., 2012 |
| *Nannengaella leucopus* (Link) J.M. García-Martín, J.C. Zamora & Lado, 2023 | Rostafiński, 1874; Jarocki, 1924; Krzemieniewska, 1957, 1960b; Stojanowska, 1972, 1980, 1980b, 1983; Wrońska, 1974; Stojanowska 1983; Drozdowicz et al., 2007; Salamaga et al., 2016; Stojanowska and Panek, 2005; Stojanowska and Panek, 2004 |
| *Nannengaella mellea* (Berk. & Broome) J.M. García-Martín, J.C. Zamora & Lado, 2023 | Paul et al., 2023 |
| *Nannengaella sulphurea* (Alb. & Schwein.) J.M. García-Martín, J.C. Zamora & Lado, 2023 | Jarocki, 1924 |
| *Neodiderma spumarioides* (Fr. & Palmquist) X.F. Li, B. Zhang & Yu Li, 2024 | Rostafiński, 1874; Jarocki, 1924; Krzemieniewska, 1947, 1957, 1960b; Stojanowska, 1972, 1983; Ławrynowicz et al., 2011; Panek and Romański, 2010; Stojanowska 1983; Stojanowska and Panek, 2004 |
| *Oligonema affine* (de Bary) García-Cunch., J.C. Zamora & Lado, 2022 | Rostafiński, 1875; Jarocki, 1924; Krupa, 1889; Krzemieniewska, 1957, 1960b; Krzemieniewska and Badura, 1954; Krawiec, 1965; Stojanowska, 1970, 1972, 1980b, 1983; Wrońska, 1974; Drozdowicz, 2003a; Ławrynowicz et al., 2011; Panek and Romański, 2010; Stojanowska 1983; Stojanowska and Panek, 2002; Stojanowska, 2004b; Salamaga, 2021; Paul et al., 2023 |
| *Oligonema favogineum* (Batsch) García-Cunch., J.C. Zamora & Lado, 2022 | Rostafiński, 1874; Jarocki, 1924; Kalinowska-Kucharska, 1975; Krawiec, 1965; Krzemieniewska, 1933, 1947, 1957; Krzemieniewska and Badura, 1954; Wrońska, 1974; Miśkiewicz, 2001; Stojanowska, 1970, 1972, 1977b, 1983, 1984, 2000a; Drozdowicz, 1992, 1997b, 2001, 2003a; Ławrynowicz et al., 2011; Wilga and Ciechanowski, 2007; Bochynek and Drozdowicz, 2011; Drozdowicz et al., 2007; Drozdowicz et al., 2012; Bochynek & Drozdowicz, 2012; Chachuła et al., 2021; Panek and Romański, 2010; Salamaga et al., 2016; Salamaga, 2021; Stojanowska 1983; Stojanowska and Panek, 2002; Stojanowska, 2004a, 2004b; Ślusarczyk, 2010, 2021 |
| *Oligonema flavidum* (Peck) Peck, 1878 | Salamaga, 2013; Salamaga, 2021 |
| *Oligonema persimile* (P. Karst.) García-Cunch., J.C. Zamora & Lado, 2022 | Rostafiński, 1874; Jarocki, 1924; Kalinowska-Kucharska, 1975; Krawiec, 1965; Krzemieniewska, 1933, 1957, 1960b; Krzemieniewska and Badura, 1954; Wrońska, 1974; Stojanowska, 1970, 1972, 1977b, 1980b, 1983, 1984, 2000a; Drozdowicz, 2003a, 2005; Stojanowska 1983; Drozdowicz et al., 2007; Drozdowicz et al., 2012; Ławrynowicz et al., 2011; Panek and Romański, 2010; Salamaga, 2021 |
| *Oligonema schweinitzii* (Berk.) G.W. Martin, 1947 | Stojanowska, 1970; Stojanowska and Panek, 2004; Salamaga, 2021 |
| *Oligonema verrucosum* (Berk.) García-Cunch., J.C. Zamora & Lado, 2022 | Tabacki, 1977; Magiera and Drozdowicz, 2004 |
| *Ophiotheca chrysosperma* Curr., 1854 | Krupa, 1887; Krzemieniewska, 1957; Krzemieniewska, 1960b; Stojanowska 1983; Stojanowska and Panek, 2004 |
| *Perichaena corticalis* (Batsch) Rostaf., 1875 | Krupa, 1889; Eichler, 1907; Migula, 1910; Jarocki, 1924; Krzemieniewska, 1929; Krzemieniewska, 1933; Krzemieniewska, 1947; Krzemieniewska, 1957; Krzemieniewska, 1960b; Stojanowska, 1972; Kalinowska-Kucharska, 1975; Stojanowska, 1977; Stojanowska, 1977b; Stojanowska, 1980a; Stojanowska, 1980b; Stojanowska 1983; Stojanowska, 1983; Stojanowska, 1992; Stojanowska and Panek, 2002; Stojanowska and Panek, 2004; Stojanowska, 2004b; Drozdowicz et al., 2007; Panek and Romański, 2010; Ławrynowicz et al., 2011; Paul et al., 2023 |
| *Perichaena depressa* Lib., 1837 | Krupa, 1887; Krzemieniewska, 1957; Stojanowska, 1972; Stojanowska, 1977; Stojanowska, 1977b; Stojanowska, 1980a; Stojanowska, 1980b; Stojanowska 1983; Stojanowska, 1983; Drozdowicz, 1992; Stojanowska, 1992; Drozdowicz, 1997; Drozdowicz, 1997a; Stojanowska and Panek, 2002; Stojanowska and Panek, 2004; Stojanowska, 2004; Bochynek and Drozdowicz, 2011; Pawłowicz et al., 2025 |
| *Perichaena liceoides* Rostaf., 1875 | Krzemieniewska, 1929 |
| *Physarum album* (Bull.) Chevall., 1826 | Jarocki, 1924; Krzemieniewska, 1933; Krzemieniewska, 1947; Krzemieniewska and Badura, 1954; Krzemieniewska, 1957; Krawiec, 1965; Stojanowska, 1970; Stojanowska, 1972; Wrońska, 1974; Kalinowska-Kucharska, 1975; Stojanowska, 1977; Stojanowska, 1977b; Stojanowska, 1980a; Stojanowska, 1980b; Stojanowska 1983; Stojanowska, 1983; Stojanowska, 1984; Drozdowicz, 1992; Stojanowska, 2000a; Drozdowicz, 2001; Miśkiewicz, 2001; Stojanowska and Panek, 2002; Stojanowska, 2004; Stojanowska and Panek, 2004; Drozdowicz et al., 2007; Wilga and Ciechanowski, 2007; Ślusarczyk, 2010; Panek and Romański, 2010; Ławrynowicz et al., 2011; Drozdowicz et al., 2012; Salamaga, 2021; Paul et al., 2023; Pawłowicz et al., 2025 |
| *Physarum alexandrowiczii* de Bary & Rostaf., 1872 | Schroeter, 1889 |
| *Physarum auriscalpium* Cooke, 1877 | Krzemieniewska, 1933; Stojanowska, 1977; Stojanowska, 1977b; Drozdowicz, 1992 |
| *Physarum cinereum* (Batsch) Pers., 1794 | Krupa, 1886; Krupa, 1887; Jarocki, 1924; Krzemieniewska, 1929; Krzemieniewska, 1933; Krzemieniewska, 1960b; Stojanowska, 1972; Kalinowska-Kucharska, 1975; Stojanowska, 1977; Stojanowska, 1977b; Stojanowska, 1980a; Stojanowska, 1980b; Stojanowska, 1981; Stojanowska 1983; Stojanowska, 1983; Stojanowska, 1984; Drozdowicz, 1992; Stojanowska, 1992; Drozdowicz, 1997; Drozdowicz, 1997a; Stojanowska, 2000a; Stojanowska and Panek, 2002; Stojanowska, 2004; Stojanowska and Panek, 2004; Wilga and Ciechanowski, 2007; Krzysztofiak et al., 2010; Panek and Romański, 2010; Ślusarczyk, 2010; Bochynek and Drozdowicz, 2011; Ławrynowicz et al., 2011; Drozdowicz et al., 2012 |
| *Physarum citrinum* Schumach., 1803 | Krupa, 1887; Krzemieniewska, 1933; Krzemieniewska, 1947; Krzemieniewska, 1957; Krzemieniewska, 1960b; Stojanowska, 1972; Kalinowska-Kucharska, 1975; Stojanowska, 1977; Stojanowska, 1977b; Stojanowska 1983; Stojanowska, 1983; Stojanowska, 1984; Drozdowicz, 1992; Stojanowska, 2000a; Stojanowska, 2004; Stojanowska and Panek, 2004; Panek and Romański, 2010; Ławrynowicz et al., 2011 |
| *Physarum compressum* Alb. & Schwein., 1805 | Rostafiński, 1874; Gutwiński, 1901; Jarocki, 1924; Krzemieniewska, 1957; Krzemieniewska, 1960b; Stojanowska 1983; Drozdowicz, 1992; Stojanowska, 1992; Stojanowska, 2000a; Stojanowska, 2004; Stojanowska and Panek, 2004; Panek and Romański, 2010; Ławrynowicz et al., 2011; Drozdowicz et al., 2012 |
| *Physarum conglomeratum* (Fr.) Rostaf., 1874 | Schroeter, 1889; Szulczewski, 1951; Panek and Romański, 2010 |
| *Physarum decipiens* M.A. Curtis, 1848 | Stojanowska, 2004; Kuhnt, 2019 |
| *Physarum diderma* Rostaf., 1874 | Rostafiński, 1874; Krzemieniewska, 1957; Krzemieniewska, 1960b; Drozdowicz, 1992; Drozdowicz et al., 2003 |
| *Physarum flavicomum* Berk., 1845 | Krupa, 1887; Drozdowicz, 1992; Stojanowska and Panek, 2002; Stojanowska and Panek, 2004; Salamaga, 2021 |
| *Physarum leucophaeum* Fr. & Palmquist, 1818 | Krupa, 1886; Krupa, 1887; Błoński and Drymmer, 1889; Jarocki, 1924; Krzemieniewska and Badura, 1954; Krzemieniewska, 1957; Krzemieniewska, 1960b; Stojanowska, 1970; Stojanowska, 1972; Wrońska, 1974; Stojanowska, 1977; Stojanowska, 1977b; Stojanowska, 1980a; Stojanowska, 1980b; Stojanowska 1983; Stojanowska, 1983; Stojanowska, 1984; Drozdowicz, 1992; Stojanowska, 1992; Stojanowska and Panek, 2002; Stojanowska, 2004; Stojanowska and Panek, 2004; Stojanowska and Panek, 2005; Drozdowicz et al., 2007; Panek and Romański, 2010; Ślusarczyk, 2010; Ławrynowicz et al., 2011; Drozdowicz et al., 2012; Paul et al., 2023 |
| *Physarum licheniforme* (Schwein.) Lado, 2001 | Rostafiński, 1874; Eichler, 1904; Krzemieniewska, 1957; Krzemieniewska, 1960b; Drozdowicz, 2005; Drozdowicz et al., 2007; Paul et al., 2023 |
| *Physarum murinum* Lister, 1894 | Panek and Romański, 2010 |
| *Physarum nitens* (Lister) Ing, 1982 | Panek and Romański, 2010 |
| *Physarum notabile* T. Macbr., 1922 | Jarocki, 1924; Krzemieniewska, 1957; Krzemieniewska, 1960b; Stojanowska, 1972; Stojanowska 1983; Drozdowicz, 1992; Drozdowicz et al., 2007; Panek and Romański, 2010; Ślusarczyk, 2010; Ławrynowicz et al., 2011 |
| *Physarum nucleatum* Rex, 1891 | Raciborski, 1884b; Drozdowicz et al., 2007 |
| *Physarum penetrale* Rex, 1891 | Krzemieniewska, 1947; Krzemieniewska, 1960b; Stojanowska 1983; Stojanowska and Panek, 2004; Drozdowicz et al., 2012 |
| *Physarum polonicum* Skup., 1924 | Skupieński, 1924; Drozdowicz et al., 2003 |
| *Physarum psittacinum* Ditmar, 1817 | Rostafiński, 1874; Krzemieniewska, 1947; Krzemieniewska, 1957; Krzemieniewska, 1960b; Stojanowska, 1972; Wrońska, 1974; Kalinowska-Kucharska, 1975; Stojanowska, 1977; Stojanowska, 1977b; Stojanowska, 1980a; Stojanowska, 1980b; Stojanowska 1983; Stojanowska, 1983; Stojanowska, 1984; Drozdowicz, 1992; Stojanowska, 2004; Stojanowska and Panek, 2004; Panek and Romański, 2010; Bochynek and Drozdowicz, 2011; Ławrynowicz et al., 2011 |
| *Physarum pusillum* (Berk. & M.A. Curtis) G. Lister, 1911 | Stojanowska, 1972; Stojanowska, 1980a; Stojanowska, 1980b; Stojanowska 1983; Stojanowska and Panek, 2002 |
| *Physarum robustum* (Lister) Nann. -Bremek., 1973 | Krzemieniewska, 1957; Krzemieniewska, 1960b; Kalinowska-Kucharska, 1975; Stojanowska, 2004; Drozdowicz et al., 2007; Panek and Romański, 2010; Ławrynowicz et al., 2011; Drozdowicz et al., 2012; Salamaga, 2021 |
| *Physarum rubiginosum* Fr. & Palmquist, 1818 | Rostafiński, 1874; Stojanowska, 2000b; Drozdowicz et al., 2003 |
| *Physarum schroeteri* Rostaf., 1875 | Stojanowska, 1972; Stojanowska 1983 |
| *Physarum sessile* Brândza, 1921 | Firich, 1962; Stojanowska 1983; Stojanowska and Panek, 2004; Drozdowicz et al., 2007 |
| *Physarum vernum* Sommerf., 1829 | Ronikier et al., 2008 |
| *Physarum virescens* Ditmar, 1817 | Rostafiński, 1874; Jarocki, 1924; Krzemieniewska, 1947; Krzemieniewska and Badura, 1954; Krzemieniewska, 1957; Krzemieniewska, 1960b; Stojanowska, 1972; Kalinowska-Kucharska, 1975; Stojanowska 1983; Stojanowska, 1983; Stojanowska, 1984; Stojanowska, 2004; Stojanowska and Panek, 2004; Panek and Romański, 2010; Ślusarczyk, 2010; Ławrynowicz et al., 2011; Salamaga et al., 2016 |
| *Physarum viride* (Bull.) Pers., 1795 | Jarocki, 1924; Krzemieniewska, 1933; Krzemieniewska, 1947; Krzemieniewska, 1957; Krzemieniewska, 1960b; Stojanowska, 1972; Wrońska, 1974; Kalinowska-Kucharska, 1975; Stojanowska, 1977; Stojanowska, 1977b; Stojanowska, 1980a; Stojanowska, 1980b; Stojanowska 1983; Stojanowska, 1983; Stojanowska, 1984; Drozdowicz, 1992; Stojanowska, 2000a; Stojanowska and Panek, 2002; Stojanowska, 2004; Stojanowska and Panek, 2004; Stojanowska and Panek, 2005; Drozdowicz et al., 2007; Panek and Romański, 2010; Ławrynowicz et al., 2011; Drozdowicz et al., 2012; Salamaga et al., 2016; Salamaga, 2021; Paul et al., 2023 |
| *Polyschismium aggregatum* (Kowalski) Prikhodko, Shchepin, Novozh., G. Moreno, López-Vill. & Schnittler, 2023 | Stojanowska, 2004; Ronikier et al., 2008 |
| *Polyschismium carestianum* (Rabenh.) A. Ronikier, J.M. García-Martín, A. Kuhnt, J.C. Zamora, M. de Haan, Janik & Lado, 2022 | Stojanowska, 2004; Bochynek & Drozdowicz, 2012; Paul et al., 2023 |
| *Polyschismium chailletii* (Rostaf.) A. Ronikier, J.M. García-Martín, A. Kuhnt, J.C. Zamora, M. de Haan, Janik & Lado, 2022 | Ronikier et al., 2008 |
| *Polyschismium fallax* (Rostaf.) A. Ronikier, J.M. García-Martín, A. Kuhnt, J.C. Zamora, M. de Haan, Janik & Lado, 2022 | Paul et al., 2023 |
| *Polyschismium neoperforatum* (A. Kuhnt) A. Ronikier, A. Kuhnt, M. de Haan & Janik, 2022 | Ronikier et al., 2022 |
| *Polyschismium trevelyanii* (Grev.) Corda ex Rostaf., 1874 | Krzemieniewska, 1960b; Drozdowicz et al., 2003 |
| *Reticularia liceoides* (Lister) Nann. -Bremek., 1973 | Chachuła et al., 2021; Krzemieniewska, 1933, 1934, 1960b |
| *Reticularia lobata* Lister, 1894 | Stojanowska 1983; Stojanowska and Panek, 2004; Stojanowska, 1983 |
| *Reticularia lycoperdon* Bull., 1790 | Krupa, 1889; Nitardy, 1904; Steinecke, 1918; Namysłowski, 1910, 1914; Jarocki, 1924; Krzemieniewska, 1933, 1957; Kalinowska-Kucharska, 1975; Stojanowska, 1970, 1972, 1977b, 1980b, 1983, 1984, 1992; Drozdowicz, 1992, 1997b, 2001, 2003a, 2003b; Drozdowicz and Wilga, 2002; Bujakiewicz, 1999; Bochynek and Drozdowicz, 2011; Ławrynowicz et al., 2011; Panek and Romański, 2010; Stojanowska 1983; Stojanowska and Panek, 2002; Stojanowska and Panek, 2004; Drozdowicz, 2005; Drozdowicz et al., 2012; Chachuła et al., 2021; Chachuła et al., 2021; Ławrynowicz et al., 2011; Panek and Romański, 2010; Paul et al., 2023; Salamaga et al., 2016; Stojanowska, 2004; Ślusarczyk, 2010 |
| *Reticularia splendens* Morgan, 1893 | Steinecke, 1918; Jarocki, 1924; Krzemieniewska, 1933, 1934, 1957, 1960b; Stojanowska, 1977b, 1983, 1984; Drozdowicz, 1992; Stojanowska 1983; Stojanowska, 2004a |
| *Stemonaria irregularis* (Rex) Nann. -Bremek., R. Sharma & Y. Yamam., 1984 | Jarocki, 1924; Krzemieniewska, 1957, 1960b; Stojanowska, 1972; Stojanowska 1983; Drozdowicz et al., 2007 |
| *Stemonaria longa* (Peck) Nann. -Bremek., R. Sharma & Y. Yamam., 1984 | Krzemieniewska, 1957, 1960b; Drozdowicz, 2017 |
| *Stemonitis axifera* (Bull.) T. Macbr., 1899 | Błoński, 1890; Błoński and Drymmer, 1889; Błoński et al., 1888; Krupa, 1886, 1888, 1889; Jarocki, 1924; Krawiec, 1965; Krzemieniewska, 1933, 1947, 1957; Michalski, 1951; Krzemieniewska and Badura, 1954; Kalinowska-Kucharska, 1975; Miśkiewicz, 2001; Wrońska, 1974; Drozdowicz, 1992, 2001, 2003a; Drozdowicz and Wilga, 2002; Stojanowska, 1972, 1977b, 1980b, 1981, 1983, 1984, 1992, 2000a; Ławrynowicz et al., 2011; Stojanowska 1983; Stojanowska and Panek, 2002; Bochynek and Drozdowicz, 2011, 2012; Panek and Romański, 2010; Drozdowicz et al., 2007, 2012; Ślusarczyk, 2010; Chachuła et al., 2021; Salamaga et al., 2016; Salamaga, 2021; Paul et al., 2023 |
| *Stemonitis flavogenita* E. Jahn, 1904 | Rostafiński, 1875; Jarocki, 1924; Krzemieniewska, 1933, 1947, 1957, 1960b; Kalinowska-Kucharska, 1975; Drozdowicz, 1992; Stojanowska, 1972, 1977b, 1980b, 1983, 1992; Błoński and Drymmer, 1889; Błoński et al., 1888; Błoński, 1890; Stojanowska and Panek, 2002; Ławrynowicz et al., 2011; Stojanowska 1983; Panek and Romański, 2010; Bochynek and Drozdowicz, 2011; Stojanowska, 2004a, 2004b |
| *Stemonitis fusca* Roth, 1787 | Błoński, 1888, 1890; Krupa, 1886, 1887; Kwieciński, 1896; Rouppert, 1909a, 1909b; Gutwiński, 1901; Stecki, 1910; Jarocki, 1924, 1931; Krzemieniewska, 1929, 1933, 1947, 1957, 1960b; Michalski, 1951; Krzemieniewska and Badura, 1954; Krawiec, 1965; Kalinowska-Kucharska, 1975; Namysłowski, 1910; Miśkiewicz, 2001; Wrońska, 1974; Bresadola, 1903; Drozdowicz, 1992, 2003a; Bochynek and Drozdowicz, 2011, 2012; Stojanowska, 1970, 1972, 1977a, 1977b, 1980, 1980b, 1981, 1983, 1984, 1992, 2000a; Stojanowska 1983; Stojanowska and Panek, 2002, 2005, 2004; Drozdowicz et al., 2007, 2012; Ławrynowicz et al., 2011; Wilga and Ciechanowski, 2007; Panek and Romański, 2010; Chachuła et al., 2021; Salamaga et al., 2016; Salamaga, 2021; Ślusarczyk, 2021; Paul et al., 2023 |
| *Stemonitis herbatica* Peck, 1873 | Michalski, 1951; Bochynek & Drozdowicz, 2012; Panek and Romański, 2010; Salamaga et al., 2016 |
| *Stemonitis pallida* Wingate, 1899 | Błoński, 1890; Krupa, 1886, 1888; Jarocki, 1924; Krzemieniewska, 1933, 1947, 1960b; Kalinowska-Kucharska, 1975; Drozdowicz, 1992; Krawiec, 1965; Wrońska, 1974; Stojanowska, 1972, 1980b, 1983, 1984, 2000a; Ławrynowicz et al., 2011; Stojanowska 1983; Stojanowska and Panek, 2004, 2005; Panek and Romański, 2010; Ślusarczyk, 2010, 2021; Salamaga, 2021; Chachuła et al., 2021 |
| *Stemonitis splendens* Rostaf., 1874 | Błoński, 1890; Krupa, 1886, 1888; Jarocki, 1924; Krzemieniewska, 1933, 1934, 1957, 1960b; Wrońska, 1974; Krzemieniewska and Badura, 1954; Drozdowicz, 1992; Stojanowska, 1972, 1977b, 1983, 1984, 1992; Stojanowska 1983; Stojanowska and Panek, 2002; Panek and Romański, 2010; Wilga and Ciechanowski, 2007; Stojanowska, 2004a, 2004b; Salamaga, 2021 |
| *Stemonitis virginiensis* Rex, 1891 | Krzemieniewska, 1933, 1934, 1957, 1960b; Jarocki, 1924; Stojanowska, 1972, 1977b, 1992; Wrońska, 1974; Stojanowska 1983; Panek and Romański, 2010; Stojanowska, 2004a, 2004b |
| *Stemonitopsis aequalis* (Peck) Y. Yamam., 1998 | Chachuła et al., 2021 |
| *Stemonitopsis amoena* (Nann. -Bremek.) Nann. -Bremek., 1975 | Salamaga et al., 2016; Salamaga, 2021 |
| *Stemonitopsis gracilis* (G. Lister) Nann. -Bremek., 1975 | Bochynek and Drozdowicz, 2011, 2012 |
| *Stemonitopsis hyperopta* (Meyl.) Nann. -Bremek., 1975 | Rostafiński, 1875; Krupa, 1889; Jarocki, 1924; Krzemieniewska, 1933, 1947, 1957, 1960b; Krzemieniewska and Badura, 1954; Kalinowska-Kucharska, 1975; Drozdowicz, 1992; Stojanowska, 1972, 1983; Wrońska, 1974; Stojanowska 1983; Panek and Romański, 2010; Bochynek and Drozdowicz, 2011, 2012; Salamaga et al., 2016; Chachuła et al., 2021; Salamaga, 2021; Drozdowicz et al., 2012; Stojanowska, 1977a, 2004b; Stojanowska and Panek, 2004, 2005 |
| *Stemonitopsis reticulata* (H.C. Gilbert) Nann. -Bremek. & Y. Yamam., 1995 | Krzemieniewska, 1957, 1960b; Stojanowska, 1977b, 1983; Wrońska, 1974; Panek and Romański, 2010; Stojanowska 1983; Stojanowska, 2004b |
| *Stemonitopsis typhina* (F.H. Wigg.) Nann. -Bremek., 1967 | Gutwiński, 1901; Jarocki, 1924; Krupa, 1886; Błoński, 1890; Kalinowska-Kucharska, 1975; Krzemieniewska, 1933, 1947, 1957; Krzemieniewska and Badura, 1954; Wrońska, 1974; Michalski, 1951; Miśkiewicz, 2001; Drozdowicz, 1992, 2001; Stojanowska, 1970, 1972, 1977b, 1980b, 1981, 1983, 1984, 1992, 2000a; Stojanowska 1983; Stojanowska and Panek, 2002; Bochynek and Drozdowicz, 2011, 2012; Drozdowicz, 2005; Drozdowicz et al., 2007, 2012; Ławrynowicz et al., 2011; Panek and Romański, 2010; Ślusarczyk, 2010; Salamaga et al., 2016; Salamaga, 2021 |
| *Symphytocarpus amaurochaetoides* Nann. -Bremek., 1967 | Krzemieniewska, 1933, 1957, 1960b; Wrońska, 1974; Stojanowska, 1972, 1977b, 1992; Stojanowska, 2004b; Salamaga, 2021 |
| *Symphytocarpus flaccidus* (Lister) Ing & Nann. -Bremek., 1967 | Krzemieniewska, 1933; Drozdowicz et al., 2012; Panek and Romański, 2010; Salamaga et al., 2016; Stojanowska, 2004b; Stojanowska and Panek, 2004; Bochynek & Drozdowicz, 2012 |
| *Trichamphora pezizoidea* Jungh., 1838 | Drozdowicz et al., 2003 |
| *Trichia alpina* (R.E. Fr.) Meyl., 1921 | Rostafiński, 1875; Drozdowicz, 1988; Ronikier et al., 2008; Stojanowska, 2004; Paul et al., 2023 |
| *Trichia botrytis* (J.F. Gmel.) Pers., 1794 | Krupa, 1886, 1888; Jarocki, 1924; Krzemieniewska, 1947, 1960b; Krzemieniewska and Badura, 1954; Miśkiewicz, 2001; Drozdowicz, 1992, 2005; Wrońska, 1974; Stojanowska, 1972, 1977b, 1980b, 1983, 1984, 2000a; Stojanowska 1983; Wilga and Ciechanowski, 2007; Stojanowska and Panek, 2002, 2005, 2004; Drozdowicz et al., 2012; Panek and Romański, 2010; Salamaga, 2021 |
| *Trichia contorta* (Ditmar) Rostaf., 1875 | Rostafiński, 1875; Krupa, 1889; Jarocki, 1924; Krawiec, 1965; Krzemieniewska, 1947, 1960b; Wrońska, 1974; Drozdowicz, 1992; Stojanowska, 1970, 1972, 1977b, 1980b, 1983, 1984; Ławrynowicz et al., 2011; Stojanowska 1983; Stojanowska, 2004; Stojanowska and Panek, 2002, 2004; Paul et al., 2023 |
| *Trichia crateriformis* G.W. Martin, 1963 | Kalinowska-Kucharska, 1975; Stojanowska 1983; Stojanowska, 1977; Drozdowicz, 2005; Stojanowska and Panek, 2005; Paul et al., 2023 |
| *Trichia erecta* Rex, 1890 | Krzemieniewska, 1957, 1960b; Drozdowicz, 2003a; Stojanowska, 2000b, 2004; Stojanowska and Panek, 2005 |
| *Trichia flavicoma* (Lister) Ing, 1967 | Bochynek, 2015 |
| *Trichia munda* (Lister) Meyl., 1927 | Krzemieniewska, 1933; Drozdowicz et al., 2003 |
| *Trichia scabra* Rostaf., 1875 | Krupa, 1886, 1889; Błoński and Drymmer, 1889; Jarocki, 1924; Krzemieniewska, 1929, 1933, 1947, 1957; Krzemieniewska and Badura, 1954; Krawiec, 1965; Kalinowska-Kucharska, 1975; Wrońska, 1974; Drozdowicz, 1992, 2005, 2003a; Stojanowska, 1970, 1972, 1977b, 1980, 1980b, 1983, 1984, 2000a, 2004; Stojanowska 1983; Stojanowska and Panek, 2002, 2005, 2004; Bochynek and Drozdowicz, 2011; Drozdowicz et al., 2007, 2012; Ławrynowicz et al., 2011; Panek and Romański, 2010; Ślusarczyk, 2010; Salamaga, 2021 |
| *Trichia sordida* Johannesen, 1984 | Wilga and Ciechanowski, 2007; Ronikier and Janik, 2020; Paul et al., 2023 |
| *Trichia subfusca* Rex, 1890 | Krzemieniewska, 1960b; Paul et al., 2023 |
| *Trichia varia* (Pers. ex J. F. Gmel.) Pers., 1794 | Krupa, 1886, 1887, 1889; Błoński and Drymmer, 1889; Jarocki, 1924; Krzemieniewska, 1933, 1947, 1957; Krzemieniewska and Badura, 1954; Kalinowska-Kucharska, 1975; Krawiec, 1965; Krzysztofiak et al., 2010; Wrońska, 1974; Drozdowicz, 1992, 2001, 2003a, 2005; Drozdowicz and Wilga, 2002; Miśkiewicz, 2001; Stojanowska, 1970, 1972, 1977b, 1980, 1983, 1984, 1992, 2000a, 2004; Stojanowska 1983; Stojanowska and Panek, 2002, 2005, 2004; Bochynek and Drozdowicz, 2011, 2012; Drozdowicz et al., 2007, 2012; Ławrynowicz et al., 2011; Panek and Romański, 2010; Ślusarczyk, 2010; Wilga and Ciechanowski, 2007; Paul et al., 2023 |
| *Tubifera ferruginosa* (Batsch) J. F. Gmel., 1792 | Błoński, 1890; Kwieciński, 1896; Krupa, 1889; Gutwiński, 1901; Stecki, 1910; Jarocki, 1924; Kalinowska-Kucharska, 1975; Krawiec, 1965; Krzemieniewska, 1933, 1947, 1957; Krzemieniewska and Badura, 1954; Michalski, 1951; Miśkiewicz, 2001; Drozdowicz, 1992, 2003a, 2003b; Drozdowicz and Wilga, 2002; Wrońska, 1974; Stojanowska, 1972, 1977b, 1980b, 1983, 1984, 2000a; Stojanowska and Panek, 2002; Bochynek and Drozdowicz, 2011; Stojanowska 1983; Ławrynowicz et al., 2011; Panek and Romański, 2010; Drozdowicz et al., 2012; Salamaga et al., 2016; Salamaga, 2021; Chachuła et al., 2021; Ślusarczyk, 2010, 2021; Wilga and Ciechanowski, 2007; Paul et al., 2023 |
| *Valtocarpus trechisporus* (Berk. ex Torrend) Gmoshinskiy, Prikhodko, Bortnikov, Shchepin & Novozh., 2024 | Salamaga et al., 2014 |
