# Supplementary material for: Recorded and predicted occurrence of slime moulds (Eumycetozoa) in Poland from Central and Eastern European data
Source: PeerJ. 2026 Jul 9;14:e21492. doi: 10.7717/peerj.21492 (PMC13356830; doi:10.7717/peerj.21492)
Supplement: Supplemental Information 2 — For each of 11 orders in the regional pool, the table shows recorded species, candidates and their theoretical maximum total. [file peerj-14-21492-s002.docx]

| Order | Recorded (PL) | Candidate (region) | Total potential species (PL) |
| --- | --- | --- | --- |
| Acytosteliales | 0 | 1 | 1 |
| Cavosteliales | 0 | 3 | 3 |
| Ceratiomyxales | 2 | 1 | 3 |
| Cribrariales | 50 | 51 | 101 |
| Dictyosteliales | 0 | 4 | 4 |
| Echinosteliales | 3 | 13 | 16 |
| Liceales | 0 | 4 | 4 |
| Physarales | 112 | 132 | 244 |
| Protosteliales | 0 | 10 | 10 |
| Stemonitidales | 55 | 94 | 149 |
| Trichiales | 56 | 52 | 108 |
