# Supplementary figures and images for: Recorded and predicted occurrence of slime moulds (Eumycetozoa) in Poland from Central and Eastern European data (part 1 of 2)
Source: PeerJ. 2026 Jul 9;14:e21492. doi: 10.7717/peerj.21492 (PMC13356830; doi:10.7717/peerj.21492)

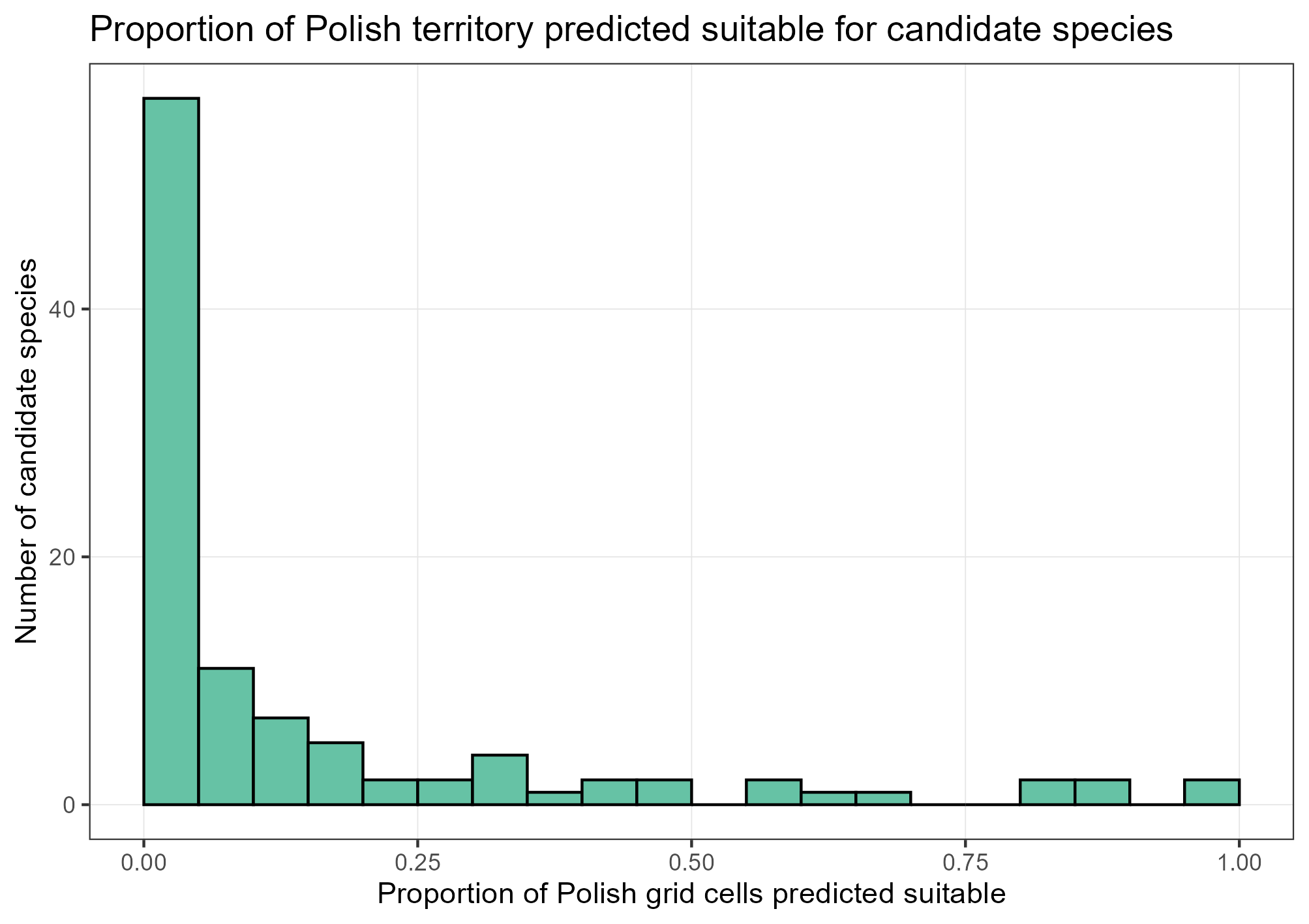

Supplement: Supplemental Information 3 — For each species, the share of 10-arc-minute cells above the 10th-percentile threshold is shown, from small to large suitable areas. [file peerj-14-21492-s003.png]

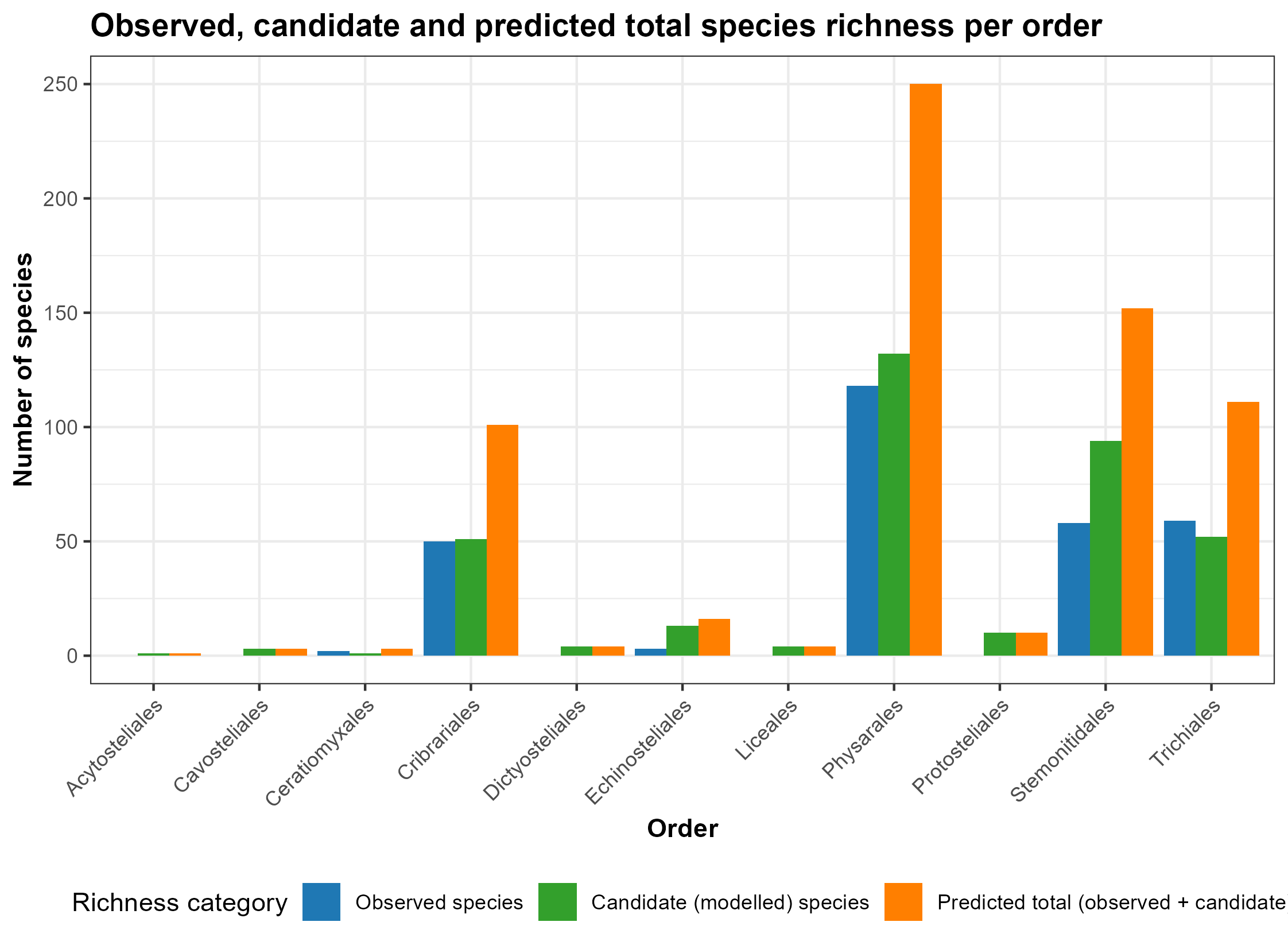

Supplement: Supplemental Information 4 — Bars show, for each of 11 orders in the regional pool, recorded species, additional candidates and their sum as the theoretical maximum per order. [file peerj-14-21492-s004.png]

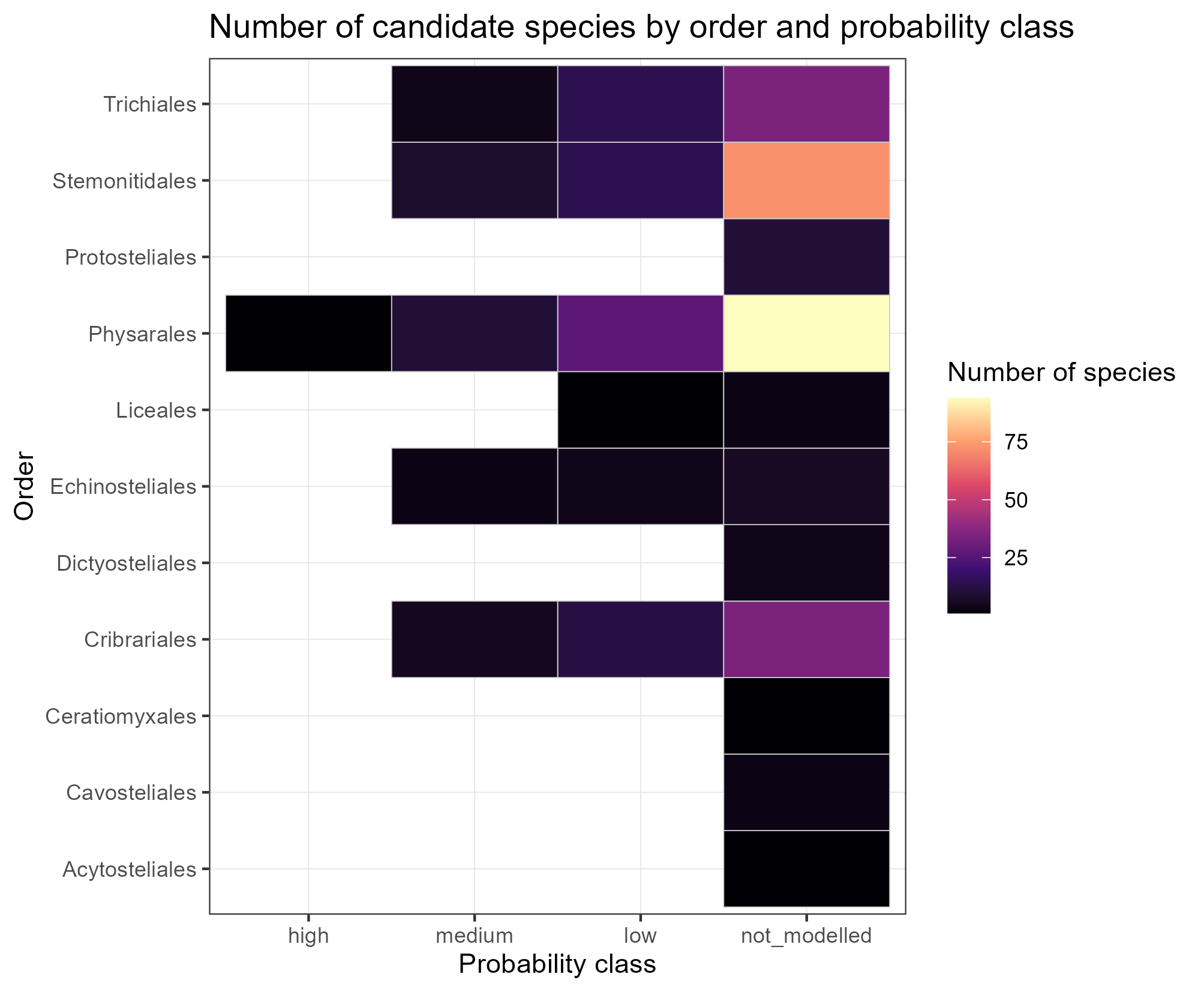

Supplement: Supplemental Information 5 — The heatmap shows, for each order, candidate species numbers in high, medium, low and not-modelled groups, highlighting Physarales and Stemonitidales. [file peerj-14-21492-s005.png]

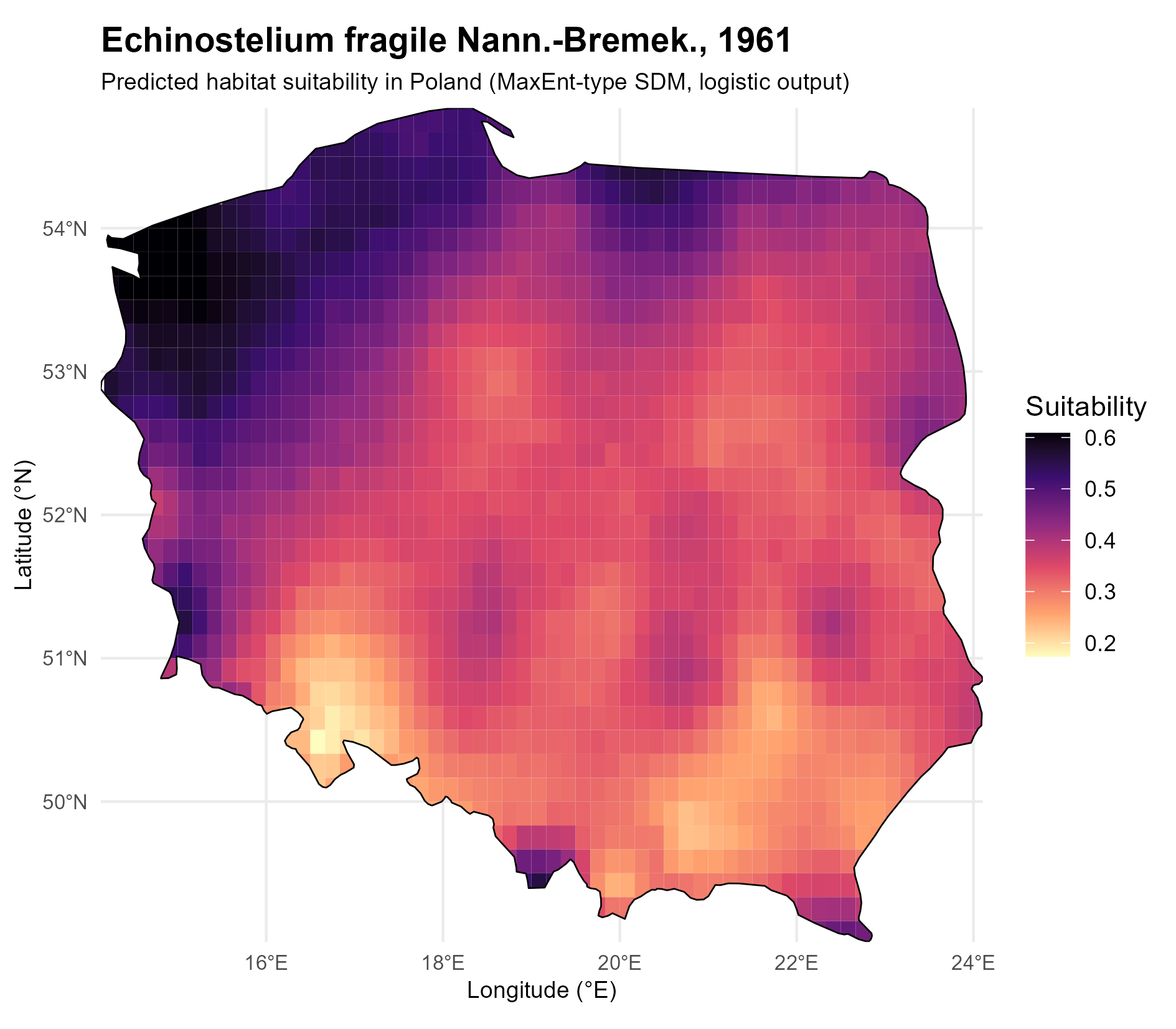

Supplement: Supplemental Information 12 — Set of 101 raster maps showing predicted potential distributions in Poland for modelled candidate species. Each figure displays continuous climatic suitability and the subset of grid cells exceeding a 10th-percentile training presence threshold. [file peerj-14-21492-s012.zip › Figure_SDM_poland_rank095_Echinostelium_fragile_Nann_Bremek_1961_MaxEnt_logistic.png]

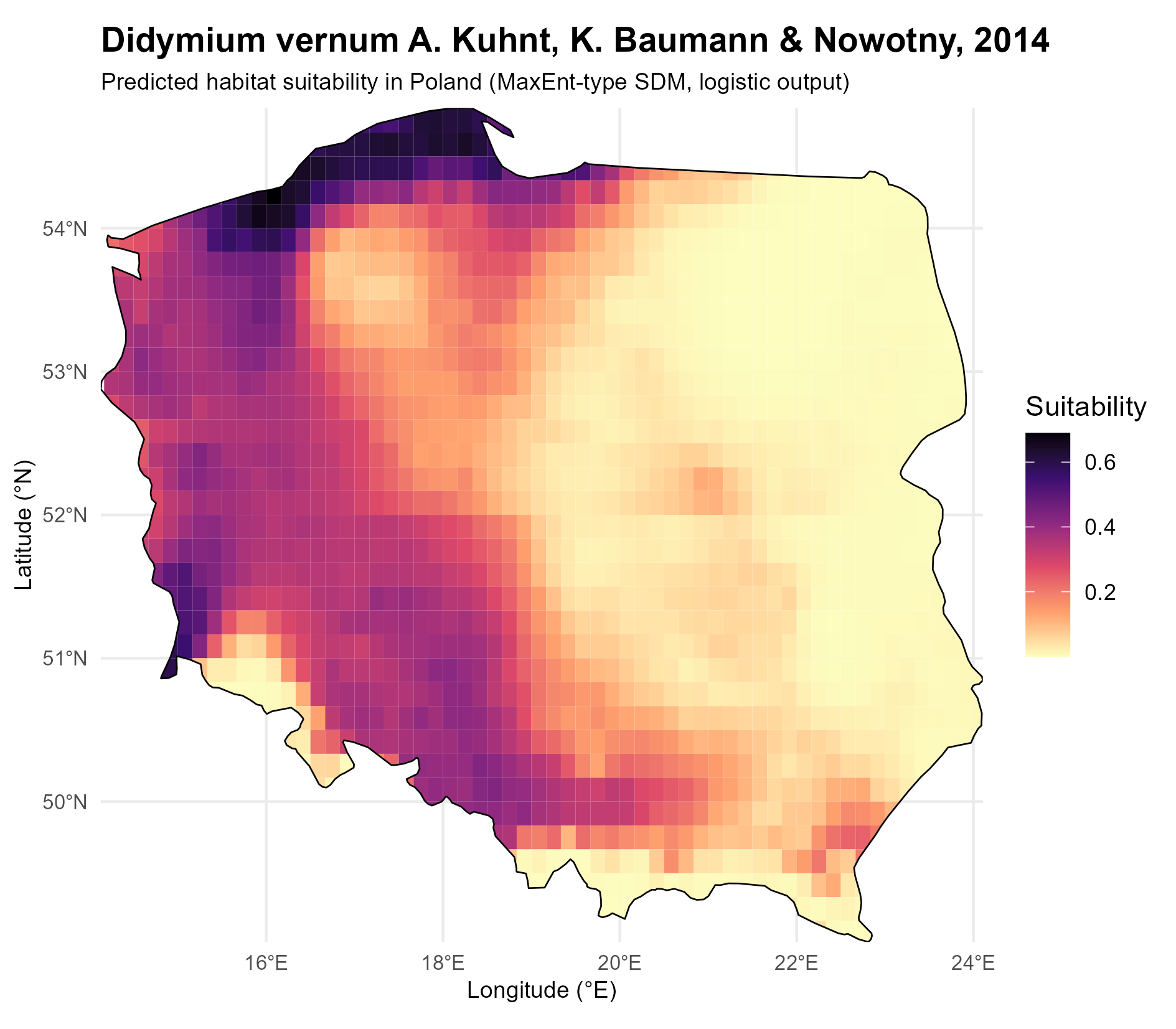

Supplement: Supplemental Information 12 — Set of 101 raster maps showing predicted potential distributions in Poland for modelled candidate species. Each figure displays continuous climatic suitability and the subset of grid cells exceeding a 10th-percentile training presence threshold. [file peerj-14-21492-s012.zip › Figure_SDM_poland_rank094_Didymium_vernum_A_Kuhnt_K_Baumann_Nowotny_2014_MaxEnt_logistic.png]

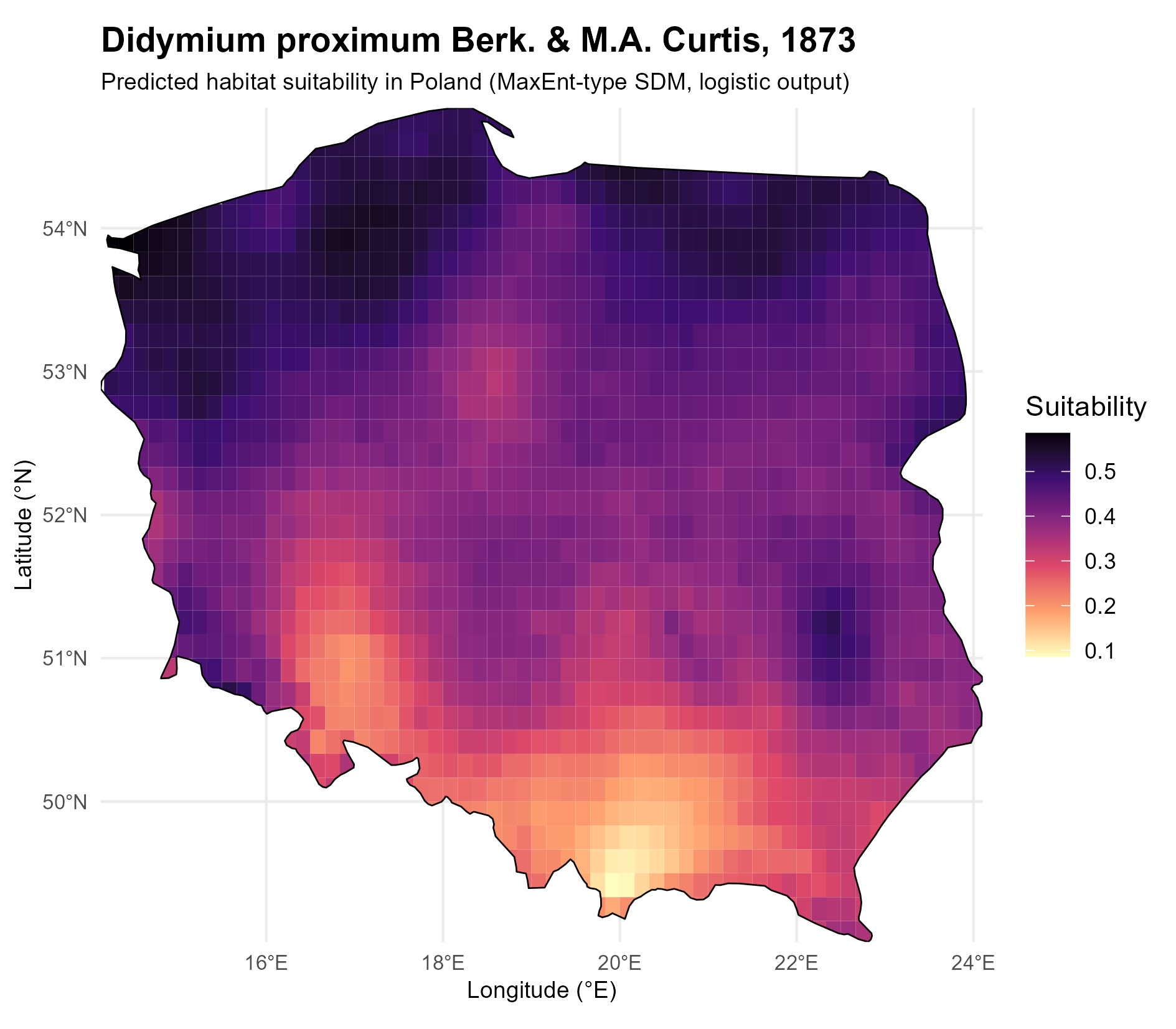

Supplement: Supplemental Information 12 — Set of 101 raster maps showing predicted potential distributions in Poland for modelled candidate species. Each figure displays continuous climatic suitability and the subset of grid cells exceeding a 10th-percentile training presence threshold. [file peerj-14-21492-s012.zip › Figure_SDM_poland_rank093_Didymium_proximum_Berk_M_A_Curtis_1873_MaxEnt_logistic.png]

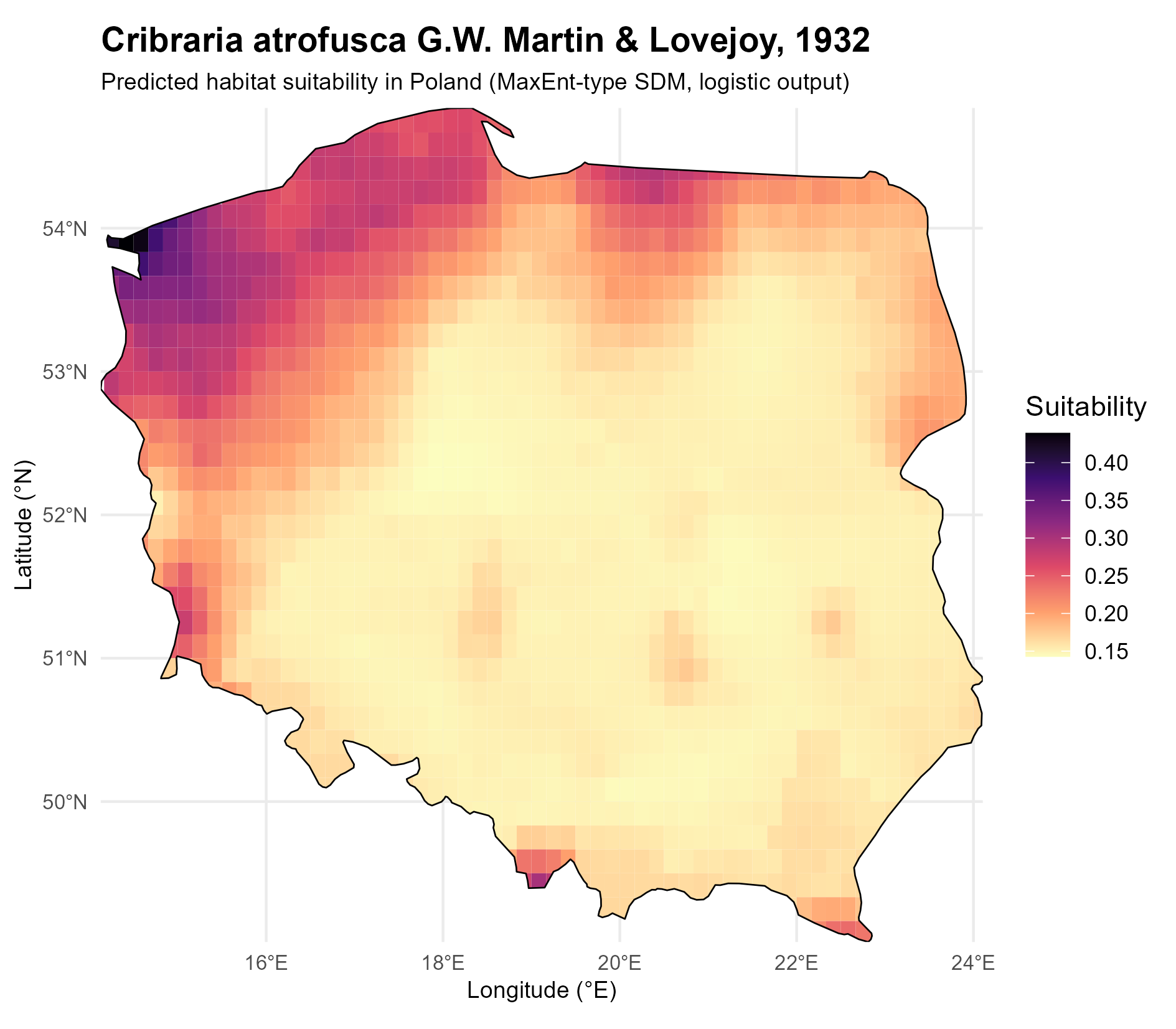

Supplement: Supplemental Information 12 — Set of 101 raster maps showing predicted potential distributions in Poland for modelled candidate species. Each figure displays continuous climatic suitability and the subset of grid cells exceeding a 10th-percentile training presence threshold. [file peerj-14-21492-s012.zip › Figure_SDM_poland_rank092_Cribraria_atrofusca_G_W_Martin_Lovejoy_1932_MaxEnt_logistic.png]

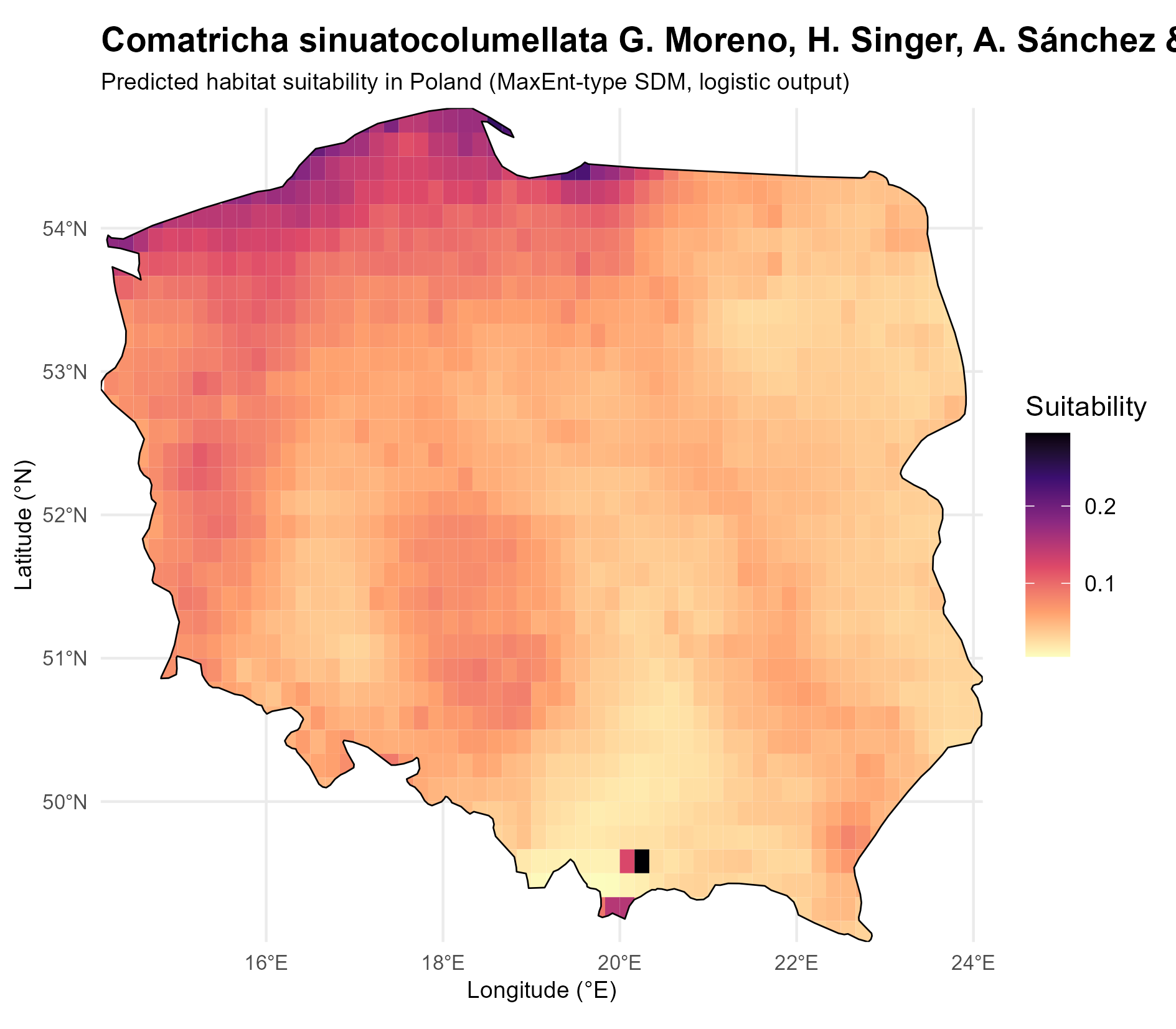

Supplement: Supplemental Information 12 — Set of 101 raster maps showing predicted potential distributions in Poland for modelled candidate species. Each figure displays continuous climatic suitability and the subset of grid cells exceeding a 10th-percentile training presence threshold. [file peerj-14-21492-s012.zip › Figure_SDM_poland_rank091_Comatricha_sinuatocolumellata_G_Moreno_H_Singer_A_Sanchez_Illana_2004_MaxEnt_logistic.png]

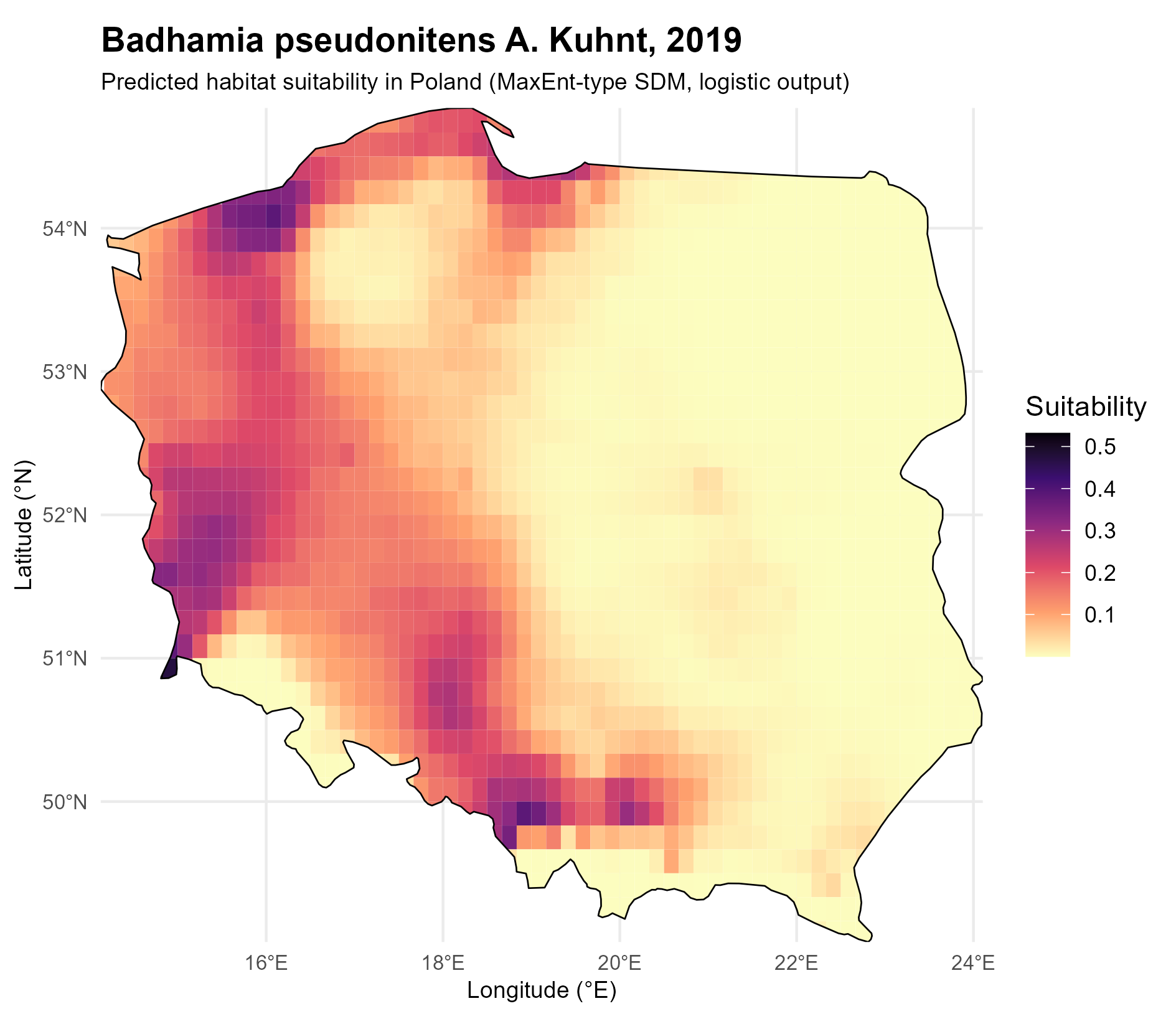

Supplement: Supplemental Information 12 — Set of 101 raster maps showing predicted potential distributions in Poland for modelled candidate species. Each figure displays continuous climatic suitability and the subset of grid cells exceeding a 10th-percentile training presence threshold. [file peerj-14-21492-s012.zip › Figure_SDM_poland_rank090_Badhamia_pseudonitens_A_Kuhnt_2019_MaxEnt_logistic.png]

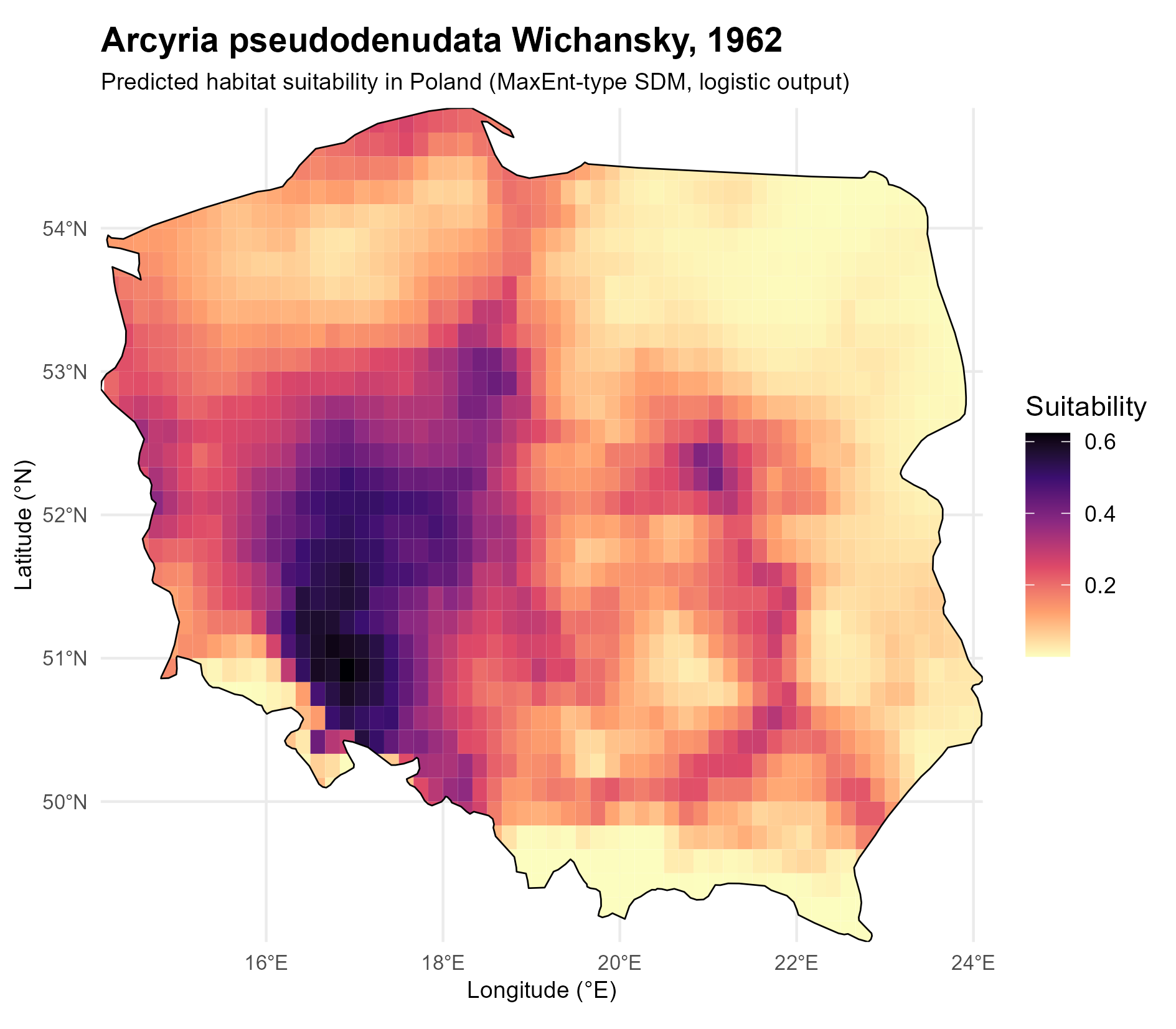

Supplement: Supplemental Information 12 — Set of 101 raster maps showing predicted potential distributions in Poland for modelled candidate species. Each figure displays continuous climatic suitability and the subset of grid cells exceeding a 10th-percentile training presence threshold. [file peerj-14-21492-s012.zip › Figure_SDM_poland_rank089_Arcyria_pseudodenudata_Wichansky_1962_MaxEnt_logistic.png]

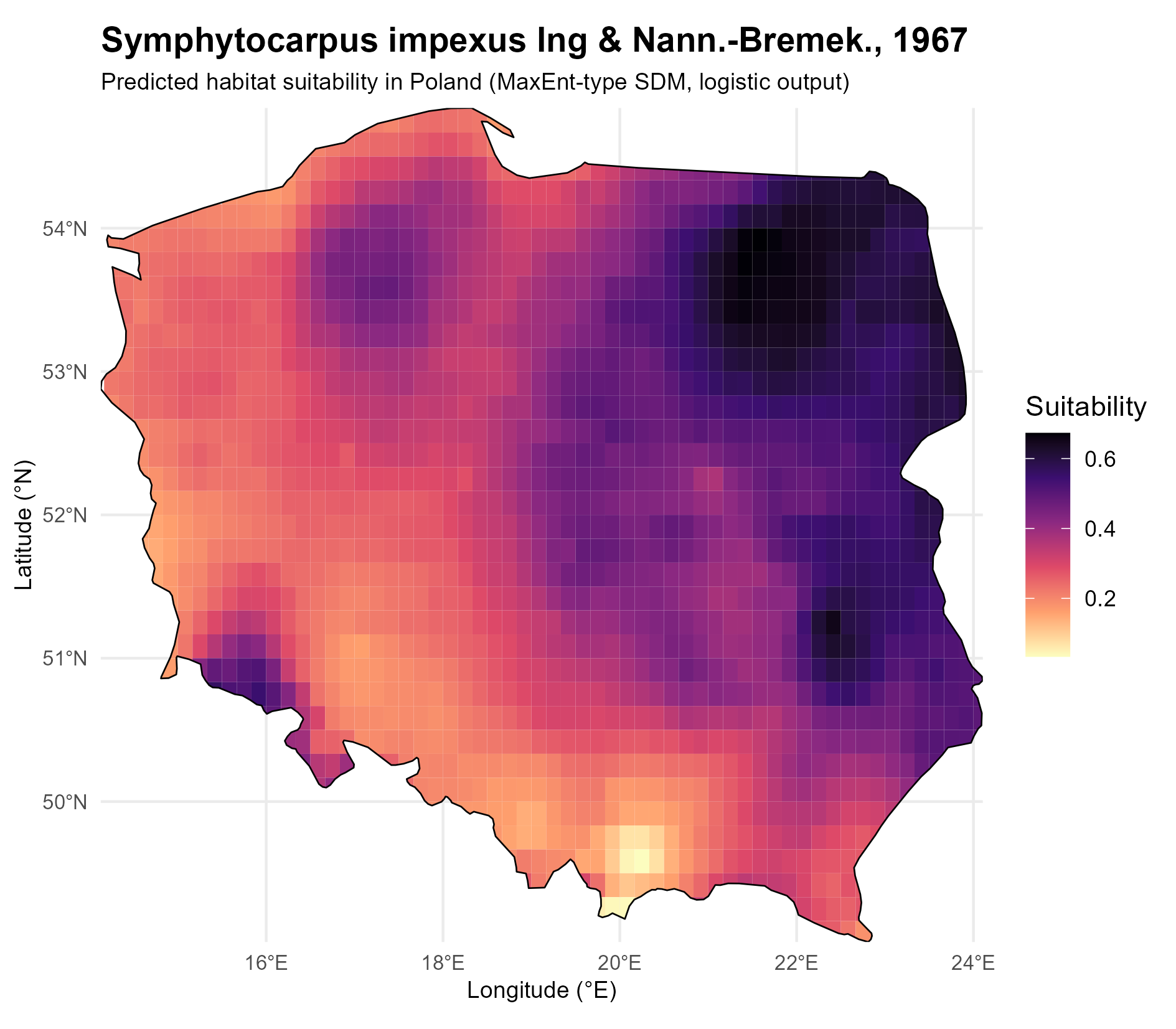

Supplement: Supplemental Information 12 — Set of 101 raster maps showing predicted potential distributions in Poland for modelled candidate species. Each figure displays continuous climatic suitability and the subset of grid cells exceeding a 10th-percentile training presence threshold. [file peerj-14-21492-s012.zip › Figure_SDM_poland_rank088_Symphytocarpus_impexus_Ing_Nann_Bremek_1967_MaxEnt_logistic.png]

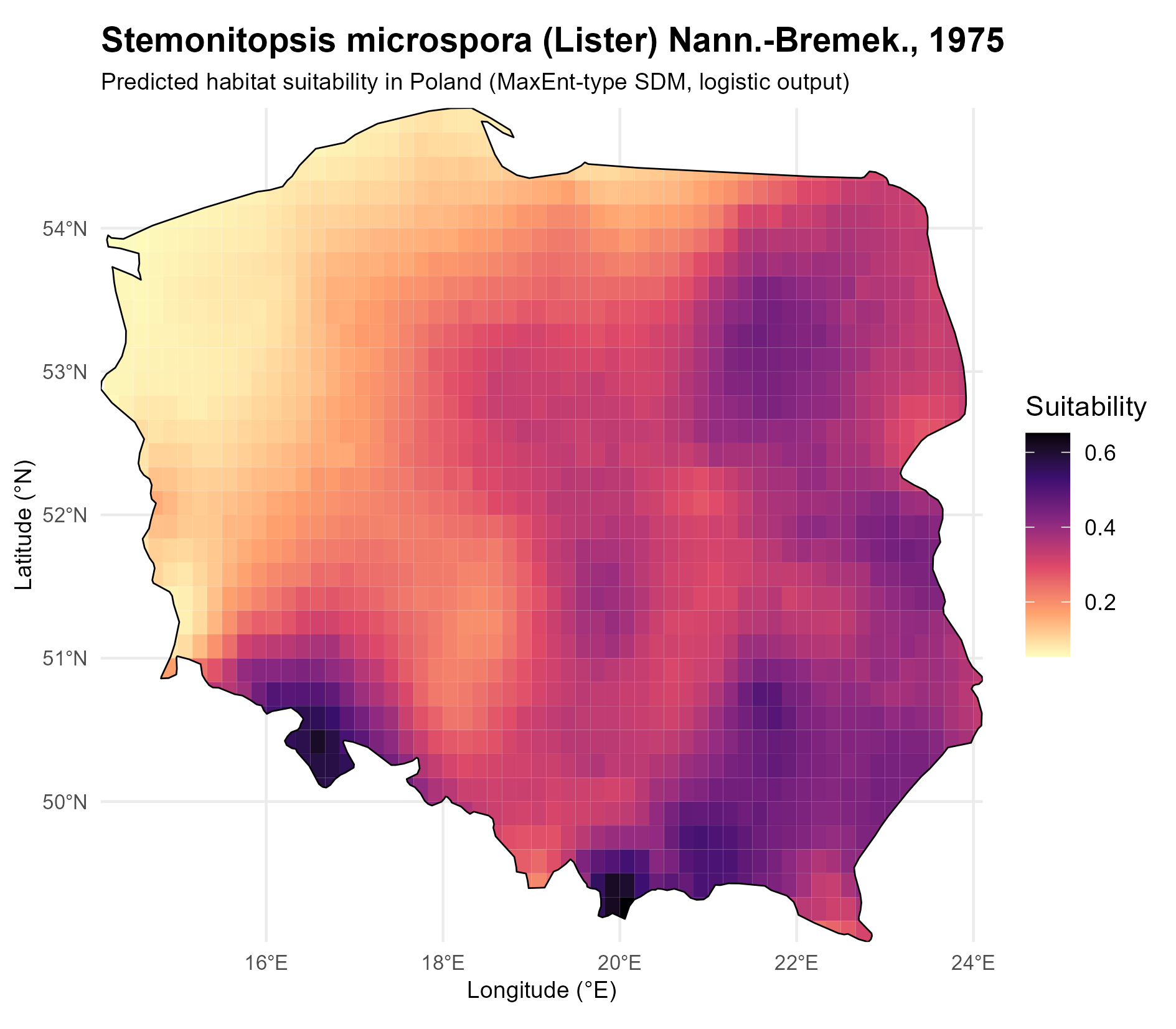

Supplement: Supplemental Information 12 — Set of 101 raster maps showing predicted potential distributions in Poland for modelled candidate species. Each figure displays continuous climatic suitability and the subset of grid cells exceeding a 10th-percentile training presence threshold. [file peerj-14-21492-s012.zip › Figure_SDM_poland_rank087_Stemonitopsis_microspora_Lister_Nann_Bremek_1975_MaxEnt_logistic.png]

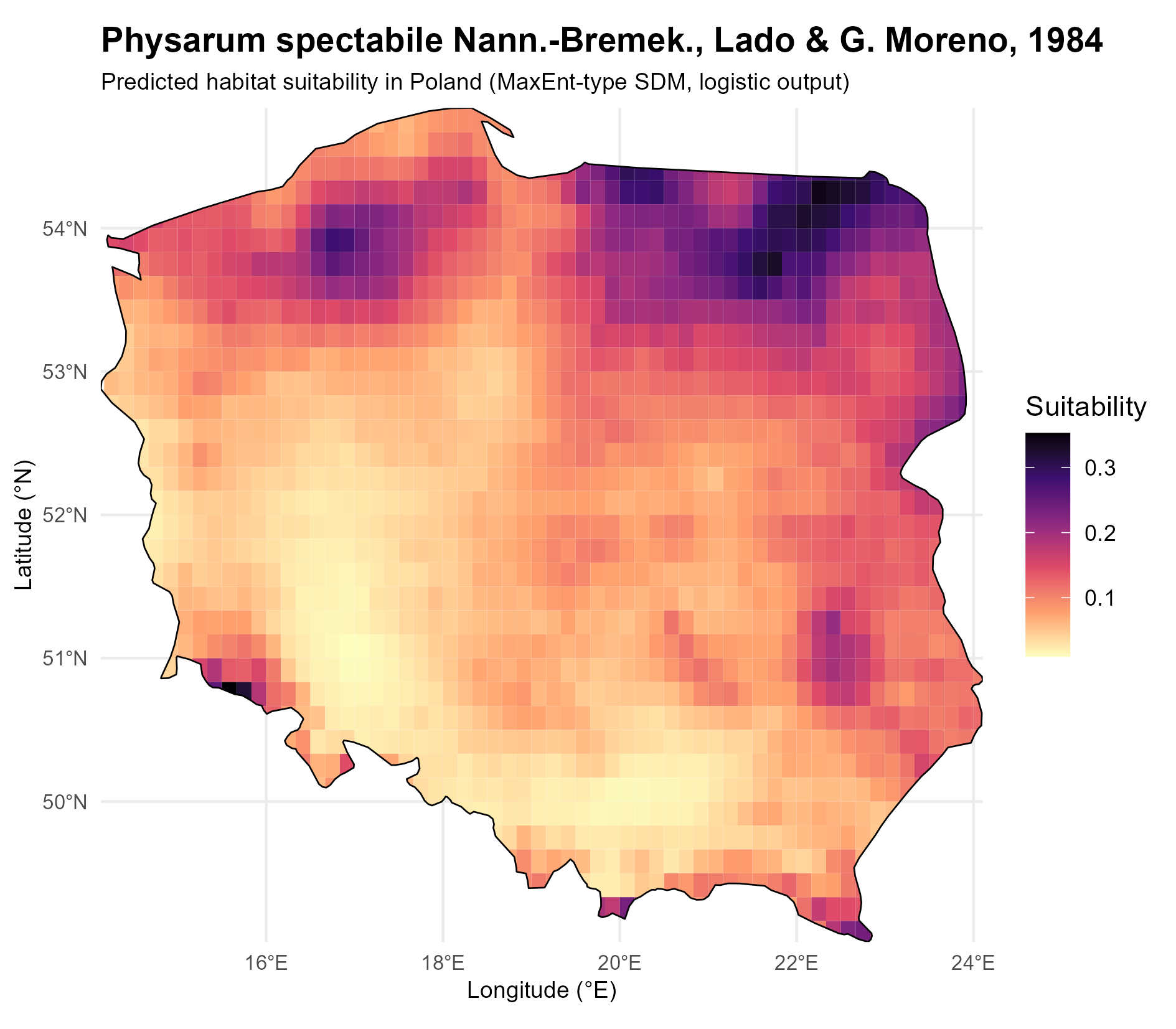

Supplement: Supplemental Information 12 — Set of 101 raster maps showing predicted potential distributions in Poland for modelled candidate species. Each figure displays continuous climatic suitability and the subset of grid cells exceeding a 10th-percentile training presence threshold. [file peerj-14-21492-s012.zip › Figure_SDM_poland_rank086_Physarum_spectabile_Nann_Bremek_Lado_G_Moreno_1984_MaxEnt_logistic.png]

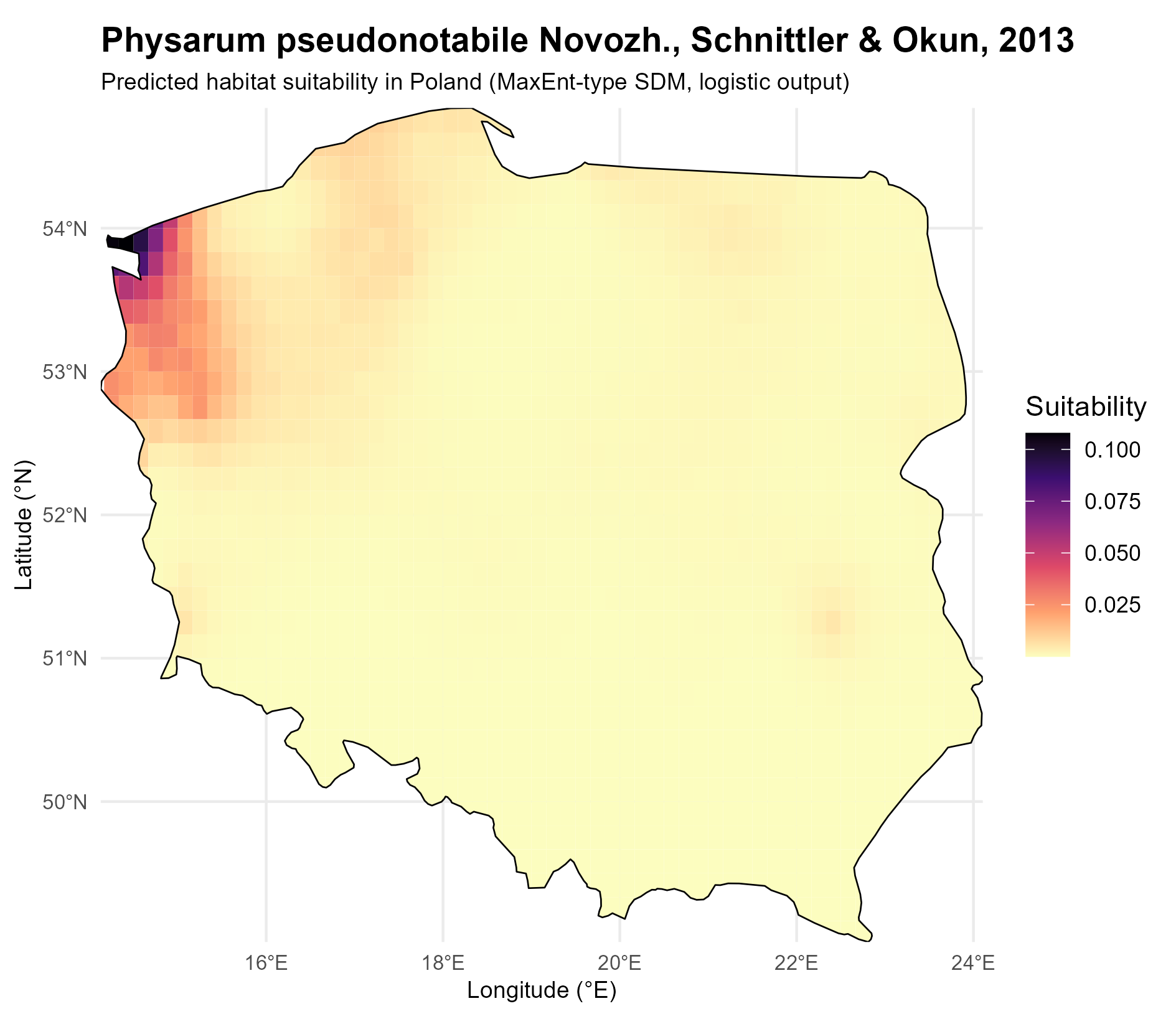

Supplement: Supplemental Information 12 — Set of 101 raster maps showing predicted potential distributions in Poland for modelled candidate species. Each figure displays continuous climatic suitability and the subset of grid cells exceeding a 10th-percentile training presence threshold. [file peerj-14-21492-s012.zip › Figure_SDM_poland_rank085_Physarum_pseudonotabile_Novozh_Schnittler_Okun_2013_MaxEnt_logistic.png]

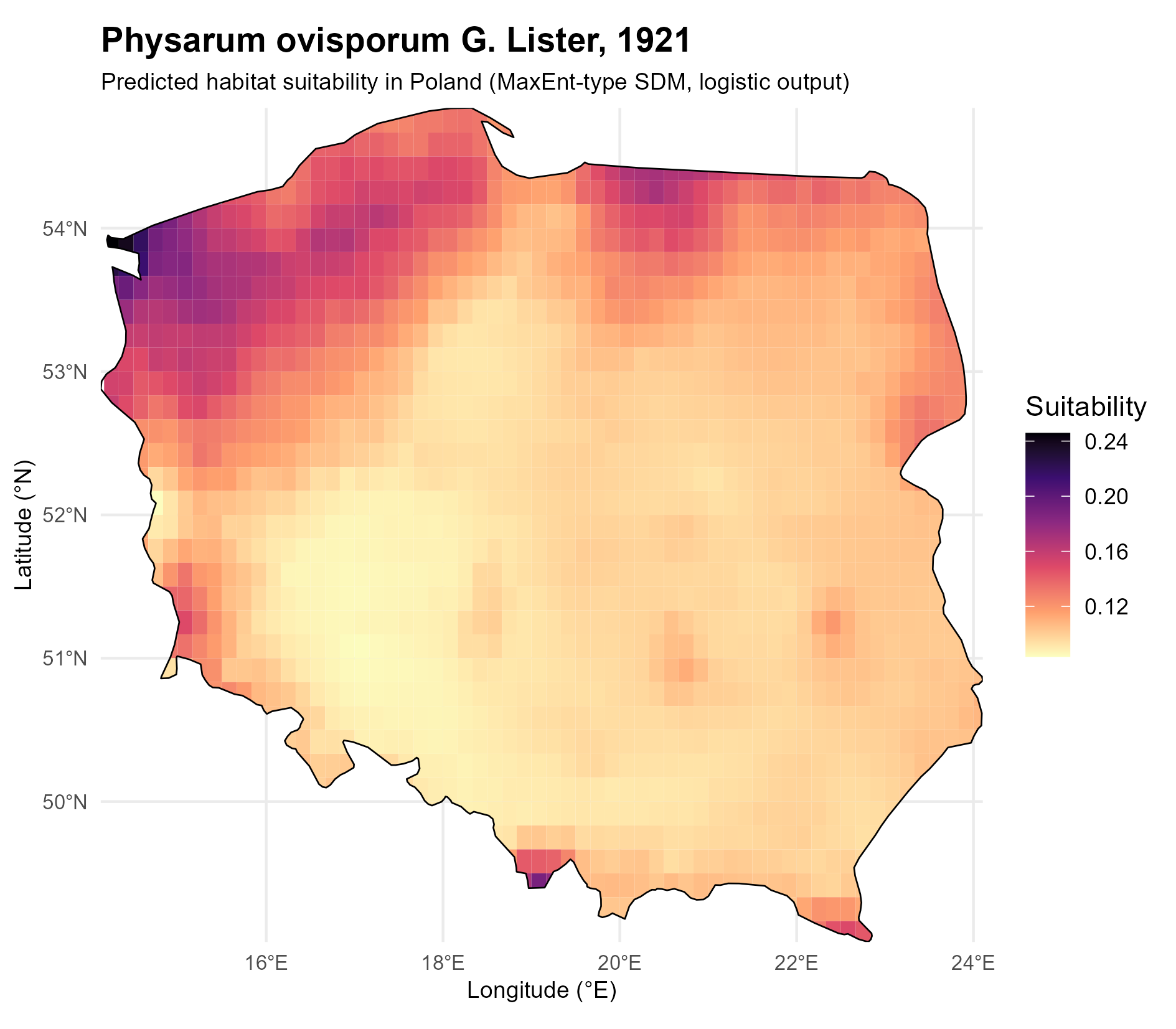

Supplement: Supplemental Information 12 — Set of 101 raster maps showing predicted potential distributions in Poland for modelled candidate species. Each figure displays continuous climatic suitability and the subset of grid cells exceeding a 10th-percentile training presence threshold. [file peerj-14-21492-s012.zip › Figure_SDM_poland_rank084_Physarum_ovisporum_G_Lister_1921_MaxEnt_logistic.png]

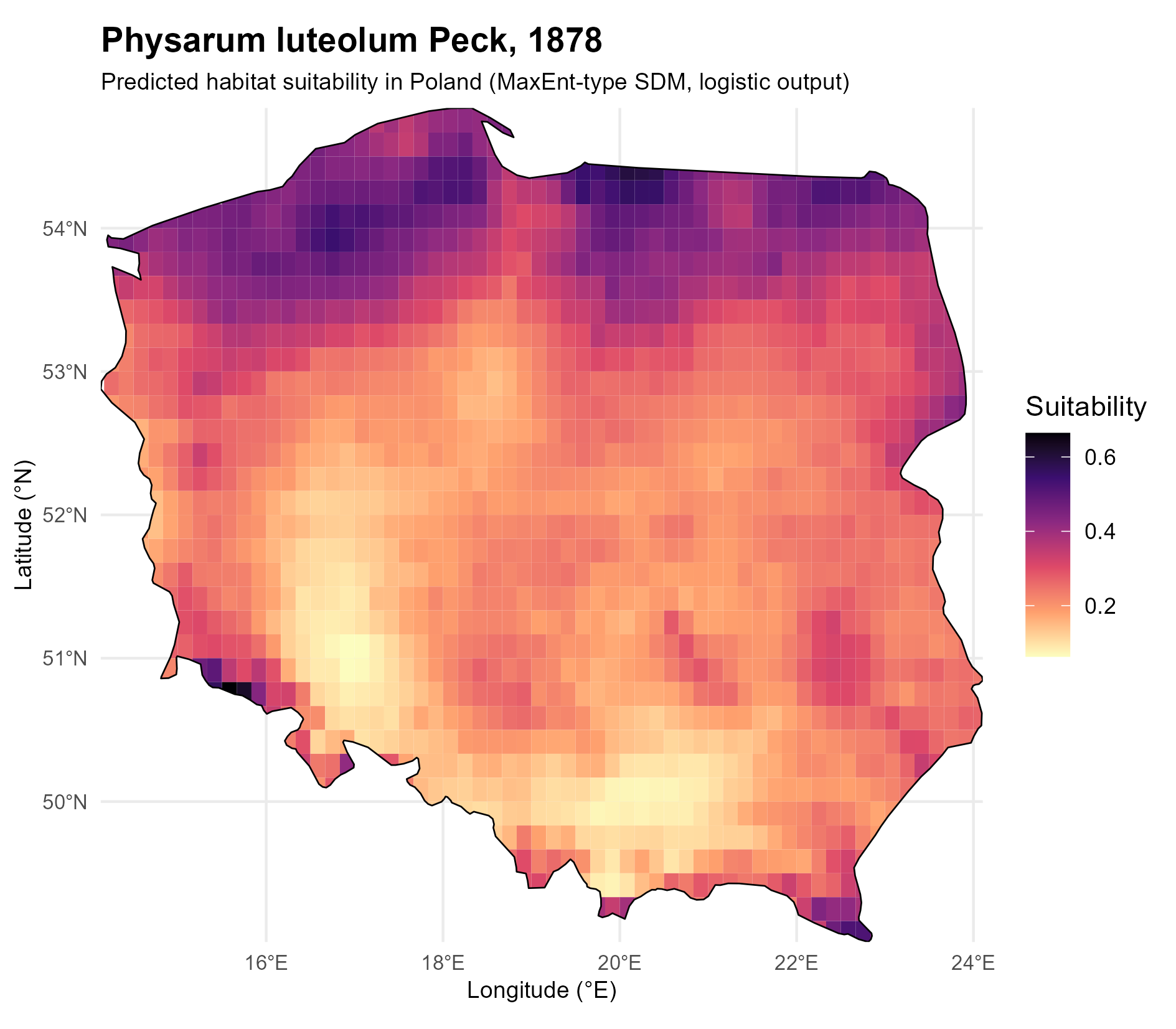

Supplement: Supplemental Information 12 — Set of 101 raster maps showing predicted potential distributions in Poland for modelled candidate species. Each figure displays continuous climatic suitability and the subset of grid cells exceeding a 10th-percentile training presence threshold. [file peerj-14-21492-s012.zip › Figure_SDM_poland_rank083_Physarum_luteolum_Peck_1878_MaxEnt_logistic.png]

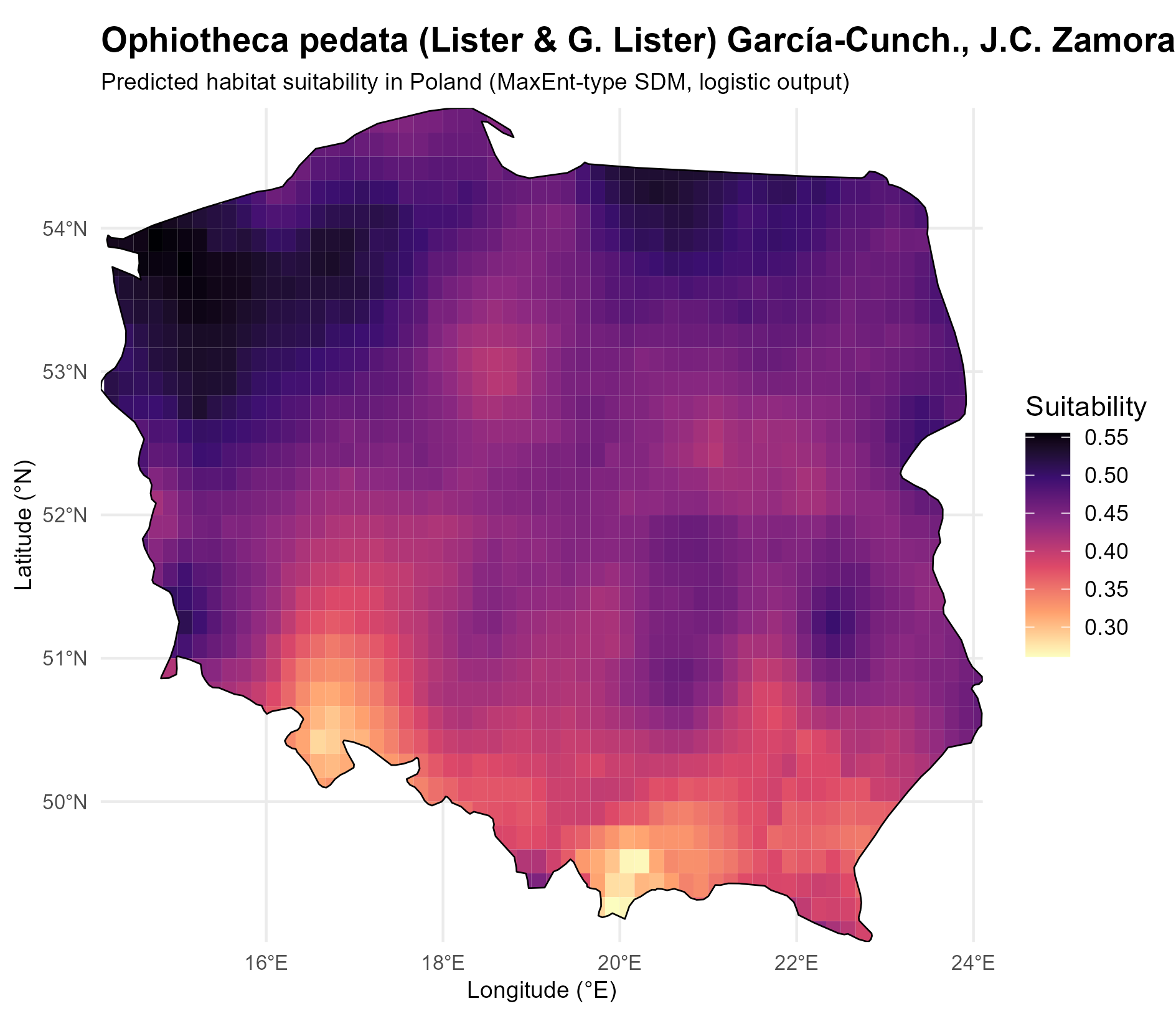

Supplement: Supplemental Information 12 — Set of 101 raster maps showing predicted potential distributions in Poland for modelled candidate species. Each figure displays continuous climatic suitability and the subset of grid cells exceeding a 10th-percentile training presence threshold. [file peerj-14-21492-s012.zip › Figure_SDM_poland_rank082_Ophiotheca_pedata_Lister_G_Lister_Garcia_Cunch_J_C_Zamora_Lado_2022_MaxEnt_logistic.png]

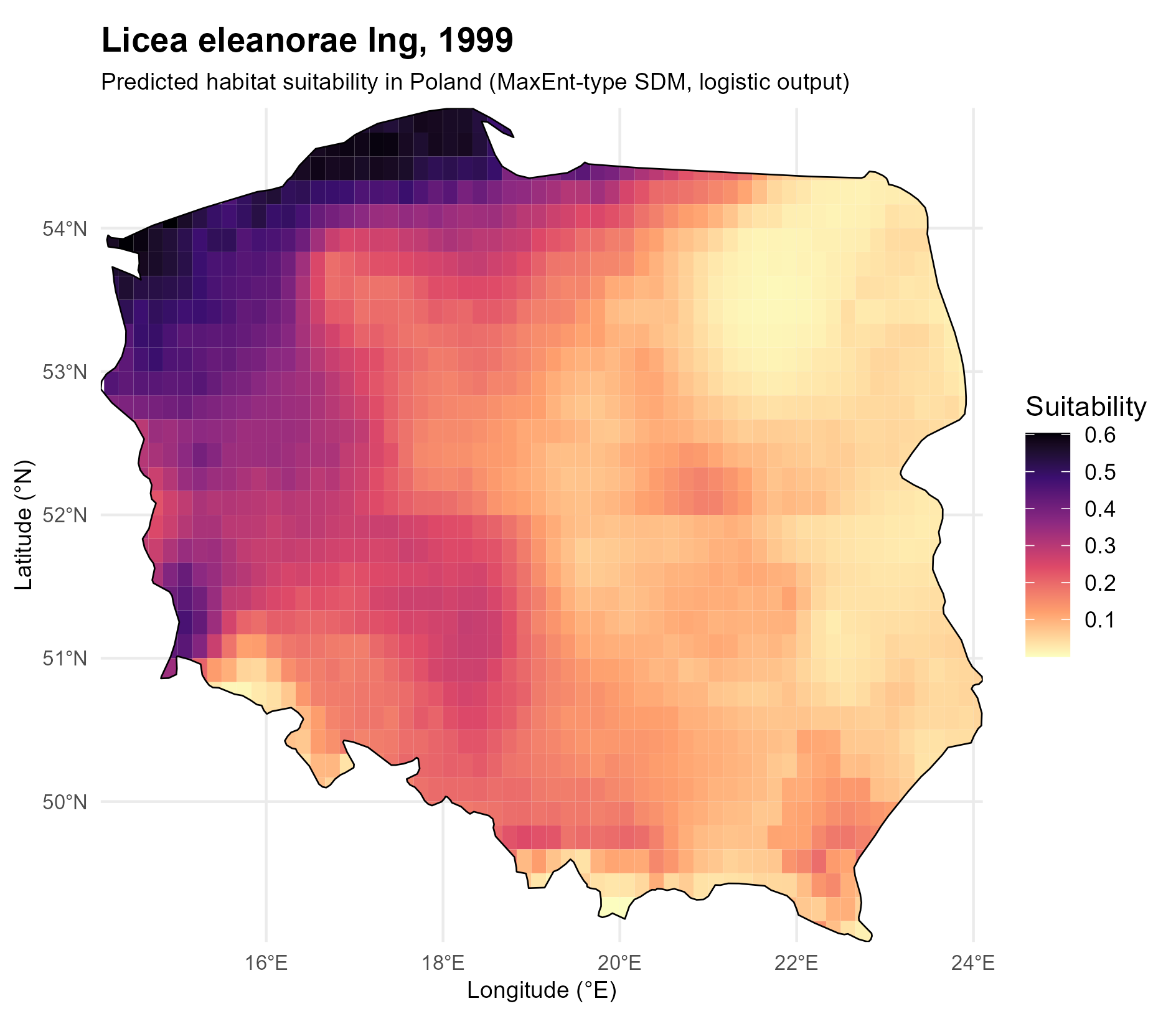

Supplement: Supplemental Information 12 — Set of 101 raster maps showing predicted potential distributions in Poland for modelled candidate species. Each figure displays continuous climatic suitability and the subset of grid cells exceeding a 10th-percentile training presence threshold. [file peerj-14-21492-s012.zip › Figure_SDM_poland_rank081_Licea_eleanorae_Ing_1999_MaxEnt_logistic.png]

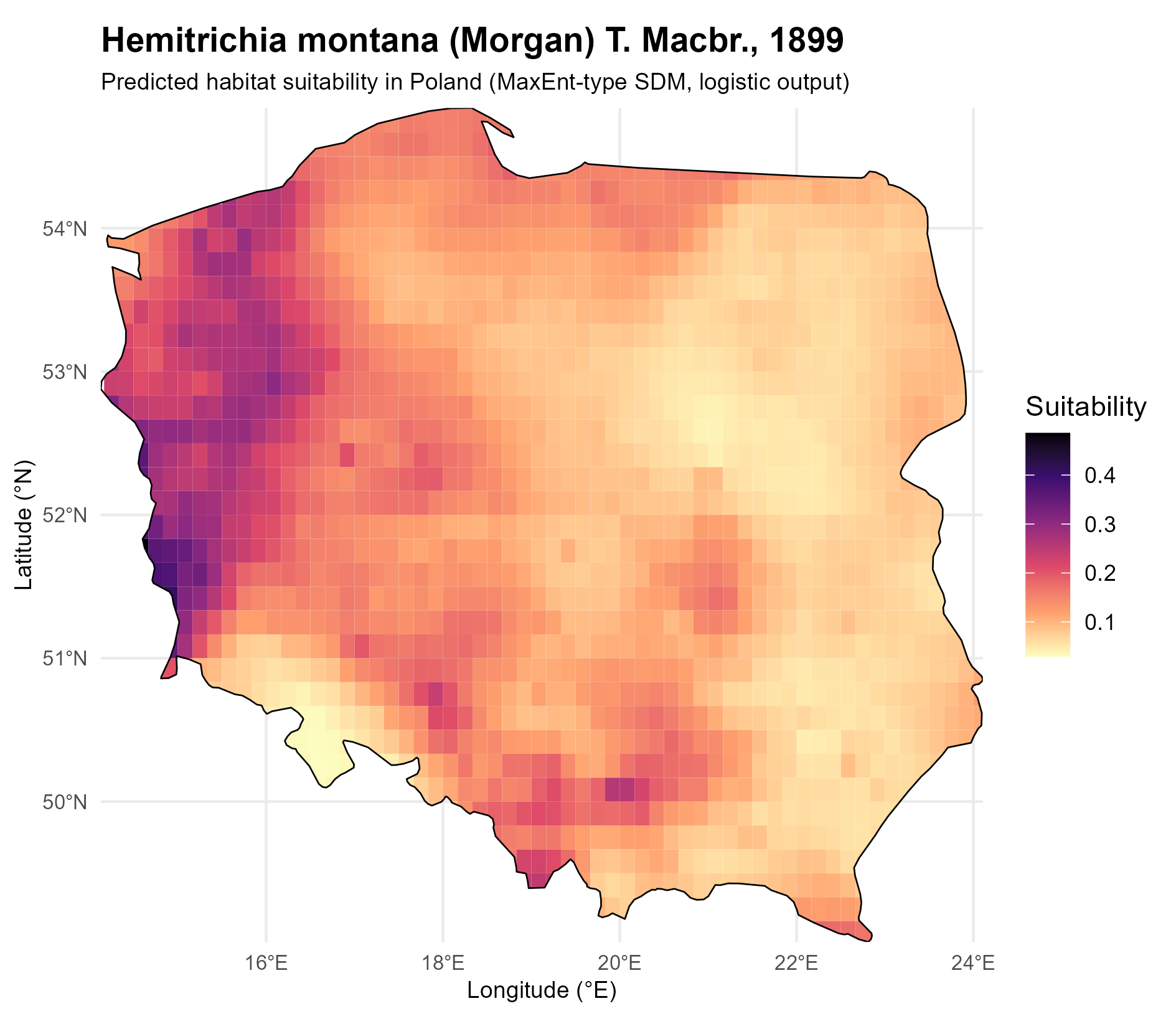

Supplement: Supplemental Information 12 — Set of 101 raster maps showing predicted potential distributions in Poland for modelled candidate species. Each figure displays continuous climatic suitability and the subset of grid cells exceeding a 10th-percentile training presence threshold. [file peerj-14-21492-s012.zip › Figure_SDM_poland_rank080_Hemitrichia_montana_Morgan_T_Macbr_1899_MaxEnt_logistic.png]

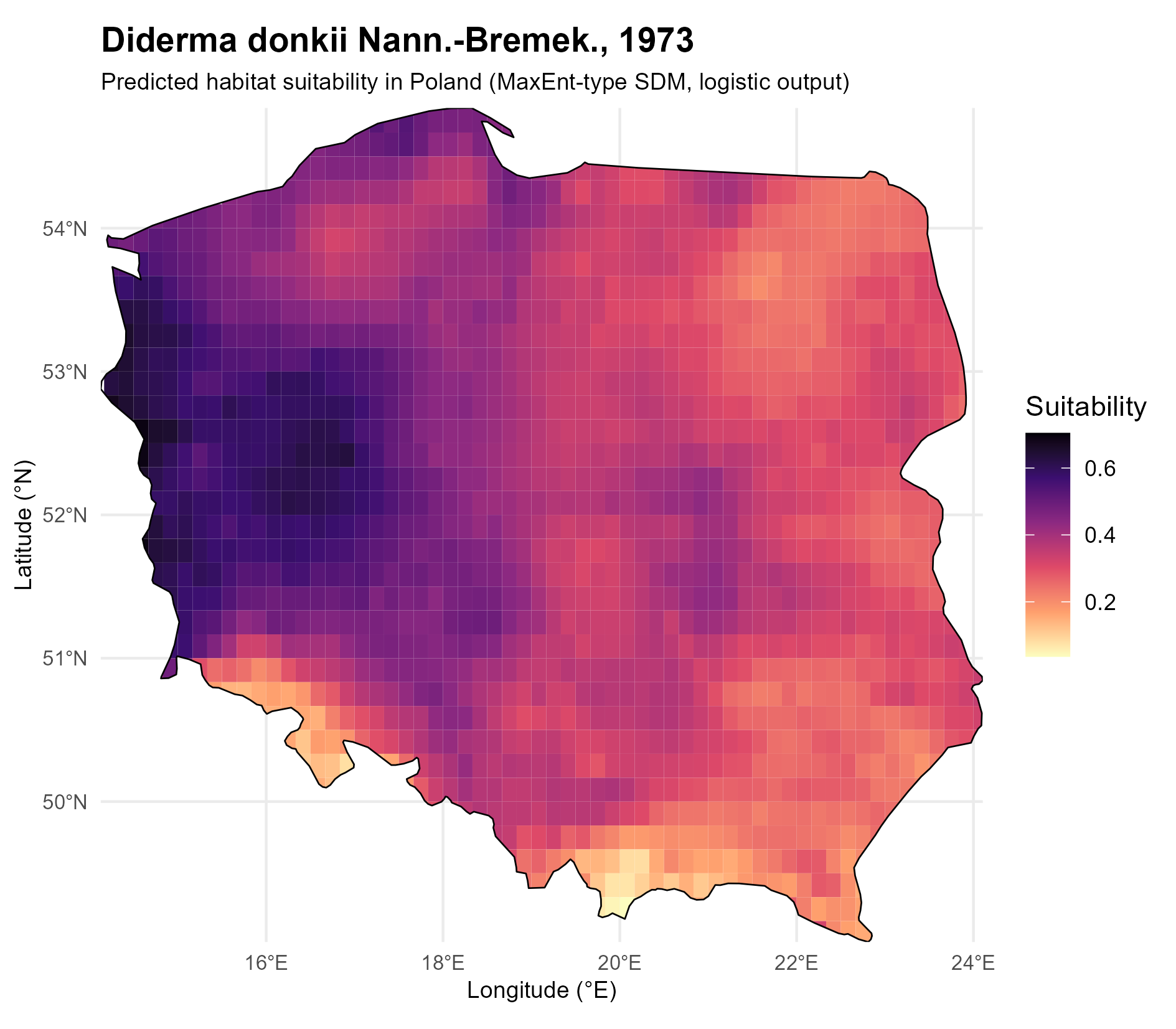

Supplement: Supplemental Information 12 — Set of 101 raster maps showing predicted potential distributions in Poland for modelled candidate species. Each figure displays continuous climatic suitability and the subset of grid cells exceeding a 10th-percentile training presence threshold. [file peerj-14-21492-s012.zip › Figure_SDM_poland_rank079_Diderma_donkii_Nann_Bremek_1973_MaxEnt_logistic.png]

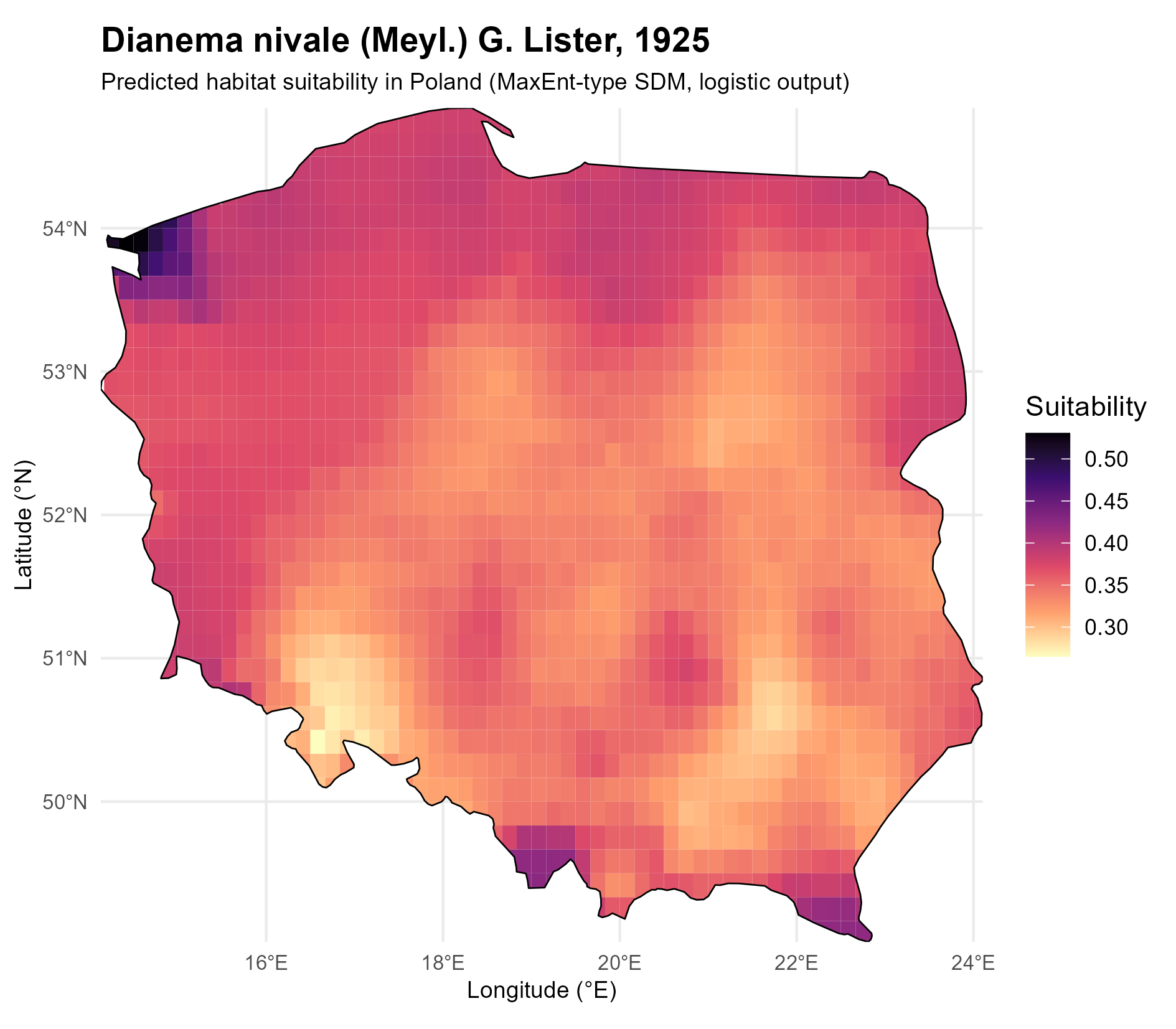

Supplement: Supplemental Information 12 — Set of 101 raster maps showing predicted potential distributions in Poland for modelled candidate species. Each figure displays continuous climatic suitability and the subset of grid cells exceeding a 10th-percentile training presence threshold. [file peerj-14-21492-s012.zip › Figure_SDM_poland_rank078_Dianema_nivale_Meyl_G_Lister_1925_MaxEnt_logistic.png]

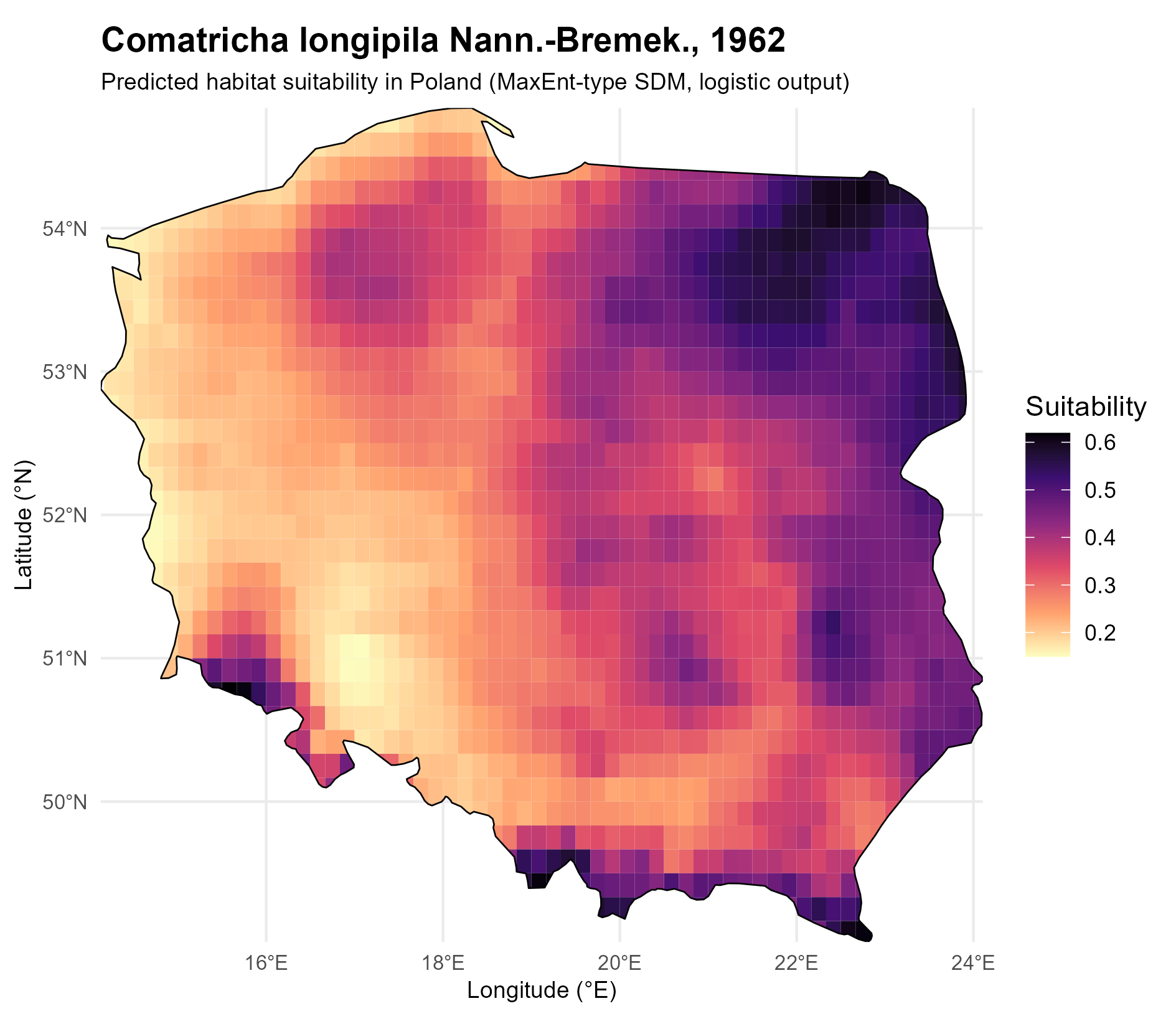

Supplement: Supplemental Information 12 — Set of 101 raster maps showing predicted potential distributions in Poland for modelled candidate species. Each figure displays continuous climatic suitability and the subset of grid cells exceeding a 10th-percentile training presence threshold. [file peerj-14-21492-s012.zip › Figure_SDM_poland_rank077_Comatricha_longipila_Nann_Bremek_1962_MaxEnt_logistic.png]

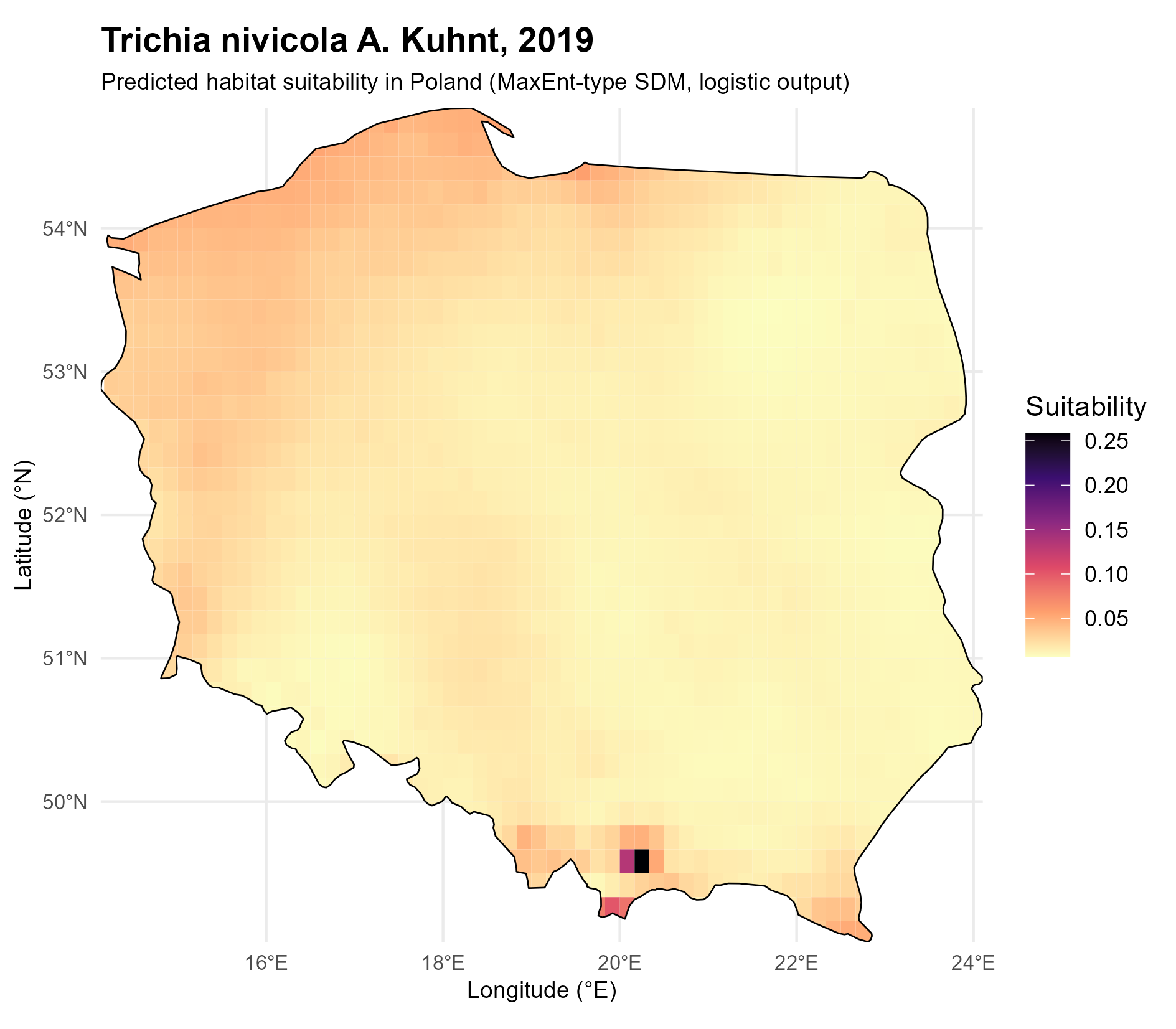

Supplement: Supplemental Information 12 — Set of 101 raster maps showing predicted potential distributions in Poland for modelled candidate species. Each figure displays continuous climatic suitability and the subset of grid cells exceeding a 10th-percentile training presence threshold. [file peerj-14-21492-s012.zip › Figure_SDM_poland_rank076_Trichia_nivicola_A_Kuhnt_2019_MaxEnt_logistic.png]

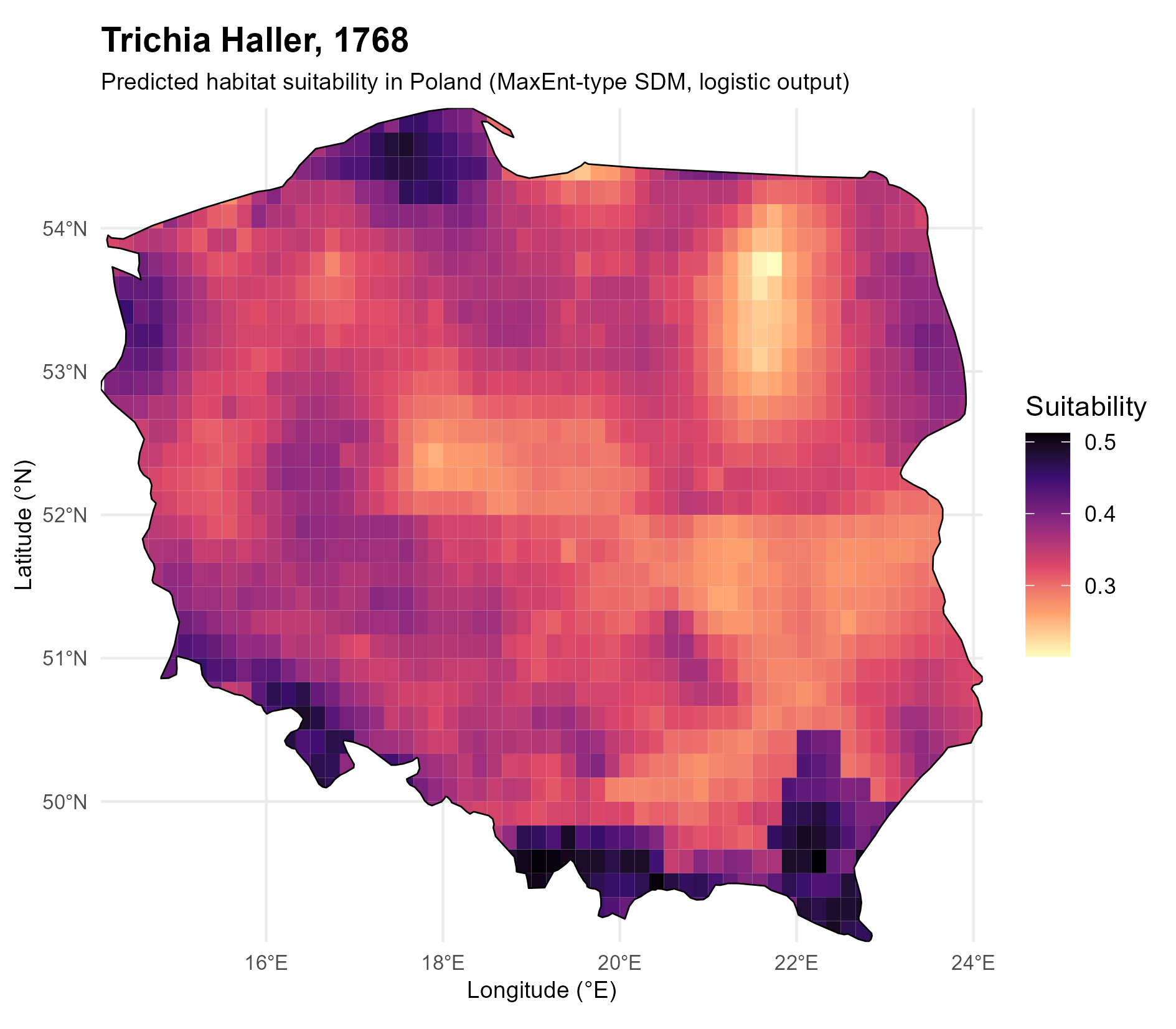

Supplement: Supplemental Information 12 — Set of 101 raster maps showing predicted potential distributions in Poland for modelled candidate species. Each figure displays continuous climatic suitability and the subset of grid cells exceeding a 10th-percentile training presence threshold. [file peerj-14-21492-s012.zip › Figure_SDM_poland_rank075_Trichia_Haller_1768_MaxEnt_logistic.png]

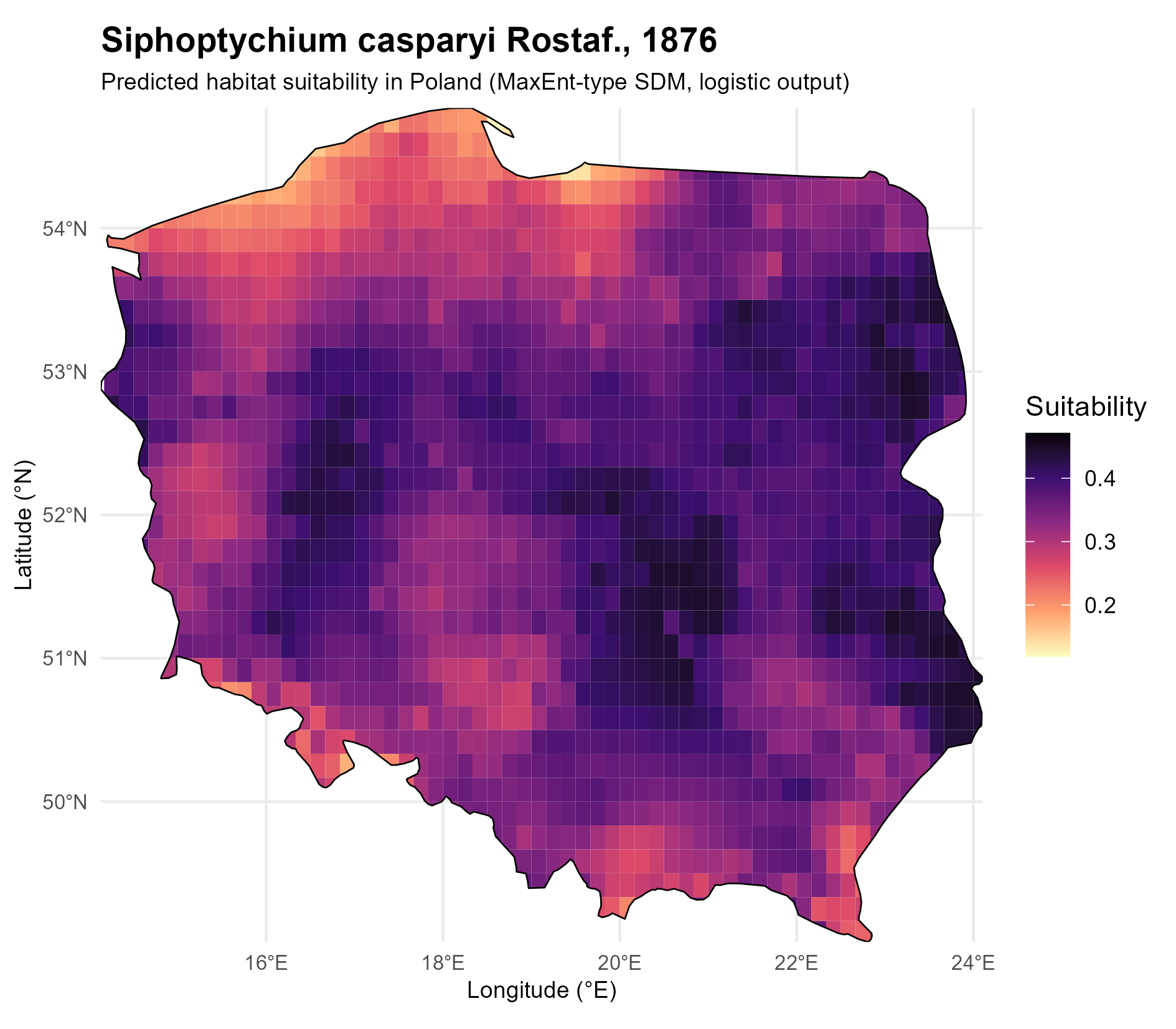

Supplement: Supplemental Information 12 — Set of 101 raster maps showing predicted potential distributions in Poland for modelled candidate species. Each figure displays continuous climatic suitability and the subset of grid cells exceeding a 10th-percentile training presence threshold. [file peerj-14-21492-s012.zip › Figure_SDM_poland_rank074_Siphoptychium_casparyi_Rostaf_1876_MaxEnt_logistic.png]

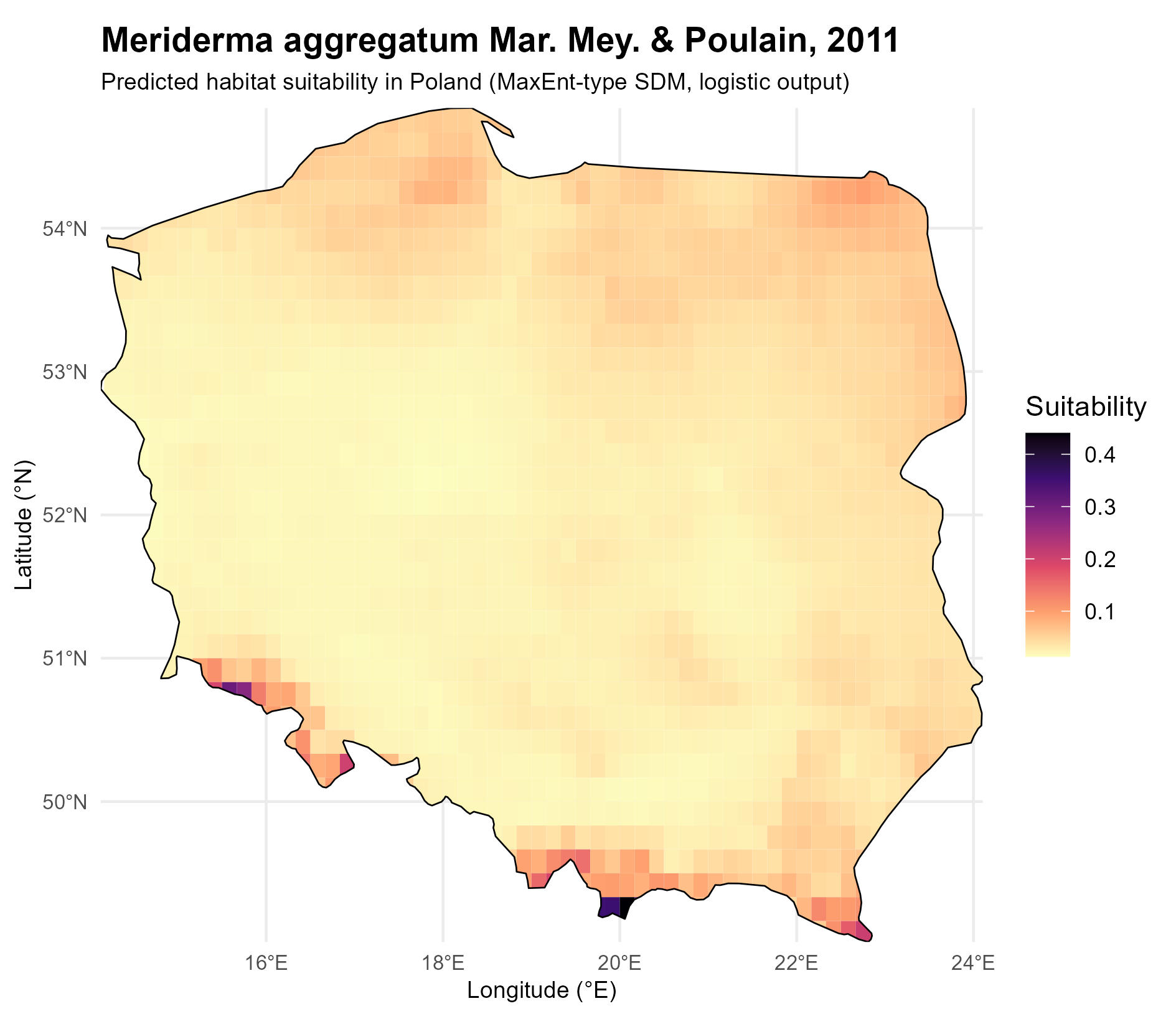

Supplement: Supplemental Information 12 — Set of 101 raster maps showing predicted potential distributions in Poland for modelled candidate species. Each figure displays continuous climatic suitability and the subset of grid cells exceeding a 10th-percentile training presence threshold. [file peerj-14-21492-s012.zip › Figure_SDM_poland_rank073_Meriderma_aggregatum_Mar_Mey_Poulain_2011_MaxEnt_logistic.png]

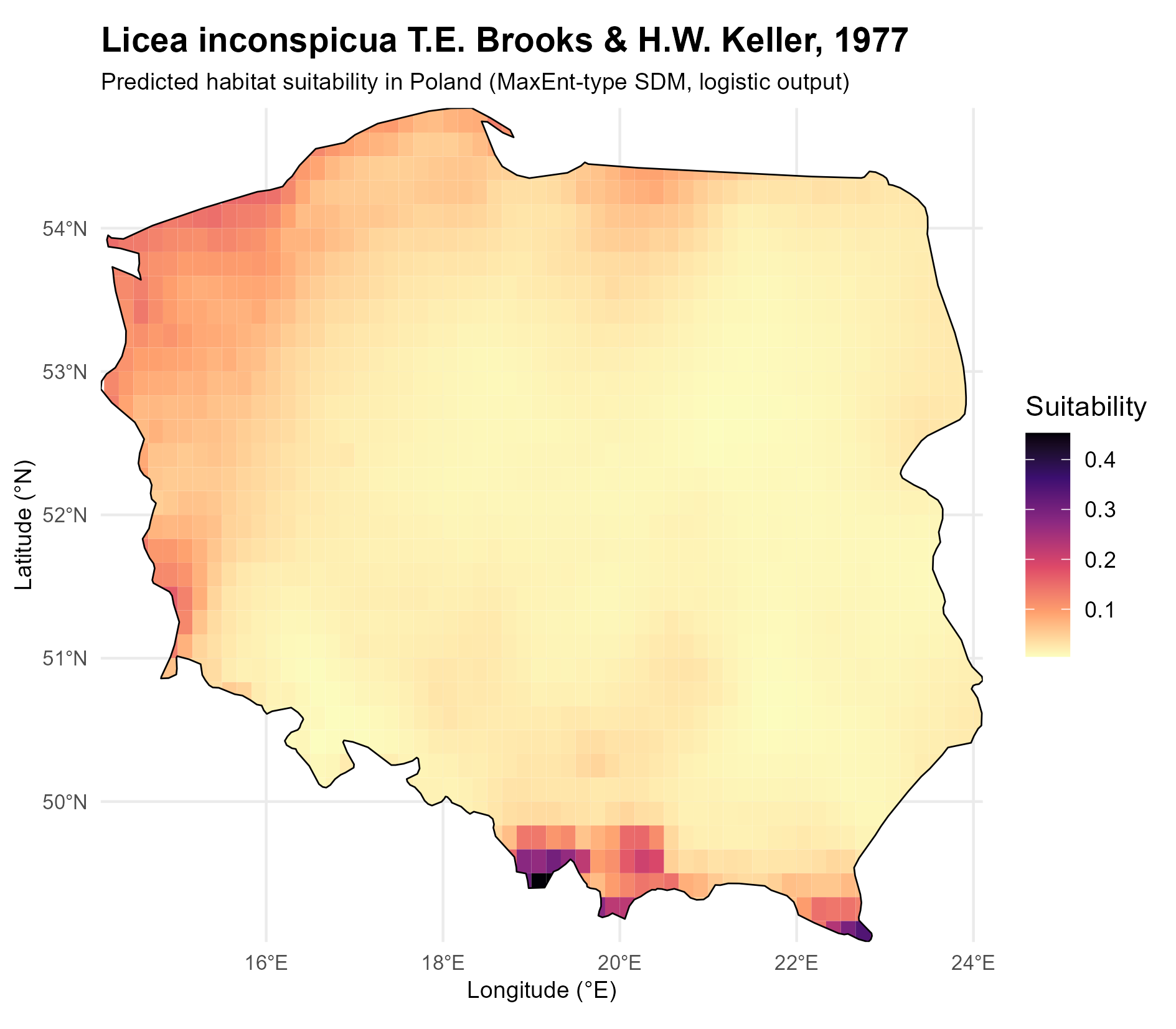

Supplement: Supplemental Information 12 — Set of 101 raster maps showing predicted potential distributions in Poland for modelled candidate species. Each figure displays continuous climatic suitability and the subset of grid cells exceeding a 10th-percentile training presence threshold. [file peerj-14-21492-s012.zip › Figure_SDM_poland_rank072_Licea_inconspicua_T_E_Brooks_H_W_Keller_1977_MaxEnt_logistic.png]

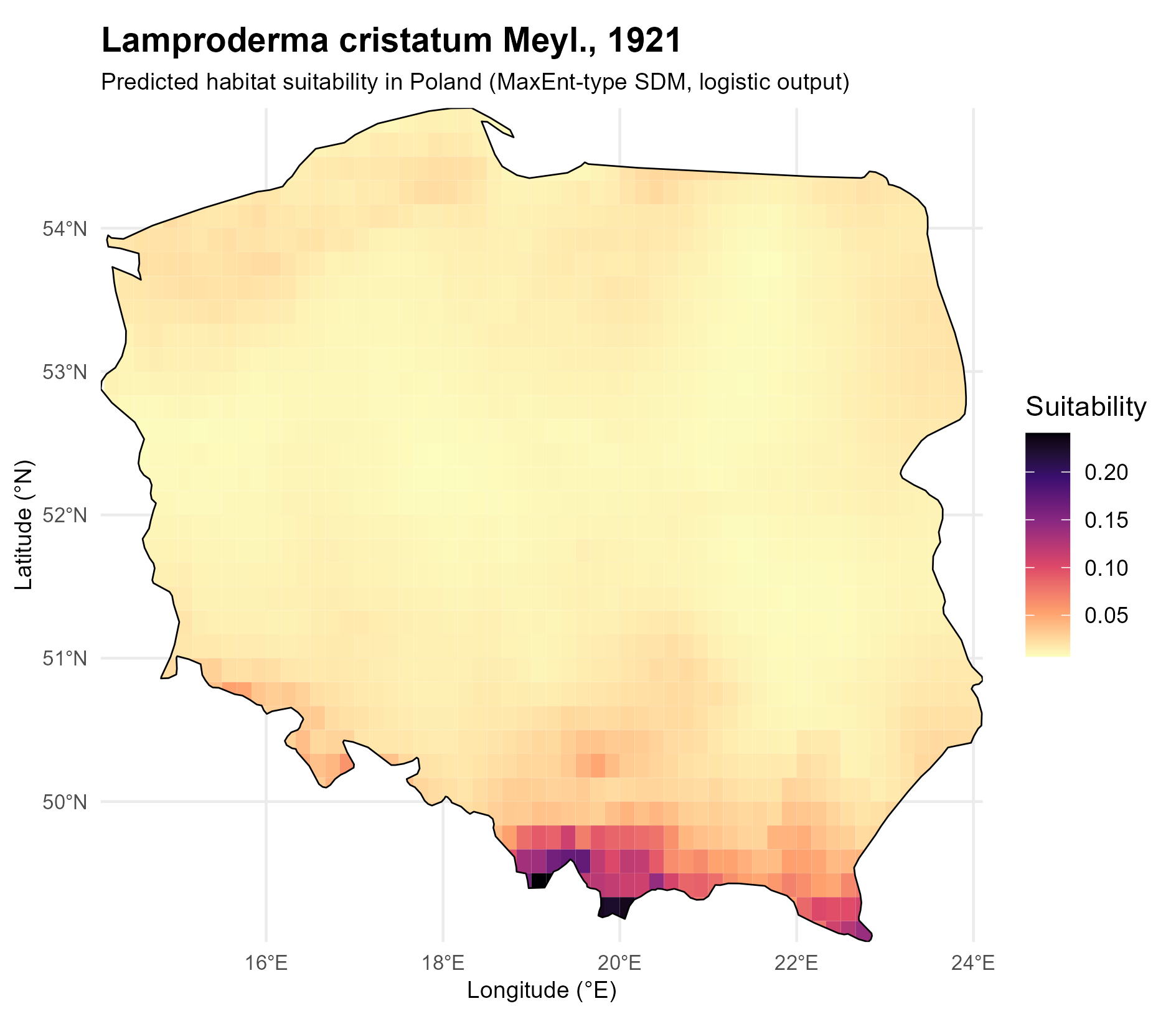

Supplement: Supplemental Information 12 — Set of 101 raster maps showing predicted potential distributions in Poland for modelled candidate species. Each figure displays continuous climatic suitability and the subset of grid cells exceeding a 10th-percentile training presence threshold. [file peerj-14-21492-s012.zip › Figure_SDM_poland_rank071_Lamproderma_cristatum_Meyl_1921_MaxEnt_logistic.png]

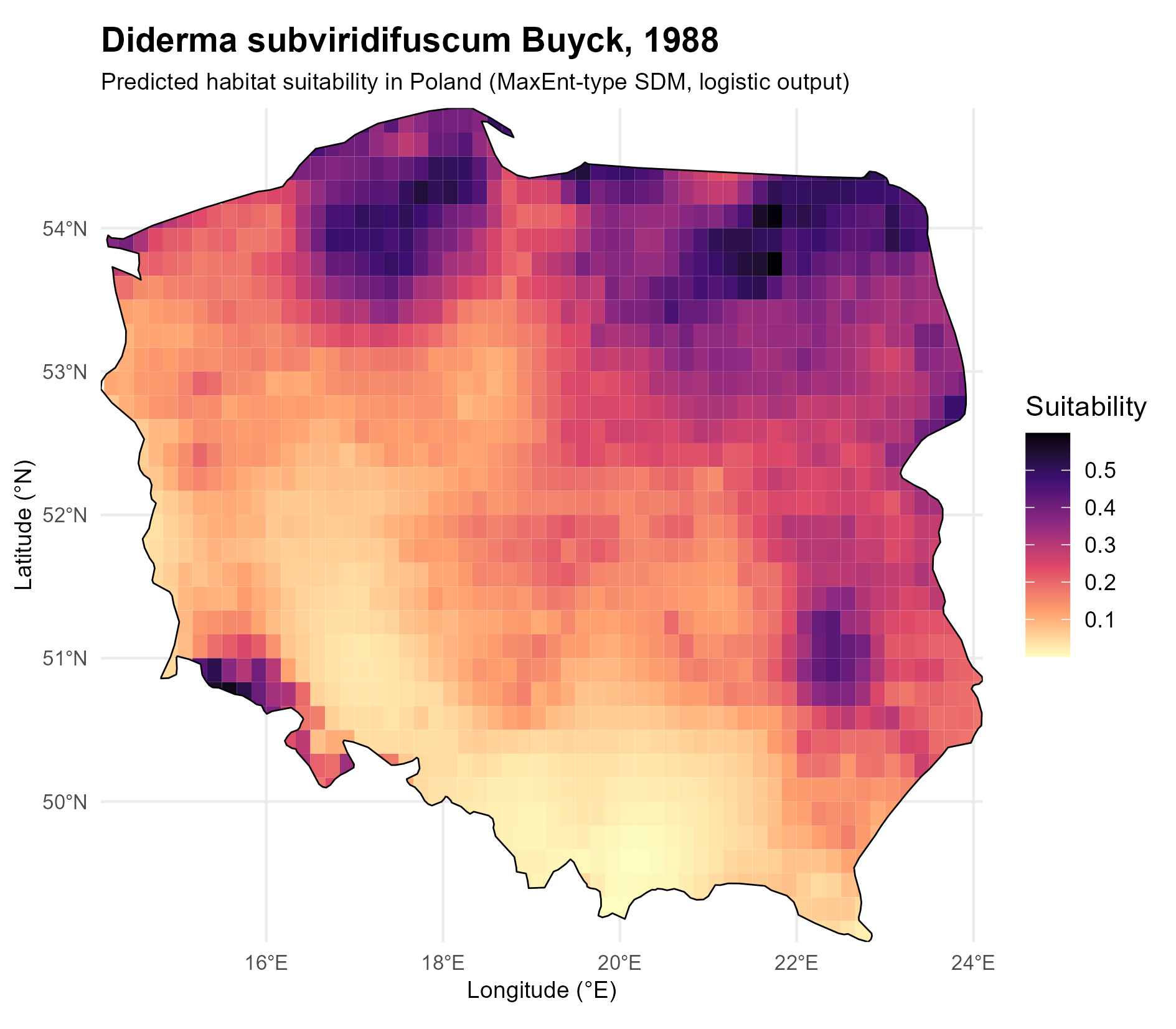

Supplement: Supplemental Information 12 — Set of 101 raster maps showing predicted potential distributions in Poland for modelled candidate species. Each figure displays continuous climatic suitability and the subset of grid cells exceeding a 10th-percentile training presence threshold. [file peerj-14-21492-s012.zip › Figure_SDM_poland_rank070_Diderma_subviridifuscum_Buyck_1988_MaxEnt_logistic.png]

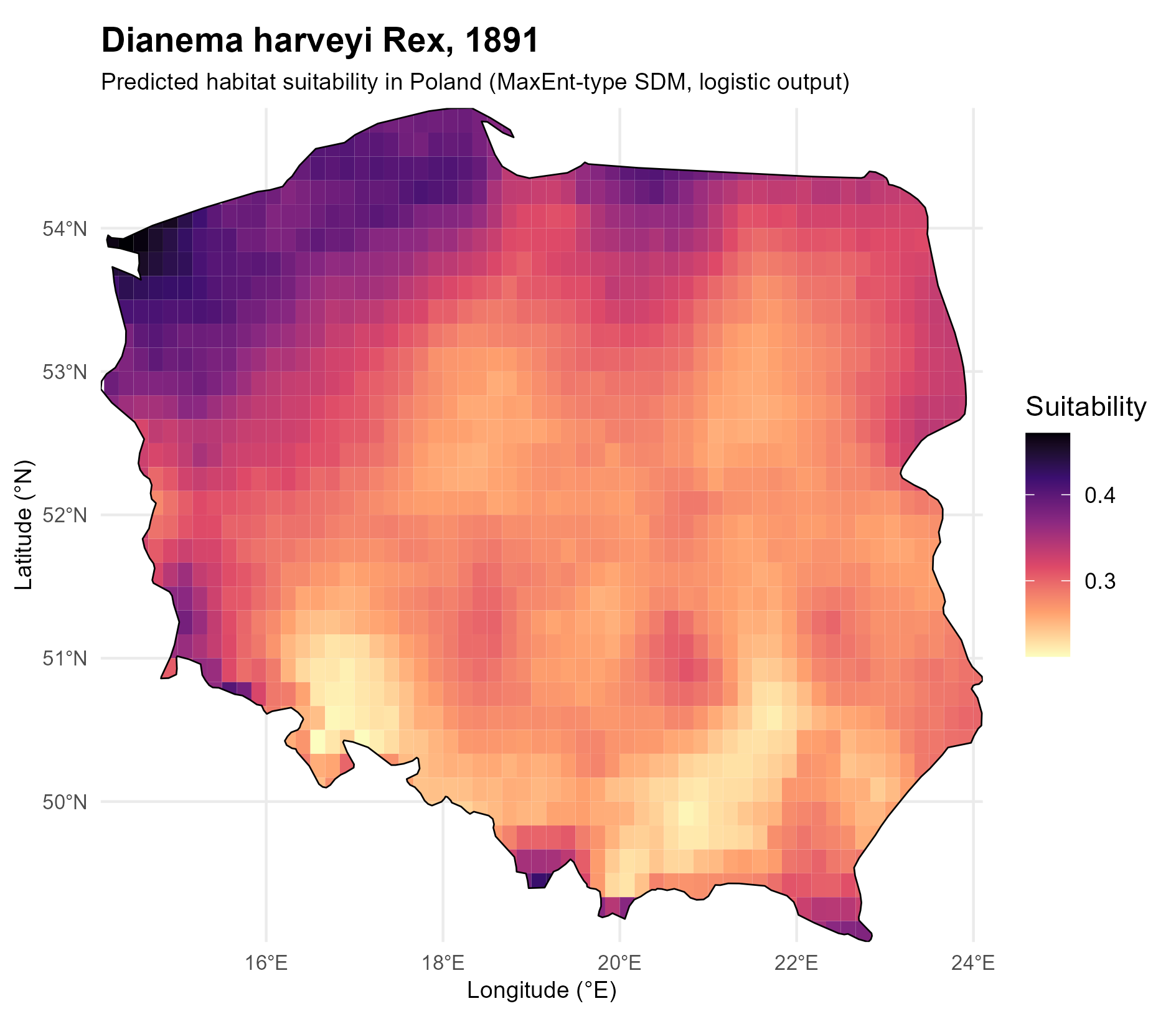

Supplement: Supplemental Information 12 — Set of 101 raster maps showing predicted potential distributions in Poland for modelled candidate species. Each figure displays continuous climatic suitability and the subset of grid cells exceeding a 10th-percentile training presence threshold. [file peerj-14-21492-s012.zip › Figure_SDM_poland_rank069_Dianema_harveyi_Rex_1891_MaxEnt_logistic.png]

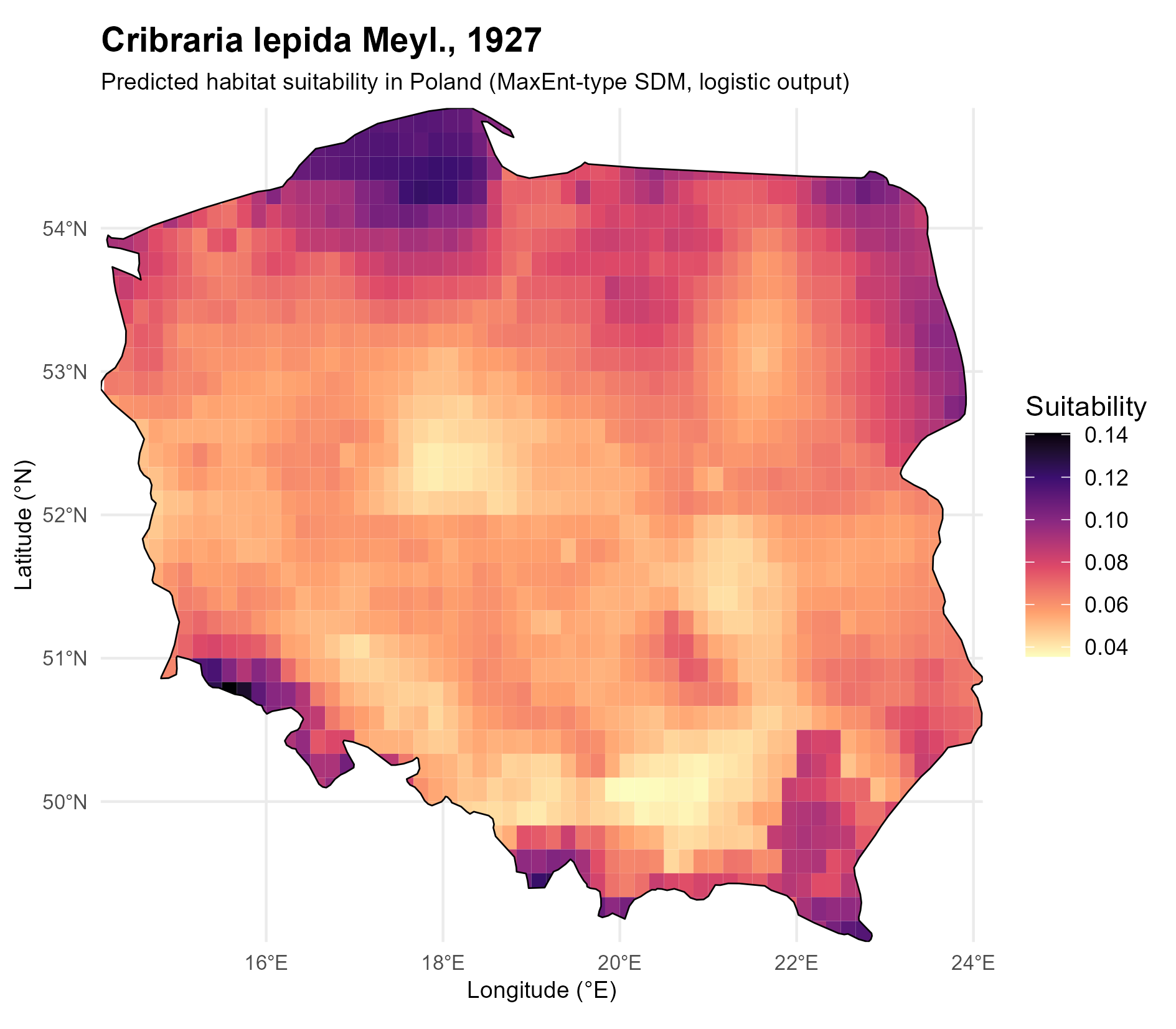

Supplement: Supplemental Information 12 — Set of 101 raster maps showing predicted potential distributions in Poland for modelled candidate species. Each figure displays continuous climatic suitability and the subset of grid cells exceeding a 10th-percentile training presence threshold. [file peerj-14-21492-s012.zip › Figure_SDM_poland_rank068_Cribraria_lepida_Meyl_1927_MaxEnt_logistic.png]

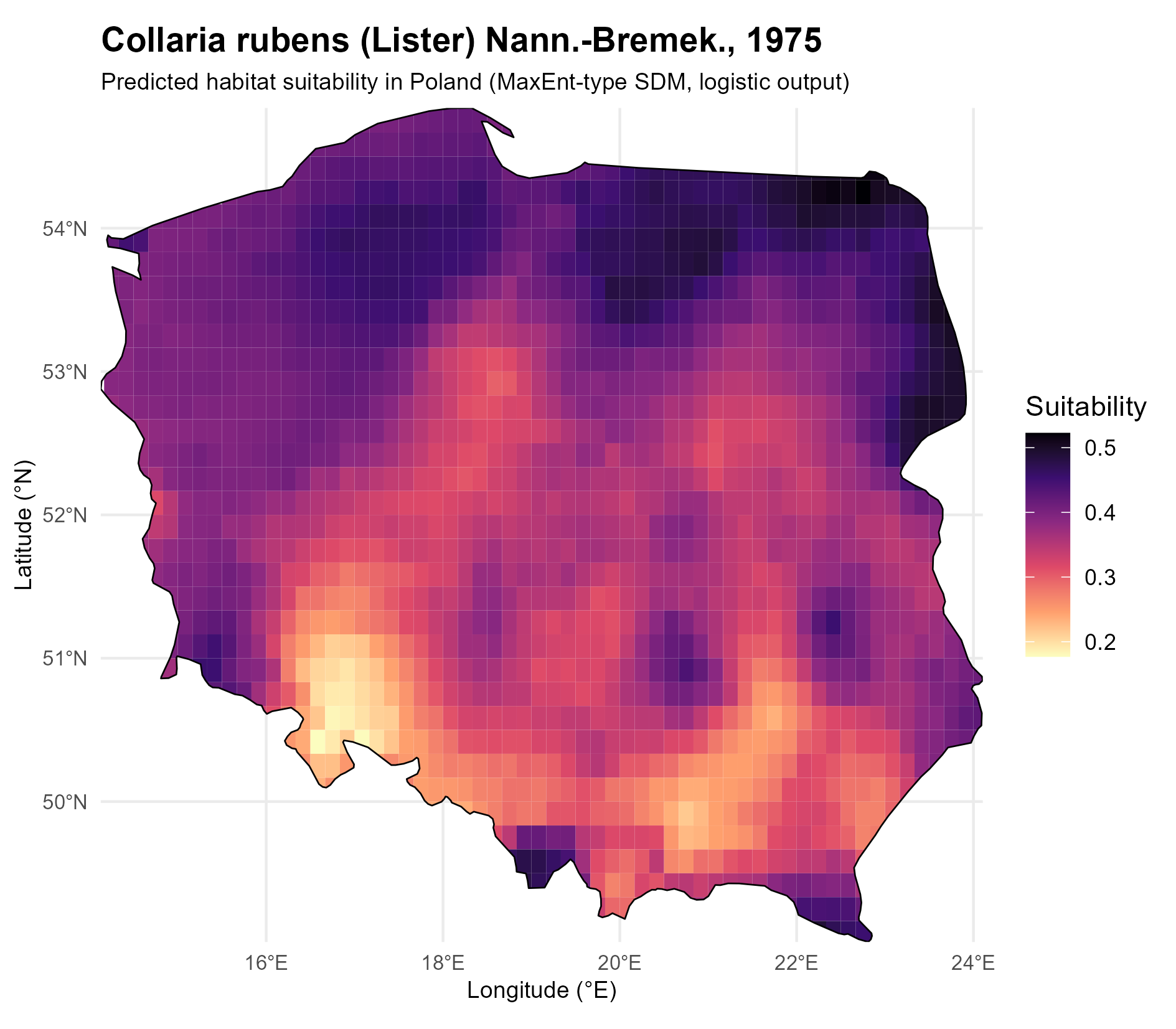

Supplement: Supplemental Information 12 — Set of 101 raster maps showing predicted potential distributions in Poland for modelled candidate species. Each figure displays continuous climatic suitability and the subset of grid cells exceeding a 10th-percentile training presence threshold. [file peerj-14-21492-s012.zip › Figure_SDM_poland_rank067_Collaria_rubens_Lister_Nann_Bremek_1975_MaxEnt_logistic.png]

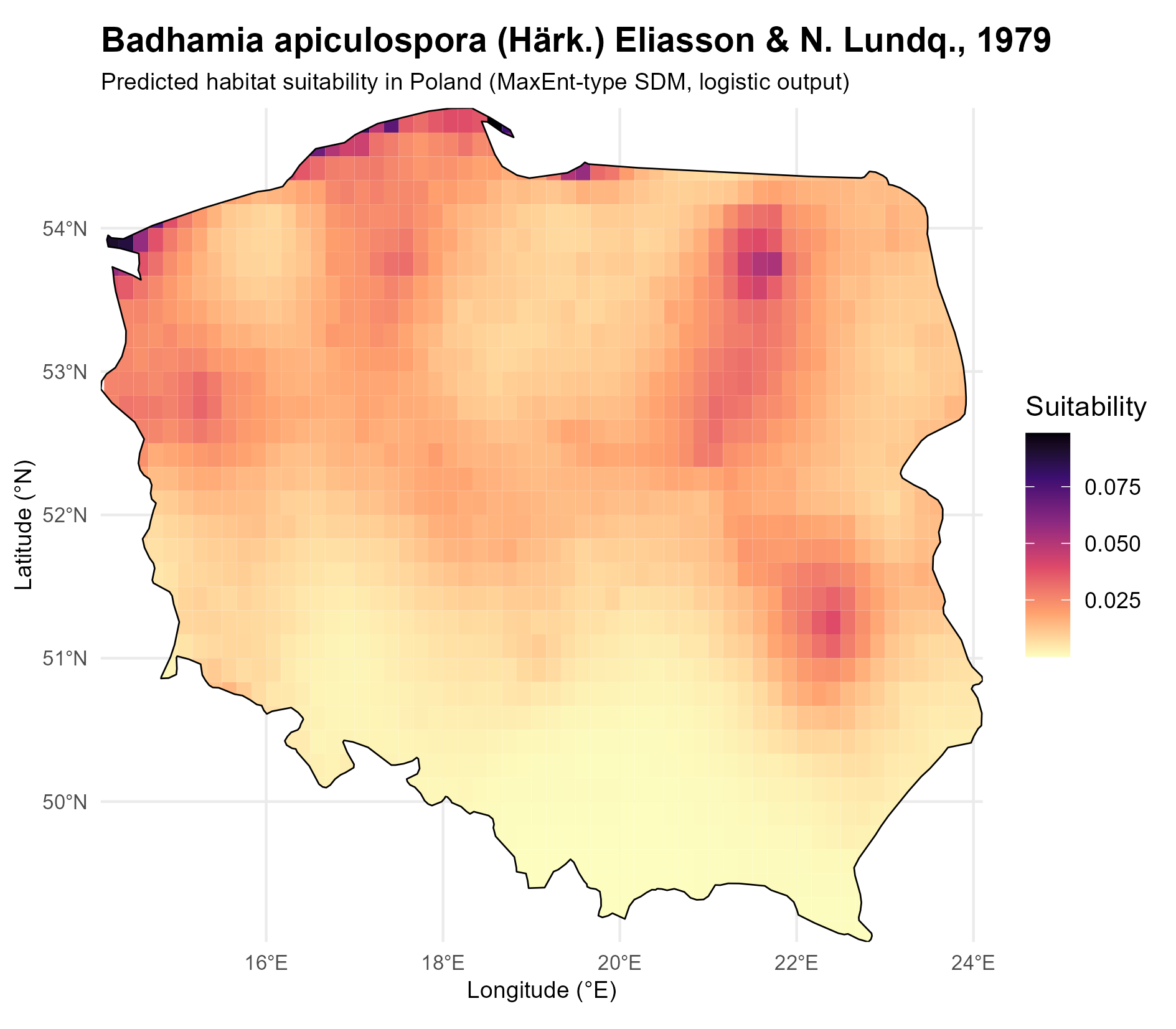

Supplement: Supplemental Information 12 — Set of 101 raster maps showing predicted potential distributions in Poland for modelled candidate species. Each figure displays continuous climatic suitability and the subset of grid cells exceeding a 10th-percentile training presence threshold. [file peerj-14-21492-s012.zip › Figure_SDM_poland_rank066_Badhamia_apiculospora_Hark_Eliasson_N_Lundq_1979_MaxEnt_logistic.png]

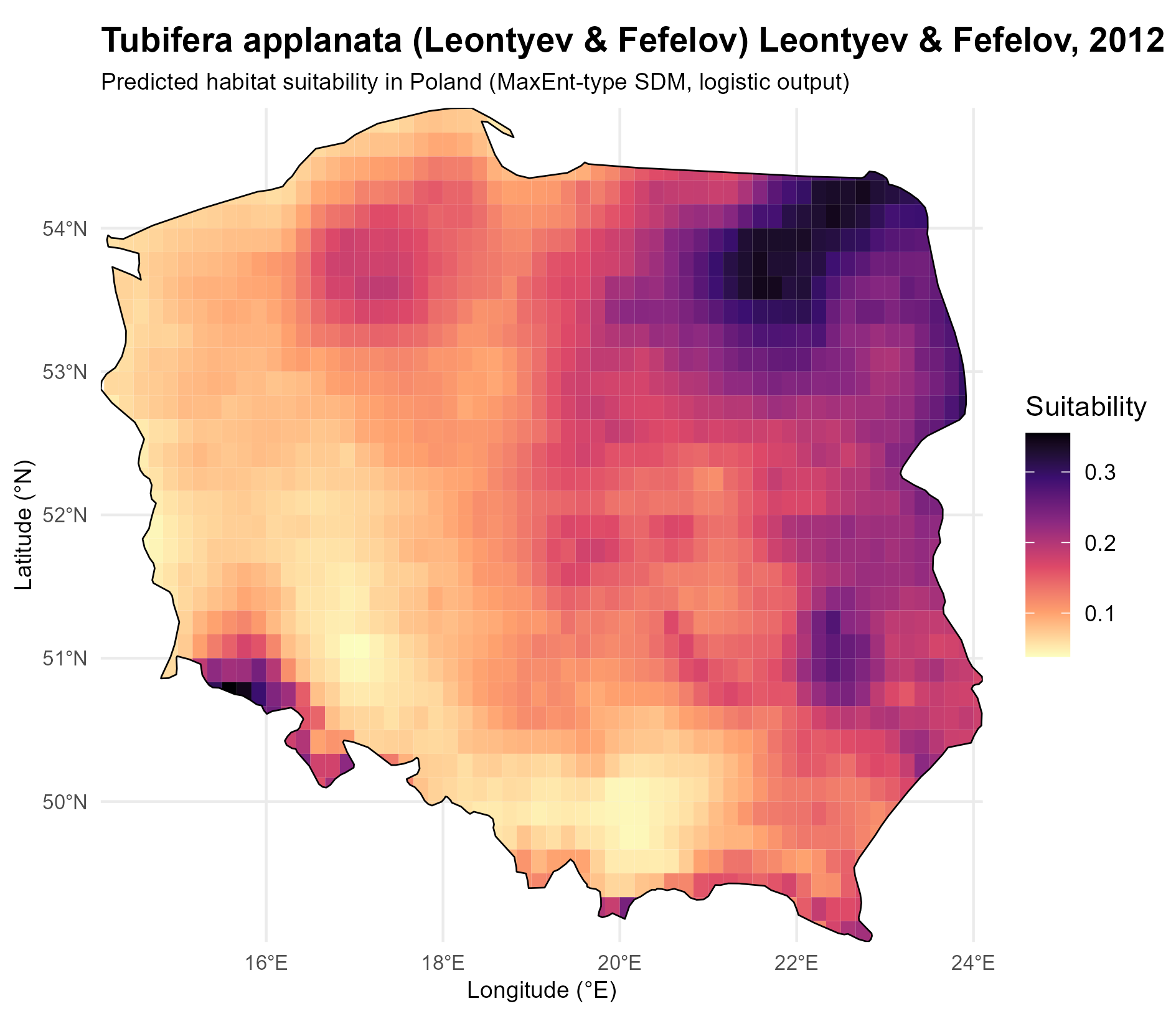

Supplement: Supplemental Information 12 — Set of 101 raster maps showing predicted potential distributions in Poland for modelled candidate species. Each figure displays continuous climatic suitability and the subset of grid cells exceeding a 10th-percentile training presence threshold. [file peerj-14-21492-s012.zip › Figure_SDM_poland_rank065_Tubifera_applanata_Leontyev_Fefelov_Leontyev_Fefelov_2012_MaxEnt_logistic.png]

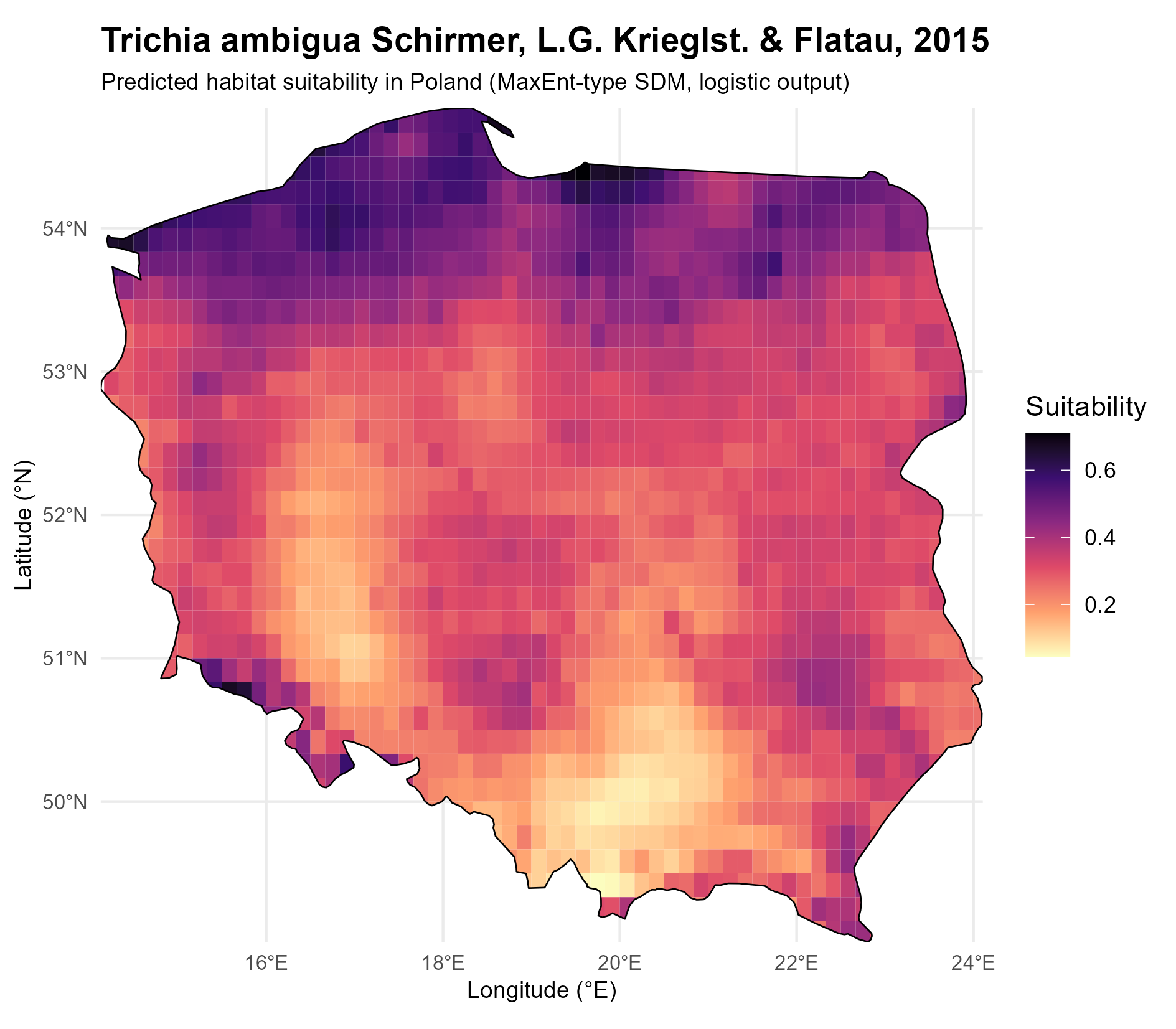

Supplement: Supplemental Information 12 — Set of 101 raster maps showing predicted potential distributions in Poland for modelled candidate species. Each figure displays continuous climatic suitability and the subset of grid cells exceeding a 10th-percentile training presence threshold. [file peerj-14-21492-s012.zip › Figure_SDM_poland_rank064_Trichia_ambigua_Schirmer_L_G_Krieglst_Flatau_2015_MaxEnt_logistic.png]

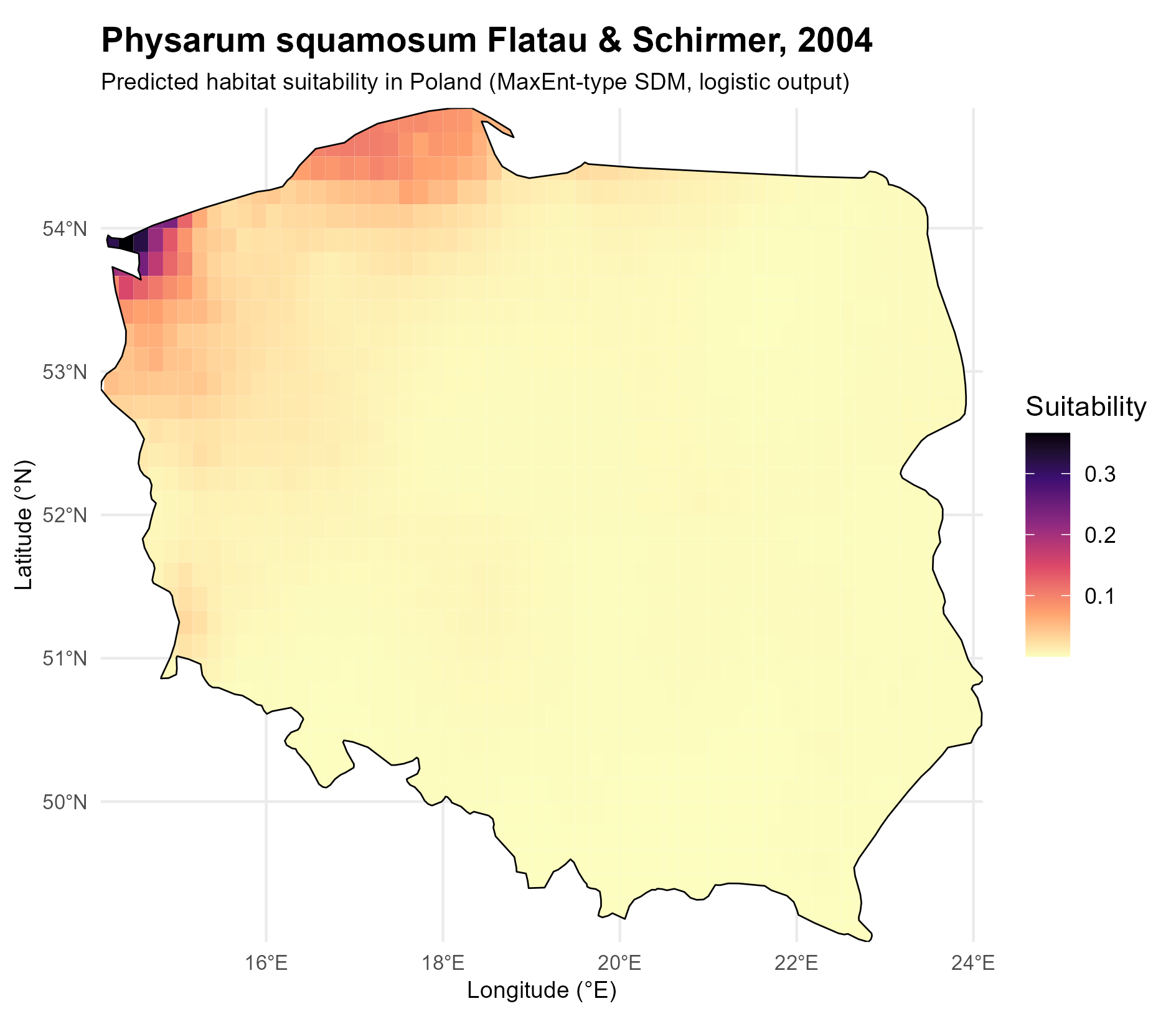

Supplement: Supplemental Information 12 — Set of 101 raster maps showing predicted potential distributions in Poland for modelled candidate species. Each figure displays continuous climatic suitability and the subset of grid cells exceeding a 10th-percentile training presence threshold. [file peerj-14-21492-s012.zip › Figure_SDM_poland_rank063_Physarum_squamosum_Flatau_Schirmer_2004_MaxEnt_logistic.png]

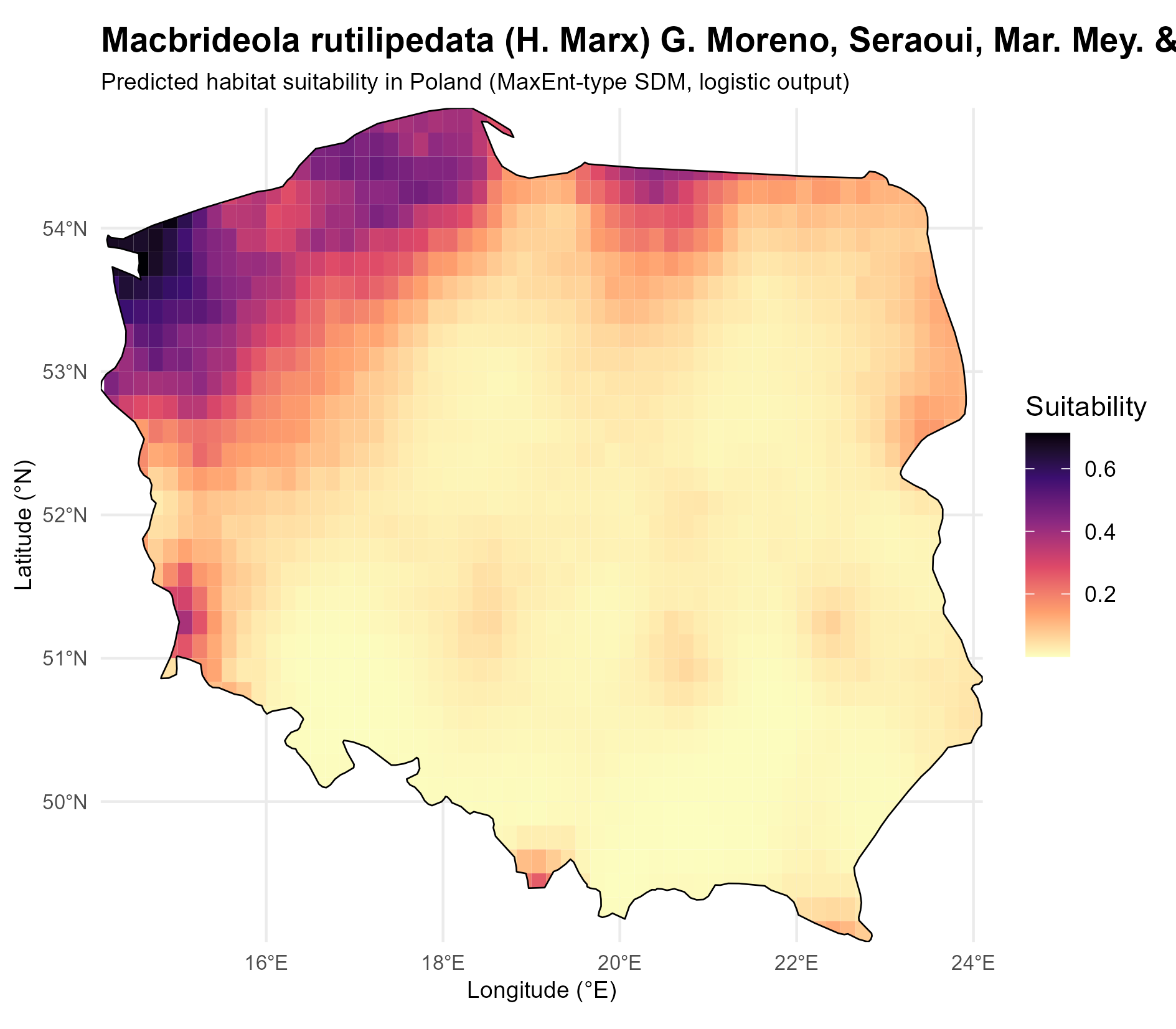

Supplement: Supplemental Information 12 — Set of 101 raster maps showing predicted potential distributions in Poland for modelled candidate species. Each figure displays continuous climatic suitability and the subset of grid cells exceeding a 10th-percentile training presence threshold. [file peerj-14-21492-s012.zip › Figure_SDM_poland_rank062_Macbrideola_rutilipedata_H_Marx_G_Moreno_Seraoui_Mar_Mey_Lopez_Vill_2023_MaxEnt_logistic.png]

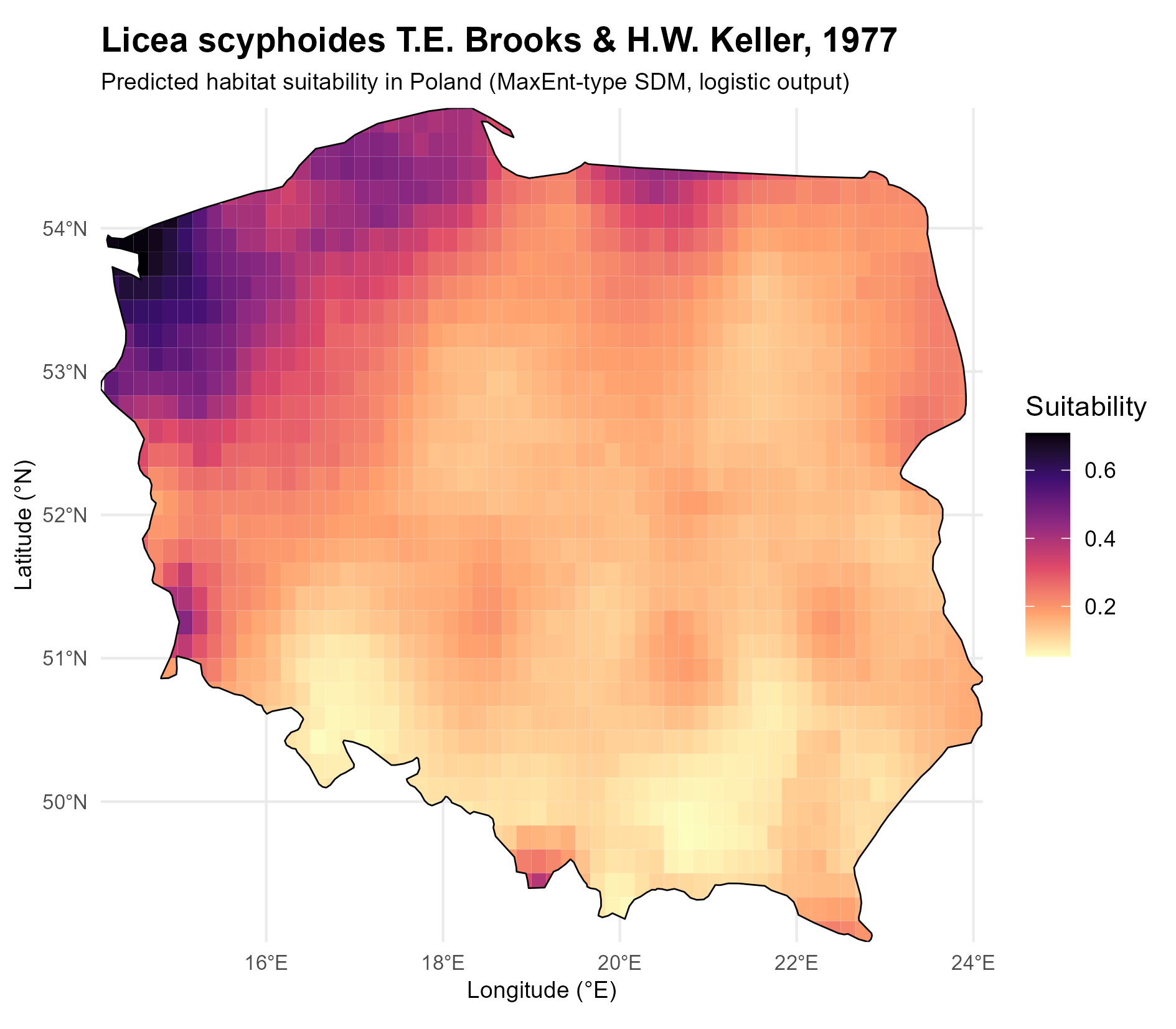

Supplement: Supplemental Information 12 — Set of 101 raster maps showing predicted potential distributions in Poland for modelled candidate species. Each figure displays continuous climatic suitability and the subset of grid cells exceeding a 10th-percentile training presence threshold. [file peerj-14-21492-s012.zip › Figure_SDM_poland_rank061_Licea_scyphoides_T_E_Brooks_H_W_Keller_1977_MaxEnt_logistic.png]

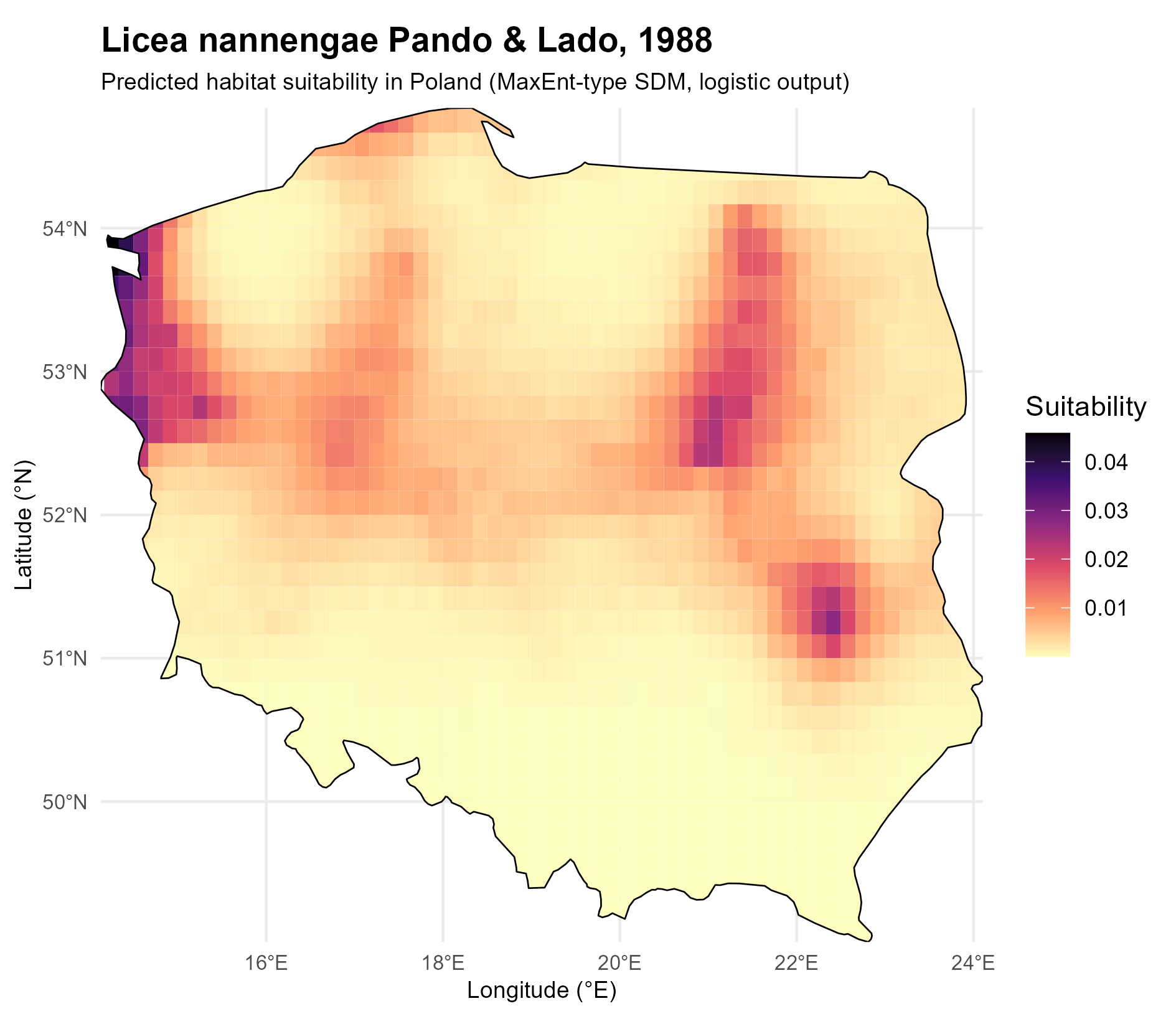

Supplement: Supplemental Information 12 — Set of 101 raster maps showing predicted potential distributions in Poland for modelled candidate species. Each figure displays continuous climatic suitability and the subset of grid cells exceeding a 10th-percentile training presence threshold. [file peerj-14-21492-s012.zip › Figure_SDM_poland_rank060_Licea_nannengae_Pando_Lado_1988_MaxEnt_logistic.png]

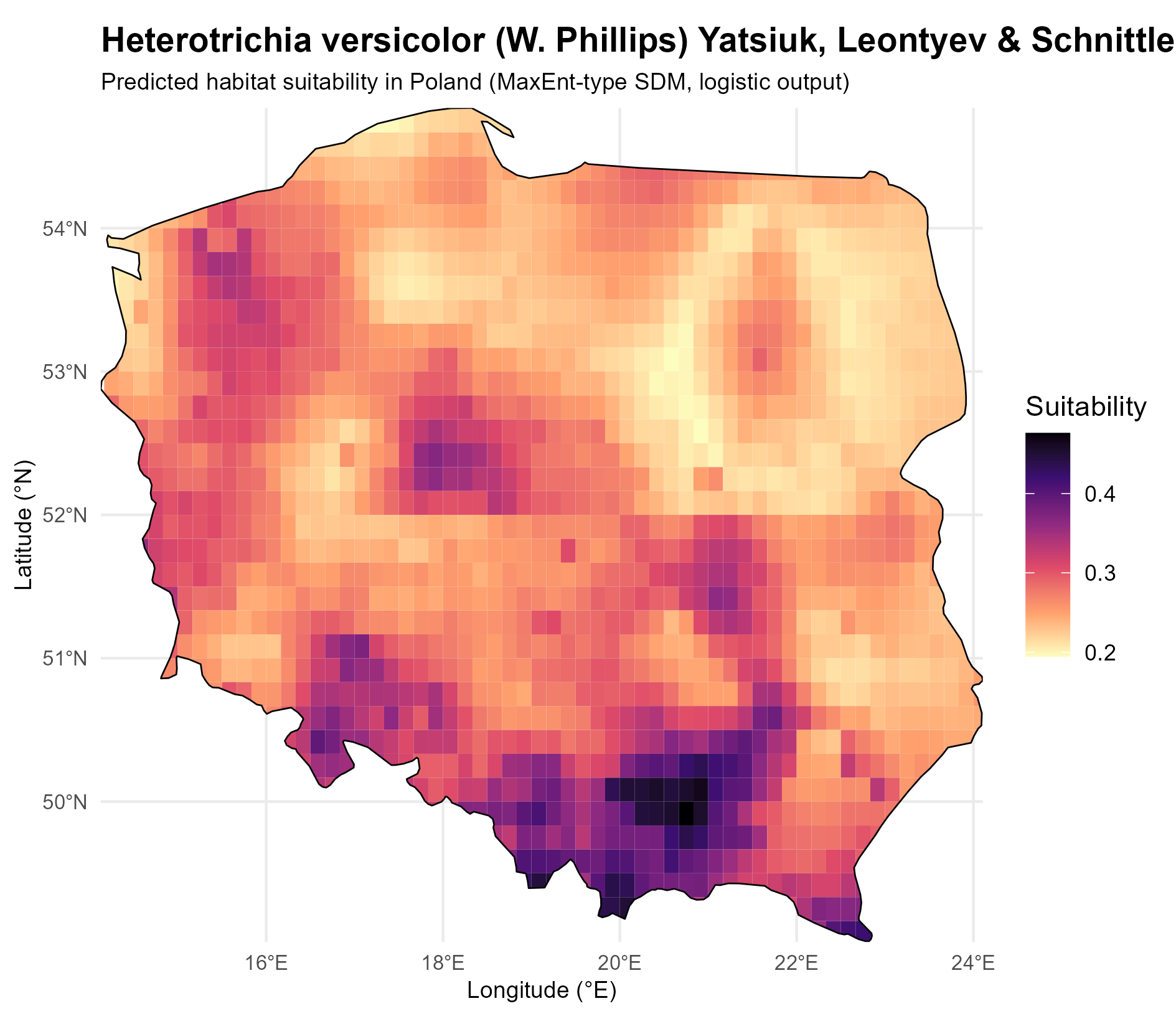

Supplement: Supplemental Information 12 — Set of 101 raster maps showing predicted potential distributions in Poland for modelled candidate species. Each figure displays continuous climatic suitability and the subset of grid cells exceeding a 10th-percentile training presence threshold. [file peerj-14-21492-s012.zip › Figure_SDM_poland_rank059_Heterotrichia_versicolor_W_Phillips_Yatsiuk_Leontyev_Schnittler_2024_MaxEnt_logistic.png]

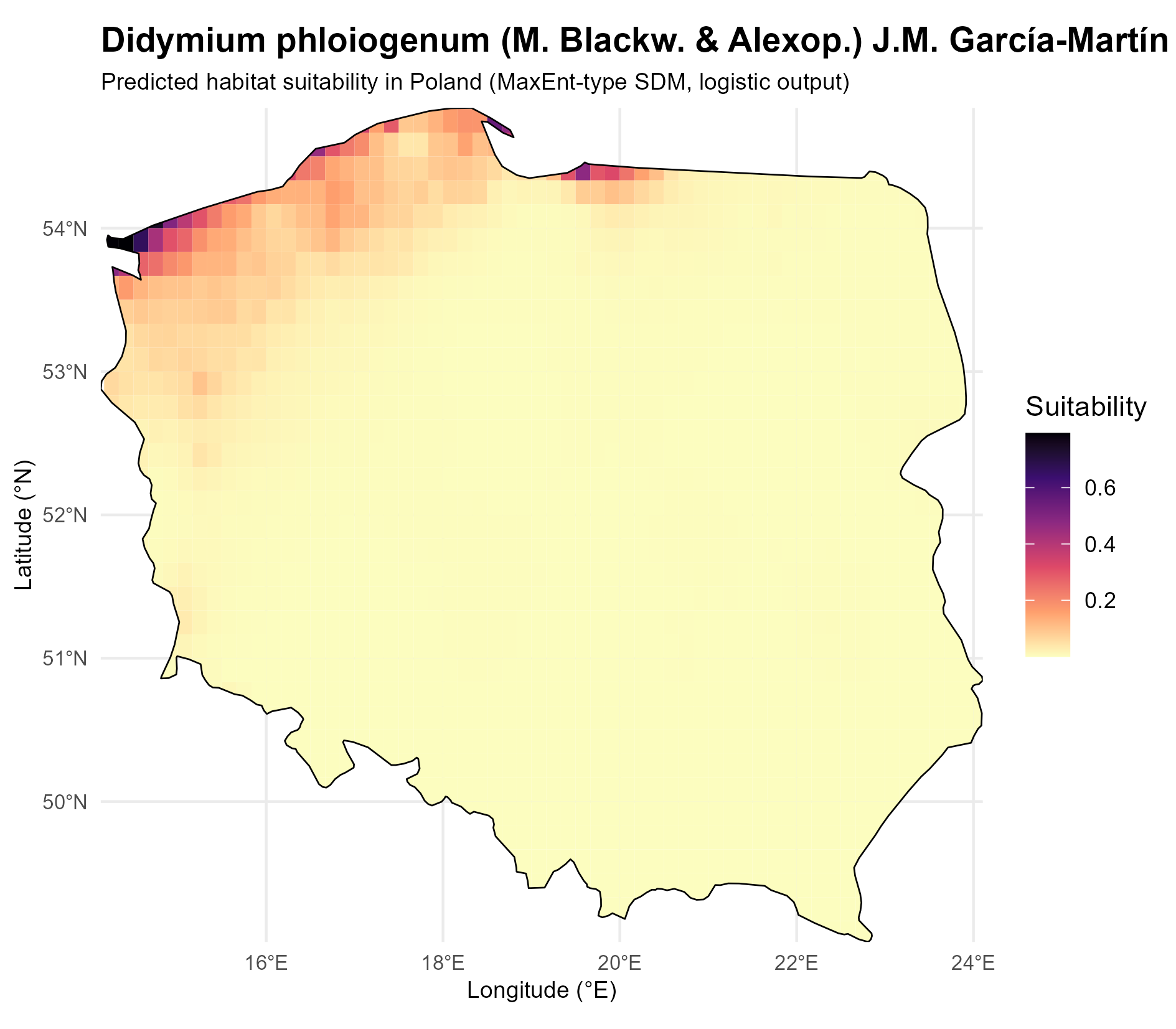

Supplement: Supplemental Information 12 — Set of 101 raster maps showing predicted potential distributions in Poland for modelled candidate species. Each figure displays continuous climatic suitability and the subset of grid cells exceeding a 10th-percentile training presence threshold. [file peerj-14-21492-s012.zip › Figure_SDM_poland_rank058_Didymium_phloiogenum_M_Blackw_Alexop_J_M_Garcia_Martin_Lado_2023_MaxEnt_logistic.png]

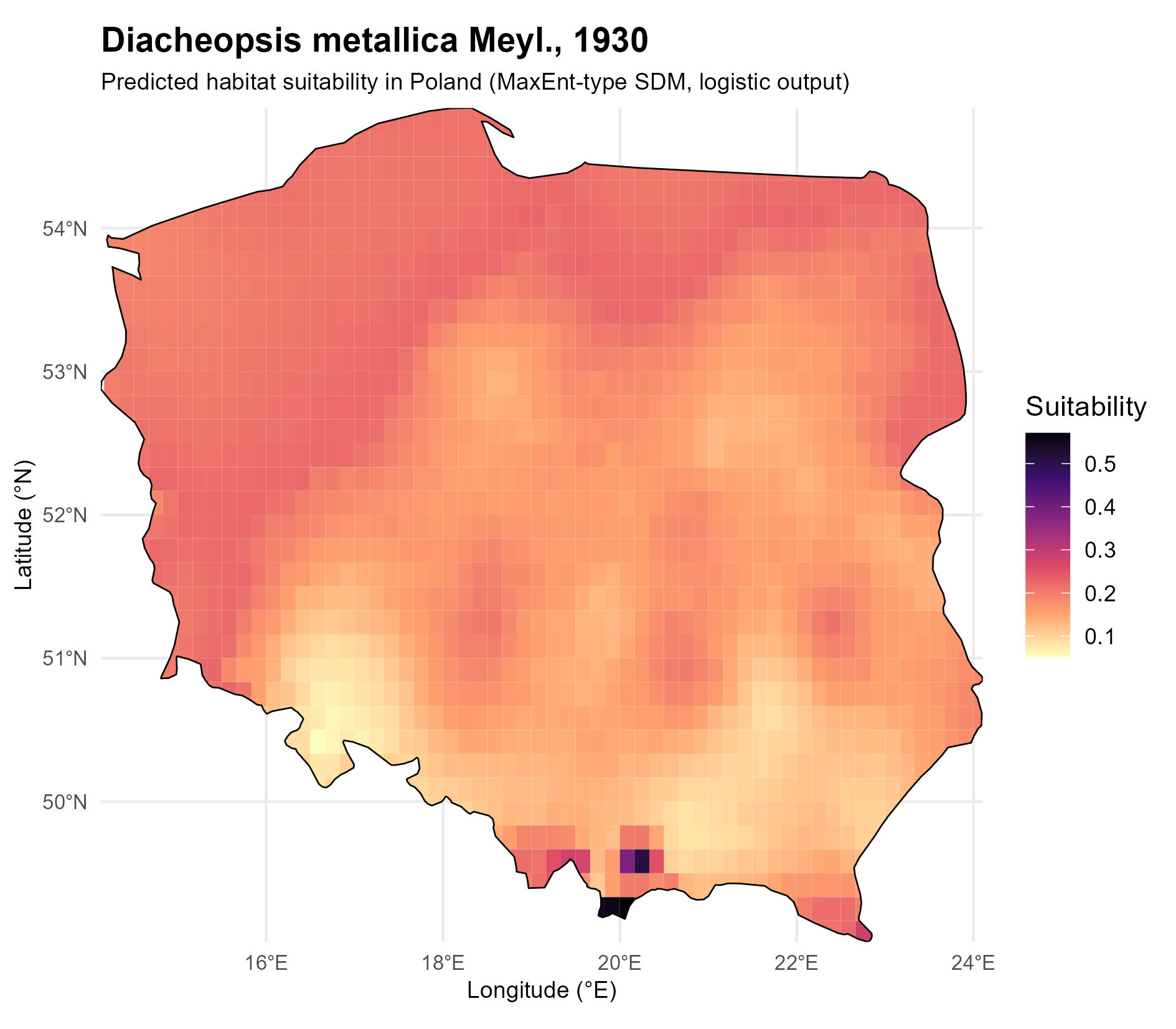

Supplement: Supplemental Information 12 — Set of 101 raster maps showing predicted potential distributions in Poland for modelled candidate species. Each figure displays continuous climatic suitability and the subset of grid cells exceeding a 10th-percentile training presence threshold. [file peerj-14-21492-s012.zip › Figure_SDM_poland_rank057_Diacheopsis_metallica_Meyl_1930_MaxEnt_logistic.png]

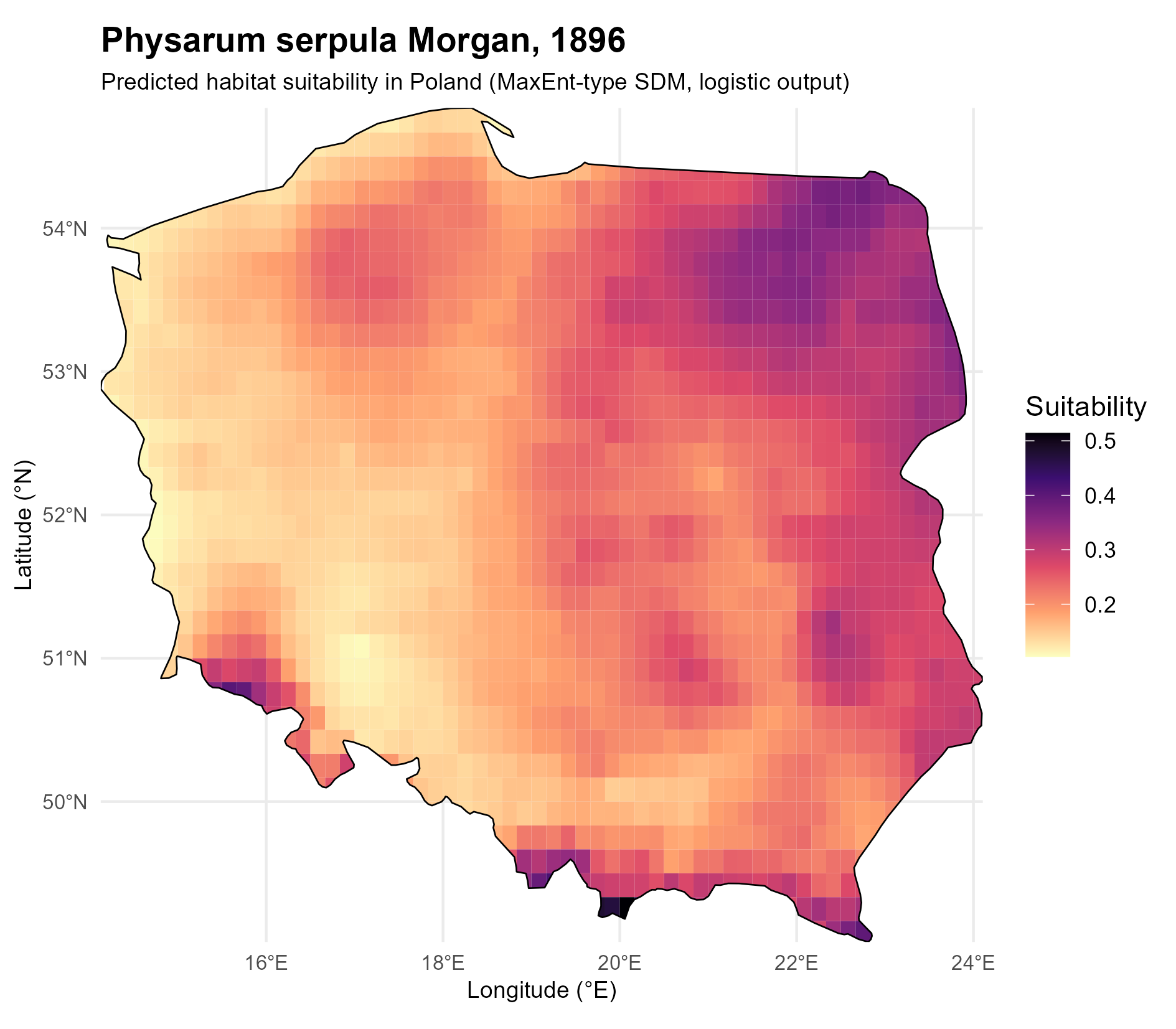

Supplement: Supplemental Information 12 — Set of 101 raster maps showing predicted potential distributions in Poland for modelled candidate species. Each figure displays continuous climatic suitability and the subset of grid cells exceeding a 10th-percentile training presence threshold. [file peerj-14-21492-s012.zip › Figure_SDM_poland_rank056_Physarum_serpula_Morgan_1896_MaxEnt_logistic.png]

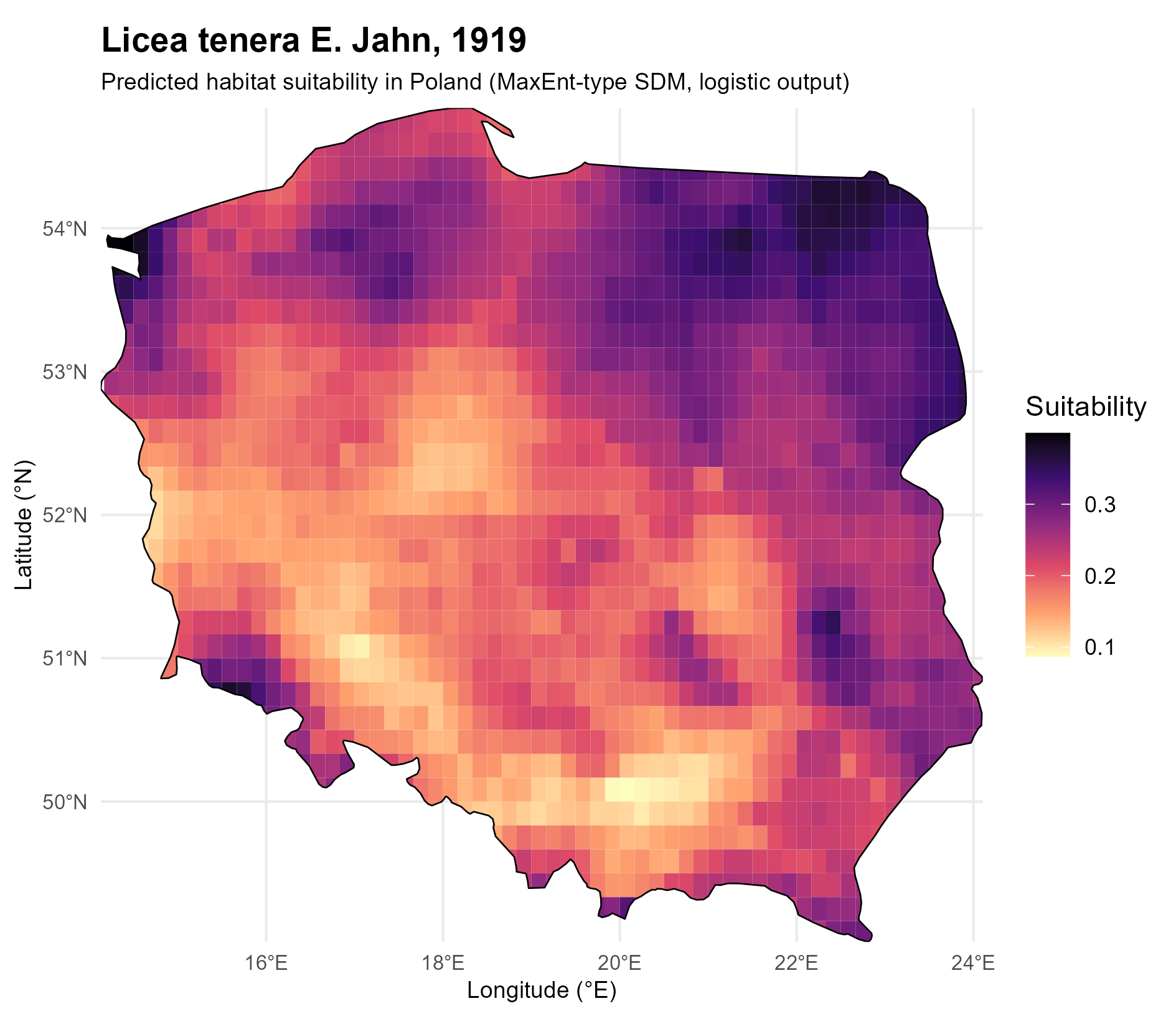

Supplement: Supplemental Information 12 — Set of 101 raster maps showing predicted potential distributions in Poland for modelled candidate species. Each figure displays continuous climatic suitability and the subset of grid cells exceeding a 10th-percentile training presence threshold. [file peerj-14-21492-s012.zip › Figure_SDM_poland_rank055_Licea_tenera_E_Jahn_1919_MaxEnt_logistic.png]

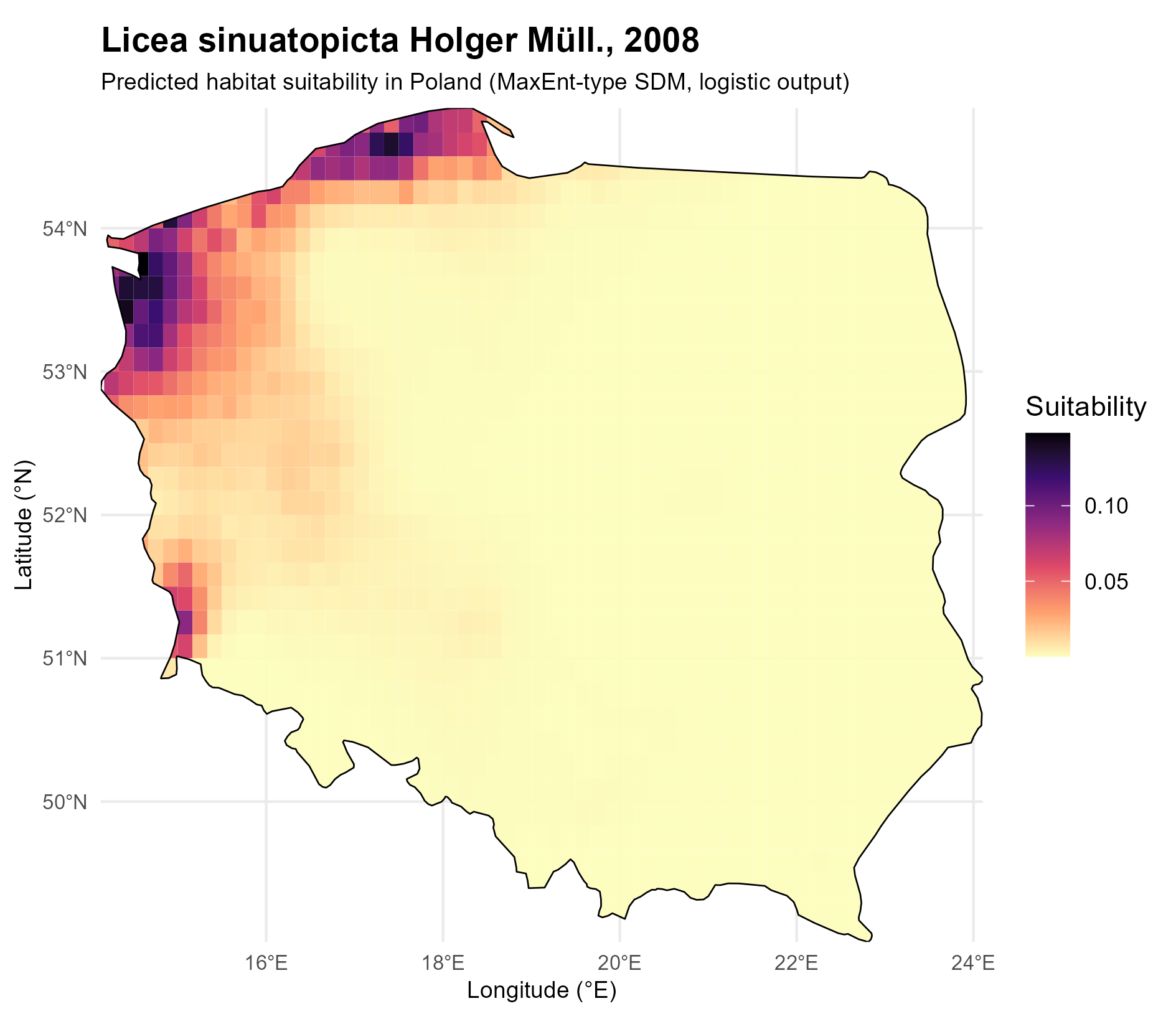

Supplement: Supplemental Information 12 — Set of 101 raster maps showing predicted potential distributions in Poland for modelled candidate species. Each figure displays continuous climatic suitability and the subset of grid cells exceeding a 10th-percentile training presence threshold. [file peerj-14-21492-s012.zip › Figure_SDM_poland_rank054_Licea_sinuatopicta_Holger_Mull_2008_MaxEnt_logistic.png]

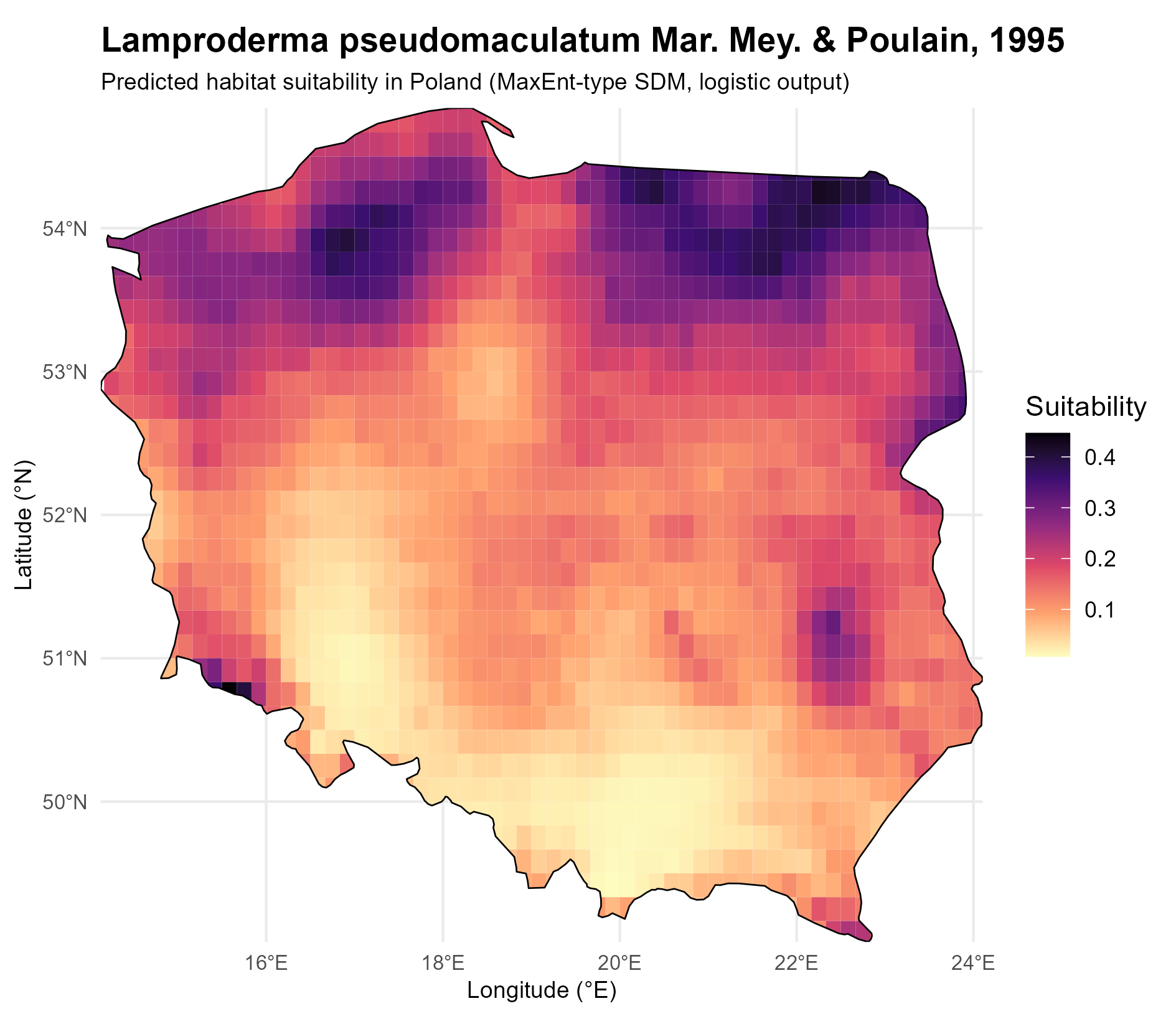

Supplement: Supplemental Information 12 — Set of 101 raster maps showing predicted potential distributions in Poland for modelled candidate species. Each figure displays continuous climatic suitability and the subset of grid cells exceeding a 10th-percentile training presence threshold. [file peerj-14-21492-s012.zip › Figure_SDM_poland_rank053_Lamproderma_pseudomaculatum_Mar_Mey_Poulain_1995_MaxEnt_logistic.png]

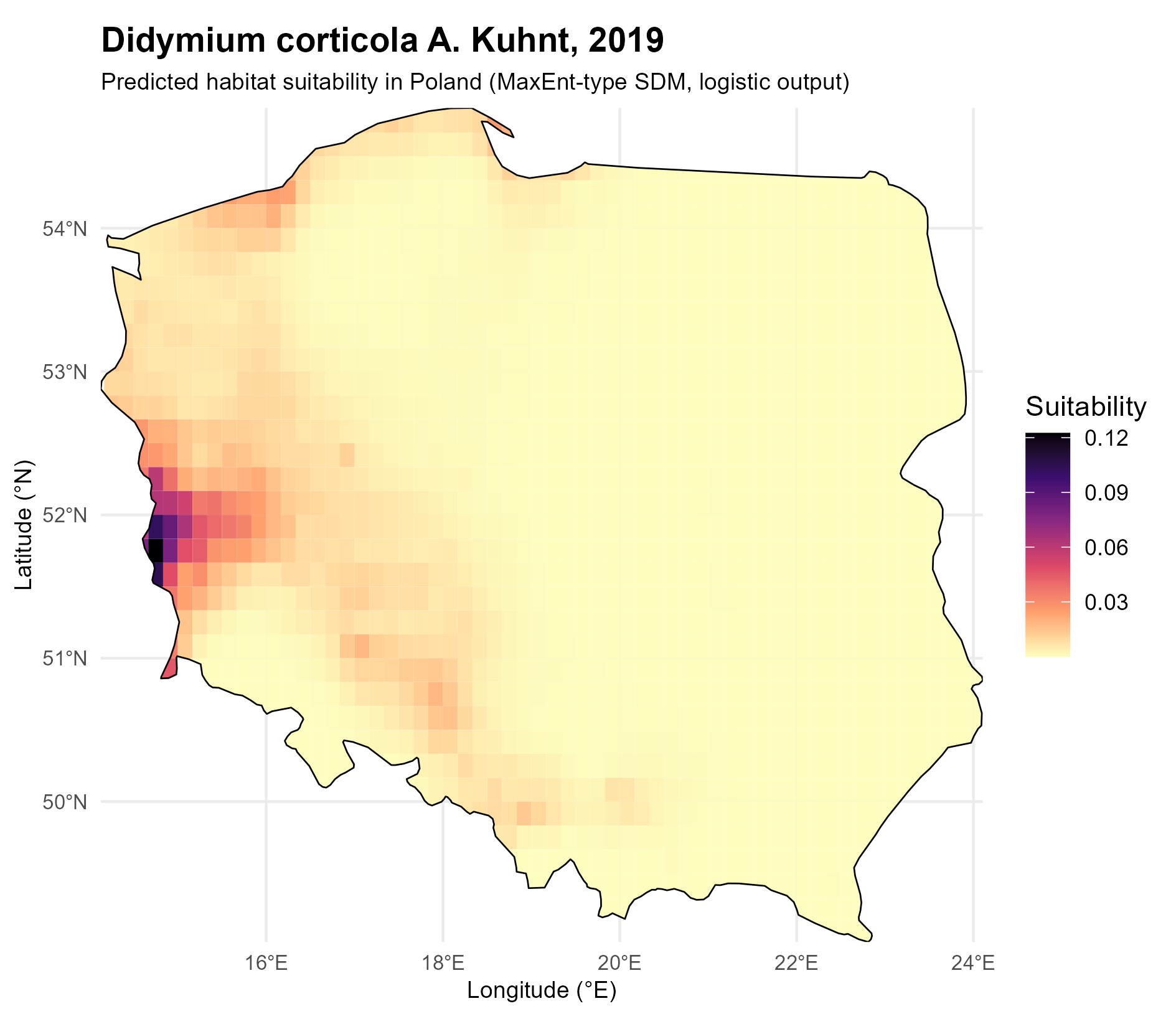

Supplement: Supplemental Information 12 — Set of 101 raster maps showing predicted potential distributions in Poland for modelled candidate species. Each figure displays continuous climatic suitability and the subset of grid cells exceeding a 10th-percentile training presence threshold. [file peerj-14-21492-s012.zip › Figure_SDM_poland_rank052_Didymium_corticola_A_Kuhnt_2019_MaxEnt_logistic.png]

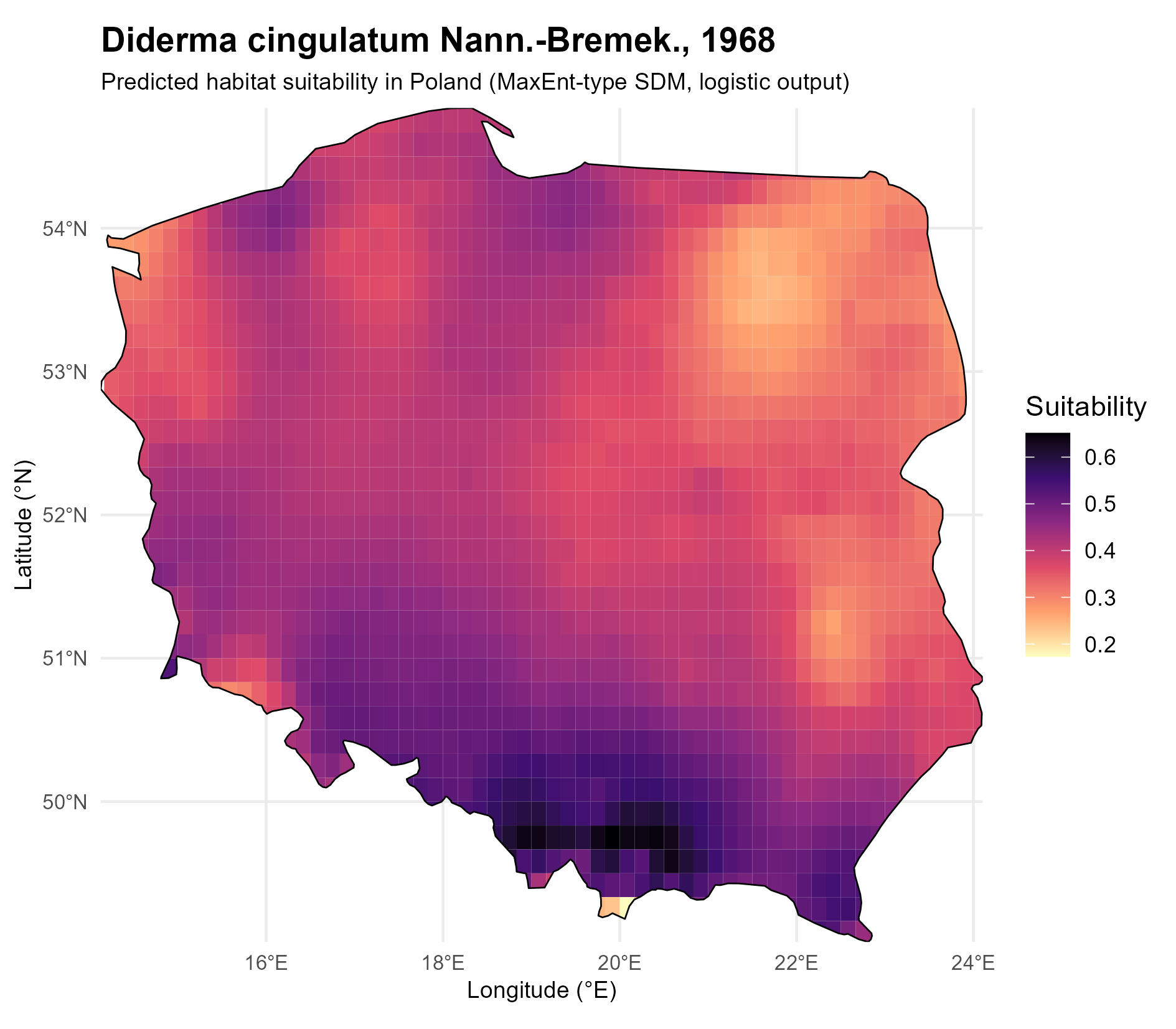

Supplement: Supplemental Information 12 — Set of 101 raster maps showing predicted potential distributions in Poland for modelled candidate species. Each figure displays continuous climatic suitability and the subset of grid cells exceeding a 10th-percentile training presence threshold. [file peerj-14-21492-s012.zip › Figure_SDM_poland_rank051_Diderma_cingulatum_Nann_Bremek_1968_MaxEnt_logistic.png]

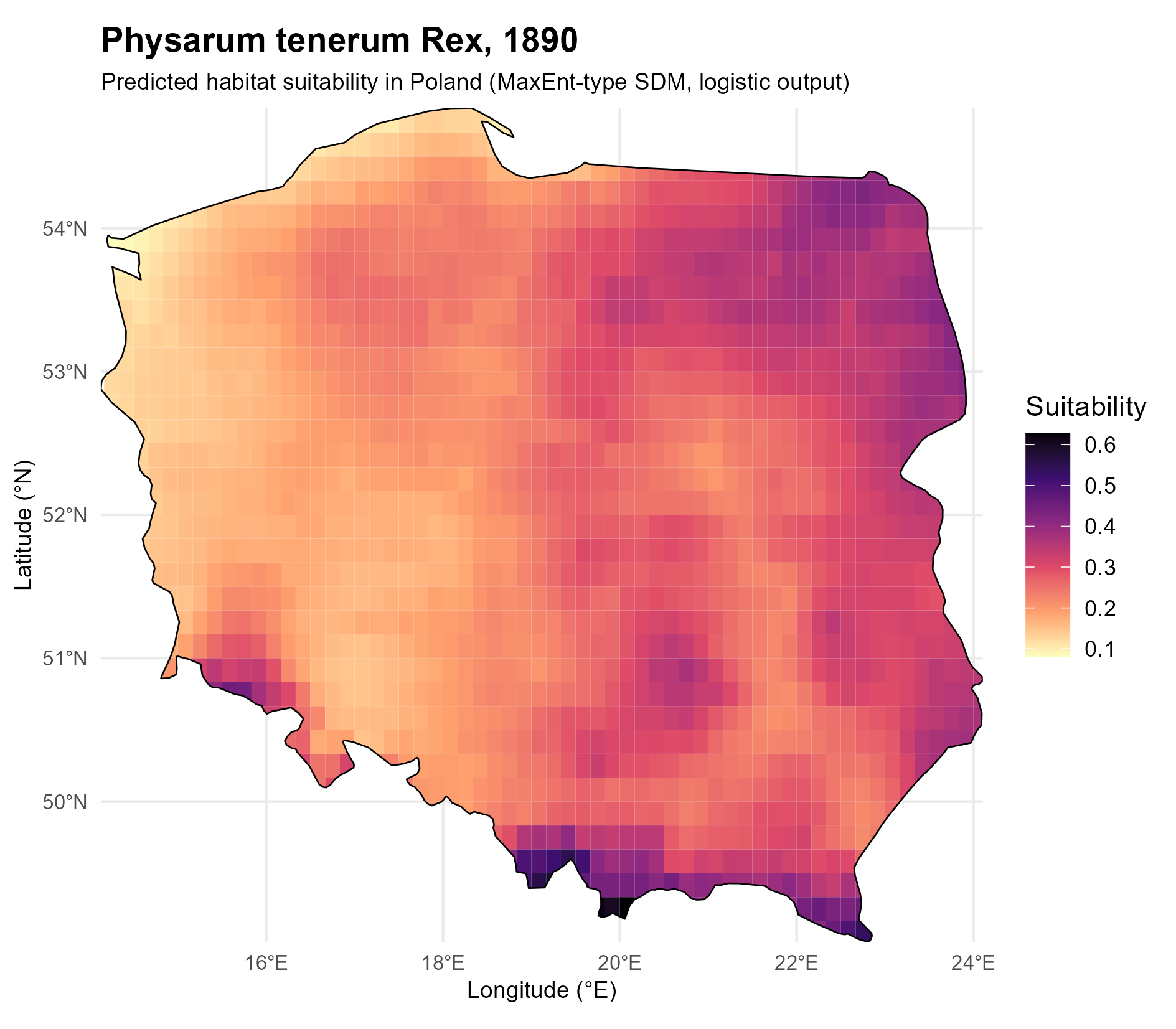

Supplement: Supplemental Information 12 — Set of 101 raster maps showing predicted potential distributions in Poland for modelled candidate species. Each figure displays continuous climatic suitability and the subset of grid cells exceeding a 10th-percentile training presence threshold. [file peerj-14-21492-s012.zip › Figure_SDM_poland_rank050_Physarum_tenerum_Rex_1890_MaxEnt_logistic.png]

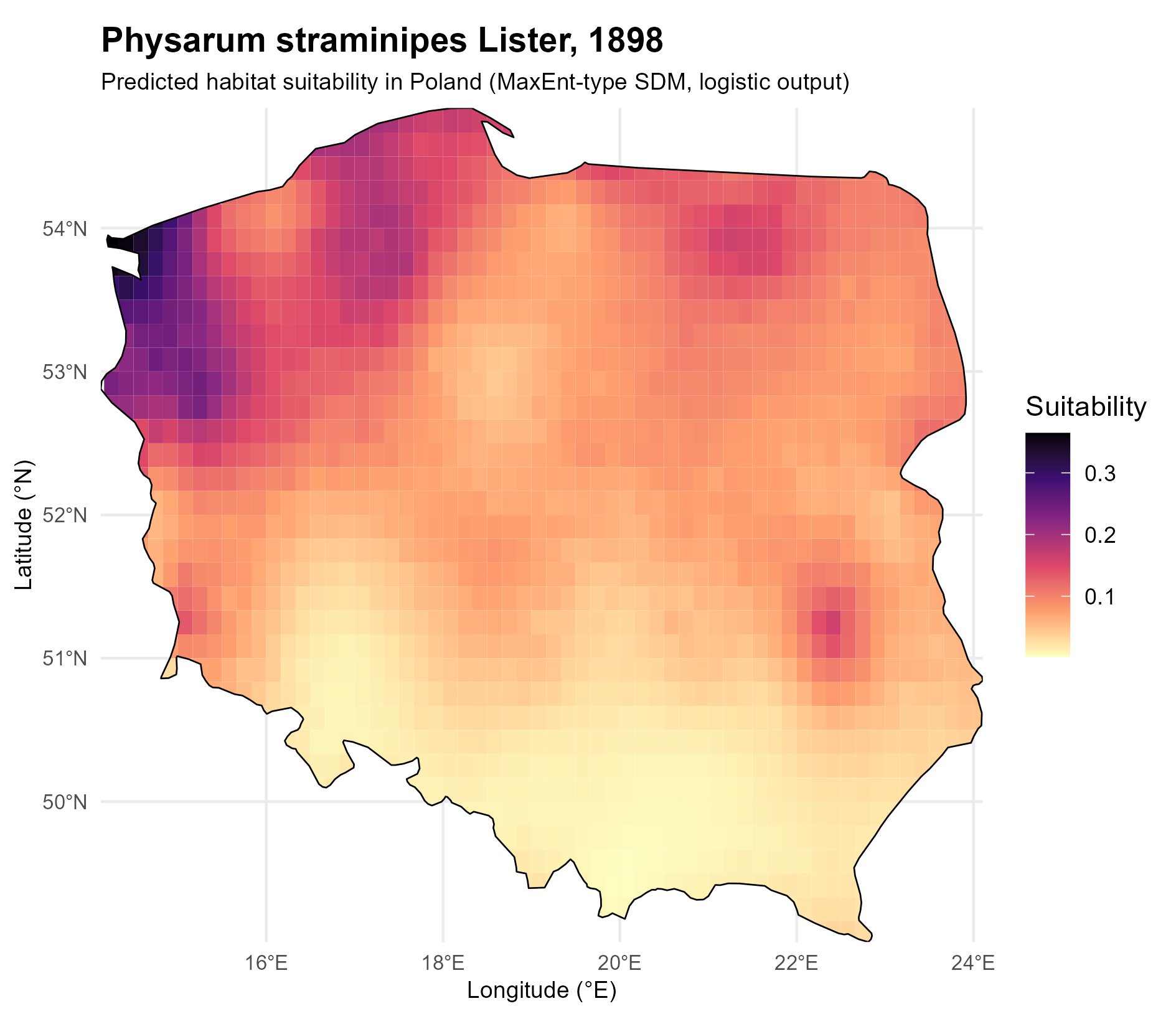

Supplement: Supplemental Information 12 — Set of 101 raster maps showing predicted potential distributions in Poland for modelled candidate species. Each figure displays continuous climatic suitability and the subset of grid cells exceeding a 10th-percentile training presence threshold. [file peerj-14-21492-s012.zip › Figure_SDM_poland_rank049_Physarum_straminipes_Lister_1898_MaxEnt_logistic.png]

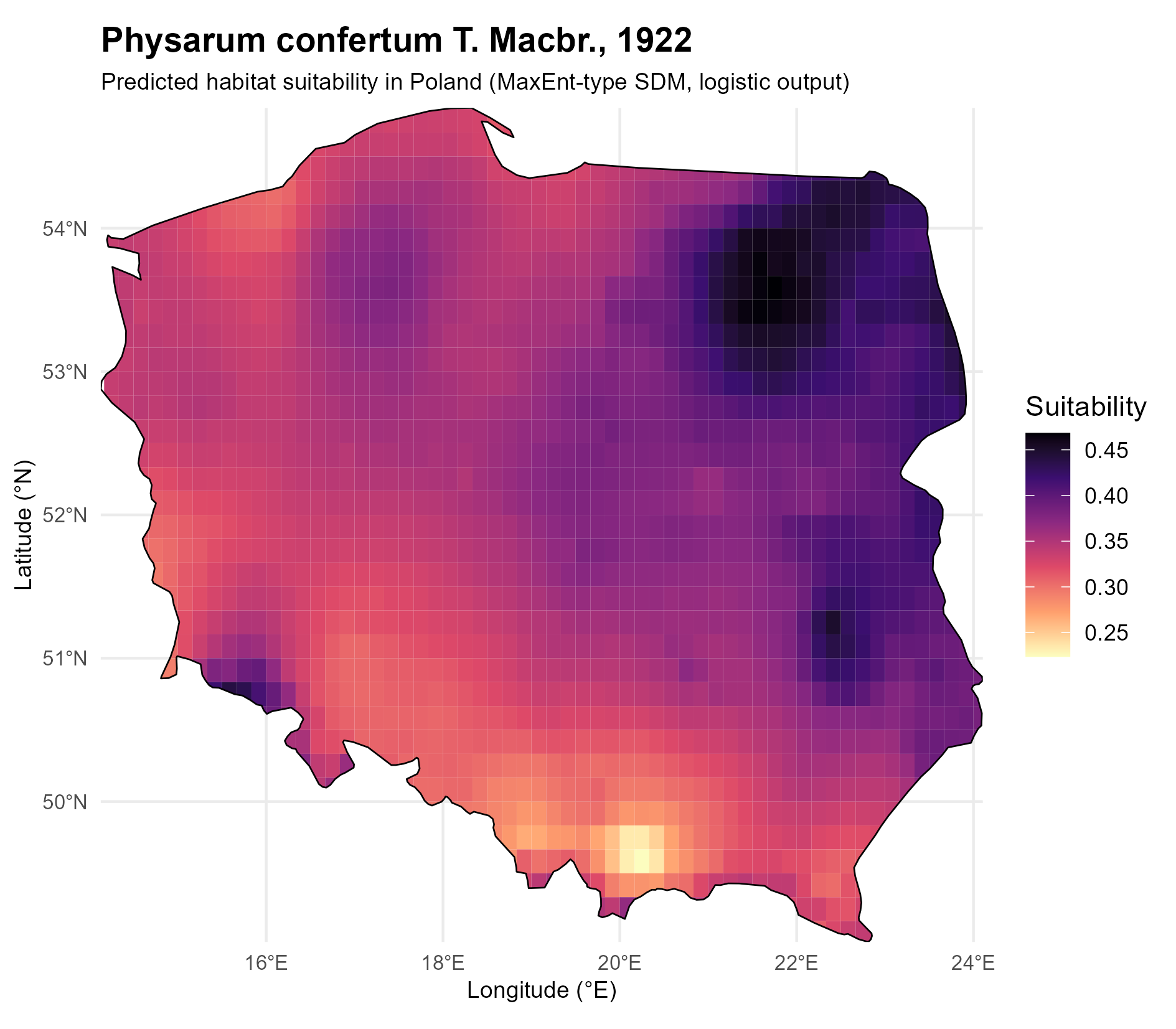

Supplement: Supplemental Information 12 — Set of 101 raster maps showing predicted potential distributions in Poland for modelled candidate species. Each figure displays continuous climatic suitability and the subset of grid cells exceeding a 10th-percentile training presence threshold. [file peerj-14-21492-s012.zip › Figure_SDM_poland_rank048_Physarum_confertum_T_Macbr_1922_MaxEnt_logistic.png]

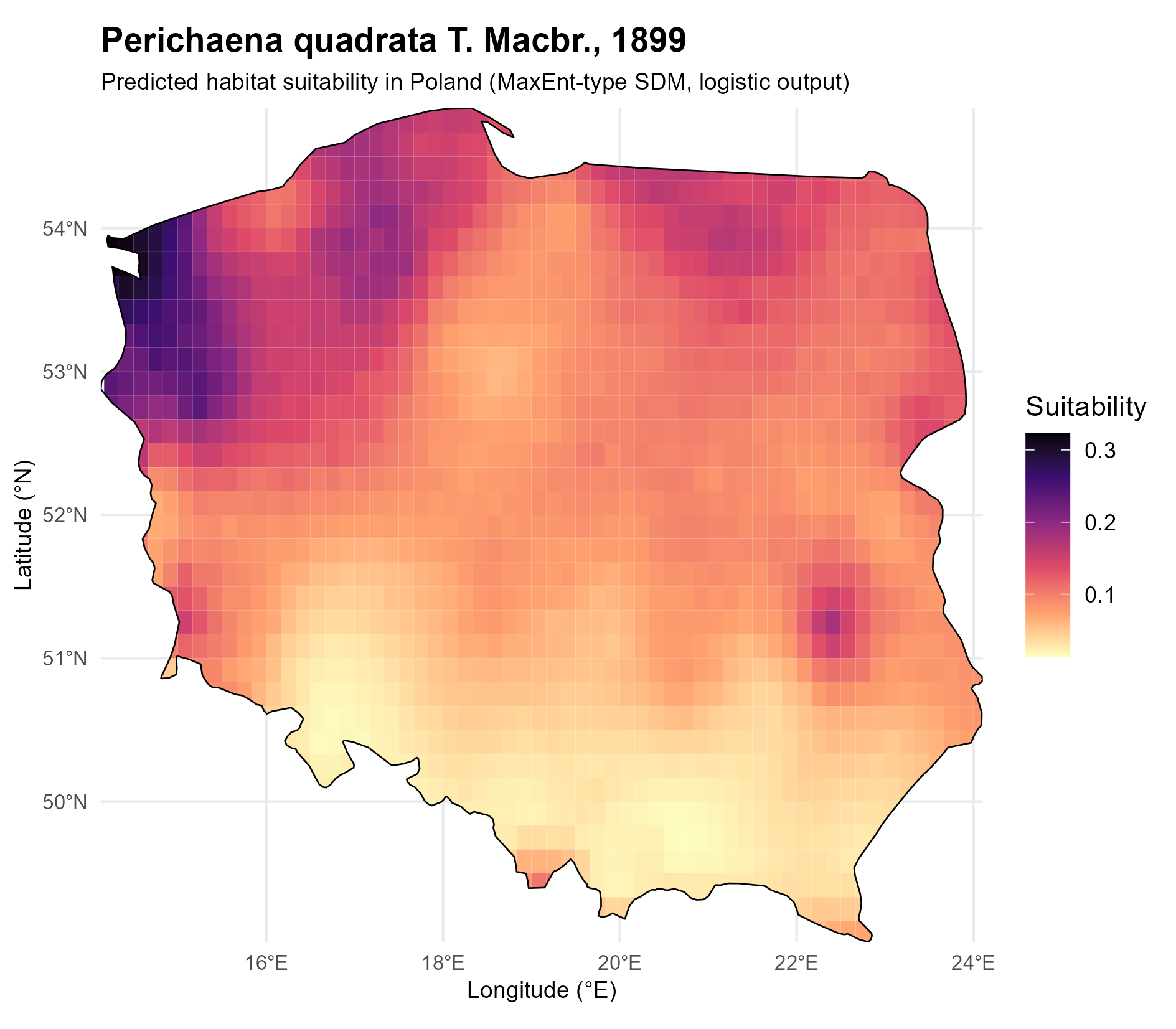

Supplement: Supplemental Information 12 — Set of 101 raster maps showing predicted potential distributions in Poland for modelled candidate species. Each figure displays continuous climatic suitability and the subset of grid cells exceeding a 10th-percentile training presence threshold. [file peerj-14-21492-s012.zip › Figure_SDM_poland_rank047_Perichaena_quadrata_T_Macbr_1899_MaxEnt_logistic.png]

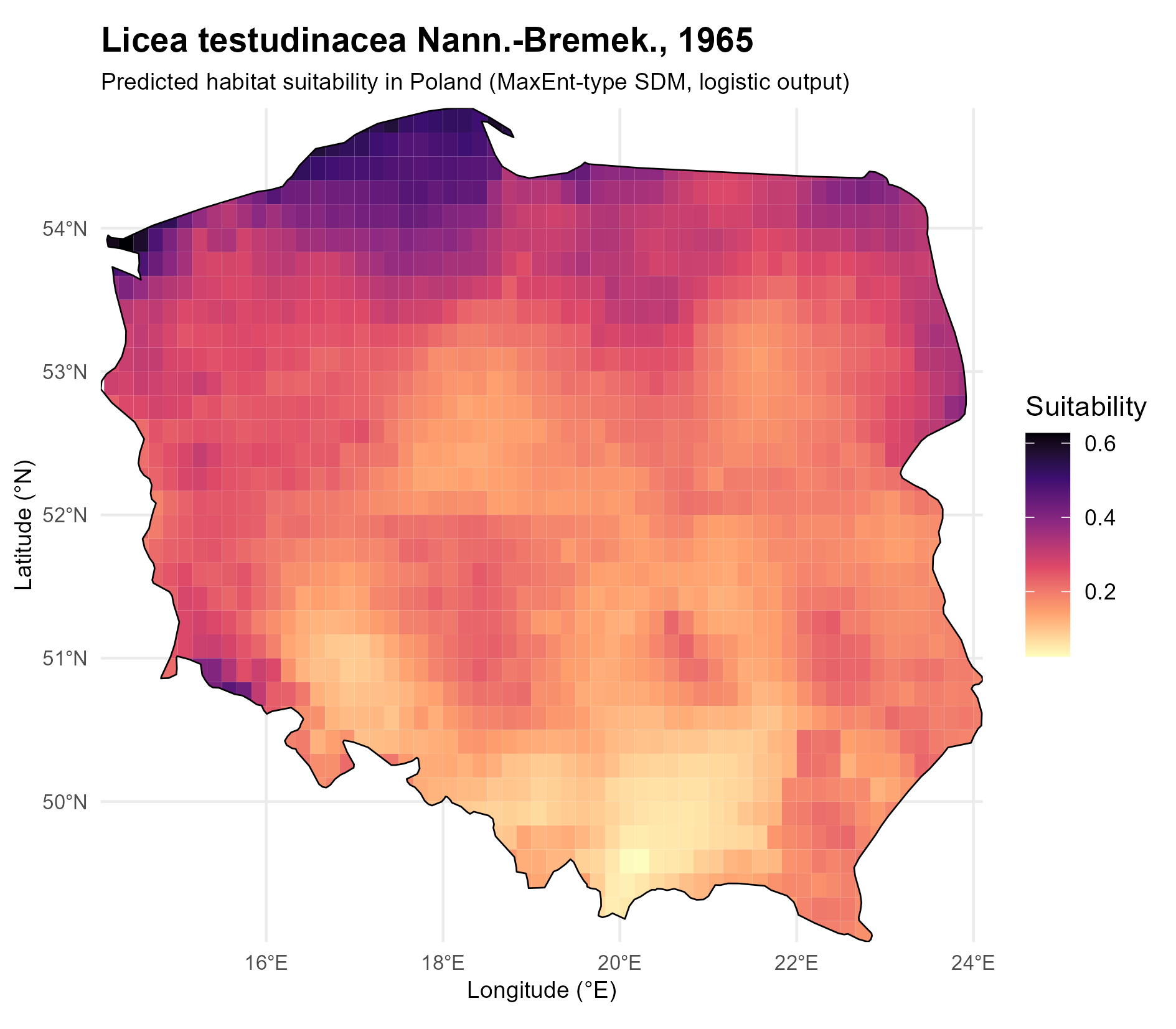

Supplement: Supplemental Information 12 — Set of 101 raster maps showing predicted potential distributions in Poland for modelled candidate species. Each figure displays continuous climatic suitability and the subset of grid cells exceeding a 10th-percentile training presence threshold. [file peerj-14-21492-s012.zip › Figure_SDM_poland_rank046_Licea_testudinacea_Nann_Bremek_1965_MaxEnt_logistic.png]

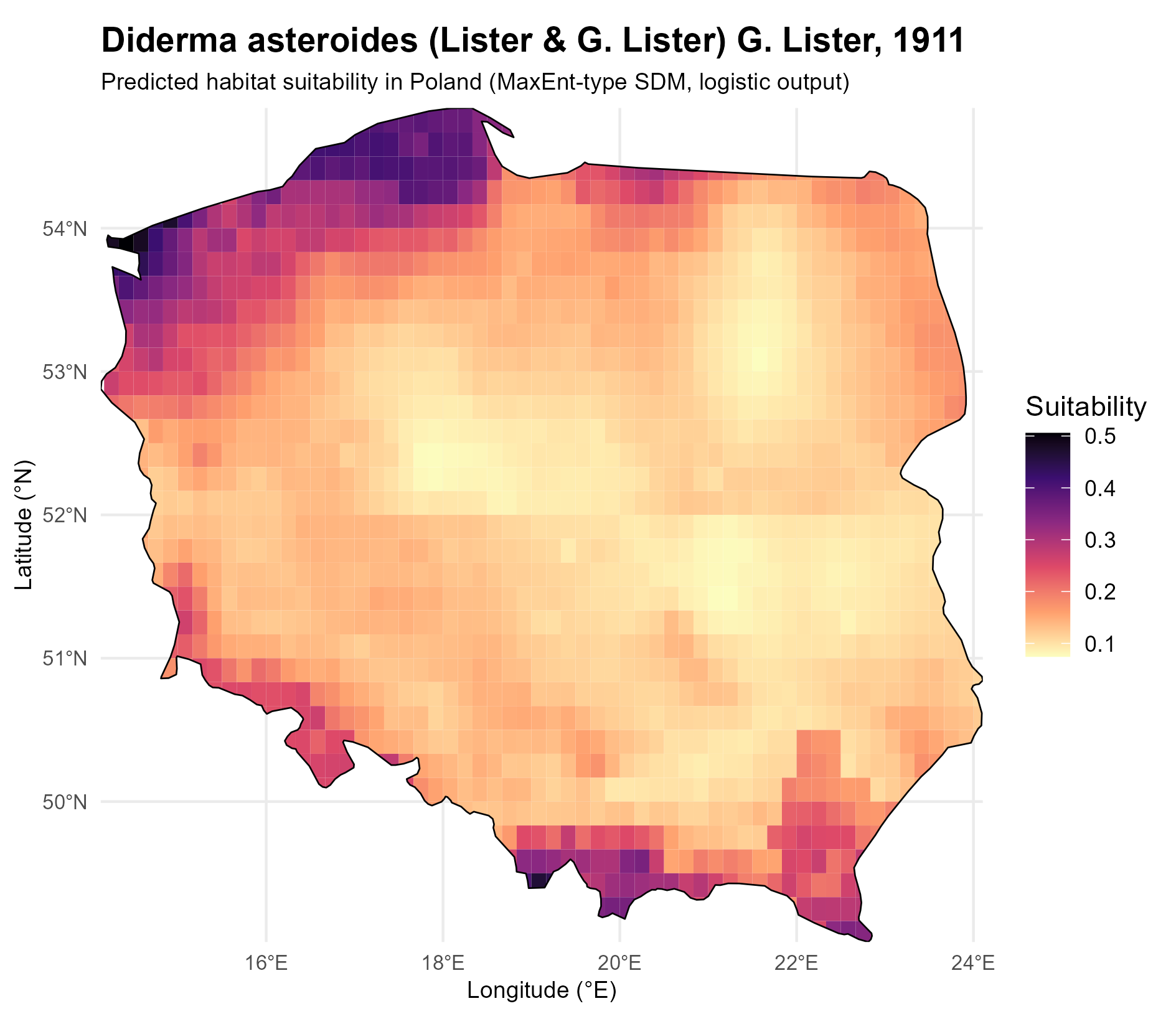

Supplement: Supplemental Information 12 — Set of 101 raster maps showing predicted potential distributions in Poland for modelled candidate species. Each figure displays continuous climatic suitability and the subset of grid cells exceeding a 10th-percentile training presence threshold. [file peerj-14-21492-s012.zip › Figure_SDM_poland_rank045_Diderma_asteroides_Lister_G_Lister_G_Lister_1911_MaxEnt_logistic.png]

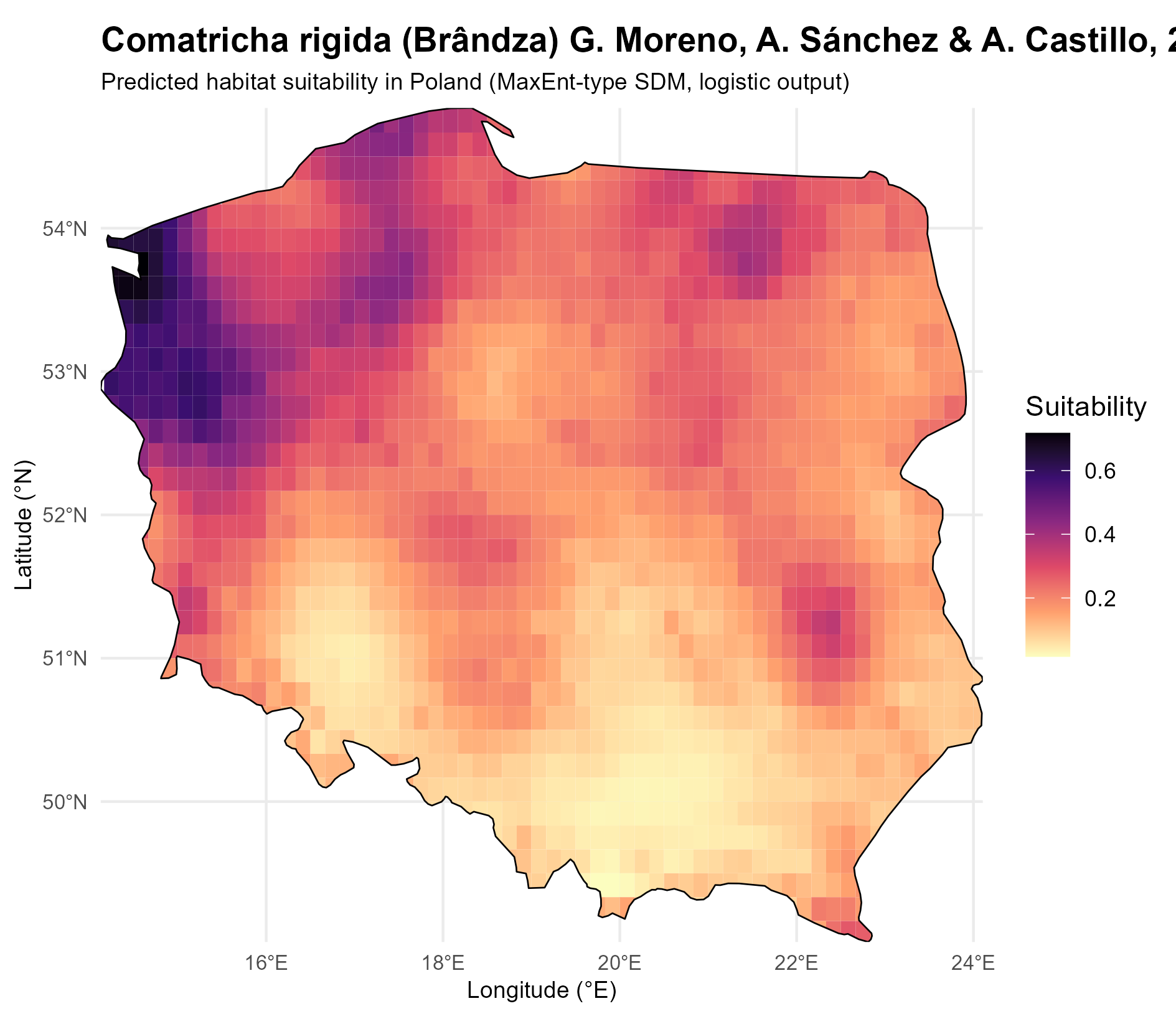

Supplement: Supplemental Information 12 — Set of 101 raster maps showing predicted potential distributions in Poland for modelled candidate species. Each figure displays continuous climatic suitability and the subset of grid cells exceeding a 10th-percentile training presence threshold. [file peerj-14-21492-s012.zip › Figure_SDM_poland_rank044_Comatricha_rigida_Brandza_G_Moreno_A_Sanchez_A_Castillo_2023_MaxEnt_logistic.png]

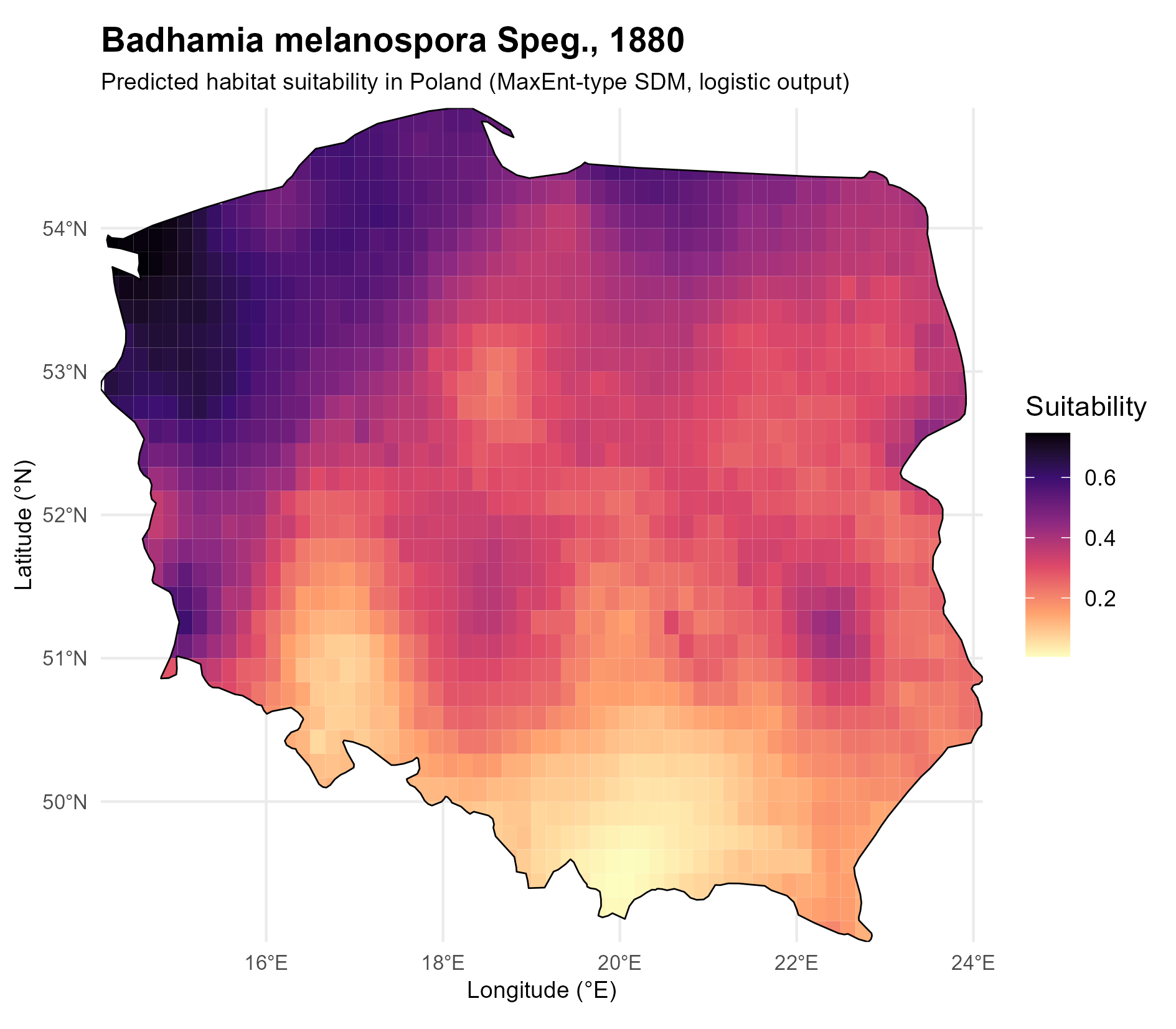

Supplement: Supplemental Information 12 — Set of 101 raster maps showing predicted potential distributions in Poland for modelled candidate species. Each figure displays continuous climatic suitability and the subset of grid cells exceeding a 10th-percentile training presence threshold. [file peerj-14-21492-s012.zip › Figure_SDM_poland_rank043_Badhamia_melanospora_Speg_1880_MaxEnt_logistic.png]

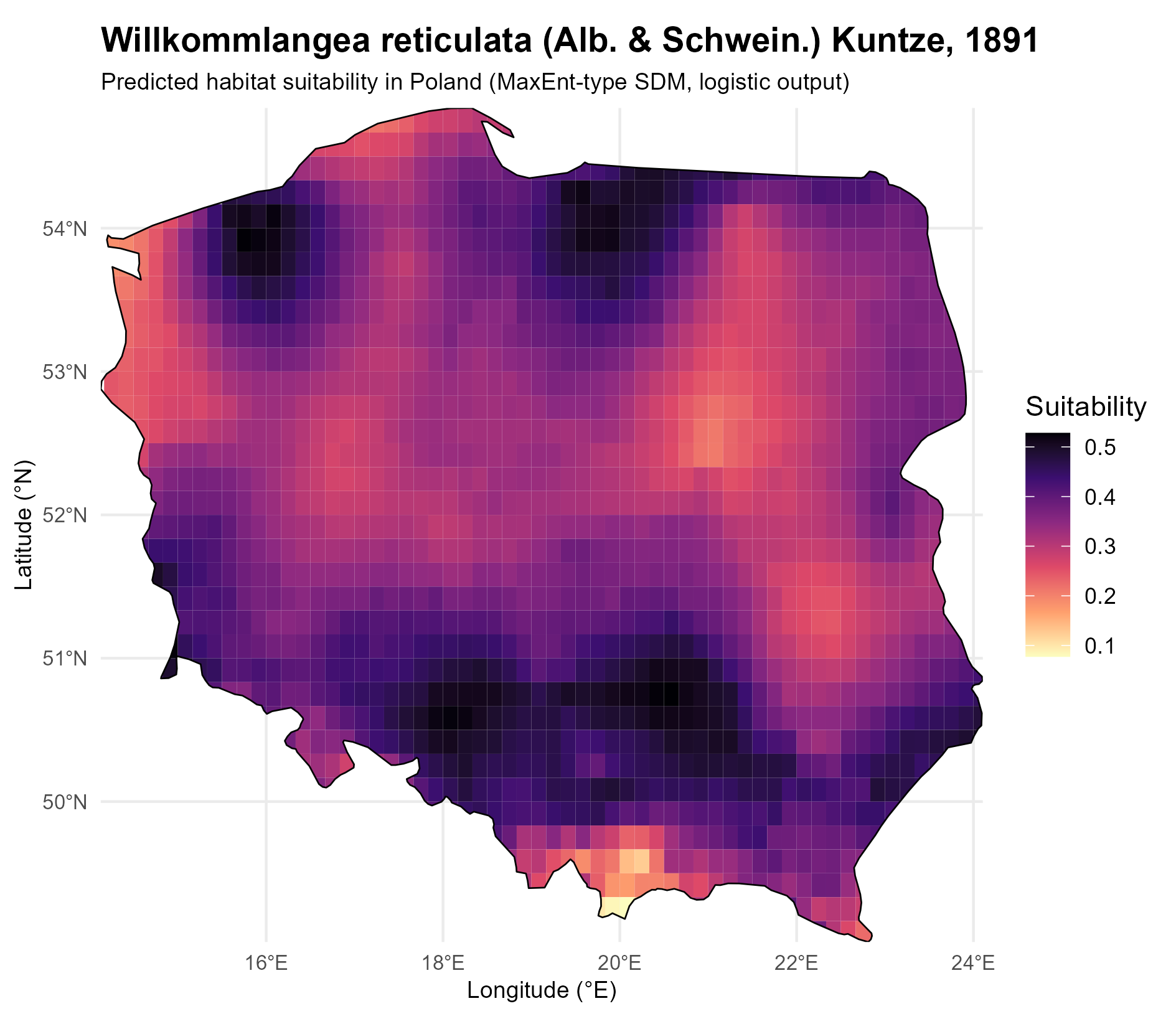

Supplement: Supplemental Information 12 — Set of 101 raster maps showing predicted potential distributions in Poland for modelled candidate species. Each figure displays continuous climatic suitability and the subset of grid cells exceeding a 10th-percentile training presence threshold. [file peerj-14-21492-s012.zip › Figure_SDM_poland_rank042_Willkommlangea_reticulata_Alb_Schwein_Kuntze_1891_MaxEnt_logistic.png]

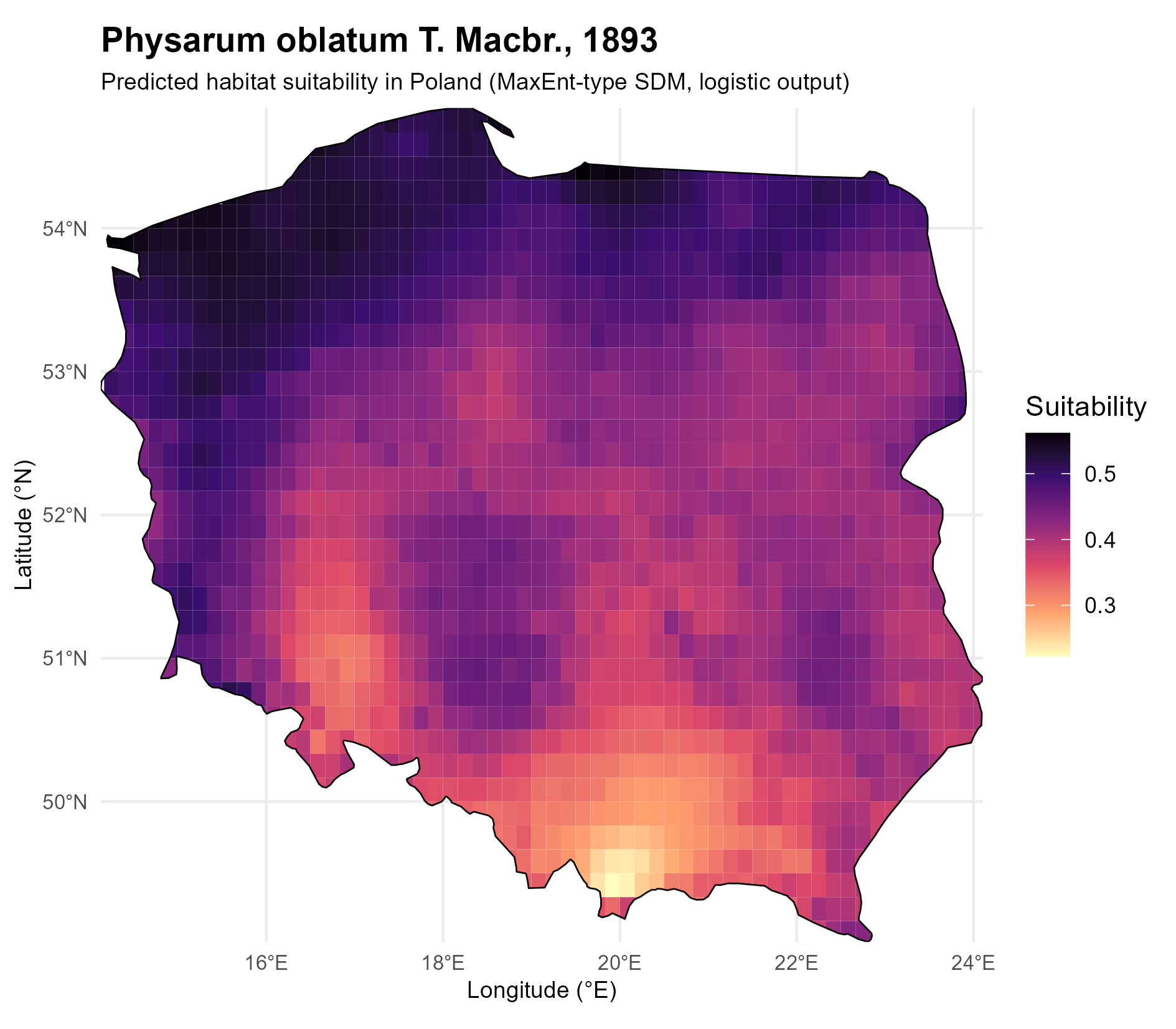

Supplement: Supplemental Information 12 — Set of 101 raster maps showing predicted potential distributions in Poland for modelled candidate species. Each figure displays continuous climatic suitability and the subset of grid cells exceeding a 10th-percentile training presence threshold. [file peerj-14-21492-s012.zip › Figure_SDM_poland_rank041_Physarum_oblatum_T_Macbr_1893_MaxEnt_logistic.png]

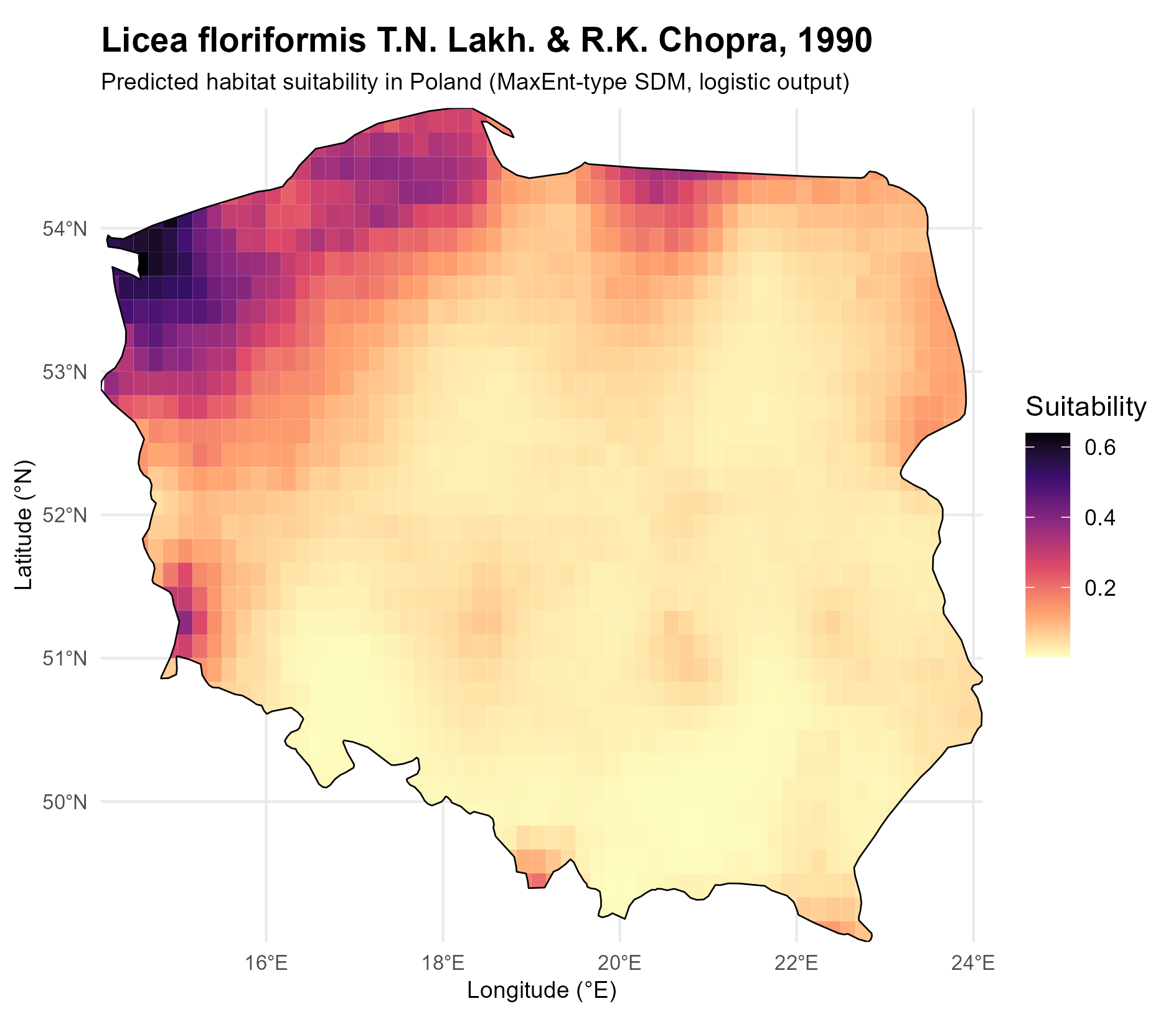

Supplement: Supplemental Information 12 — Set of 101 raster maps showing predicted potential distributions in Poland for modelled candidate species. Each figure displays continuous climatic suitability and the subset of grid cells exceeding a 10th-percentile training presence threshold. [file peerj-14-21492-s012.zip › Figure_SDM_poland_rank040_Licea_floriformis_T_N_Lakh_R_K_Chopra_1990_MaxEnt_logistic.png]

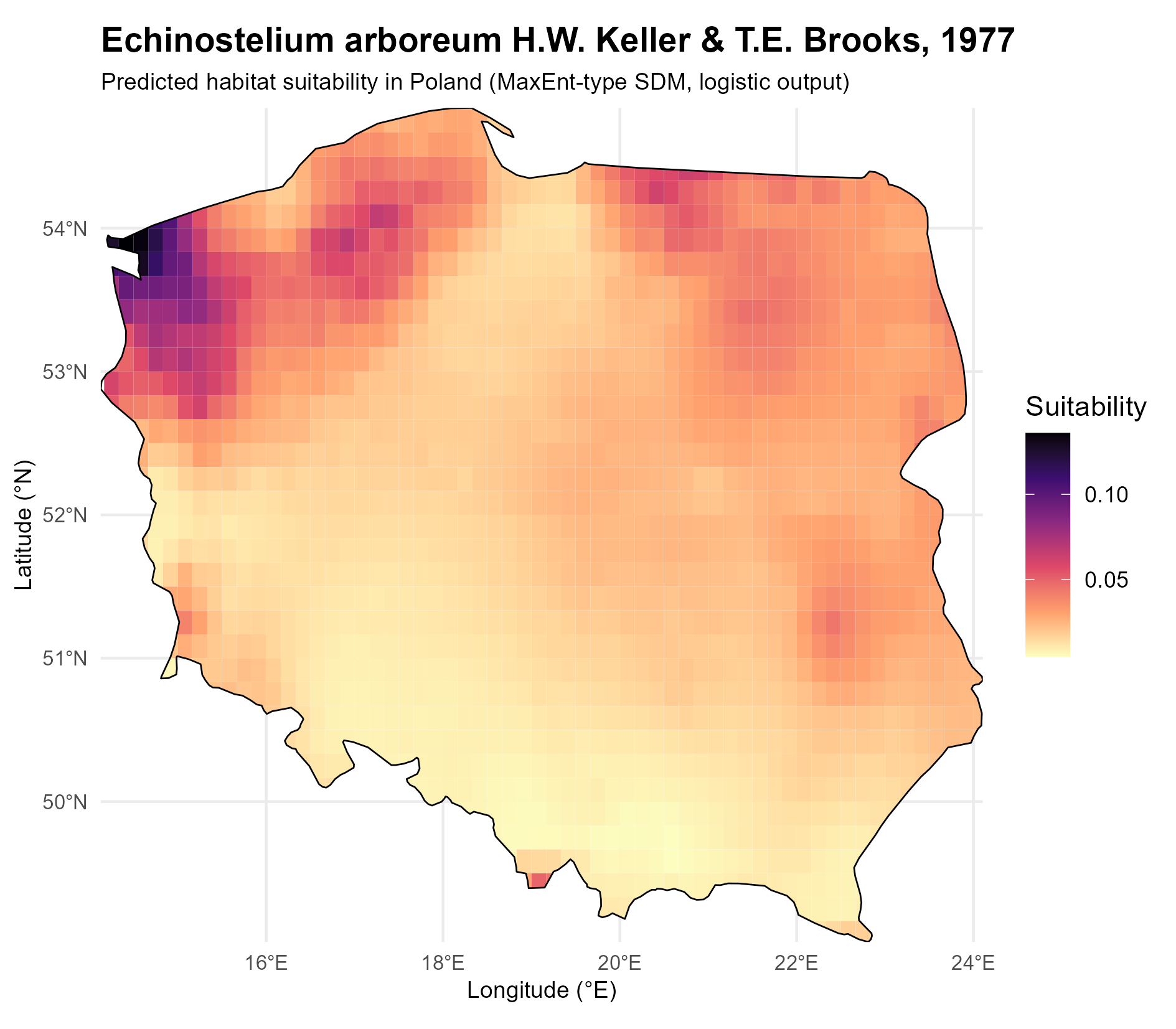

Supplement: Supplemental Information 12 — Set of 101 raster maps showing predicted potential distributions in Poland for modelled candidate species. Each figure displays continuous climatic suitability and the subset of grid cells exceeding a 10th-percentile training presence threshold. [file peerj-14-21492-s012.zip › Figure_SDM_poland_rank039_Echinostelium_arboreum_H_W_Keller_T_E_Brooks_1977_MaxEnt_logistic.png]

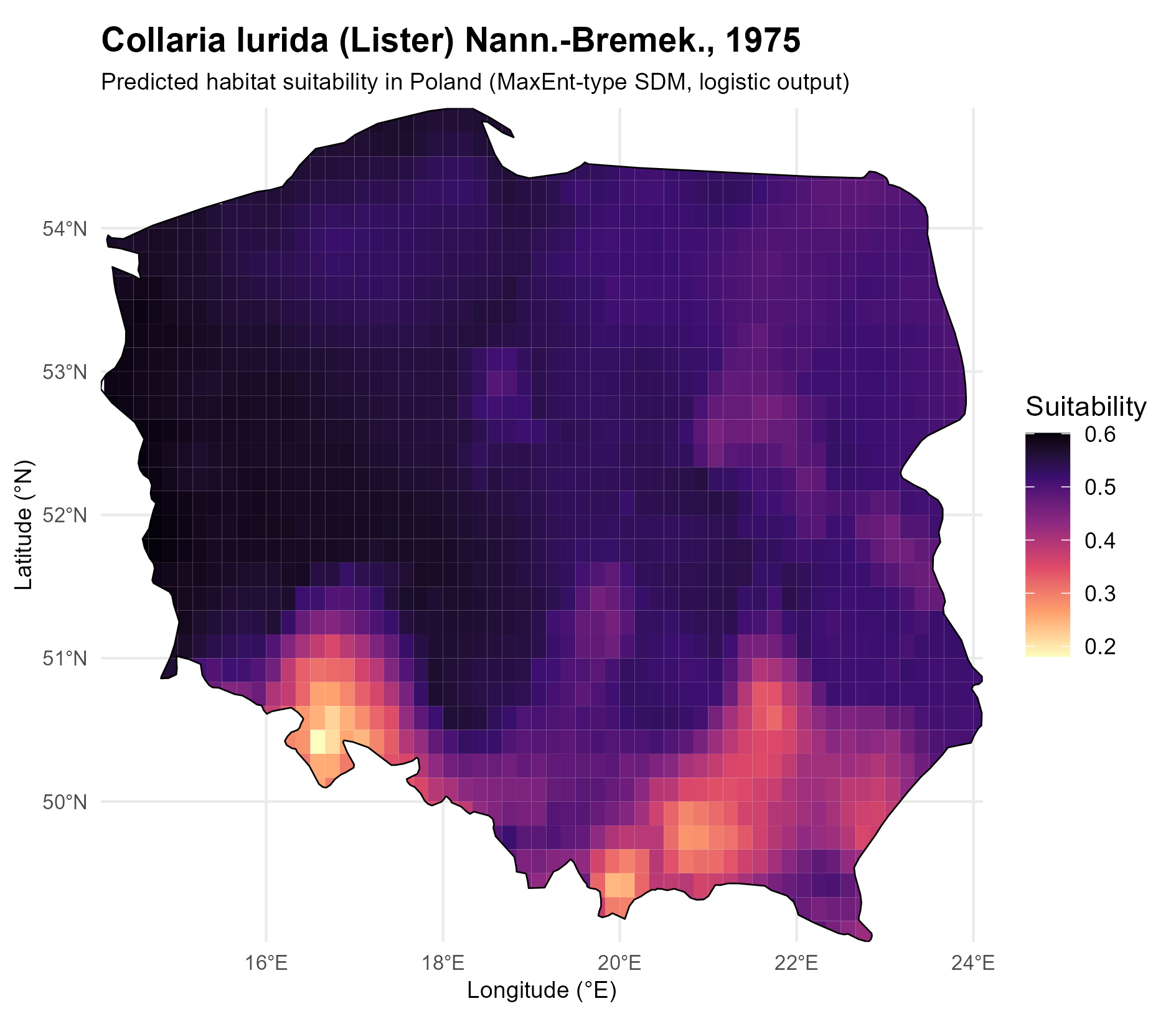

Supplement: Supplemental Information 12 — Set of 101 raster maps showing predicted potential distributions in Poland for modelled candidate species. Each figure displays continuous climatic suitability and the subset of grid cells exceeding a 10th-percentile training presence threshold. [file peerj-14-21492-s012.zip › Figure_SDM_poland_rank038_Collaria_lurida_Lister_Nann_Bremek_1975_MaxEnt_logistic.png]

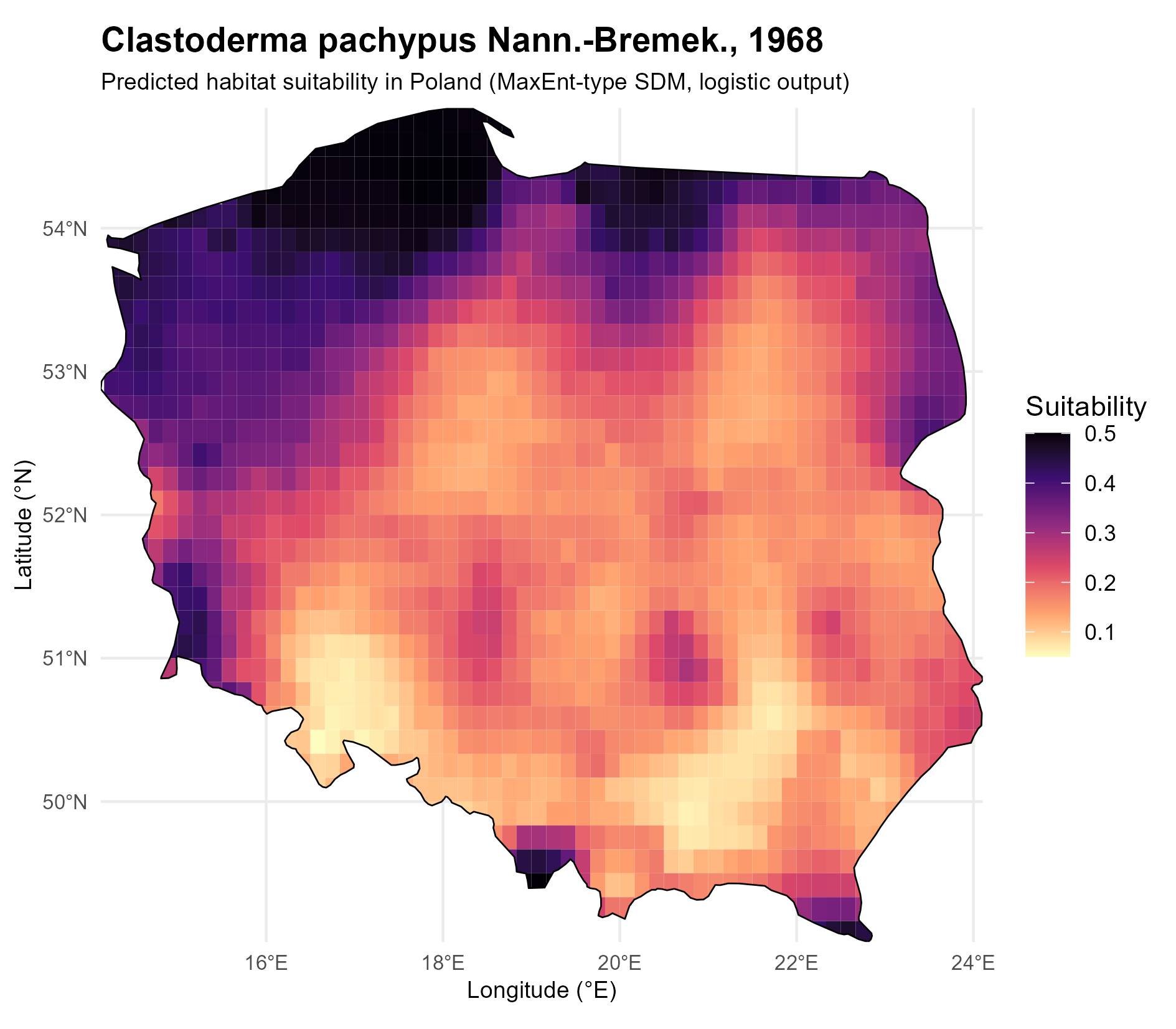

Supplement: Supplemental Information 12 — Set of 101 raster maps showing predicted potential distributions in Poland for modelled candidate species. Each figure displays continuous climatic suitability and the subset of grid cells exceeding a 10th-percentile training presence threshold. [file peerj-14-21492-s012.zip › Figure_SDM_poland_rank037_Clastoderma_pachypus_Nann_Bremek_1968_MaxEnt_logistic.png]

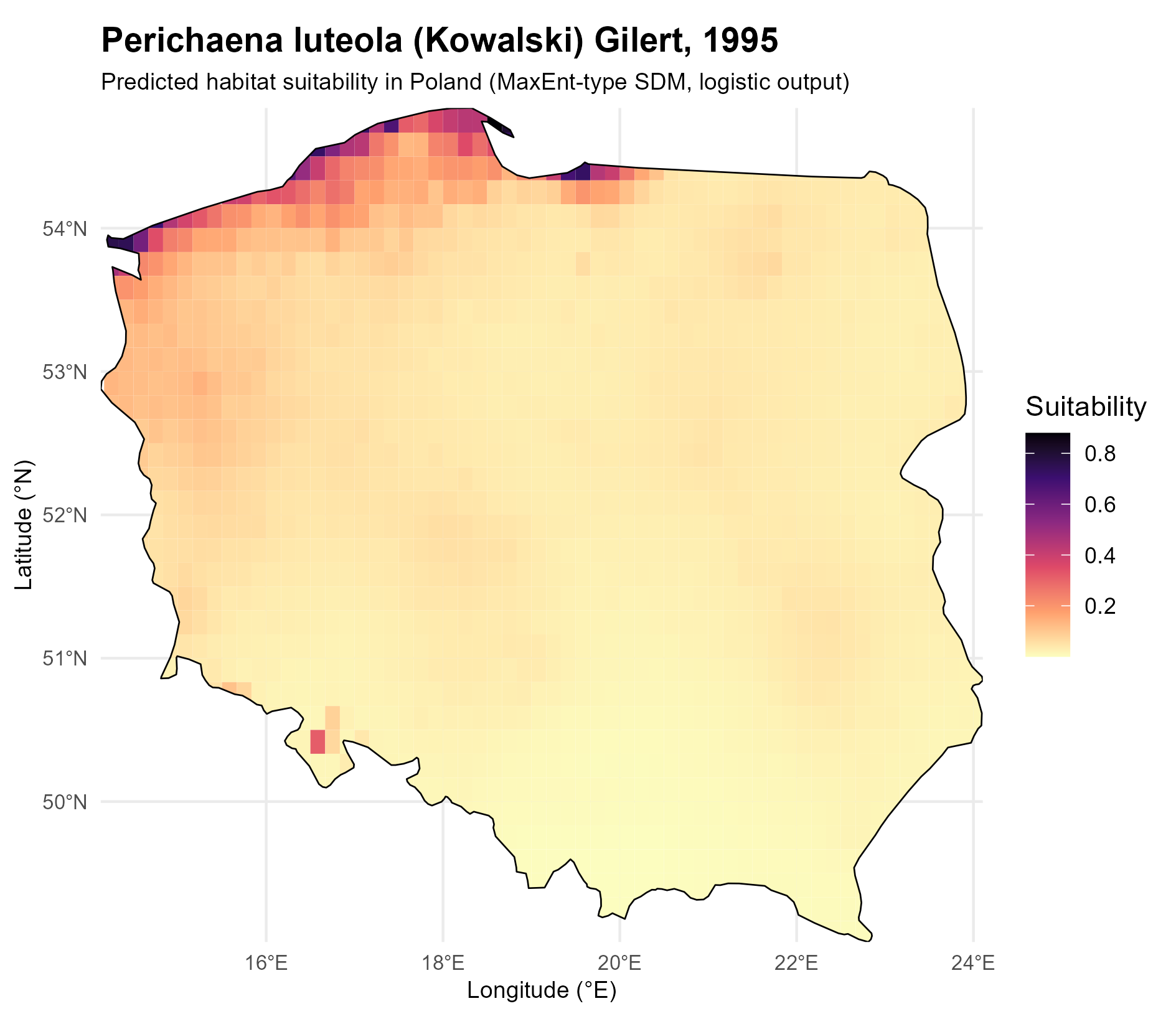

Supplement: Supplemental Information 12 — Set of 101 raster maps showing predicted potential distributions in Poland for modelled candidate species. Each figure displays continuous climatic suitability and the subset of grid cells exceeding a 10th-percentile training presence threshold. [file peerj-14-21492-s012.zip › Figure_SDM_poland_rank036_Perichaena_luteola_Kowalski_Gilert_1995_MaxEnt_logistic.png]

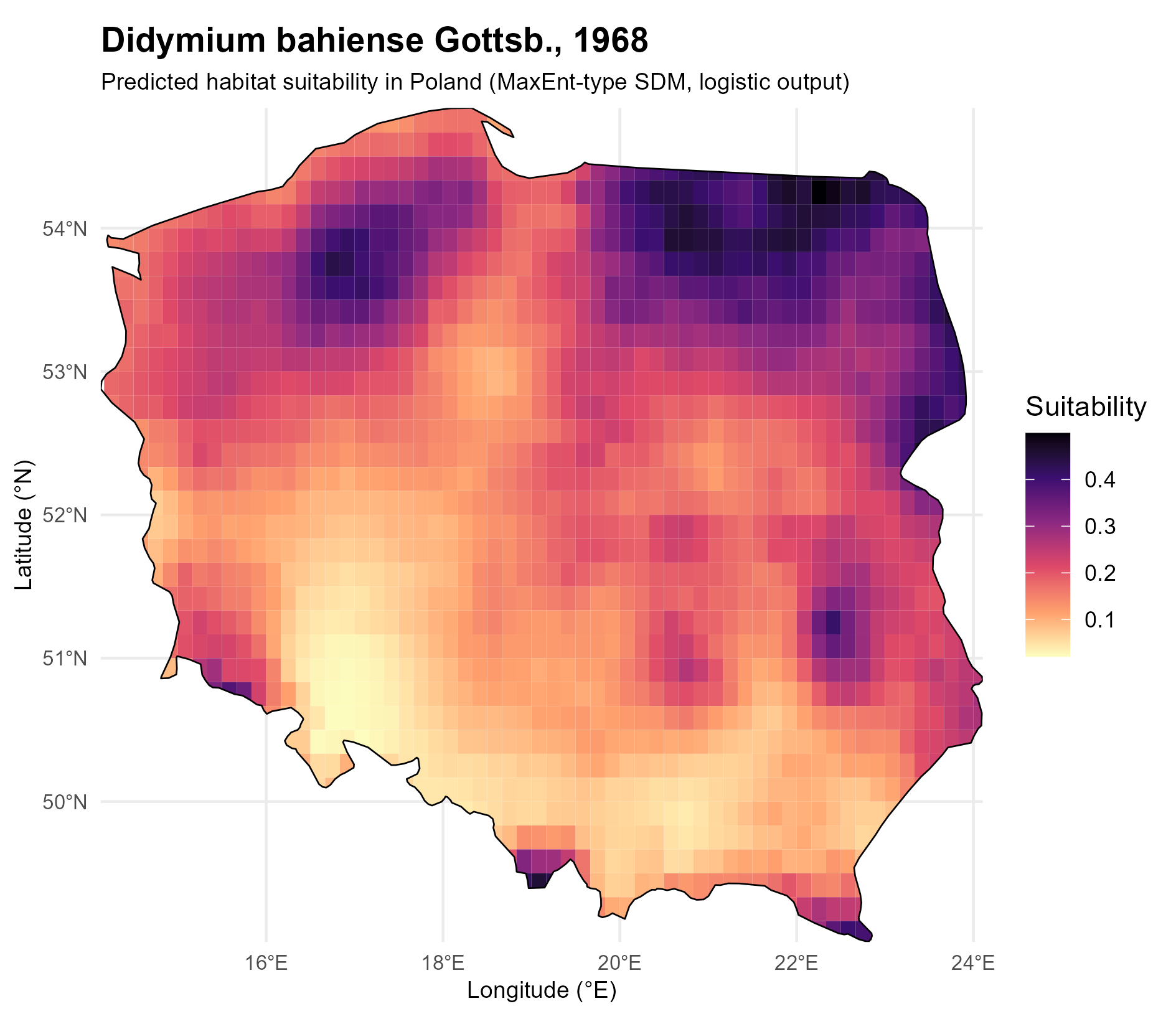

Supplement: Supplemental Information 12 — Set of 101 raster maps showing predicted potential distributions in Poland for modelled candidate species. Each figure displays continuous climatic suitability and the subset of grid cells exceeding a 10th-percentile training presence threshold. [file peerj-14-21492-s012.zip › Figure_SDM_poland_rank035_Didymium_bahiense_Gottsb_1968_MaxEnt_logistic.png]

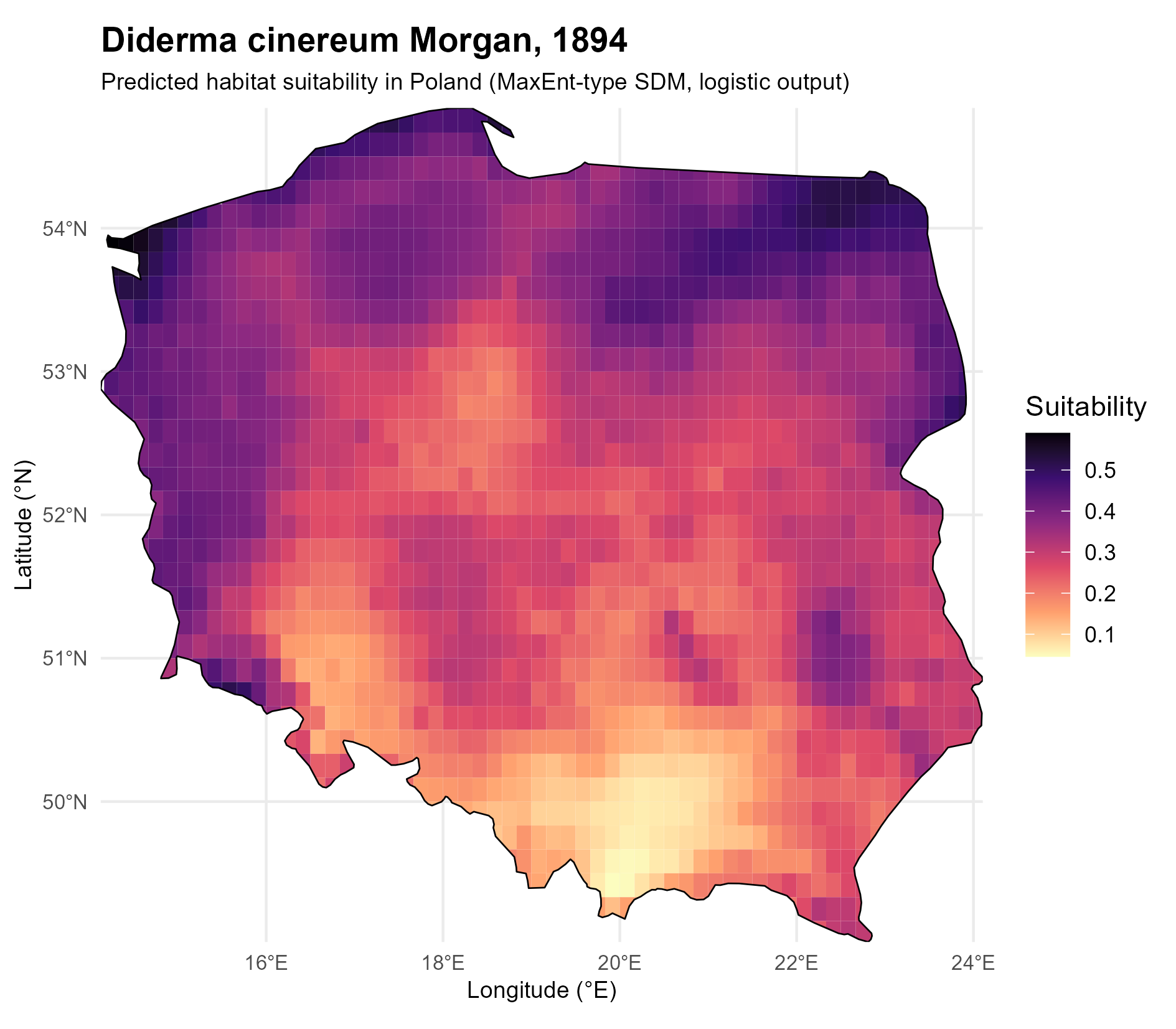

Supplement: Supplemental Information 12 — Set of 101 raster maps showing predicted potential distributions in Poland for modelled candidate species. Each figure displays continuous climatic suitability and the subset of grid cells exceeding a 10th-percentile training presence threshold. [file peerj-14-21492-s012.zip › Figure_SDM_poland_rank034_Diderma_cinereum_Morgan_1894_MaxEnt_logistic.png]

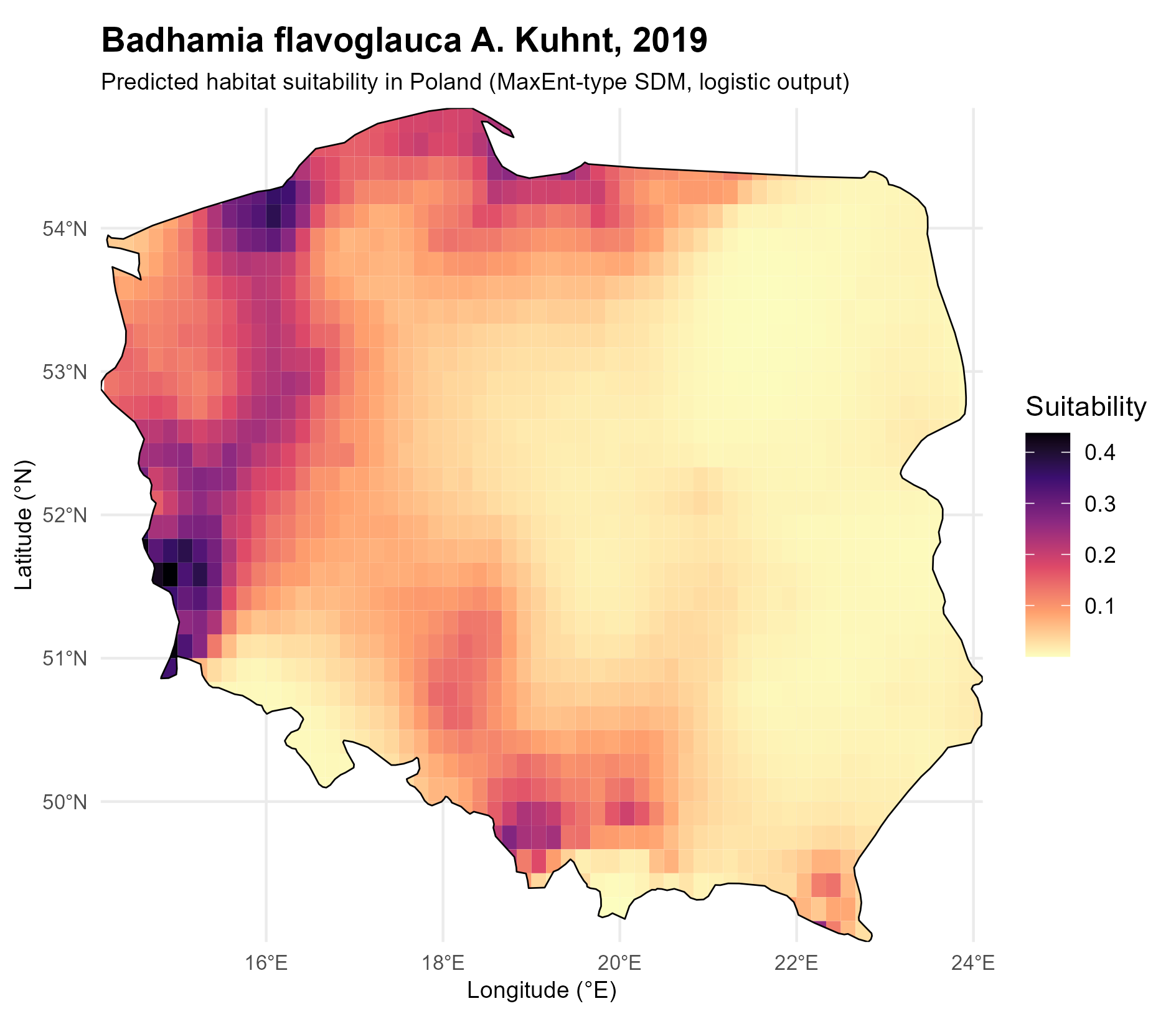

Supplement: Supplemental Information 12 — Set of 101 raster maps showing predicted potential distributions in Poland for modelled candidate species. Each figure displays continuous climatic suitability and the subset of grid cells exceeding a 10th-percentile training presence threshold. [file peerj-14-21492-s012.zip › Figure_SDM_poland_rank033_Badhamia_flavoglauca_A_Kuhnt_2019_MaxEnt_logistic.png]

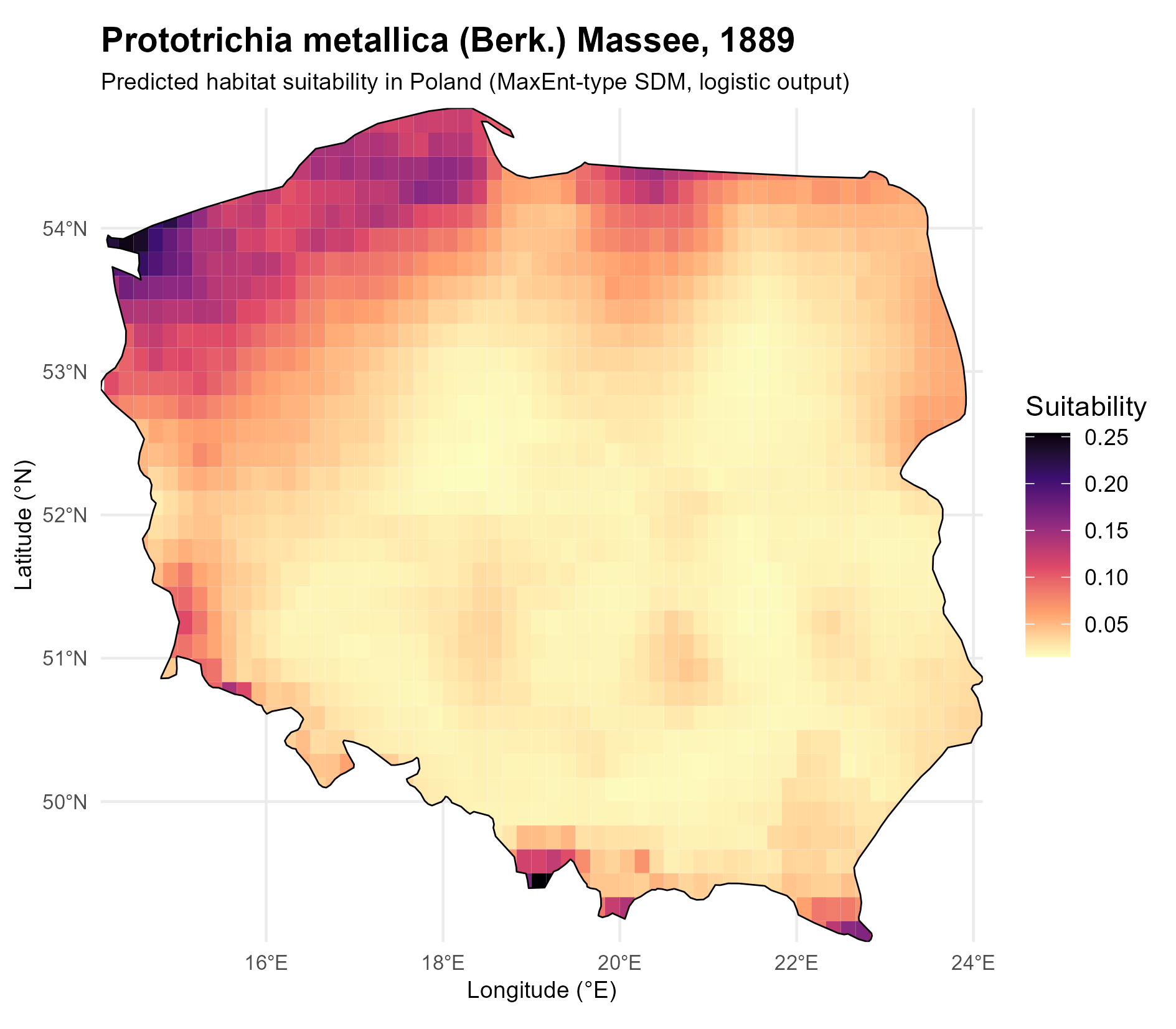

Supplement: Supplemental Information 12 — Set of 101 raster maps showing predicted potential distributions in Poland for modelled candidate species. Each figure displays continuous climatic suitability and the subset of grid cells exceeding a 10th-percentile training presence threshold. [file peerj-14-21492-s012.zip › Figure_SDM_poland_rank032_Prototrichia_metallica_Berk_Massee_1889_MaxEnt_logistic.png]

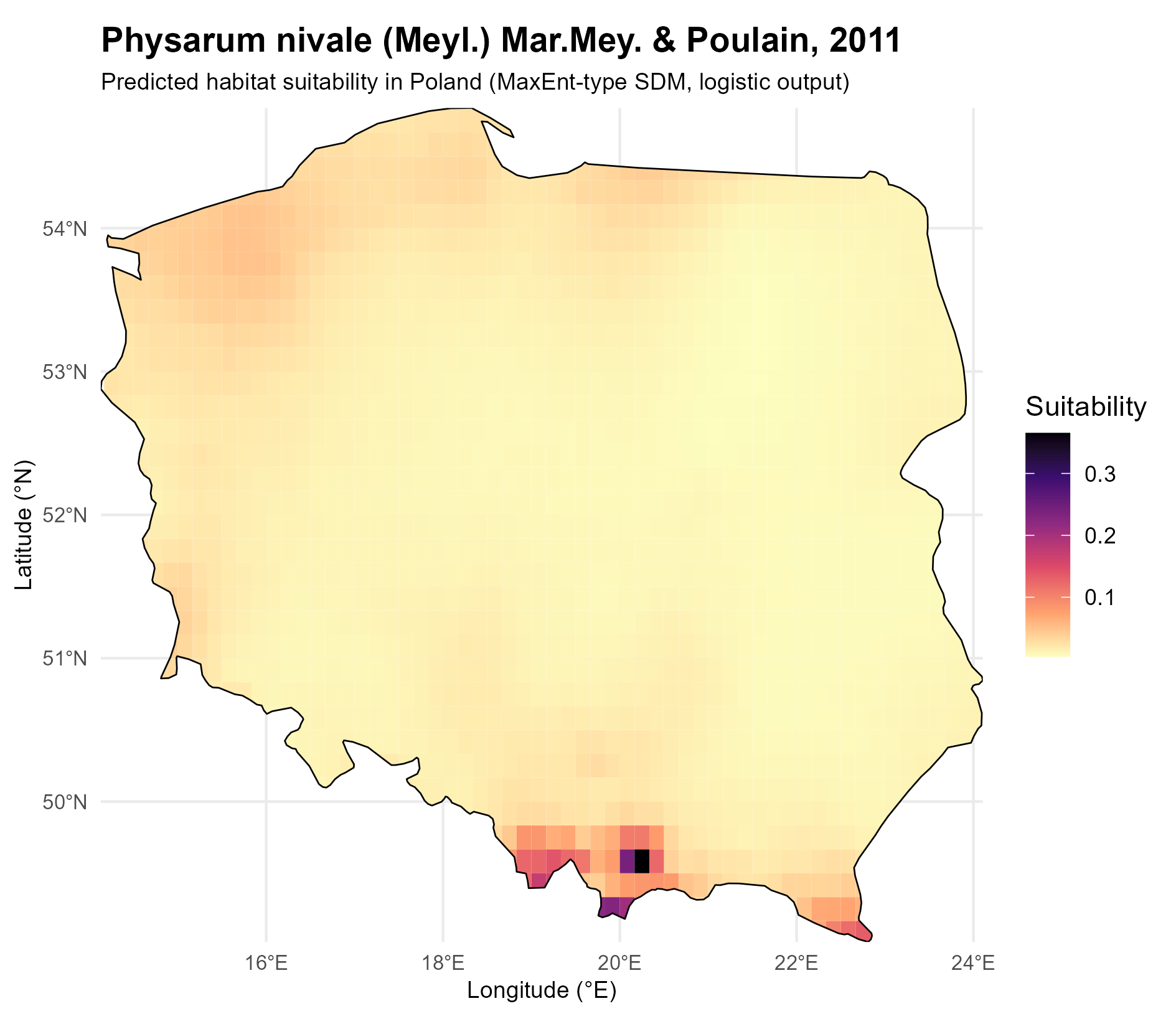

Supplement: Supplemental Information 12 — Set of 101 raster maps showing predicted potential distributions in Poland for modelled candidate species. Each figure displays continuous climatic suitability and the subset of grid cells exceeding a 10th-percentile training presence threshold. [file peerj-14-21492-s012.zip › Figure_SDM_poland_rank031_Physarum_nivale_Meyl_Mar_Mey_Poulain_2011_MaxEnt_logistic.png]

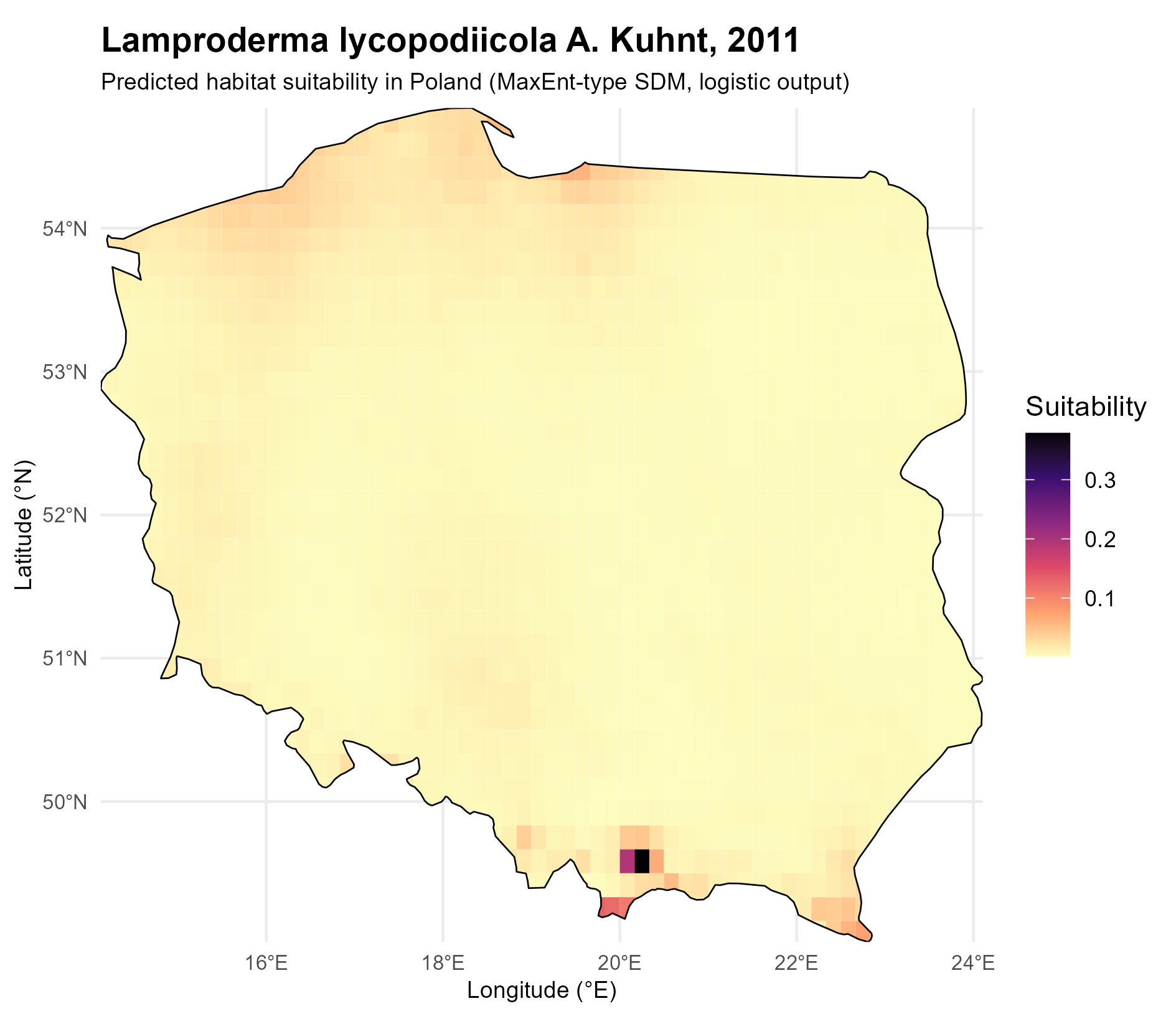

Supplement: Supplemental Information 12 — Set of 101 raster maps showing predicted potential distributions in Poland for modelled candidate species. Each figure displays continuous climatic suitability and the subset of grid cells exceeding a 10th-percentile training presence threshold. [file peerj-14-21492-s012.zip › Figure_SDM_poland_rank030_Lamproderma_lycopodiicola_A_Kuhnt_2011_MaxEnt_logistic.png]

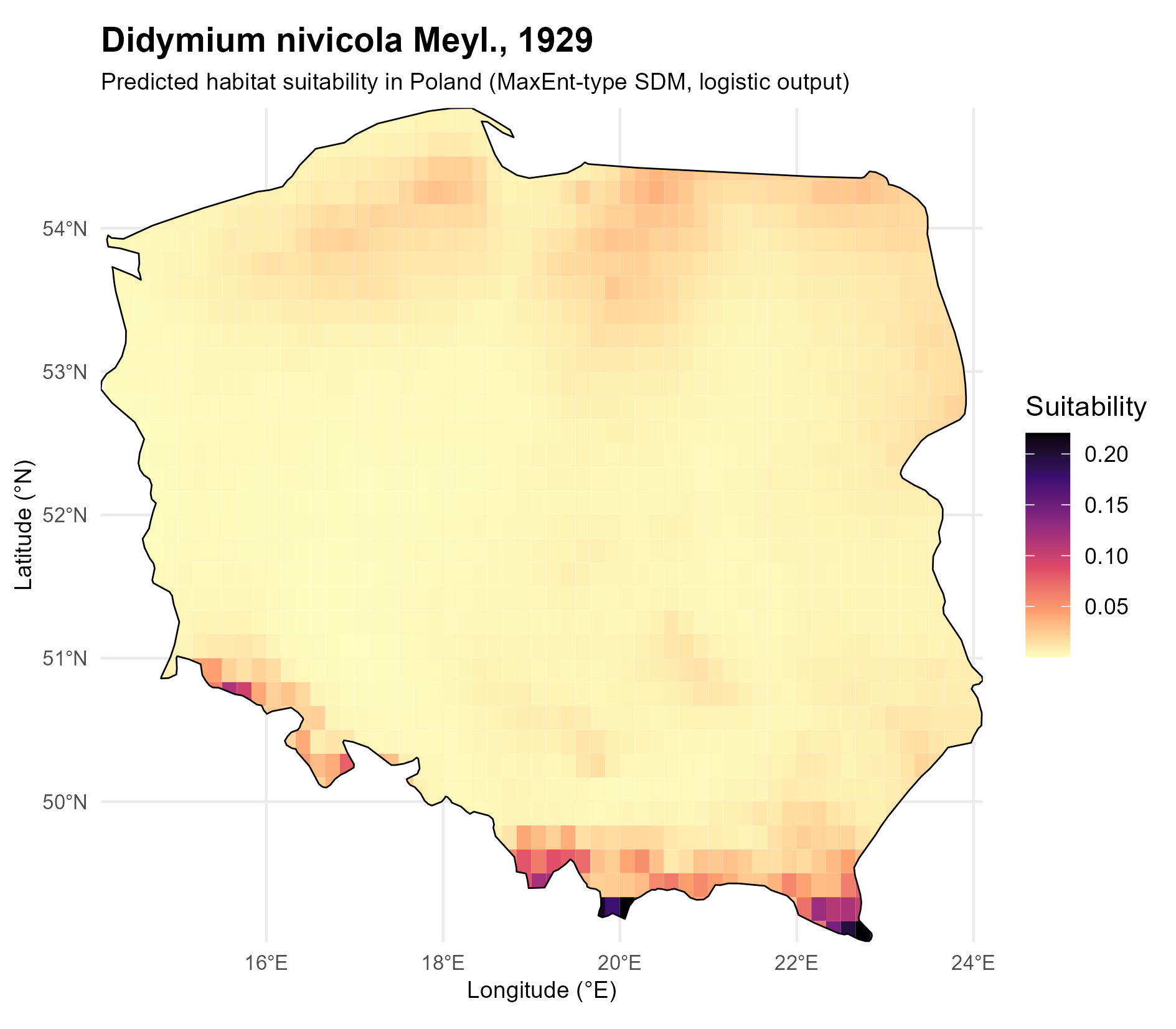

Supplement: Supplemental Information 12 — Set of 101 raster maps showing predicted potential distributions in Poland for modelled candidate species. Each figure displays continuous climatic suitability and the subset of grid cells exceeding a 10th-percentile training presence threshold. [file peerj-14-21492-s012.zip › Figure_SDM_poland_rank029_Didymium_nivicola_Meyl_1929_MaxEnt_logistic.png]

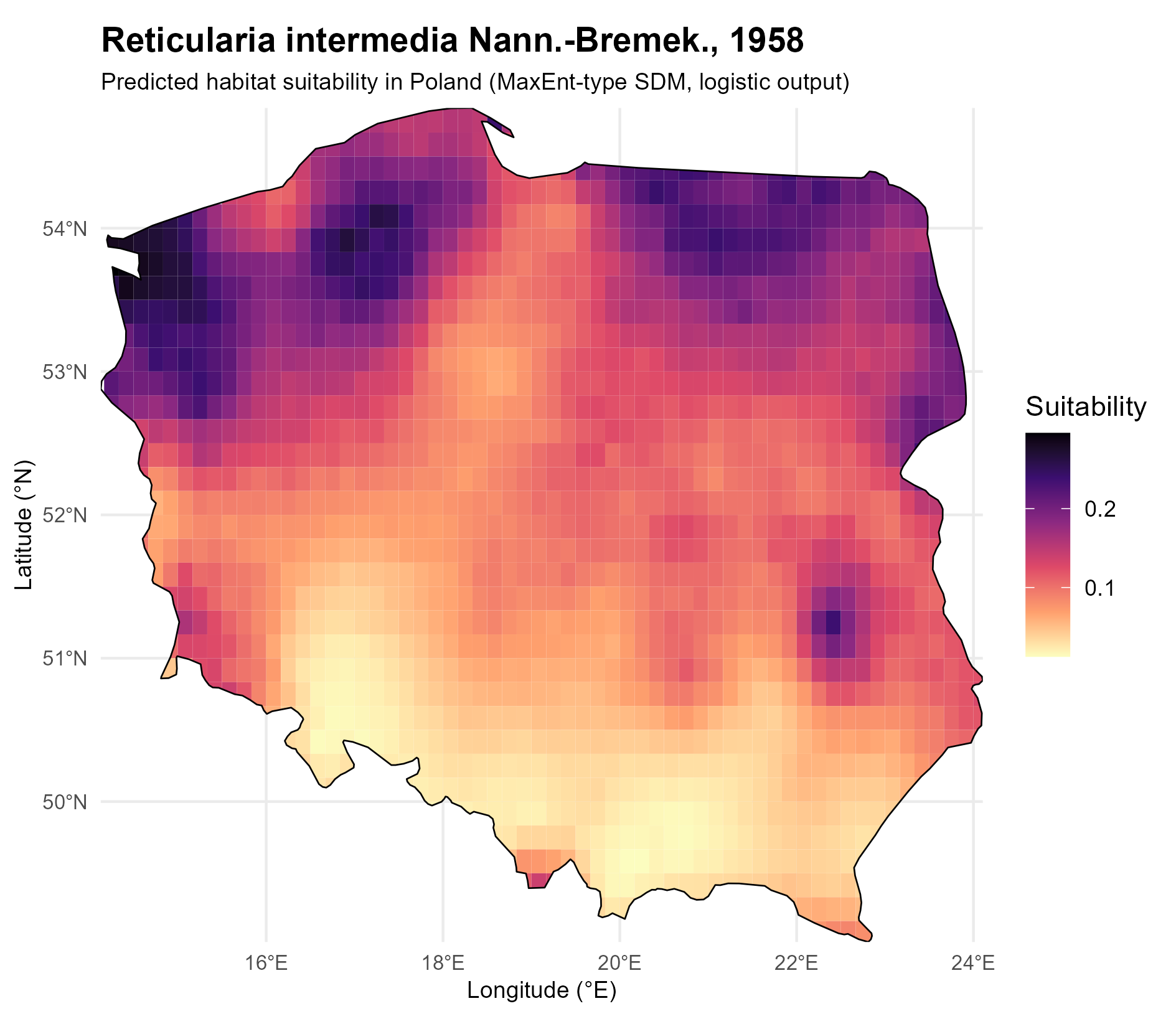

Supplement: Supplemental Information 12 — Set of 101 raster maps showing predicted potential distributions in Poland for modelled candidate species. Each figure displays continuous climatic suitability and the subset of grid cells exceeding a 10th-percentile training presence threshold. [file peerj-14-21492-s012.zip › Figure_SDM_poland_rank028_Reticularia_intermedia_Nann_Bremek_1958_MaxEnt_logistic.png]

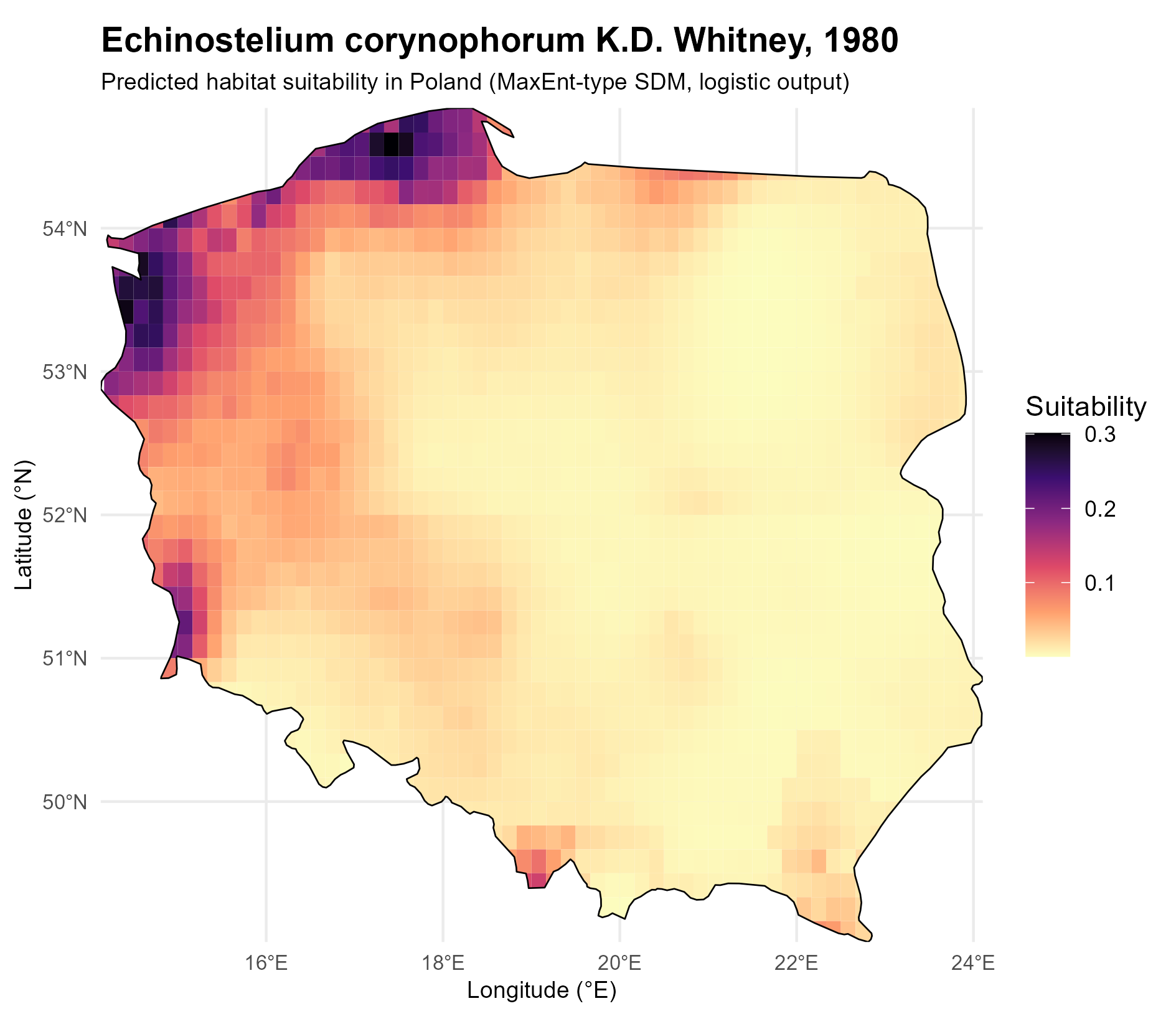

Supplement: Supplemental Information 12 — Set of 101 raster maps showing predicted potential distributions in Poland for modelled candidate species. Each figure displays continuous climatic suitability and the subset of grid cells exceeding a 10th-percentile training presence threshold. [file peerj-14-21492-s012.zip › Figure_SDM_poland_rank027_Echinostelium_corynophorum_K_D_Whitney_1980_MaxEnt_logistic.png]

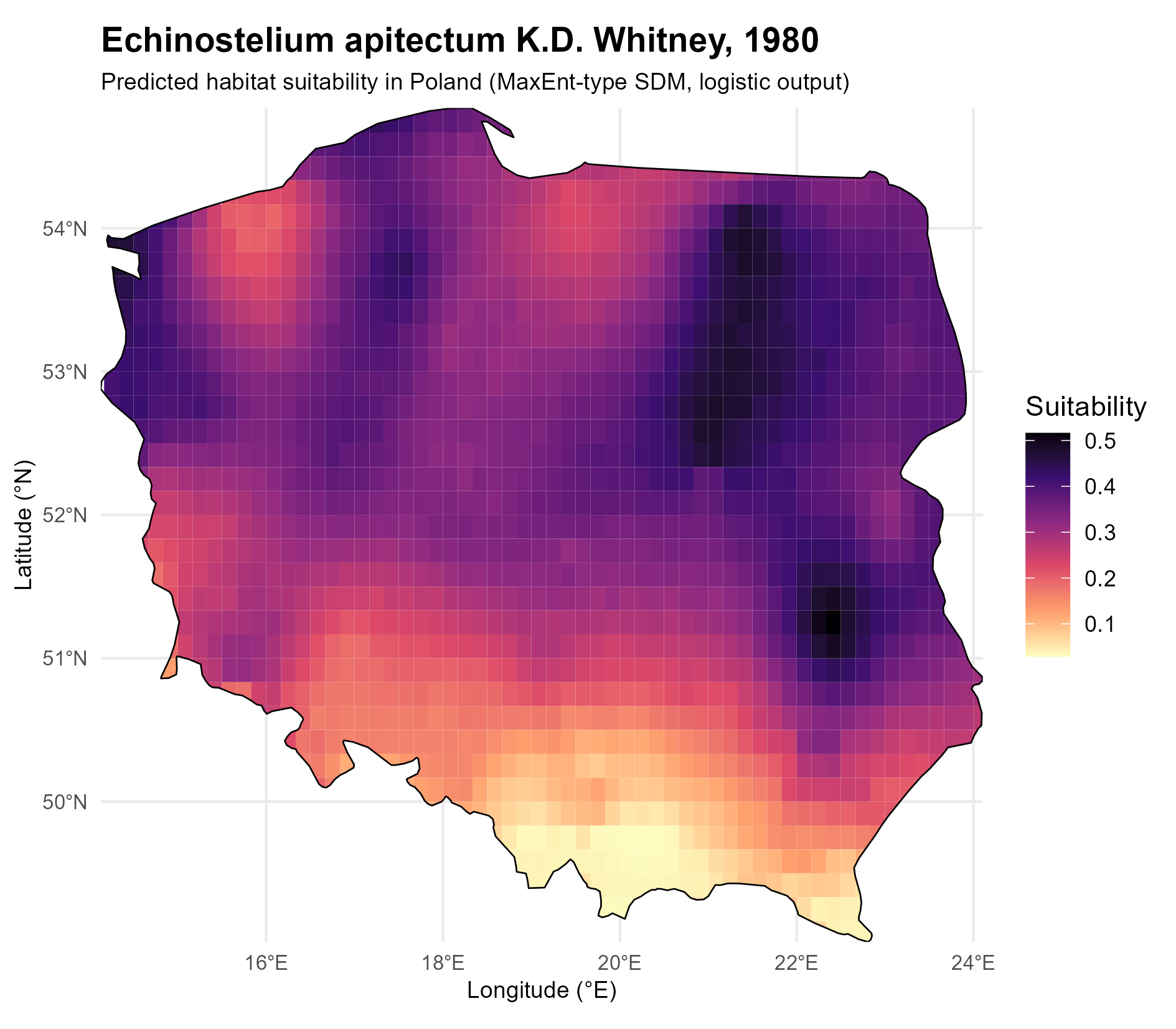

Supplement: Supplemental Information 12 — Set of 101 raster maps showing predicted potential distributions in Poland for modelled candidate species. Each figure displays continuous climatic suitability and the subset of grid cells exceeding a 10th-percentile training presence threshold. [file peerj-14-21492-s012.zip › Figure_SDM_poland_rank026_Echinostelium_apitectum_K_D_Whitney_1980_MaxEnt_logistic.png]

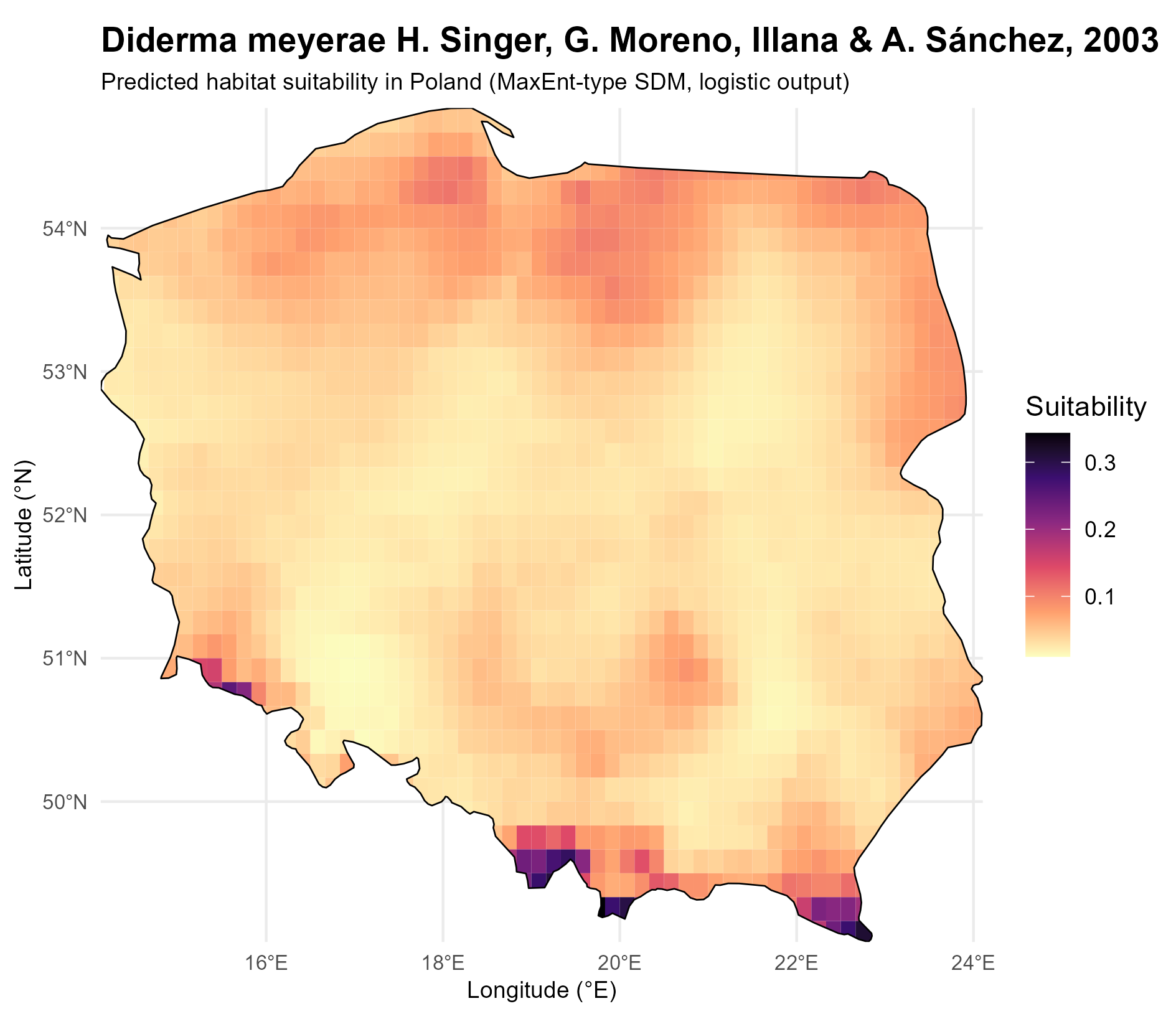

Supplement: Supplemental Information 12 — Set of 101 raster maps showing predicted potential distributions in Poland for modelled candidate species. Each figure displays continuous climatic suitability and the subset of grid cells exceeding a 10th-percentile training presence threshold. [file peerj-14-21492-s012.zip › Figure_SDM_poland_rank025_Diderma_meyerae_H_Singer_G_Moreno_Illana_A_Sanchez_2003_MaxEnt_logistic.png]

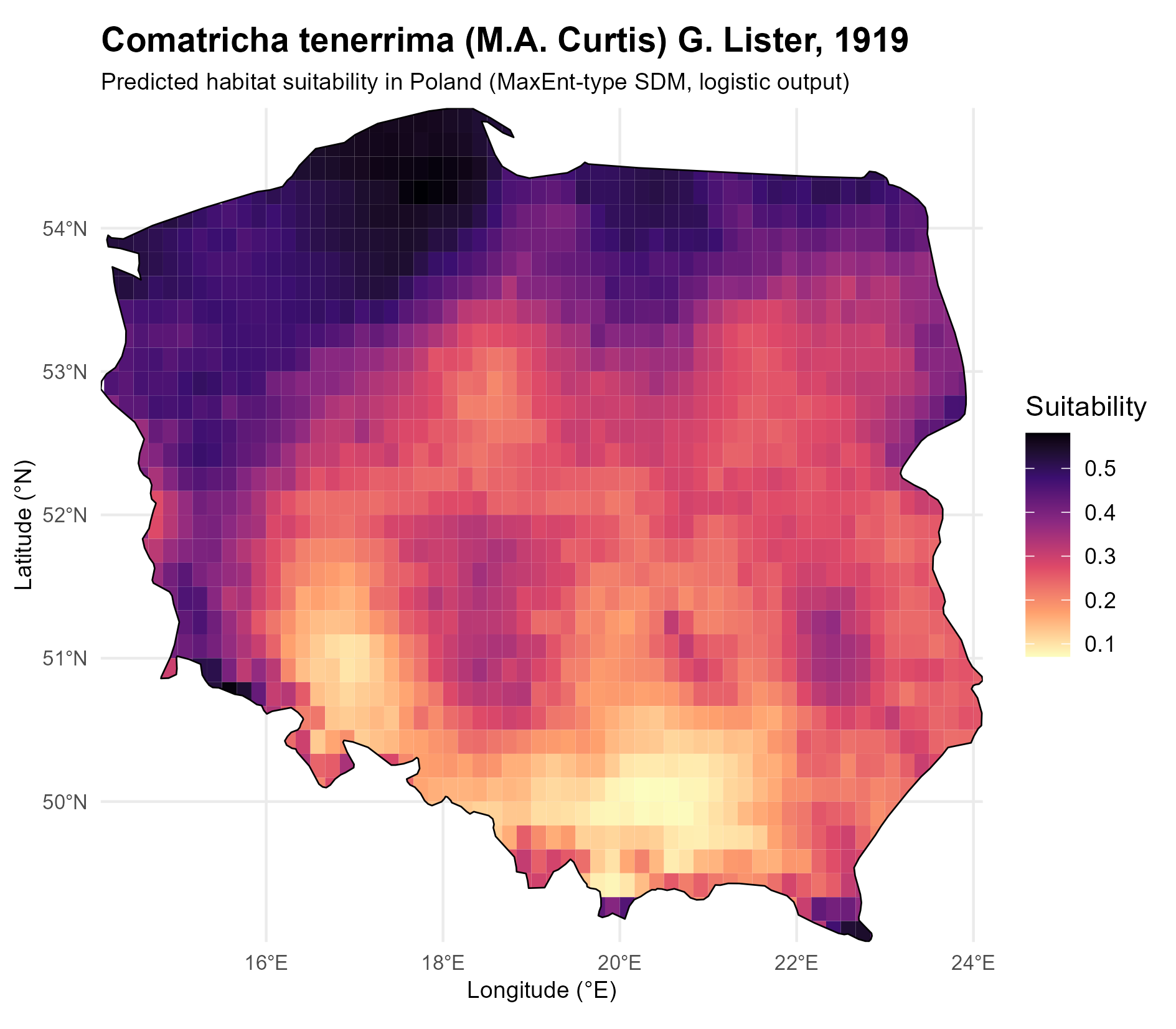

Supplement: Supplemental Information 12 — Set of 101 raster maps showing predicted potential distributions in Poland for modelled candidate species. Each figure displays continuous climatic suitability and the subset of grid cells exceeding a 10th-percentile training presence threshold. [file peerj-14-21492-s012.zip › Figure_SDM_poland_rank024_Comatricha_tenerrima_M_A_Curtis_G_Lister_1919_MaxEnt_logistic.png]

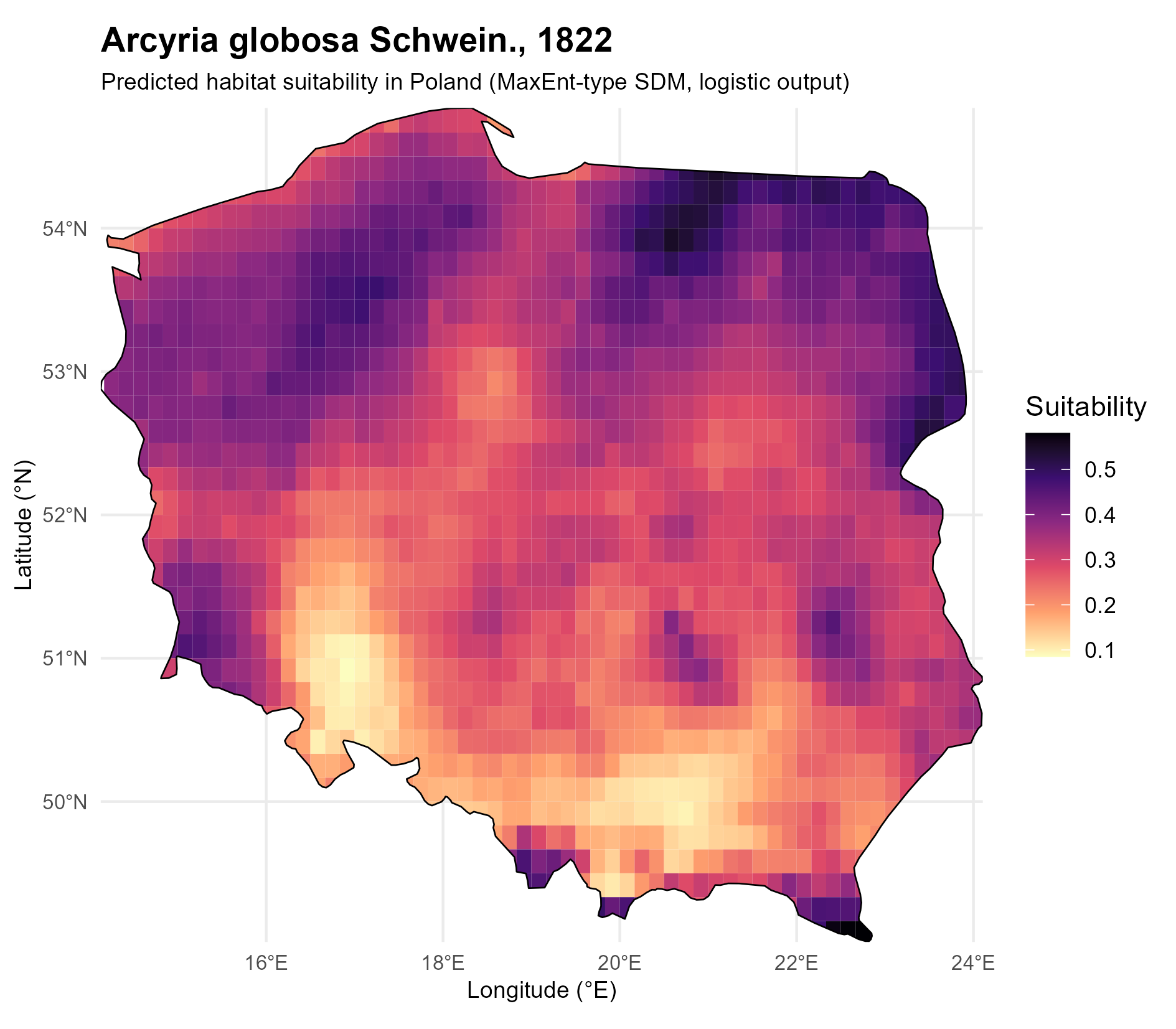

Supplement: Supplemental Information 12 — Set of 101 raster maps showing predicted potential distributions in Poland for modelled candidate species. Each figure displays continuous climatic suitability and the subset of grid cells exceeding a 10th-percentile training presence threshold. [file peerj-14-21492-s012.zip › Figure_SDM_poland_rank023_Arcyria_globosa_Schwein_1822_MaxEnt_logistic.png]

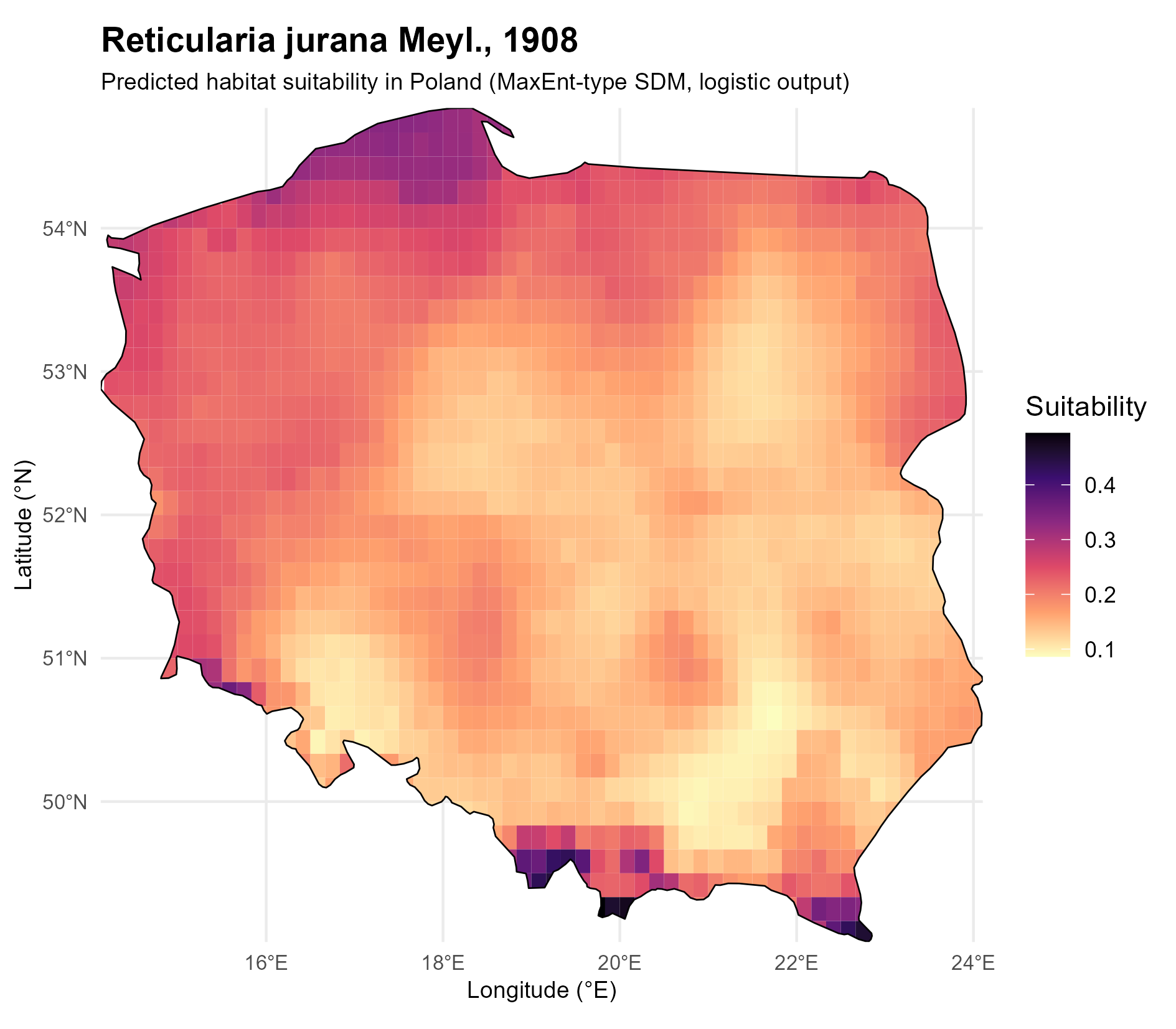

Supplement: Supplemental Information 12 — Set of 101 raster maps showing predicted potential distributions in Poland for modelled candidate species. Each figure displays continuous climatic suitability and the subset of grid cells exceeding a 10th-percentile training presence threshold. [file peerj-14-21492-s012.zip › Figure_SDM_poland_rank022_Reticularia_jurana_Meyl_1908_MaxEnt_logistic.png]

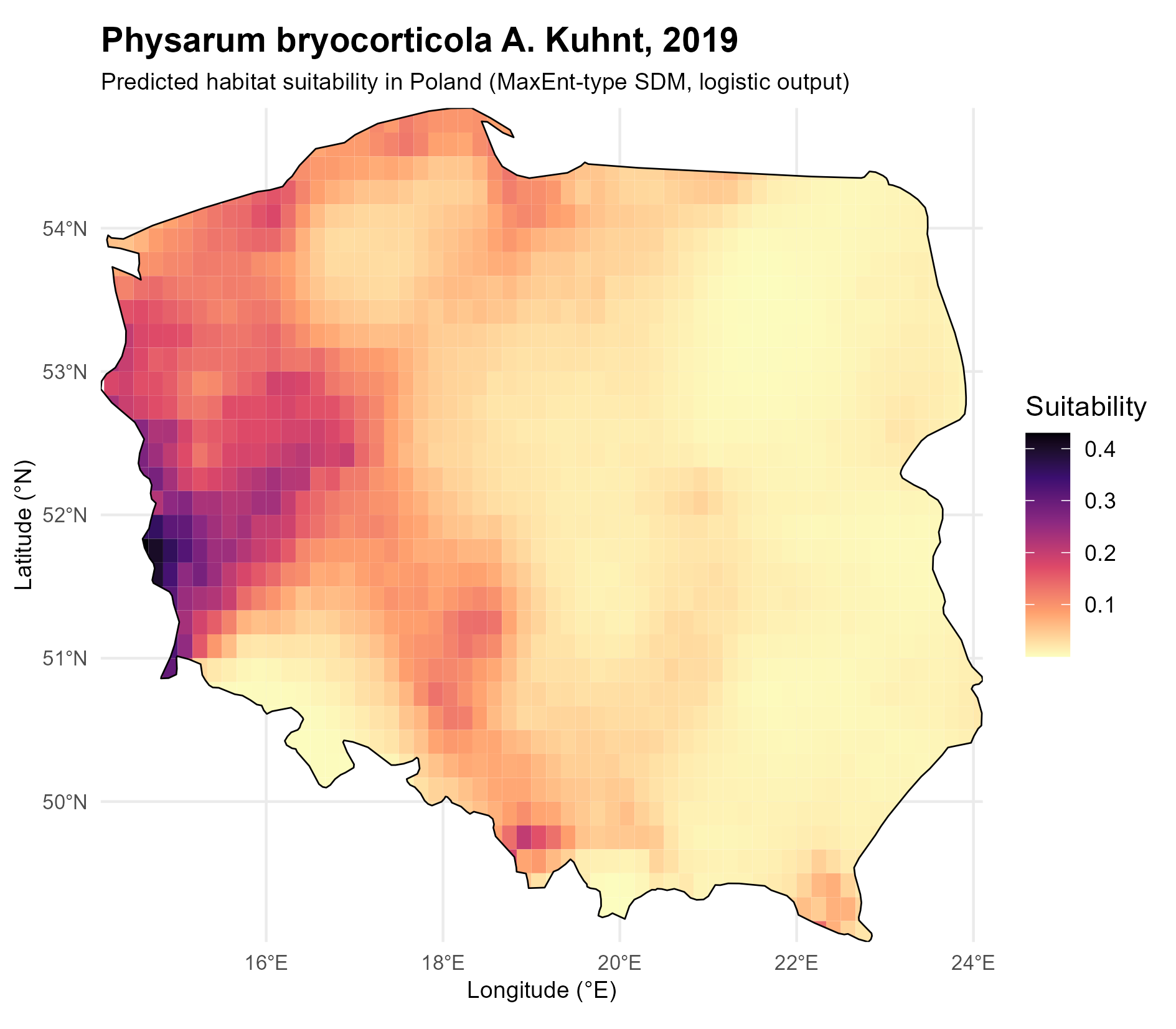

Supplement: Supplemental Information 12 — Set of 101 raster maps showing predicted potential distributions in Poland for modelled candidate species. Each figure displays continuous climatic suitability and the subset of grid cells exceeding a 10th-percentile training presence threshold. [file peerj-14-21492-s012.zip › Figure_SDM_poland_rank021_Physarum_bryocorticola_A_Kuhnt_2019_MaxEnt_logistic.png]

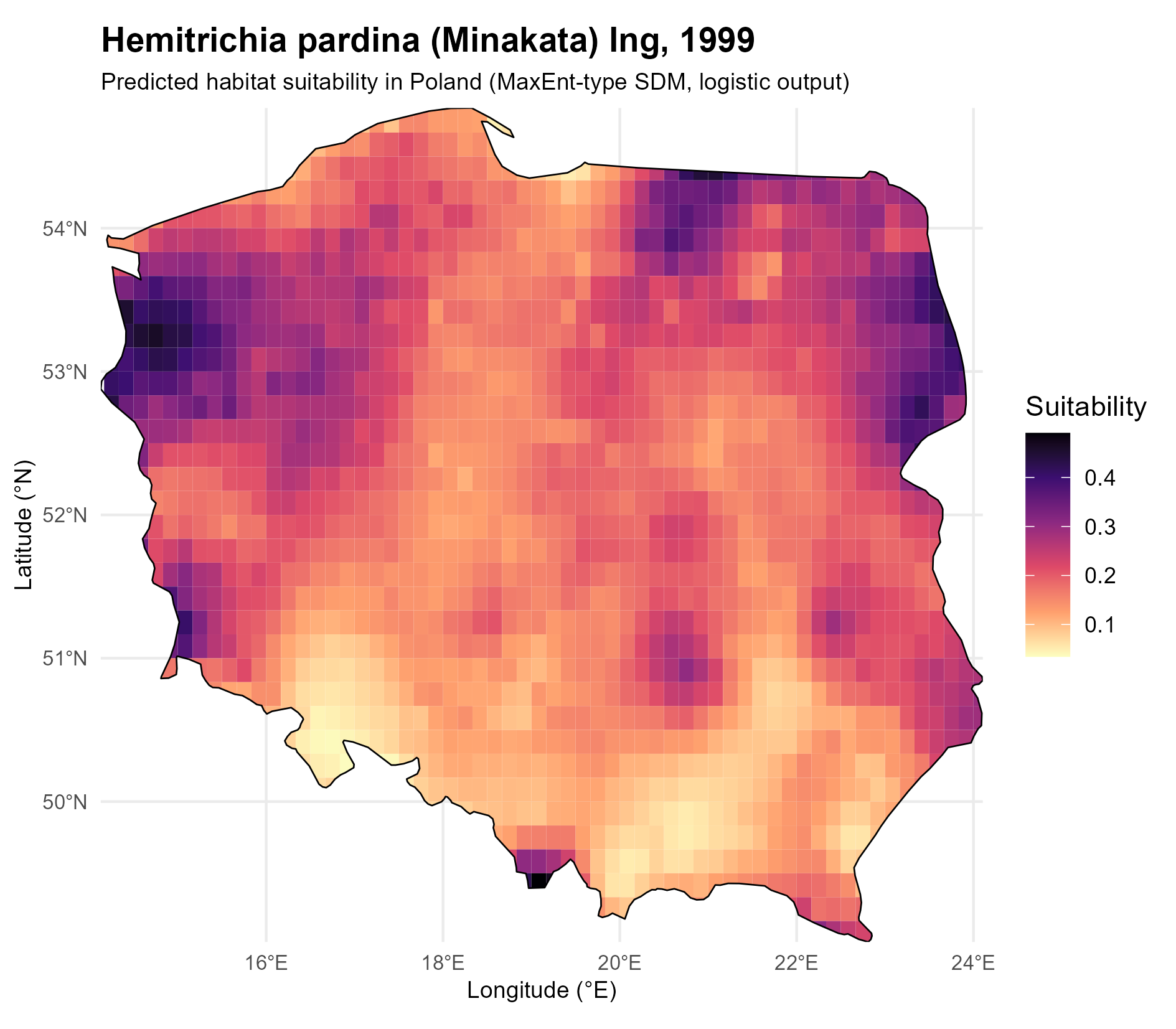

Supplement: Supplemental Information 12 — Set of 101 raster maps showing predicted potential distributions in Poland for modelled candidate species. Each figure displays continuous climatic suitability and the subset of grid cells exceeding a 10th-percentile training presence threshold. [file peerj-14-21492-s012.zip › Figure_SDM_poland_rank020_Hemitrichia_pardina_Minakata_Ing_1999_MaxEnt_logistic.png]

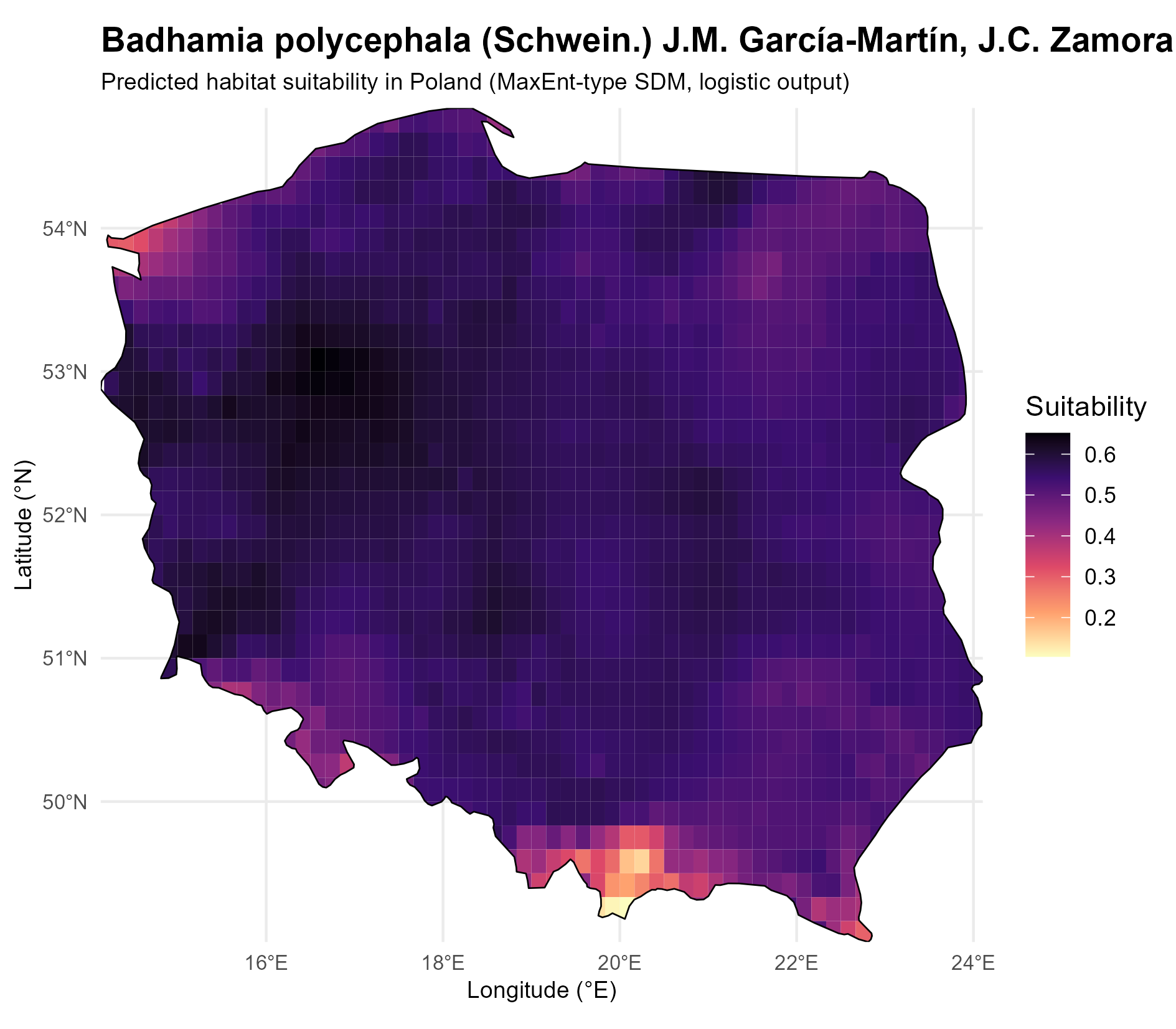

Supplement: Supplemental Information 12 — Set of 101 raster maps showing predicted potential distributions in Poland for modelled candidate species. Each figure displays continuous climatic suitability and the subset of grid cells exceeding a 10th-percentile training presence threshold. [file peerj-14-21492-s012.zip › Figure_SDM_poland_rank019_Badhamia_polycephala_Schwein_J_M_Garcia_Martin_J_C_Zamora_Lado_2023_MaxEnt_logistic.png]

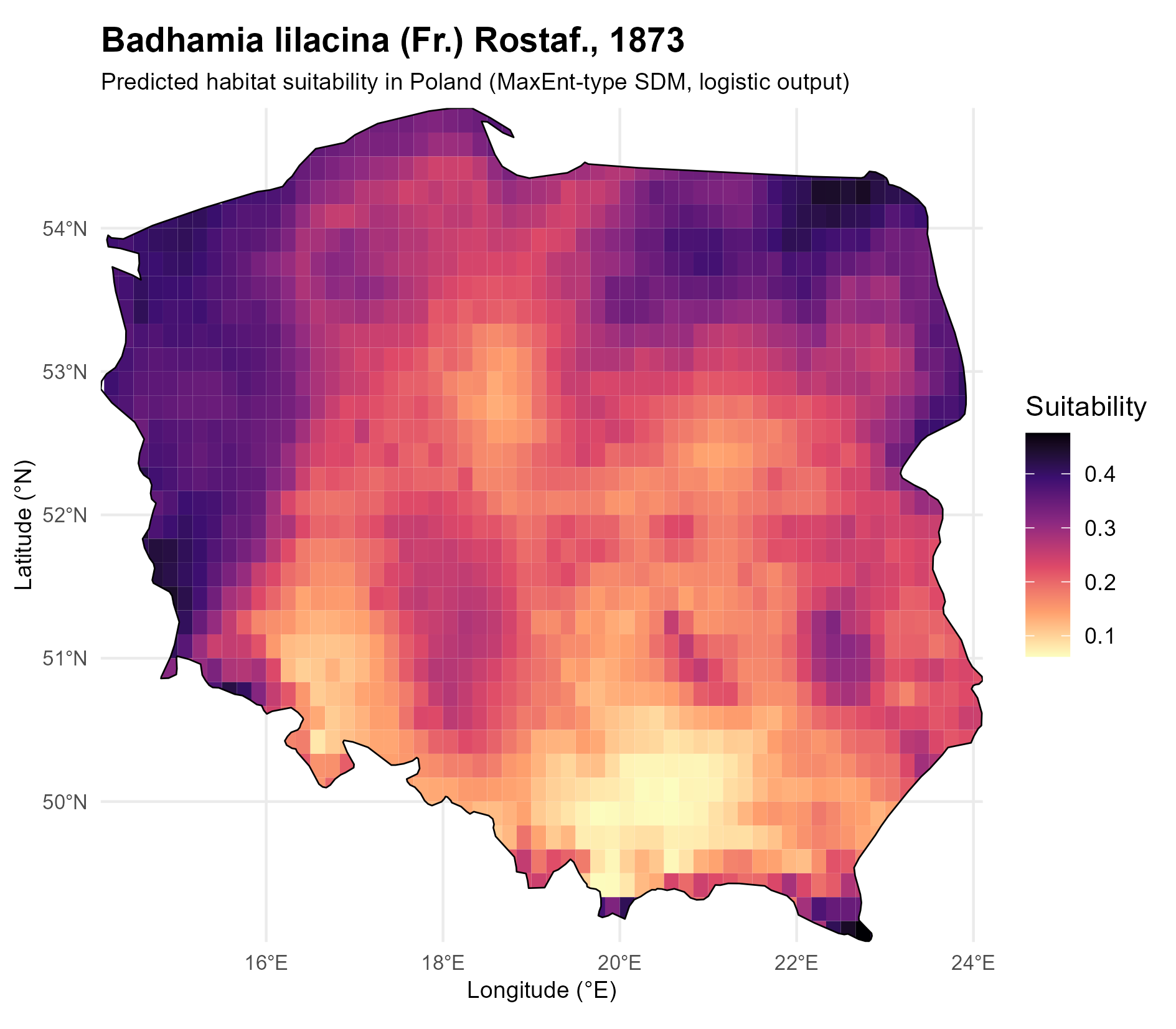

Supplement: Supplemental Information 12 — Set of 101 raster maps showing predicted potential distributions in Poland for modelled candidate species. Each figure displays continuous climatic suitability and the subset of grid cells exceeding a 10th-percentile training presence threshold. [file peerj-14-21492-s012.zip › Figure_SDM_poland_rank018_Badhamia_lilacina_Fr_Rostaf_1873_MaxEnt_logistic.png]

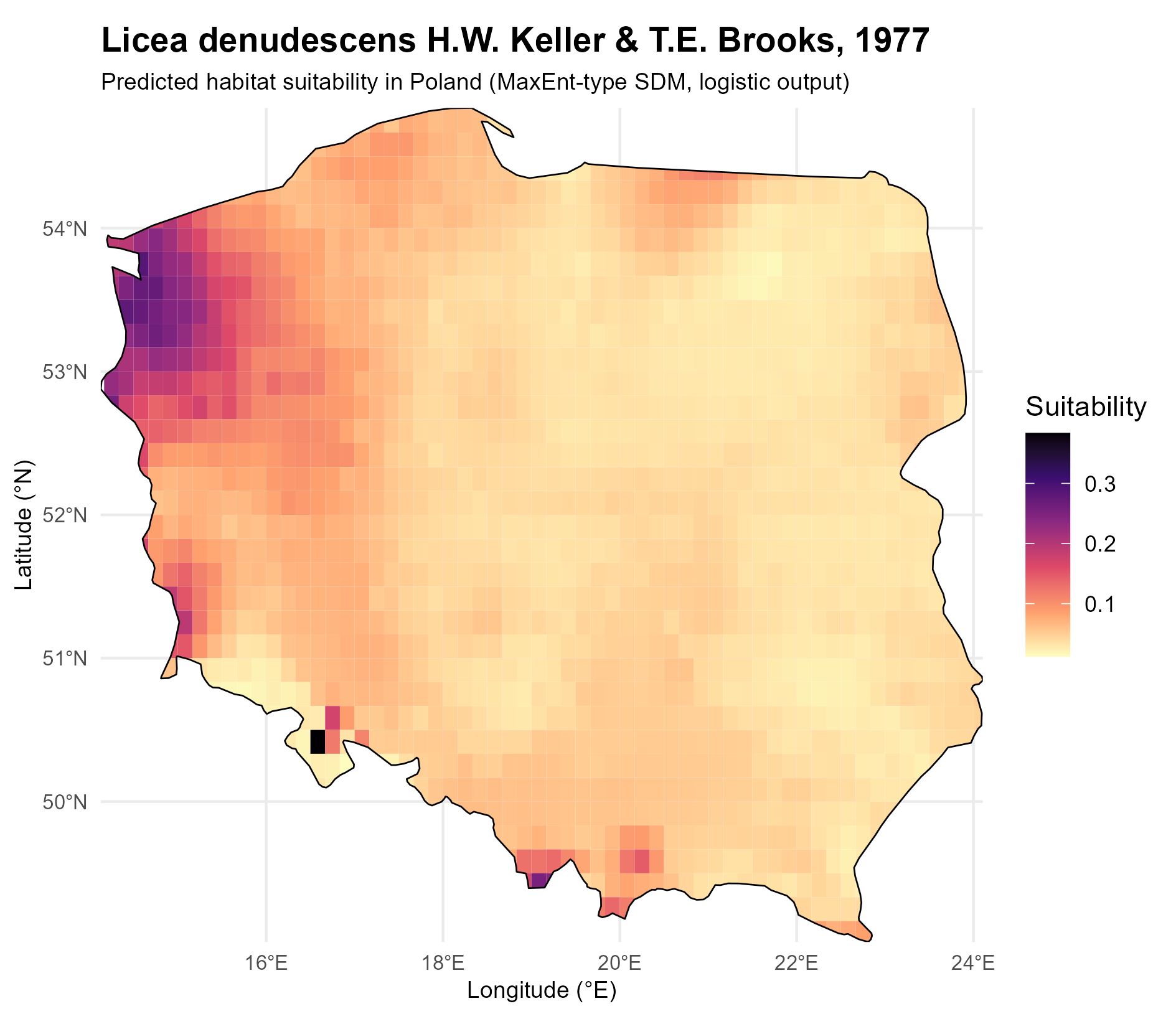

Supplement: Supplemental Information 12 — Set of 101 raster maps showing predicted potential distributions in Poland for modelled candidate species. Each figure displays continuous climatic suitability and the subset of grid cells exceeding a 10th-percentile training presence threshold. [file peerj-14-21492-s012.zip › Figure_SDM_poland_rank017_Licea_denudescens_H_W_Keller_T_E_Brooks_1977_MaxEnt_logistic.png]

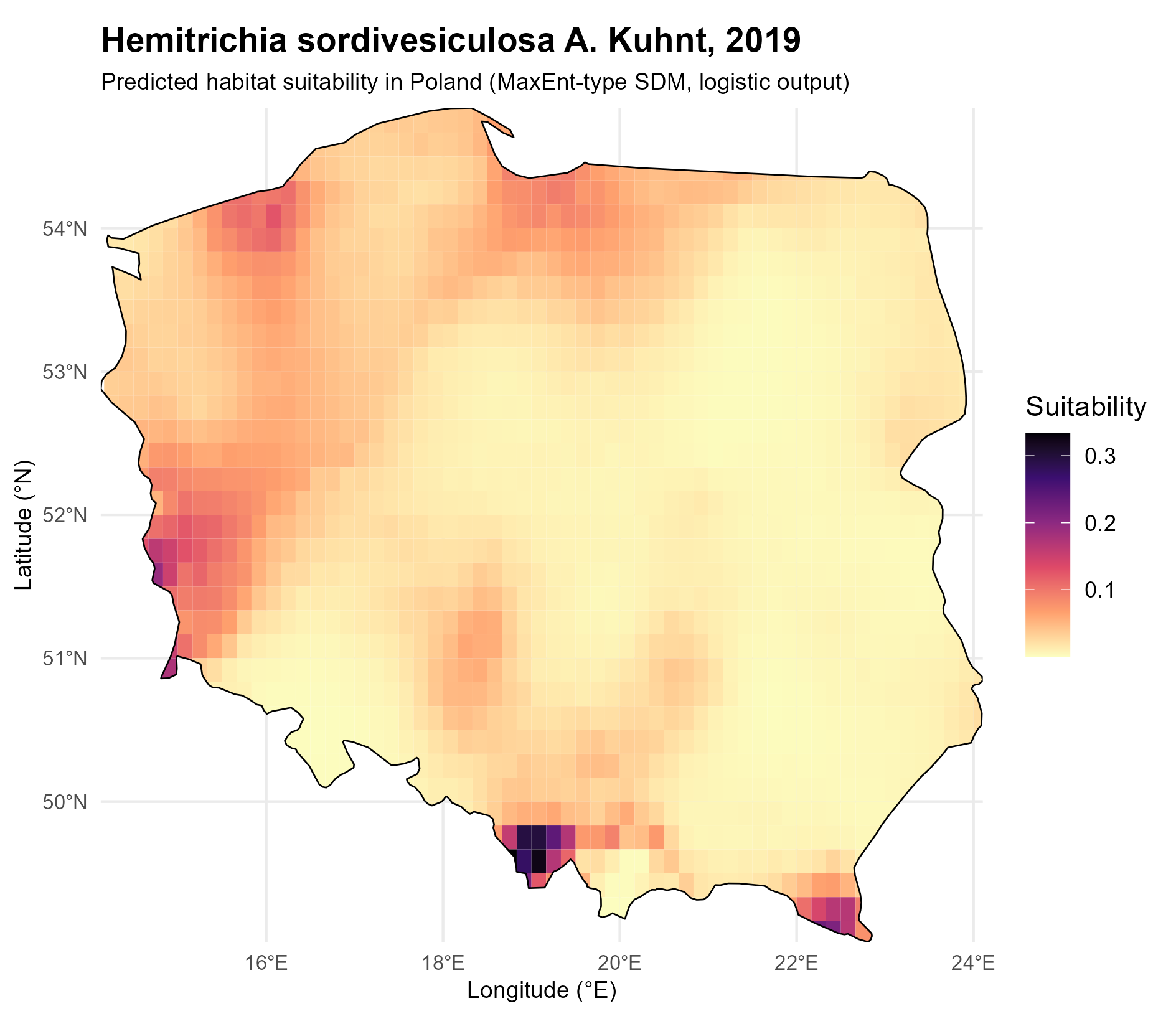

Supplement: Supplemental Information 12 — Set of 101 raster maps showing predicted potential distributions in Poland for modelled candidate species. Each figure displays continuous climatic suitability and the subset of grid cells exceeding a 10th-percentile training presence threshold. [file peerj-14-21492-s012.zip › Figure_SDM_poland_rank016_Hemitrichia_sordivesiculosa_A_Kuhnt_2019_MaxEnt_logistic.png]

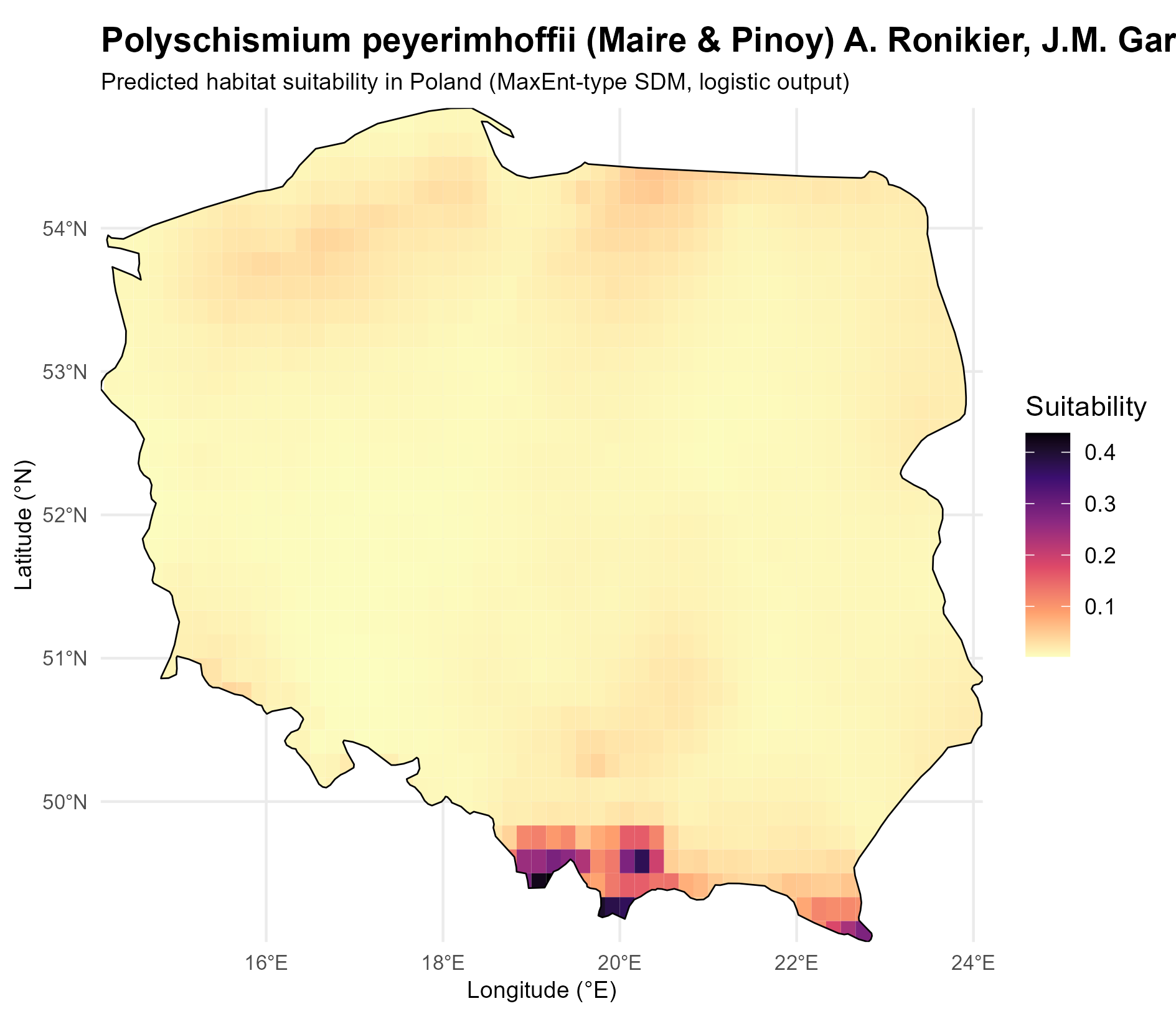

Supplement: Supplemental Information 12 — Set of 101 raster maps showing predicted potential distributions in Poland for modelled candidate species. Each figure displays continuous climatic suitability and the subset of grid cells exceeding a 10th-percentile training presence threshold. [file peerj-14-21492-s012.zip › Figure_SDM_poland_rank015_Polyschismium_peyerimhoffii_Maire_Pinoy_A_Ronikier_J_M_Garcia_Martin_A_Kuhnt_J_C_Zamora_M_de_Haan_Janik_Lado_2022_MaxEnt_logistic.png]

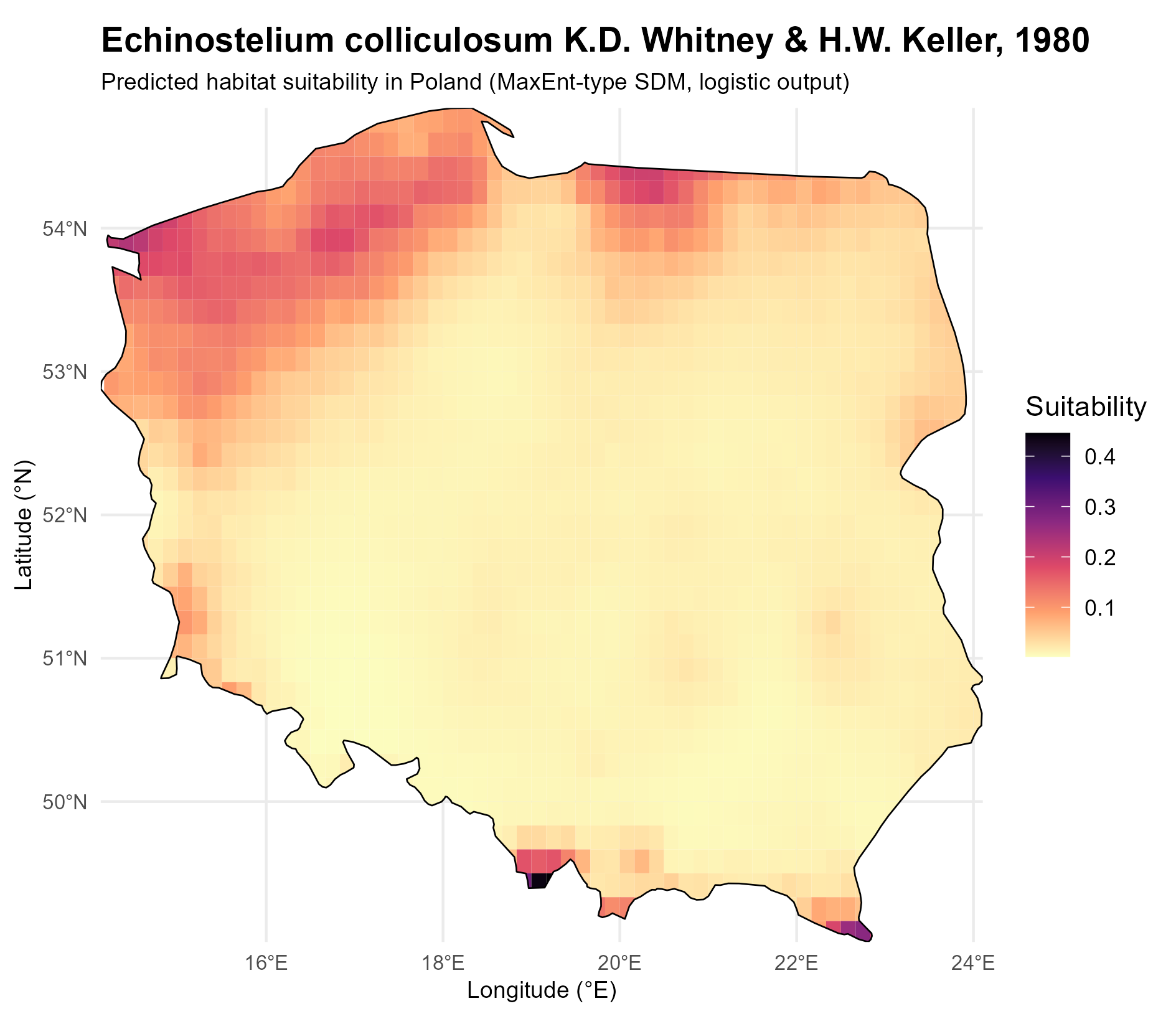

Supplement: Supplemental Information 12 — Set of 101 raster maps showing predicted potential distributions in Poland for modelled candidate species. Each figure displays continuous climatic suitability and the subset of grid cells exceeding a 10th-percentile training presence threshold. [file peerj-14-21492-s012.zip › Figure_SDM_poland_rank014_Echinostelium_colliculosum_K_D_Whitney_H_W_Keller_1980_MaxEnt_logistic.png]

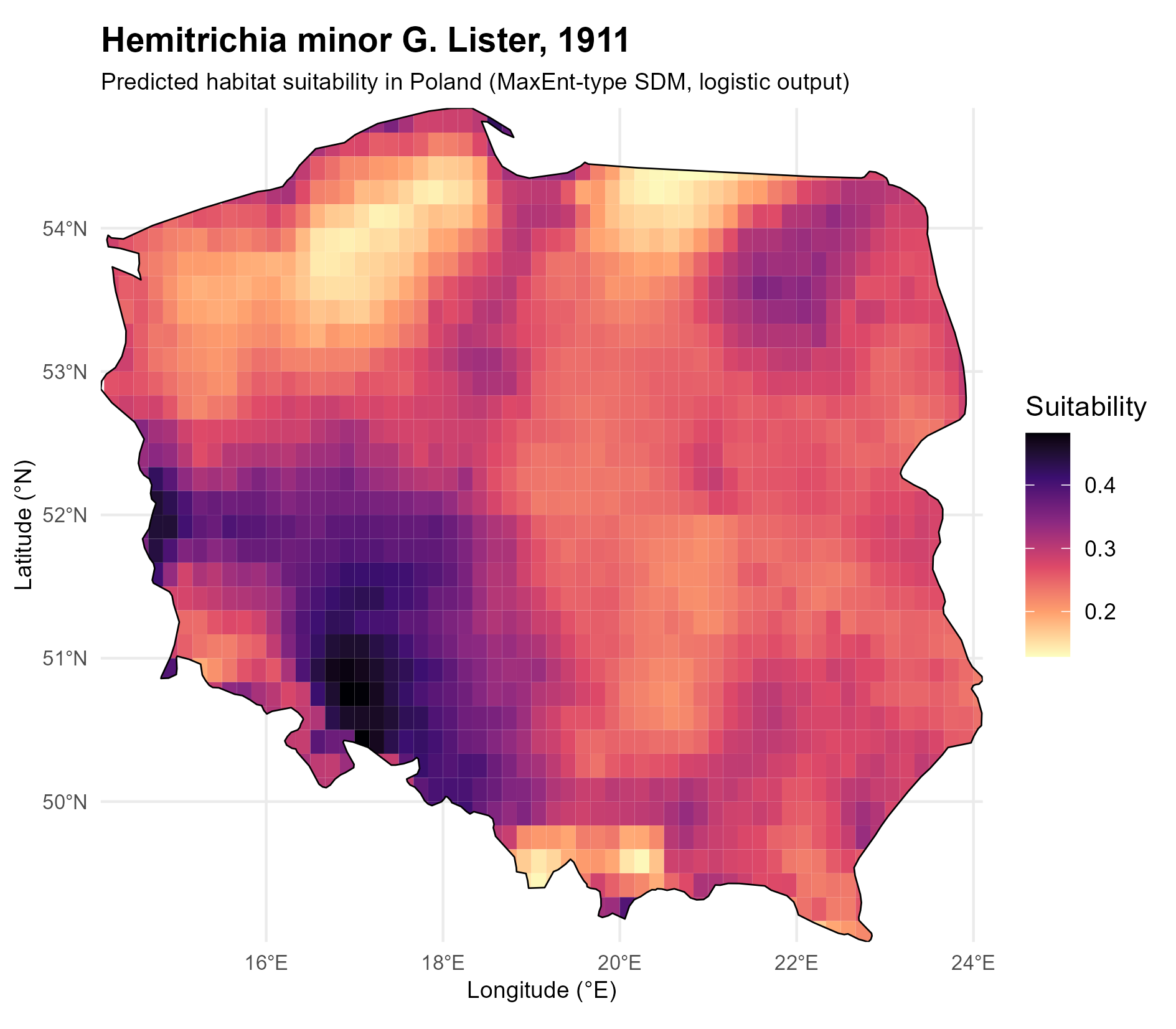

Supplement: Supplemental Information 12 — Set of 101 raster maps showing predicted potential distributions in Poland for modelled candidate species. Each figure displays continuous climatic suitability and the subset of grid cells exceeding a 10th-percentile training presence threshold. [file peerj-14-21492-s012.zip › Figure_SDM_poland_rank013_Hemitrichia_minor_G_Lister_1911_MaxEnt_logistic.png]

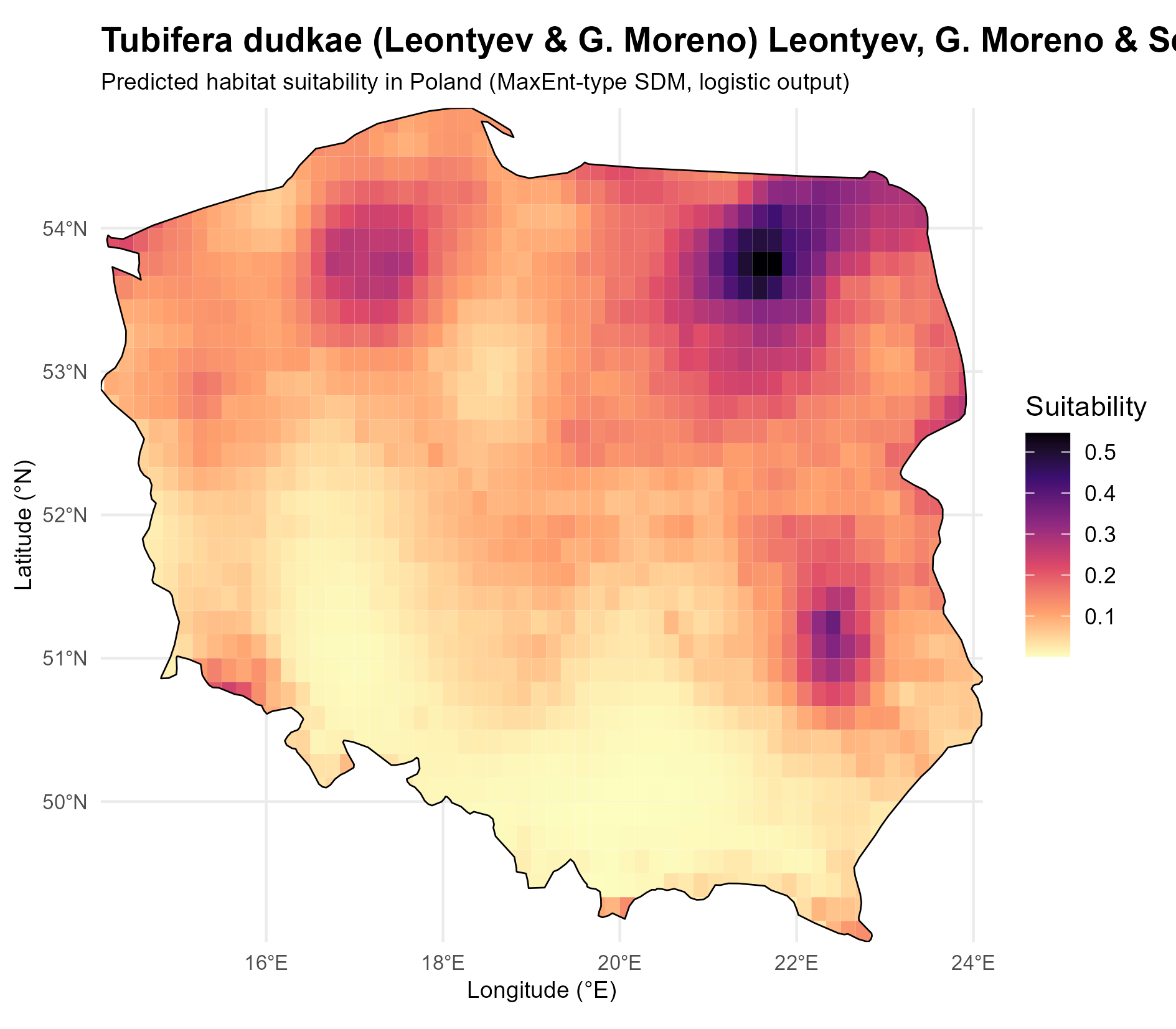

Supplement: Supplemental Information 12 — Set of 101 raster maps showing predicted potential distributions in Poland for modelled candidate species. Each figure displays continuous climatic suitability and the subset of grid cells exceeding a 10th-percentile training presence threshold. [file peerj-14-21492-s012.zip › Figure_SDM_poland_rank012_Tubifera_dudkae_Leontyev_G_Moreno_Leontyev_G_Moreno_Schnittler_2015_MaxEnt_logistic.png]

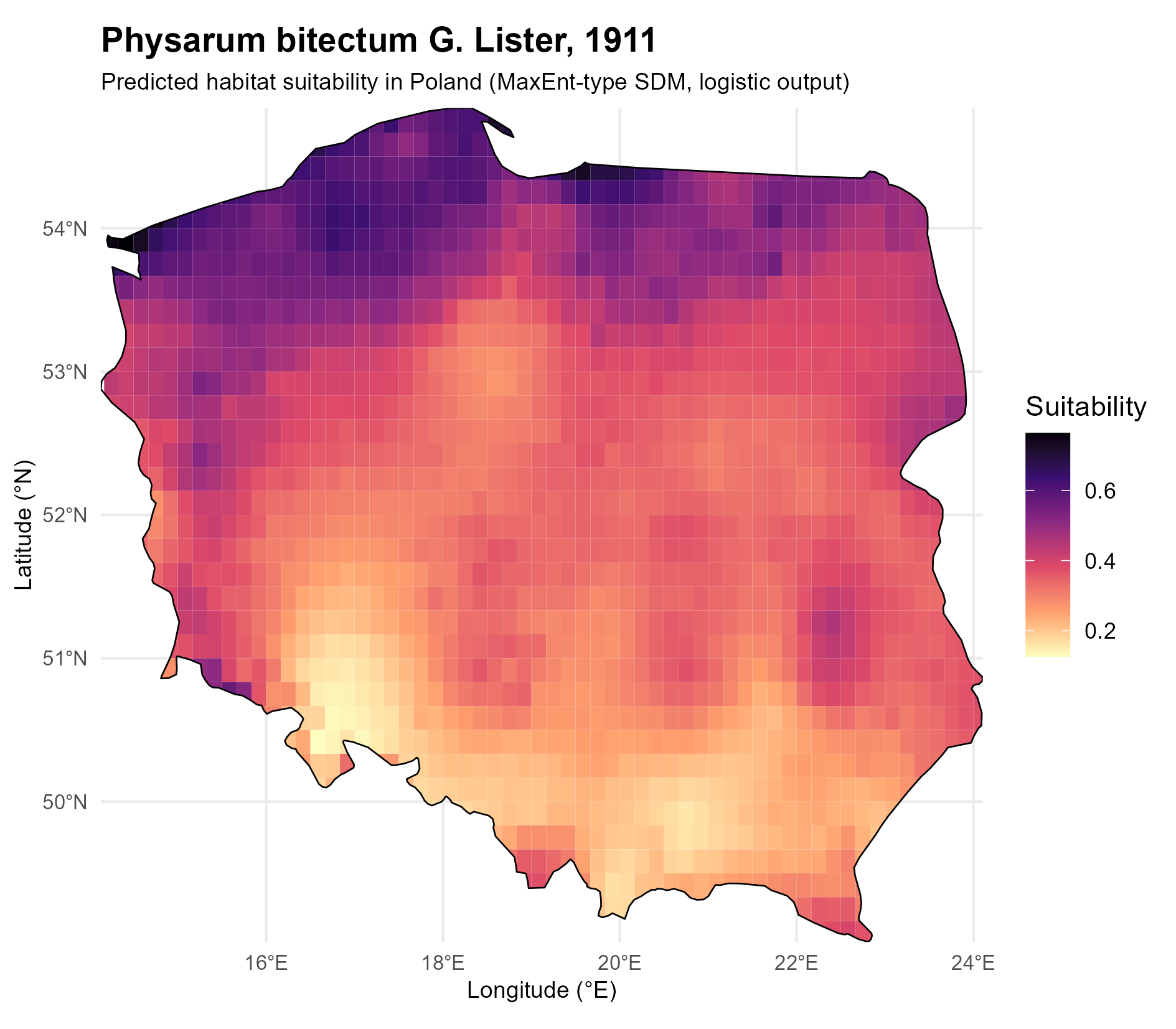

Supplement: Supplemental Information 12 — Set of 101 raster maps showing predicted potential distributions in Poland for modelled candidate species. Each figure displays continuous climatic suitability and the subset of grid cells exceeding a 10th-percentile training presence threshold. [file peerj-14-21492-s012.zip › Figure_SDM_poland_rank011_Physarum_bitectum_G_Lister_1911_MaxEnt_logistic.png]

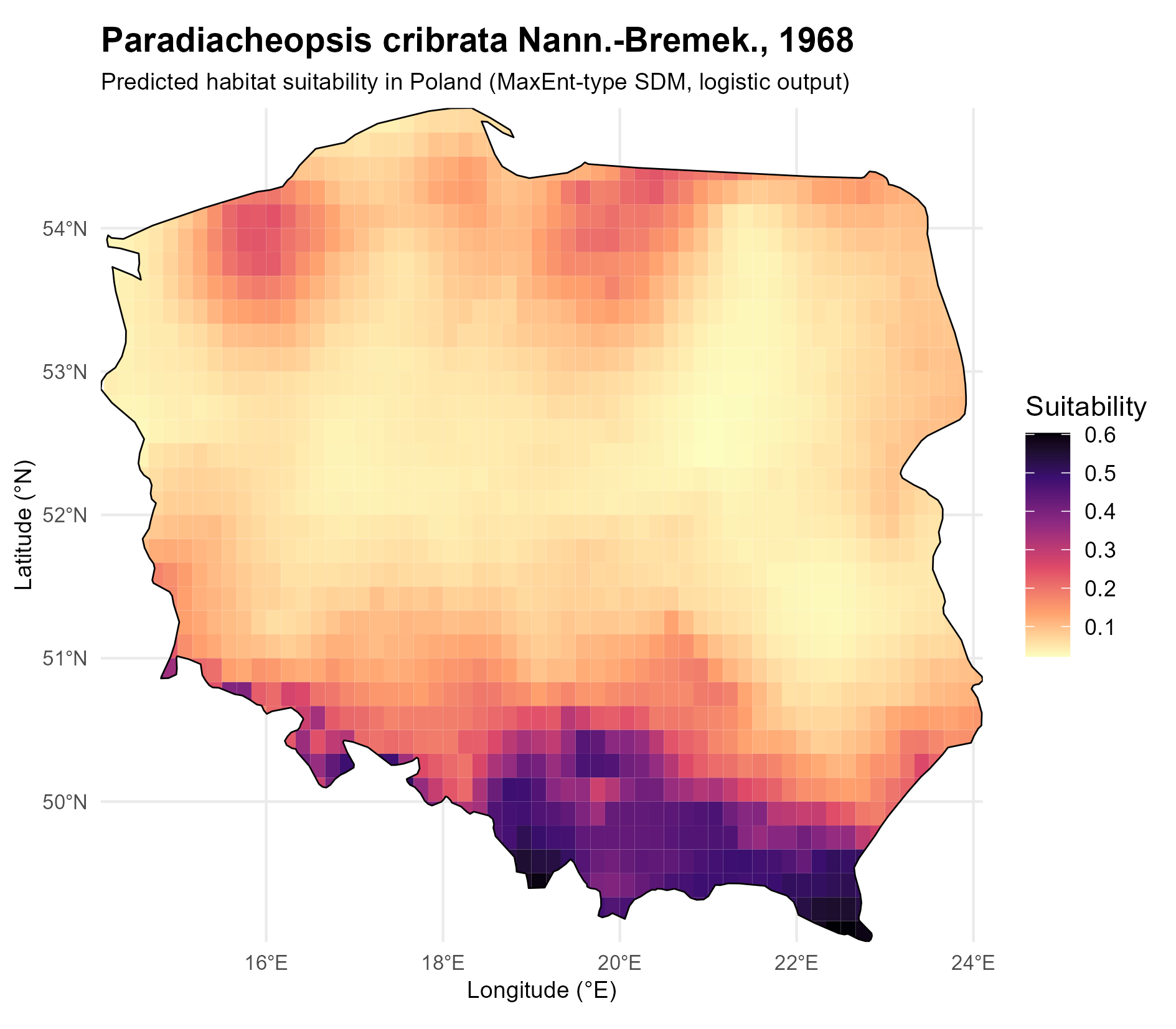

Supplement: Supplemental Information 12 — Set of 101 raster maps showing predicted potential distributions in Poland for modelled candidate species. Each figure displays continuous climatic suitability and the subset of grid cells exceeding a 10th-percentile training presence threshold. [file peerj-14-21492-s012.zip › Figure_SDM_poland_rank010_Paradiacheopsis_cribrata_Nann_Bremek_1968_MaxEnt_logistic.png]

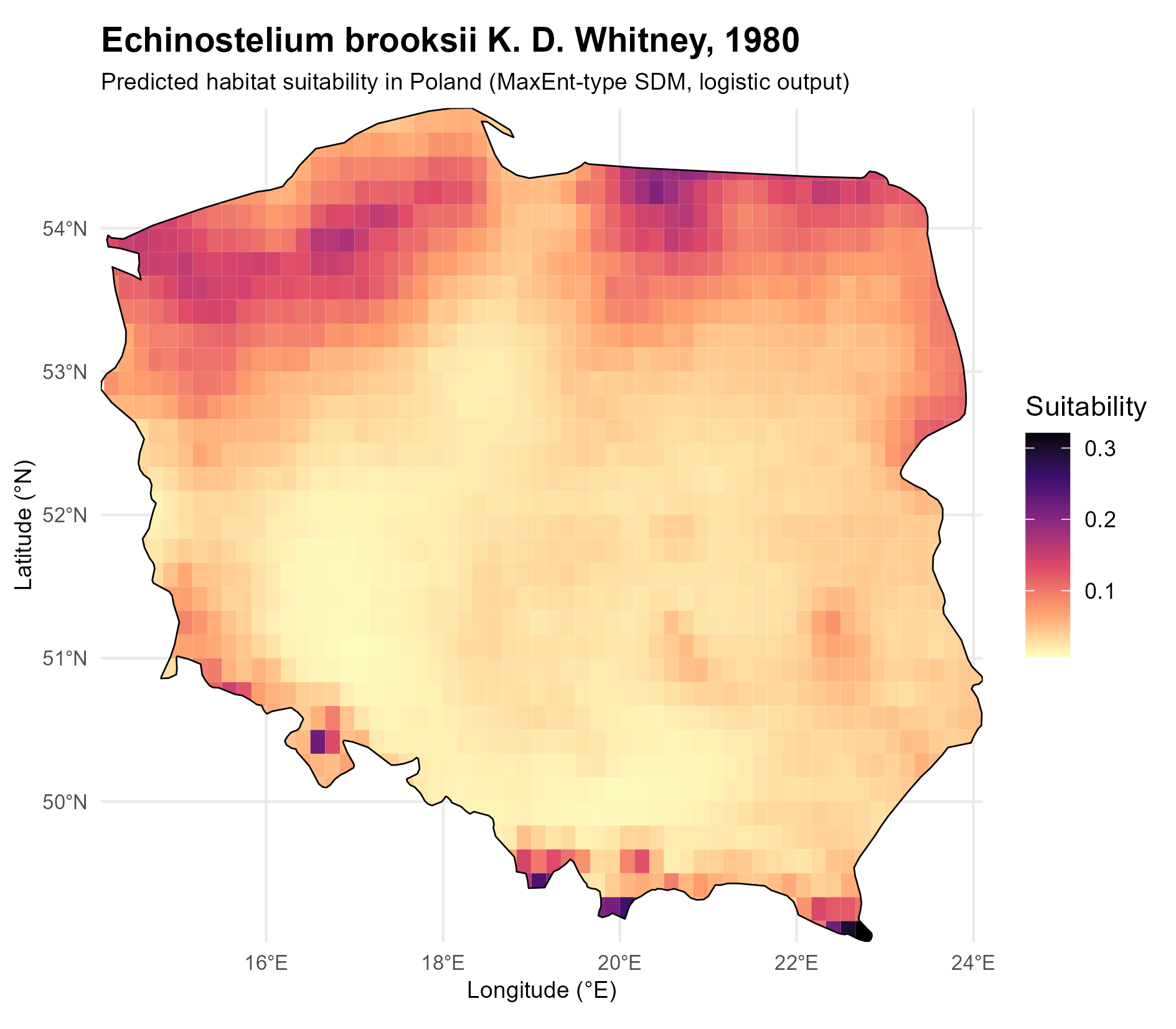

Supplement: Supplemental Information 12 — Set of 101 raster maps showing predicted potential distributions in Poland for modelled candidate species. Each figure displays continuous climatic suitability and the subset of grid cells exceeding a 10th-percentile training presence threshold. [file peerj-14-21492-s012.zip › Figure_SDM_poland_rank009_Echinostelium_brooksii_K_D_Whitney_1980_MaxEnt_logistic.png]

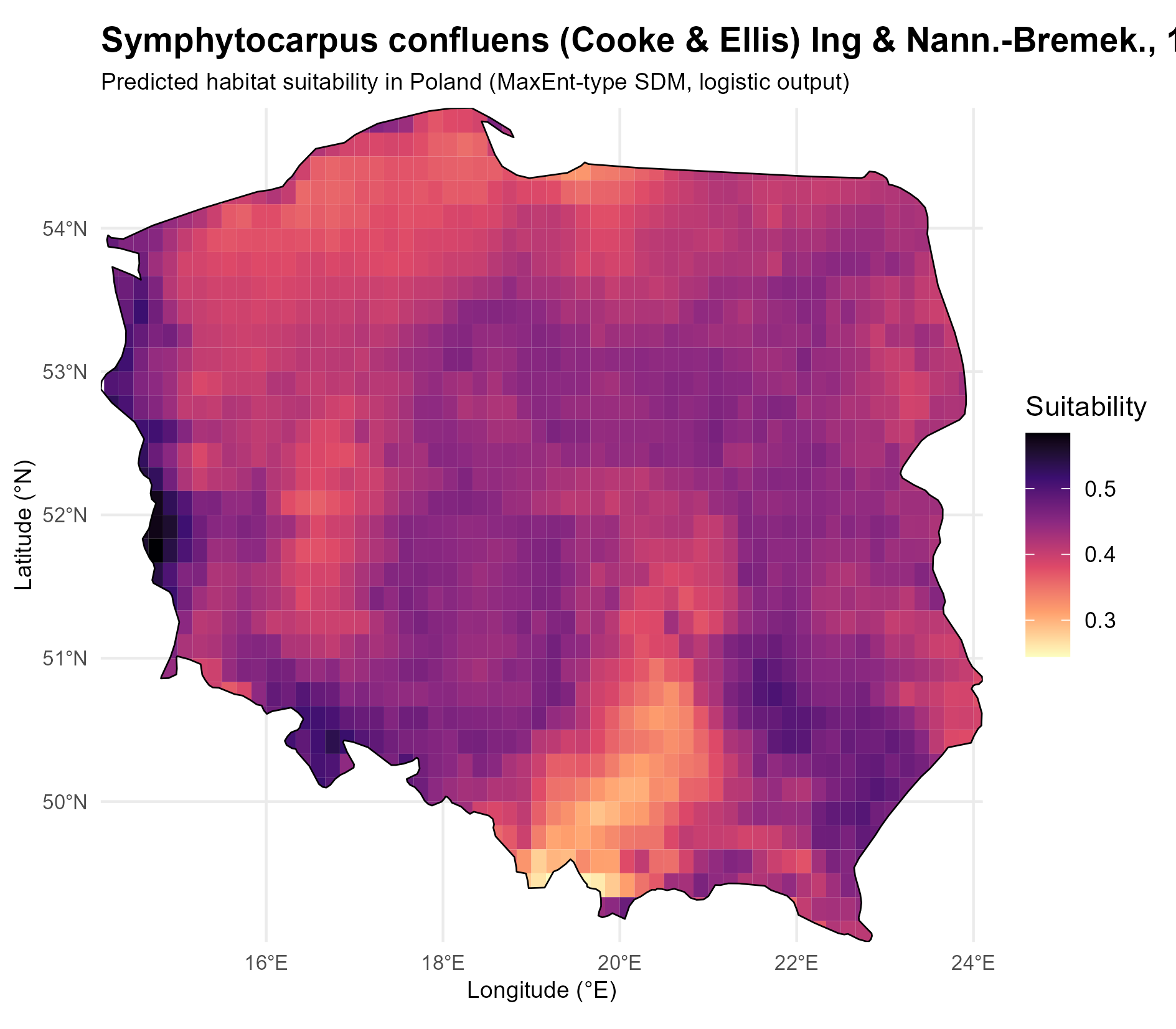

Supplement: Supplemental Information 12 — Set of 101 raster maps showing predicted potential distributions in Poland for modelled candidate species. Each figure displays continuous climatic suitability and the subset of grid cells exceeding a 10th-percentile training presence threshold. [file peerj-14-21492-s012.zip › Figure_SDM_poland_rank008_Symphytocarpus_confluens_Cooke_Ellis_Ing_Nann_Bremek_1975_MaxEnt_logistic.png]

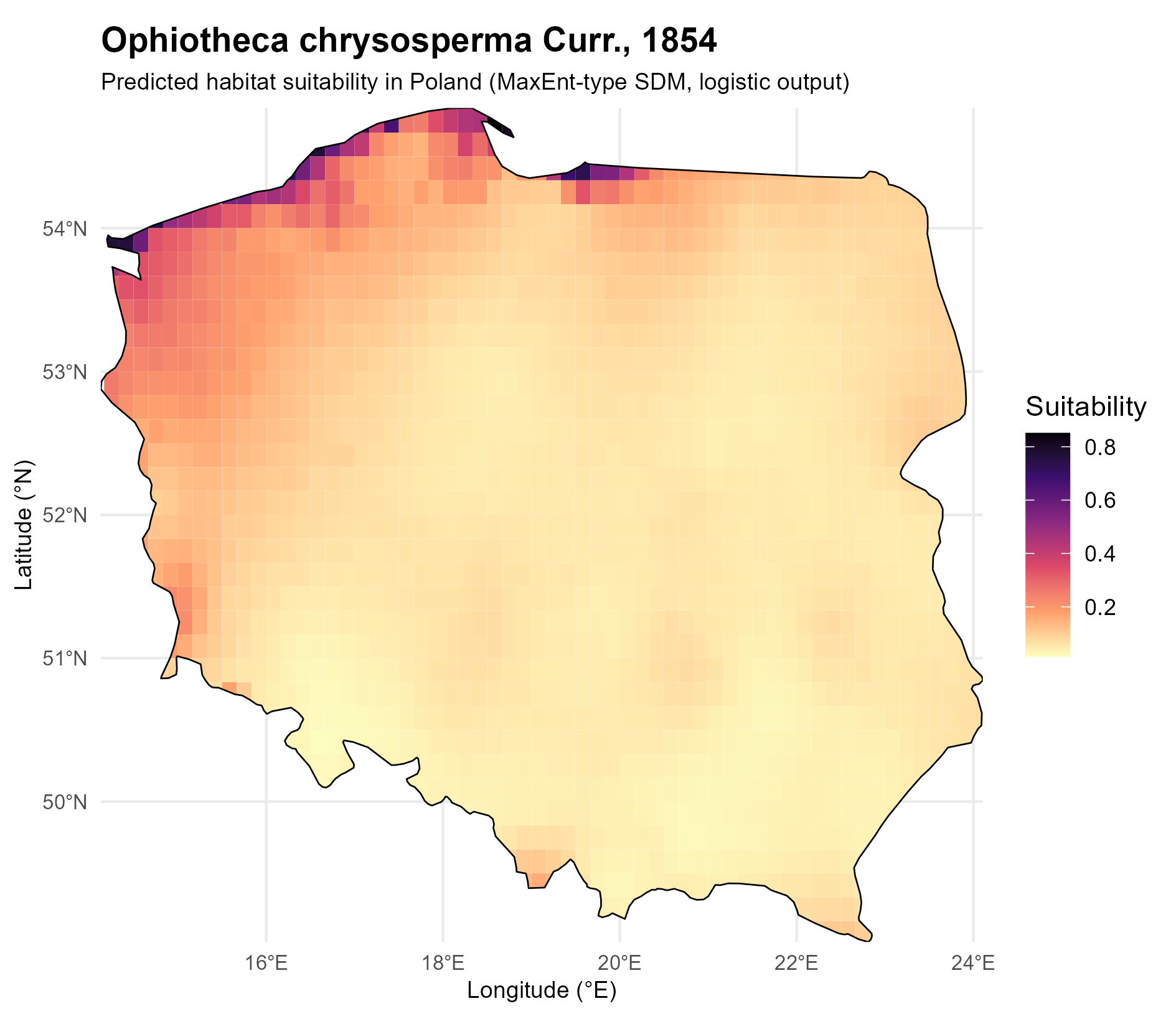

Supplement: Supplemental Information 12 — Set of 101 raster maps showing predicted potential distributions in Poland for modelled candidate species. Each figure displays continuous climatic suitability and the subset of grid cells exceeding a 10th-percentile training presence threshold. [file peerj-14-21492-s012.zip › Figure_SDM_poland_rank007_Ophiotheca_chrysosperma_Curr_1854_MaxEnt_logistic.png]

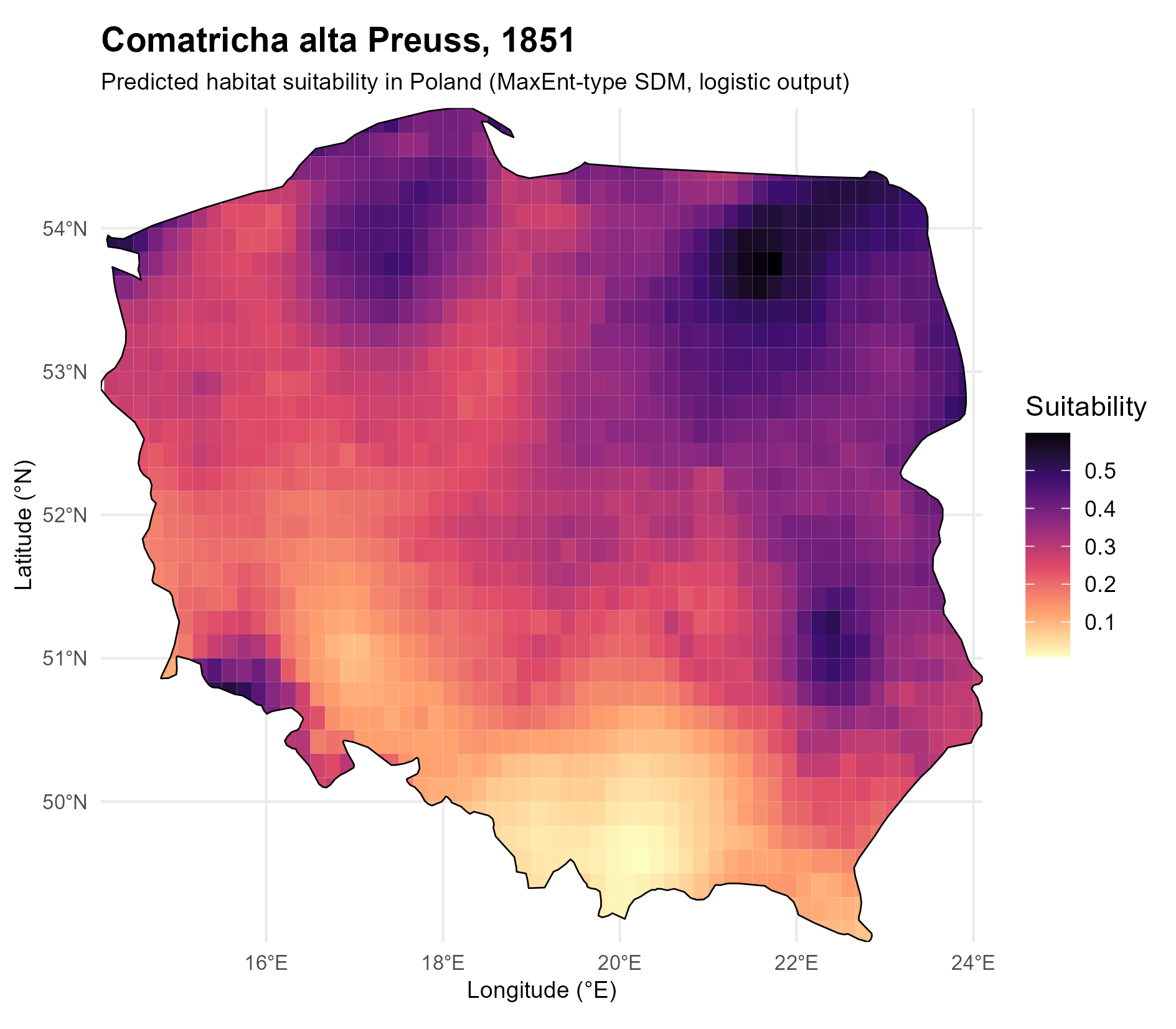

Supplement: Supplemental Information 12 — Set of 101 raster maps showing predicted potential distributions in Poland for modelled candidate species. Each figure displays continuous climatic suitability and the subset of grid cells exceeding a 10th-percentile training presence threshold. [file peerj-14-21492-s012.zip › Figure_SDM_poland_rank006_Comatricha_alta_Preuss_1851_MaxEnt_logistic.png]

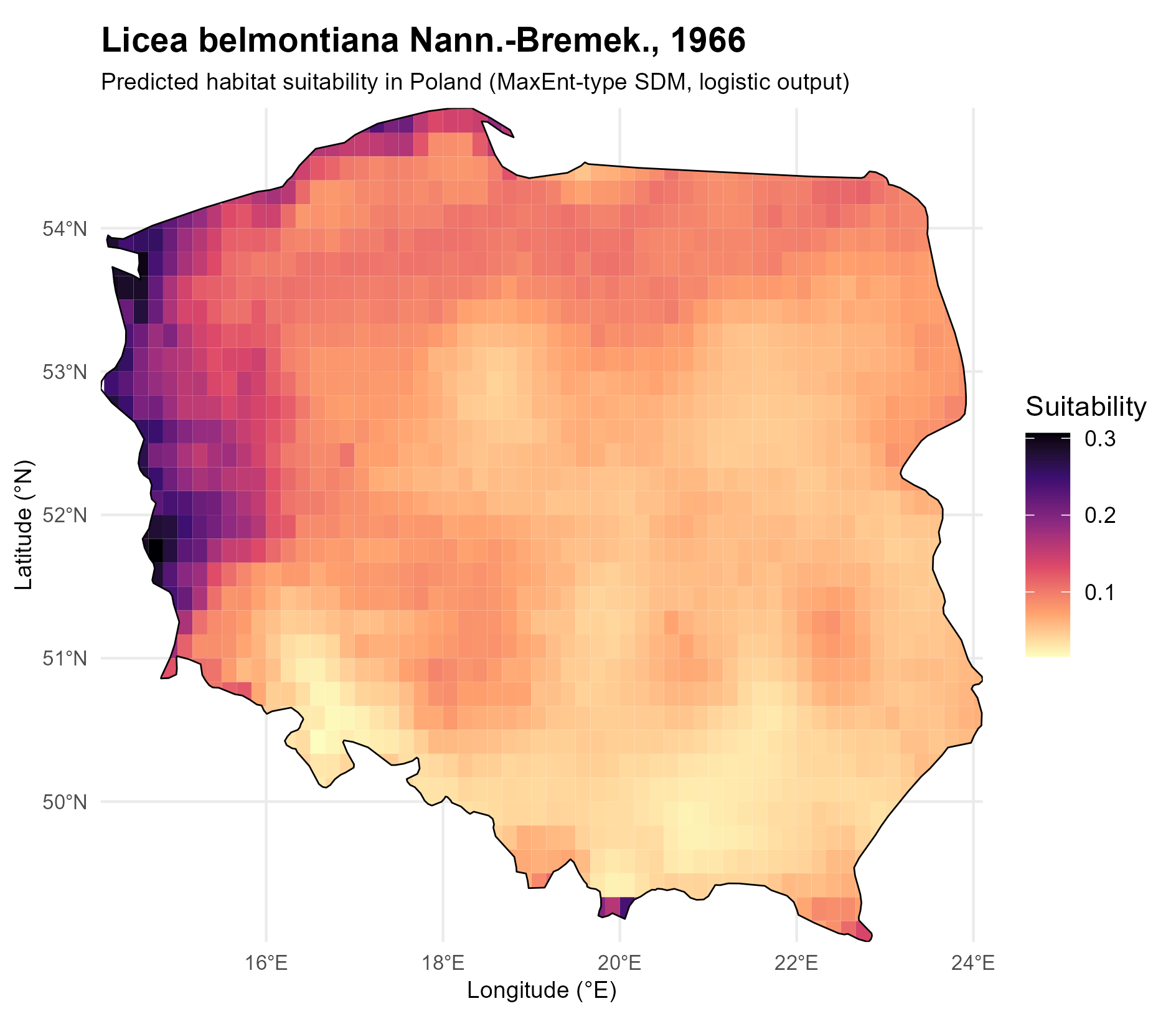

Supplement: Supplemental Information 12 — Set of 101 raster maps showing predicted potential distributions in Poland for modelled candidate species. Each figure displays continuous climatic suitability and the subset of grid cells exceeding a 10th-percentile training presence threshold. [file peerj-14-21492-s012.zip › Figure_SDM_poland_rank005_Licea_belmontiana_Nann_Bremek_1966_MaxEnt_logistic.png]

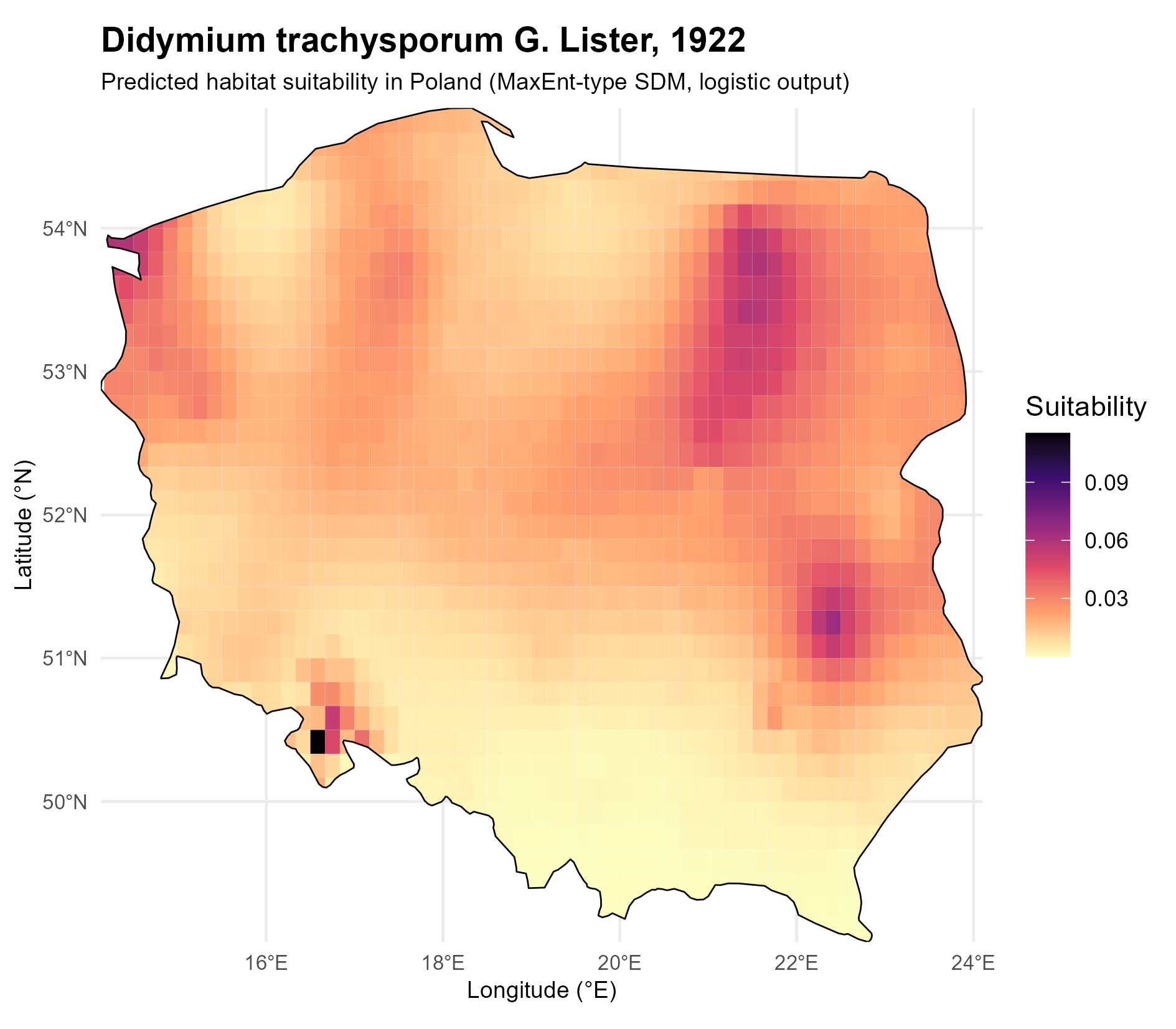

Supplement: Supplemental Information 12 — Set of 101 raster maps showing predicted potential distributions in Poland for modelled candidate species. Each figure displays continuous climatic suitability and the subset of grid cells exceeding a 10th-percentile training presence threshold. [file peerj-14-21492-s012.zip › Figure_SDM_poland_rank004_Didymium_trachysporum_G_Lister_1922_MaxEnt_logistic.png]

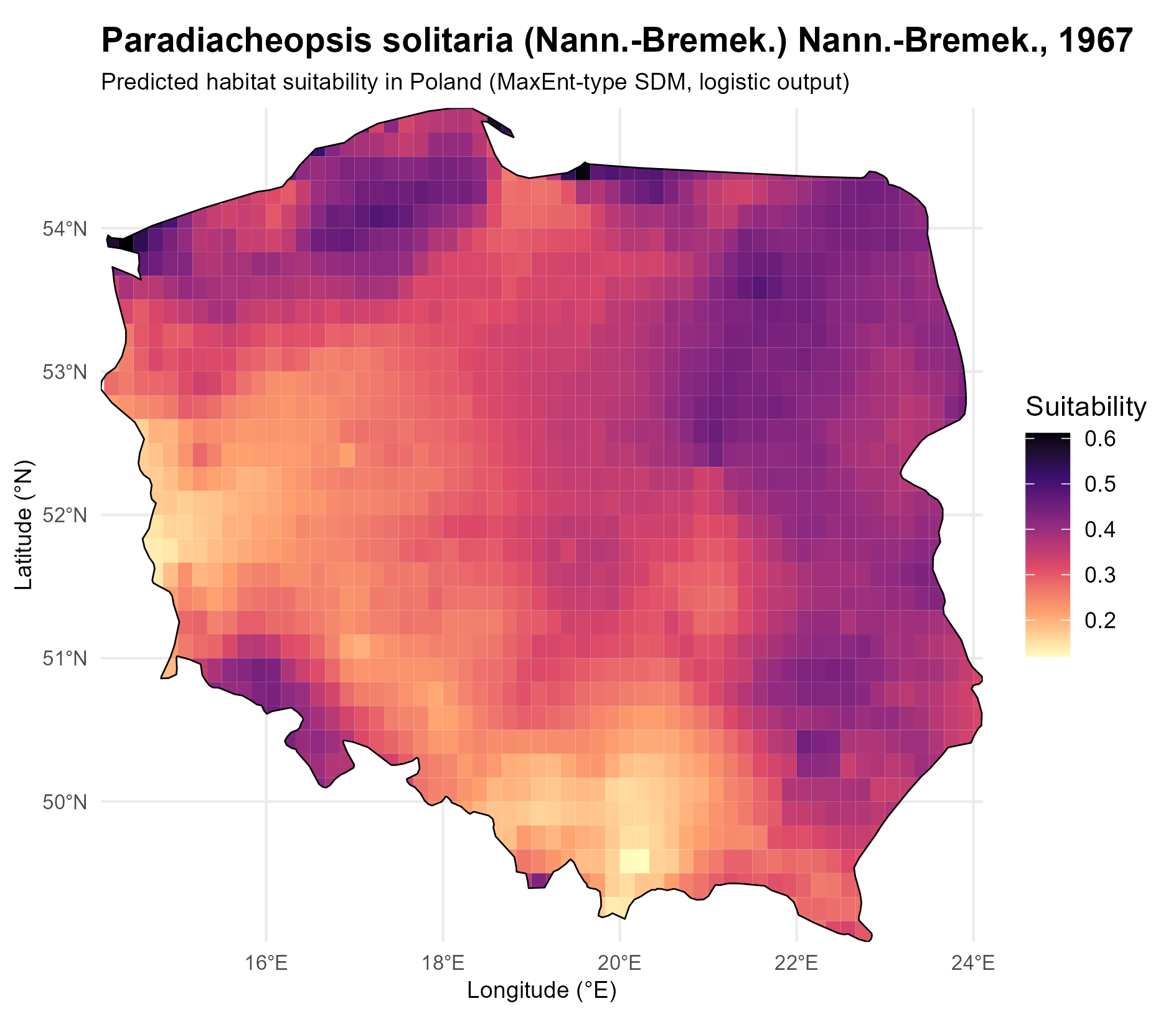

Supplement: Supplemental Information 12 — Set of 101 raster maps showing predicted potential distributions in Poland for modelled candidate species. Each figure displays continuous climatic suitability and the subset of grid cells exceeding a 10th-percentile training presence threshold. [file peerj-14-21492-s012.zip › Figure_SDM_poland_rank003_Paradiacheopsis_solitaria_Nann_Bremek_Nann_Bremek_1967_MaxEnt_logistic.png]

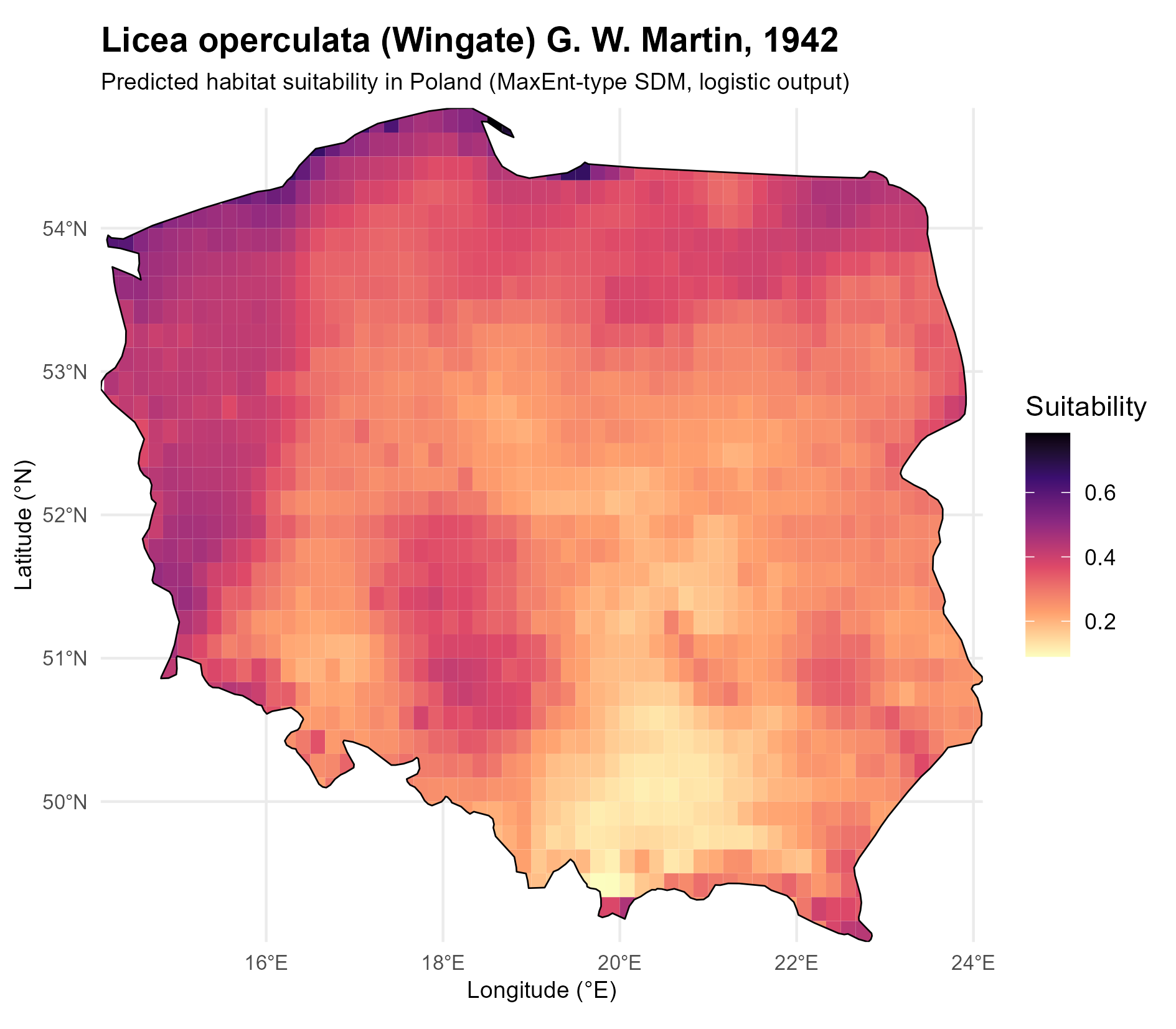

Supplement: Supplemental Information 12 — Set of 101 raster maps showing predicted potential distributions in Poland for modelled candidate species. Each figure displays continuous climatic suitability and the subset of grid cells exceeding a 10th-percentile training presence threshold. [file peerj-14-21492-s012.zip › Figure_SDM_poland_rank002_Licea_operculata_Wingate_G_W_Martin_1942_MaxEnt_logistic.png]

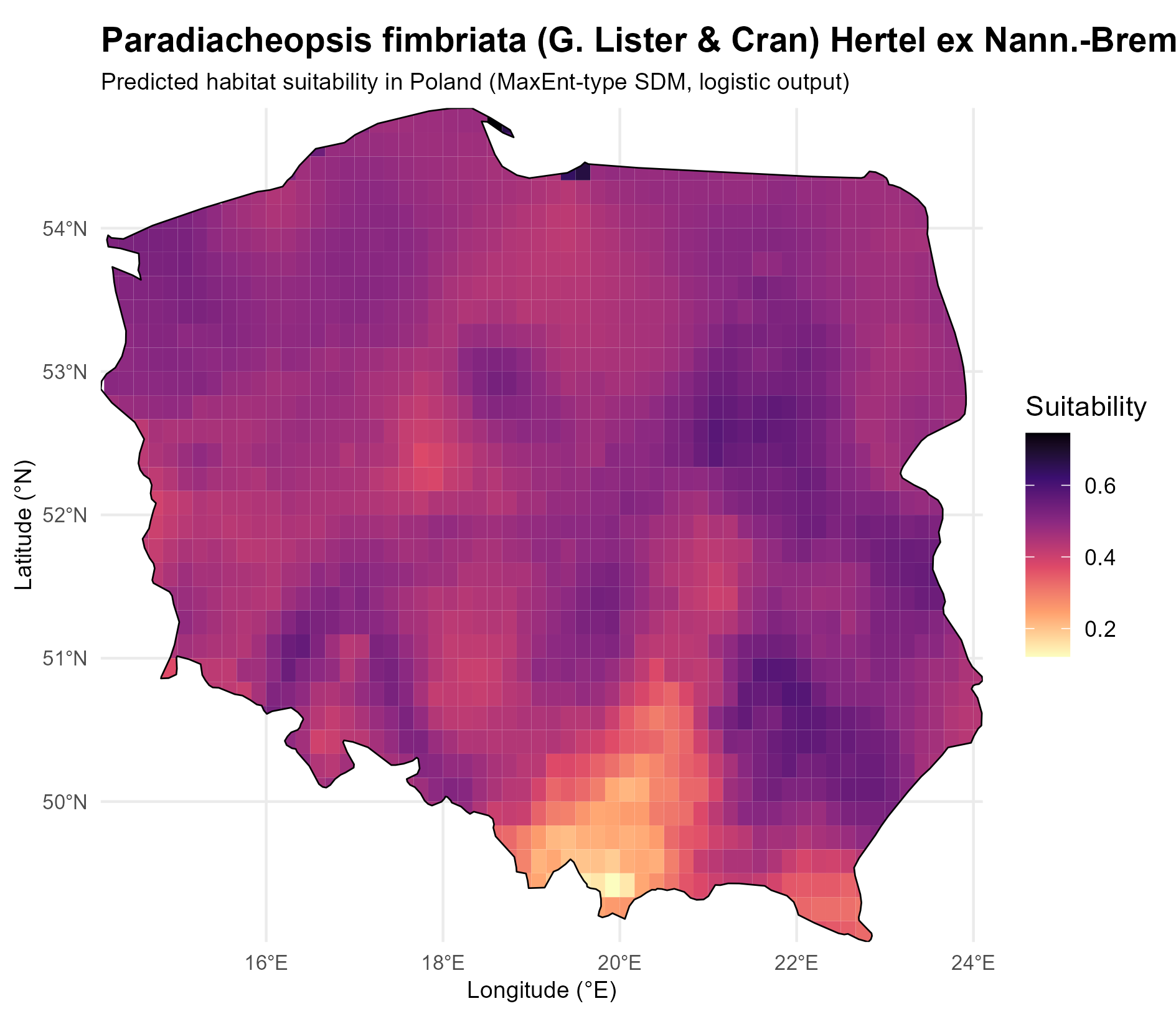

Supplement: Supplemental Information 12 — Set of 101 raster maps showing predicted potential distributions in Poland for modelled candidate species. Each figure displays continuous climatic suitability and the subset of grid cells exceeding a 10th-percentile training presence threshold. [file peerj-14-21492-s012.zip › Figure_SDM_poland_rank001_Paradiacheopsis_fimbriata_G_Lister_Cran_Hertel_ex_Nann_Bremek_1975_MaxEnt_logistic.png]

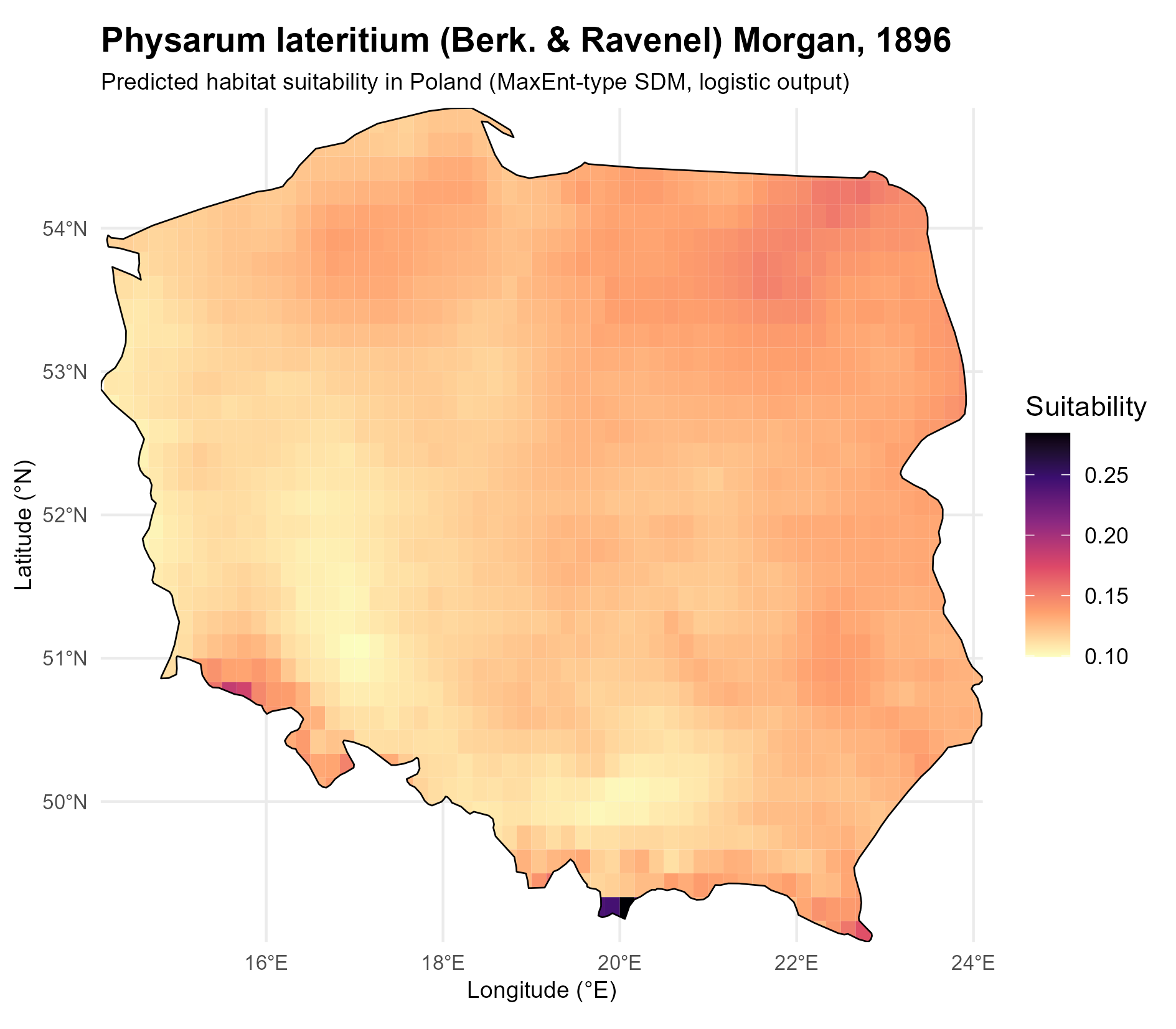

Supplement: Supplemental Information 12 — Set of 101 raster maps showing predicted potential distributions in Poland for modelled candidate species. Each figure displays continuous climatic suitability and the subset of grid cells exceeding a 10th-percentile training presence threshold. [file peerj-14-21492-s012.zip › Figure_SDM_poland_rank101_Physarum_lateritium_Berk_Ravenel_Morgan_1896_MaxEnt_logistic.png]

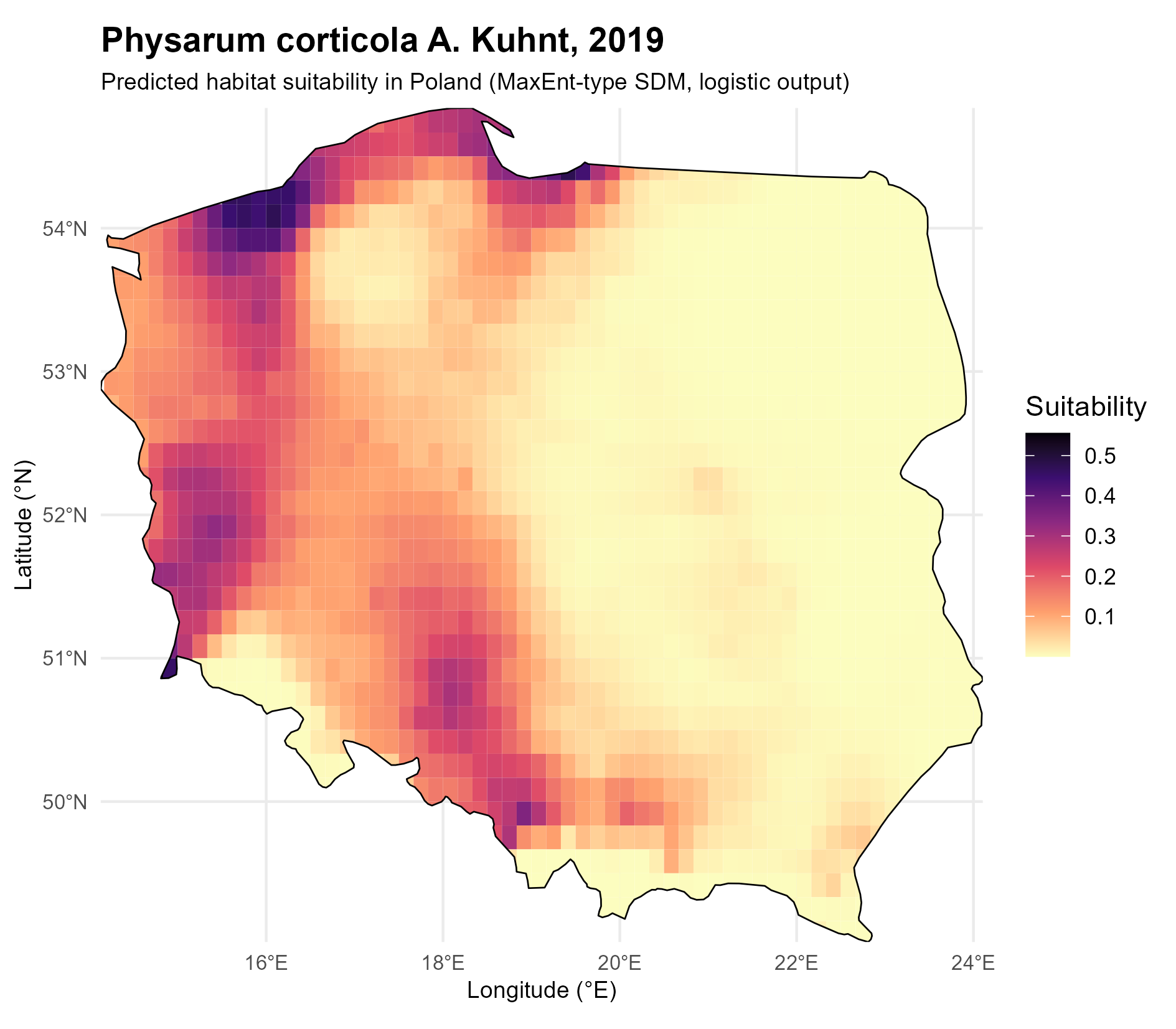

Supplement: Supplemental Information 12 — Set of 101 raster maps showing predicted potential distributions in Poland for modelled candidate species. Each figure displays continuous climatic suitability and the subset of grid cells exceeding a 10th-percentile training presence threshold. [file peerj-14-21492-s012.zip › Figure_SDM_poland_rank100_Physarum_corticola_A_Kuhnt_2019_MaxEnt_logistic.png]
