# Supplementary figures and images for: Recorded and predicted occurrence of slime moulds (Eumycetozoa) in Poland from Central and Eastern European data (part 2 of 2)
Source: PeerJ. 2026 Jul 9;14:e21492. doi: 10.7717/peerj.21492 (PMC13356830; doi:10.7717/peerj.21492)

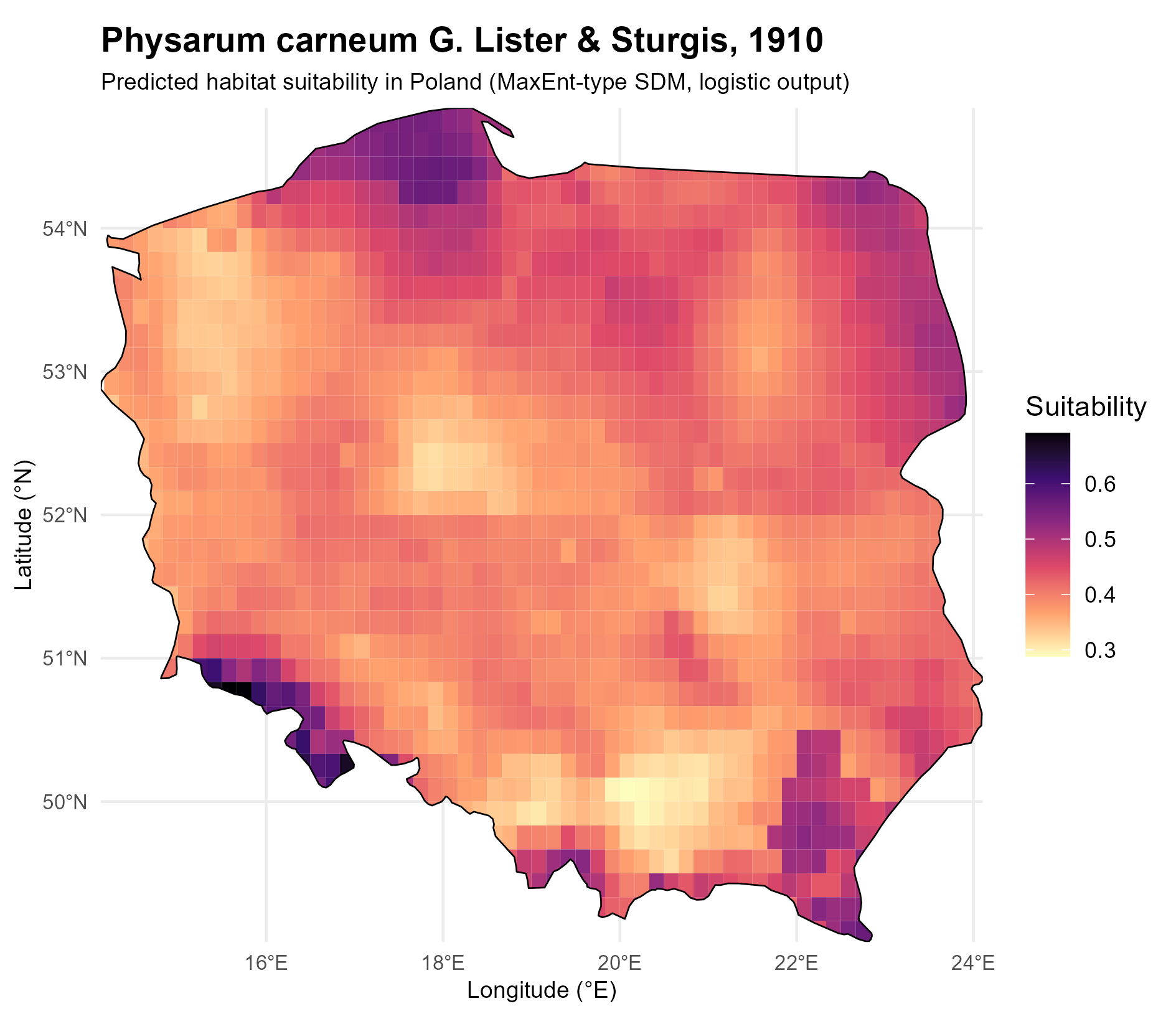

Supplement: Supplemental Information 12 — Set of 101 raster maps showing predicted potential distributions in Poland for modelled candidate species. Each figure displays continuous climatic suitability and the subset of grid cells exceeding a 10th-percentile training presence threshold. [file peerj-14-21492-s012.zip › Figure_SDM_poland_rank099_Physarum_carneum_G_Lister_Sturgis_1910_MaxEnt_logistic.png]

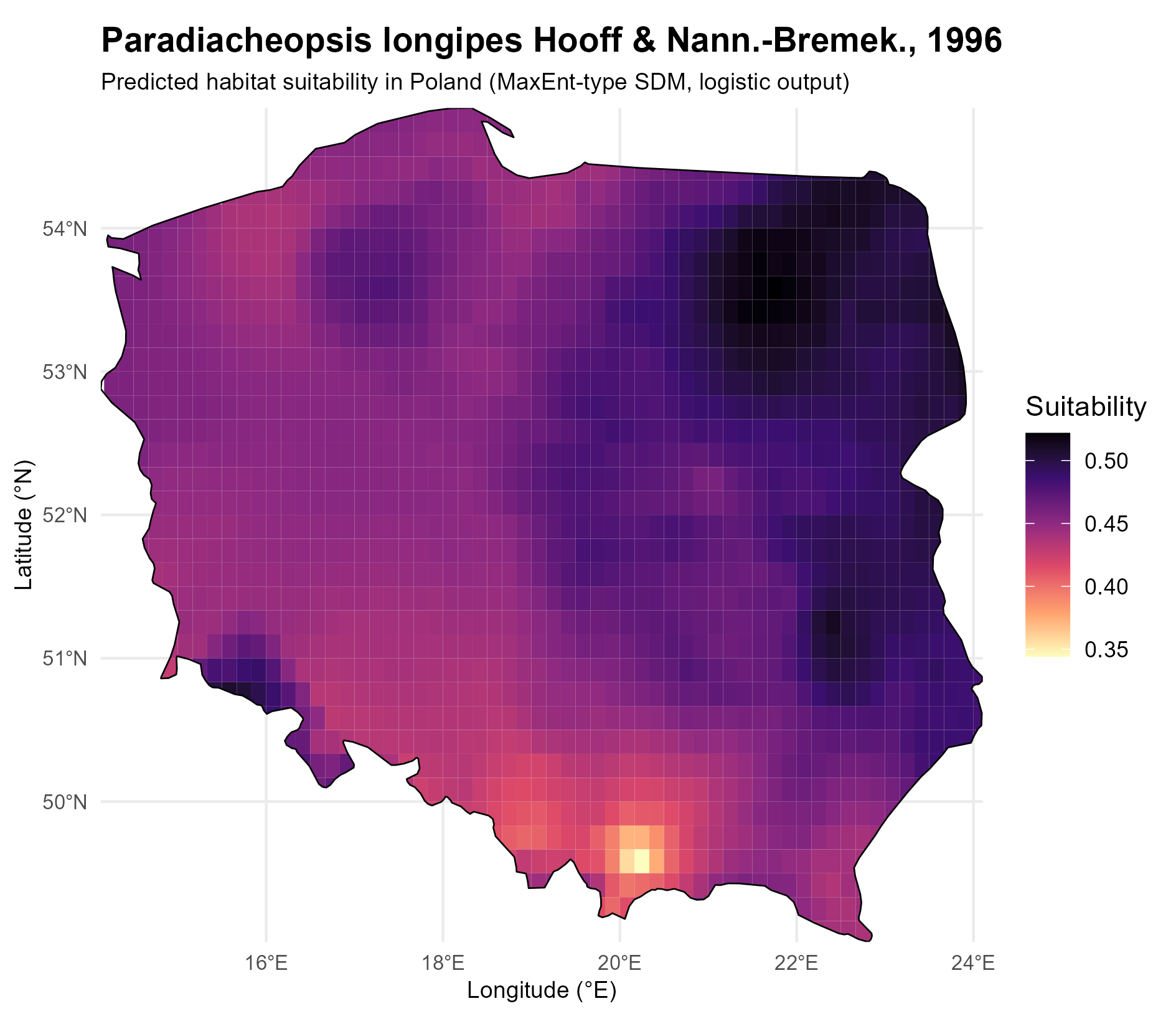

Supplement: Supplemental Information 12 — Set of 101 raster maps showing predicted potential distributions in Poland for modelled candidate species. Each figure displays continuous climatic suitability and the subset of grid cells exceeding a 10th-percentile training presence threshold. [file peerj-14-21492-s012.zip › Figure_SDM_poland_rank098_Paradiacheopsis_longipes_Hooff_Nann_Bremek_1996_MaxEnt_logistic.png]

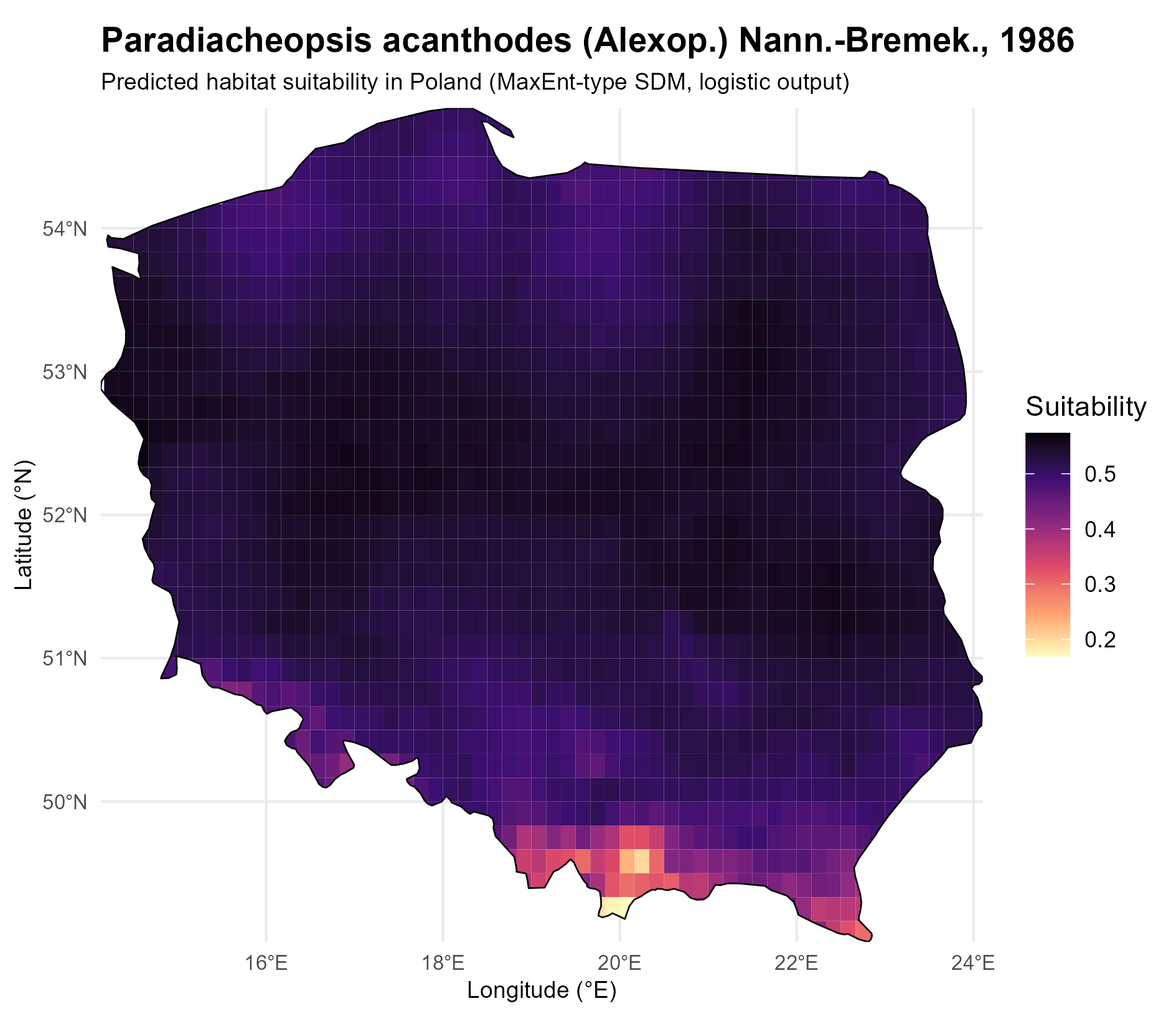

Supplement: Supplemental Information 12 — Set of 101 raster maps showing predicted potential distributions in Poland for modelled candidate species. Each figure displays continuous climatic suitability and the subset of grid cells exceeding a 10th-percentile training presence threshold. [file peerj-14-21492-s012.zip › Figure_SDM_poland_rank097_Paradiacheopsis_acanthodes_Alexop_Nann_Bremek_1986_MaxEnt_logistic.png]

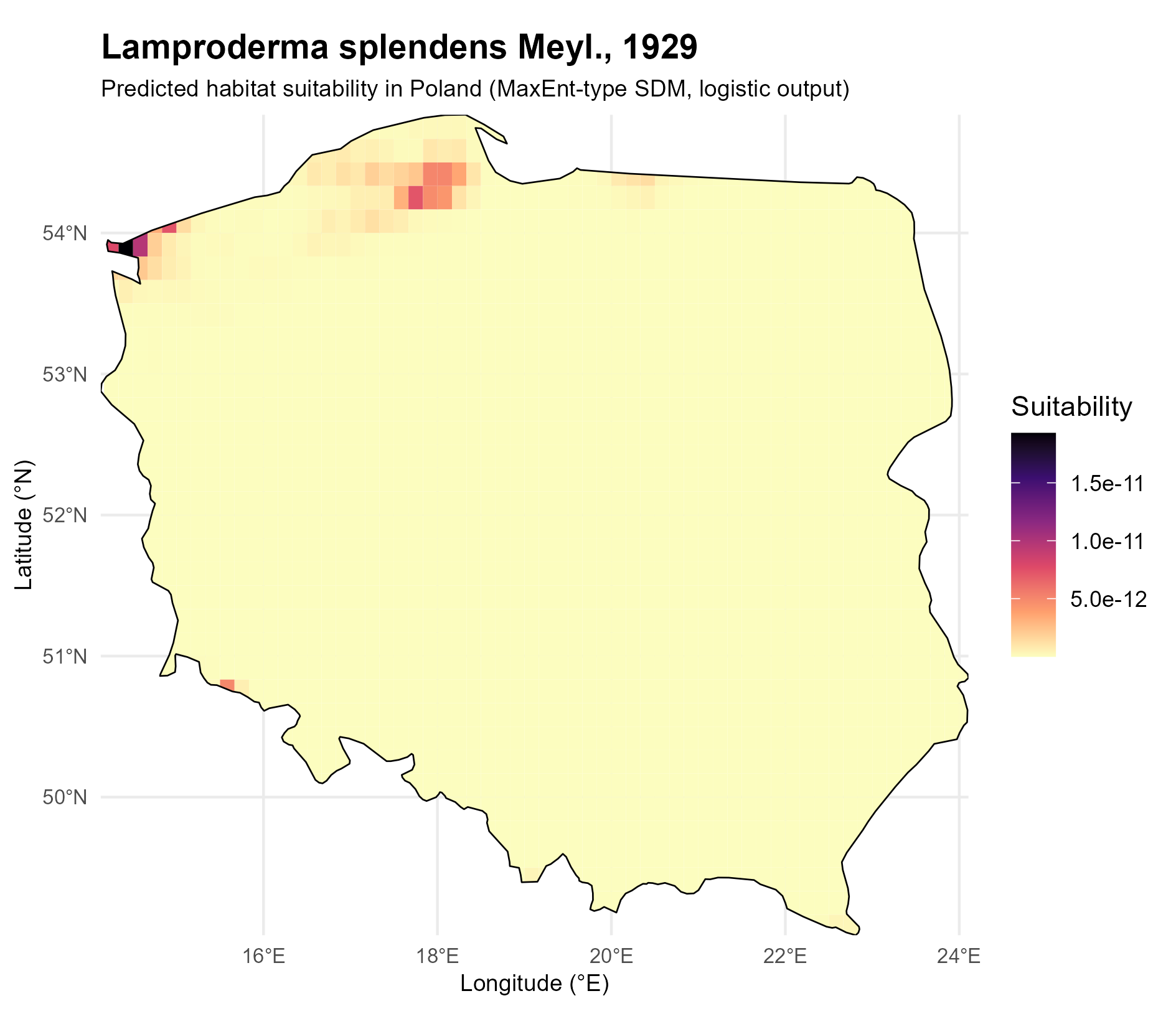

Supplement: Supplemental Information 12 — Set of 101 raster maps showing predicted potential distributions in Poland for modelled candidate species. Each figure displays continuous climatic suitability and the subset of grid cells exceeding a 10th-percentile training presence threshold. [file peerj-14-21492-s012.zip › Figure_SDM_poland_rank096_Lamproderma_splendens_Meyl_1929_MaxEnt_logistic.png]
